# Barcoding the butterflies of southern South America: Species delimitation efficacy, cryptic diversity and geographic patterns of divergence

**DOI:** 10.1371/journal.pone.0186845

**Published:** 2017-10-19

**Authors:** Pablo D. Lavinia, Ezequiel O. Núñez Bustos, Cecilia Kopuchian, Darío A. Lijtmaer, Natalia C. García, Paul D. N. Hebert, Pablo L. Tubaro

**Affiliations:** 1 Museo Argentino de Ciencias Naturales ‘Bernardino Rivadavia’ (MACN-CONICET), Buenos Aires, Argentina; 2 Laboratorio de Biología de la Conservación, Centro de Ecología Aplicada del Litoral (CECOAL-CONICET), Corrientes, Argentina; 3 Centre for Biodiversity Genomics, University of Guelph, Guelph, Ontario, Canada; Charles University, CZECH REPUBLIC

## Abstract

Because the tropical regions of America harbor the highest concentration of butterfly species, its fauna has attracted considerable attention. Much less is known about the butterflies of southern South America, particularly Argentina, where over 1,200 species occur. To advance understanding of this fauna, we assembled a DNA barcode reference library for 417 butterfly species of Argentina, focusing on the Atlantic Forest, a biodiversity hotspot. We tested the efficacy of this library for specimen identification, used it to assess the frequency of cryptic species, and examined geographic patterns of genetic variation, making this study the first large-scale genetic assessment of the butterflies of southern South America. The average sequence divergence to the nearest neighbor (i.e. minimum interspecific distance) was 6.91%, ten times larger than the mean distance to the furthest conspecific (0.69%), with a clear barcode gap present in all but four of the species represented by two or more specimens. As a consequence, the DNA barcode library was extremely effective in the discrimination of these species, allowing a correct identification in more than 95% of the cases. Singletons (i.e. species represented by a single sequence) were also distinguishable in the gene trees since they all had unique DNA barcodes, divergent from those of the closest non-conspecific. The clustering algorithms implemented recognized from 416 to 444 barcode clusters, suggesting that the actual diversity of butterflies in Argentina is 3%–9% higher than currently recognized. Furthermore, our survey added three new records of butterflies for the country (*Eurema agave*, *Mithras hannelore*, *Melanis hillapana*). In summary, this study not only supported the utility of DNA barcoding for the identification of the butterfly species of Argentina, but also highlighted several cases of both deep intraspecific and shallow interspecific divergence that should be studied in more detail.

## Introduction

Lepidopterans constitute one of the most diverse groups of insects with almost 160,000 species described worldwide and an estimated diversity of nearly half a million species [1,2]. While moths comprise the majority of Lepidoptera diversity, butterflies (ca. 20,000 species in the world) have received more attention [1,2]. In fact, butterflies are one of the best studied groups of insects, being model organisms in numerous areas such as evolutionary and developmental biology, ecology, genetics and animal behavior [3,4]. The highest diversity of Lepidoptera occurs in the Neotropics with around 45,000 species [5], 17% of which (ca. 8,000 species) are butterflies [6]. Most past studies aiming at elucidating the evolutionary history of Neotropical butterflies have examined the Amazon basin and the tropical Andes, where diversity is highest [7–10]. By contrast, with the exception of southeastern Brazil [11–13], much less is known about the butterfly communities of southern South America.

At least 1,253 species occur in Argentina [14], including representatives of 507 genera and all seven families of butterflies (superfamily Papilionoidea, *sensu* [1,15]; but see also [16]). Around 70% of these species occur in the Misiones province of northeastern Argentina [17]. This remarkable richness is due to the presence in this area of the southernmost portion of the Atlantic Forest, a biodiversity hotspot and priority area for conservation [18]. Unfortunately, 90% of the Atlantic Forest’s original extension has been lost as a consequence of anthropogenic transformation. The majority of what remains exists as small (< 50 ha) fragmented patches, with the exception of the Paraná Forest in Misiones which is the largest area of continuous Atlantic Forest that still exists (ca. 1 million ha; [19,20]).

DNA barcodes [21], a specimen identification system based on short standardized genetic markers, has proven to be extremely effective for species discrimination in many groups of animals in general [22–24], and especially in Lepidoptera [25–29]. This tool is based on the observation that genetic divergence in the mitochondrial cytochrome *c* oxidase subunit I gene (COI, the marker used for most metazoans [30]) is higher among species than within them [21,22]. Consequently, a species name can be assigned to sequences from unidentified specimens by comparing them against a DNA barcode reference library composed of sequences of known taxonomic origin [30]. However, DNA barcodes are now widely used not only for specimen identification, but also as tools in ecology, evolution and conservation [30]. In particular, their association with clustering algorithms developed to delineate putative species based on sequence data (e.g. [31,32]), has proven the utility of DNA barcodes for accelerating biodiversity inventories [33,34], speeding taxonomic workflows [35], and for the study of cryptic diversity and geographic patterns of genetic variation [36–41]. At the same time, the results of all these applications can be used together with traditional taxonomy to establish conservation programs for particular taxonomic groups and biodiversity hotspots [30,42,43].

Despite the existence of several illustrated field guides for the identification of the local species of butterflies based on external morphology [17,44], lepidopterans have attracted little genetic investigation in Argentina (but see [45] for a recent exception in moths). Even though wing coloration patterns seem to be effective for the identification of most butterfly species, genetic tools such as DNA barcoding can help to detect and describe cryptic diversity that would go unnoticed when assessing only morphological characters, even in presumably well-investigated groups [23,40,41]. In this context, we performed a genetic examination of over one third of all the butterfly species occurring in Argentina with a focus on the Atlantic Forest. First, we assembled a DNA barcode reference library and subsequently tested its effectiveness for species discrimination. Sequence data was then used to assess the frequency of cryptic taxa and to explore phylogeographic patterns of intraspecific variation. This study constitutes the first large-scale genetic assessment of the butterflies of southern South America in general and of Argentina in particular.

## Materials and methods

### Sampling

**Fig 1:**
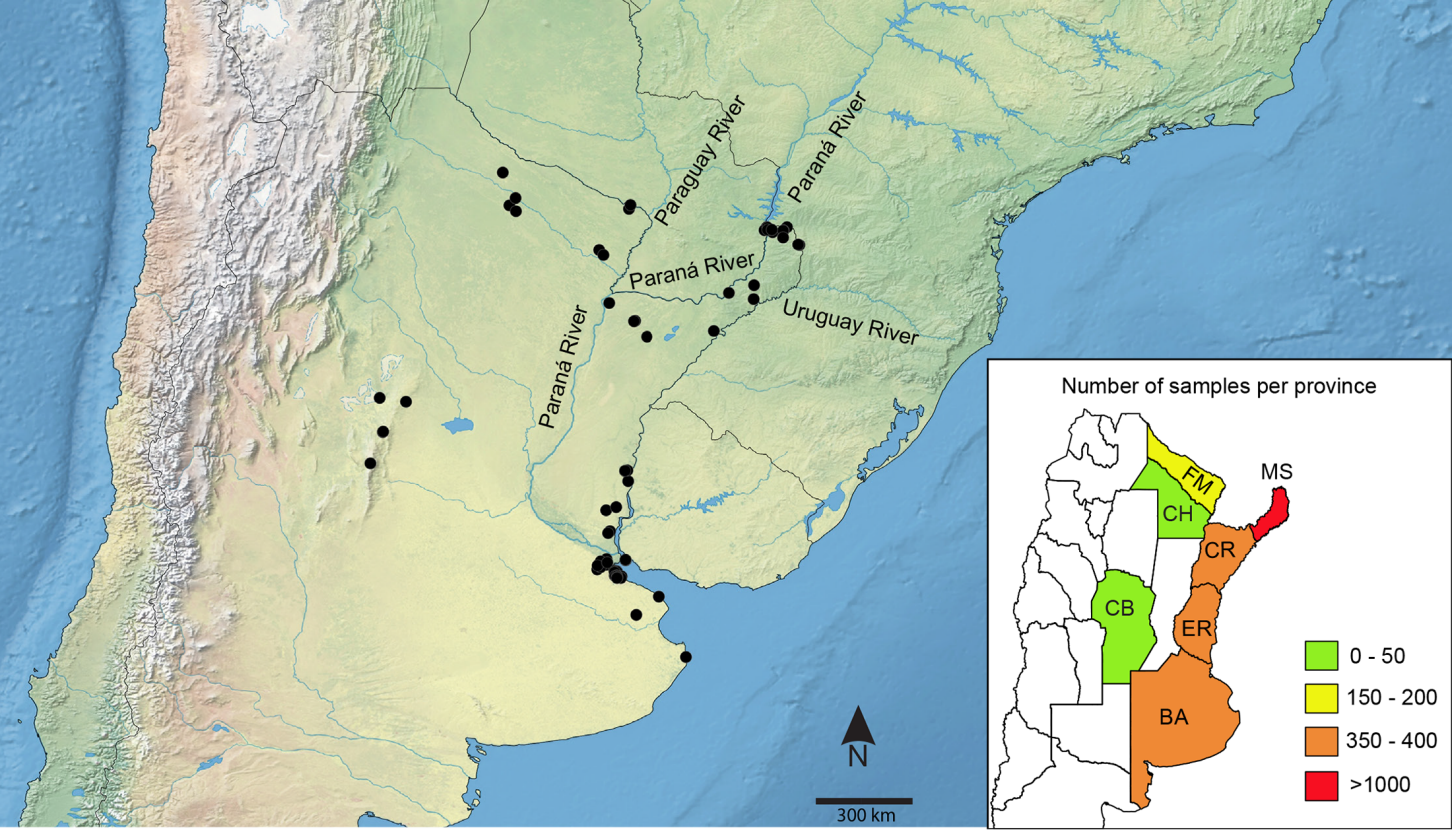
Sampling localities for the 2,161 butterflies collected for this study. The three major rivers within the Del Plata Basin are indicated. The inset (lower right) shows the total number of specimens collected in each province: BA, Buenos Aires; CH, Chaco; CB, Córdoba; CR, Corrientes; ER, Entre Ríos; FM, Formosa; MS, Misiones. More detailed information can be found in Tables A and B in S1 Supporting Information.

Collection was performed using both insect nets and fruit bait traps between 2010 and 2015 in seven different provinces of northeastern and central Argentina (Buenos Aires, Chaco, Córdoba, Corrientes, Entre Ríos, Formosa and Misiones; Fig 1 and Tables A and B in S1 Supporting Information). All field work was conducted with the authorization of the National Parks Administration from Argentina and the Offices of Fauna of each Argentinian province, who granted all collection permits needed. Specimens were deposited in the Entomological Collection of the Museo Argentino de Ciencias Naturales “Bernardino Rivadavia” (MACN). All specimens (listed in Table A in S1 Supporting Information) were identified by ENB based on external morphology (mainly wing coloration pattern) and following different illustrated field guides to the butterflies of Argentina [17,44,46,47] and the “Butterflies of America” website [48] for some particular cases. Only 13 records (0.6% of the total) lacked a species name. Specimens were obtained from six of the seven families of butterflies [1,15] present in Argentina. The missing family (Hedylidae) is known from only a single species in Argentina and it does not occur in the area that was sampled. All specimen data, including images of the vouchers, are available in the public data set “DS-BUNEACAR” (dx.doi.org/10.5883/DS-BUNEACAR) on BOLD (www.boldsystems.org, [49]). A summarized version of that information can also be found in Tables A and B in S1 Supporting Information.

### Laboratory procedures

Genomic DNA was obtained from one leg of each specimen. Tissue lysis, DNA extraction and amplification were performed at the MACN or the Canadian Centre for DNA Barcoding (CCDB) following standard protocols [50,51]. The 658-bp barcode region of the COI was amplified using either the LepF1 and LepR1 pair of primers [52] or the primer cocktails C_LepFolF and C_LepFolR [52,53]. Sequencing was performed bidirectionally at the CCDB. Sequences were edited and aligned using CodonCode Alligner 4.0.4 (CondonCode Corporation, Dedham, MA) and translated into an amino acid sequence to verify the lack of stop codons. They were also visually examined to assess the presence of indels in the alignment using MEGA 5.0 [54]. Sequence information is available in the public data set “DS-BUNEACAR” on BOLD, and in Table A in S1 Supporting Information. COI sequences can also be found in GenBank under accession numbers MF545386–MF547405.

### Genetic distances and gene trees

Analyses only considered sequences longer than 500 bp and with less than 1% ambiguous calls. The sole exception (one sequence from *Hamadryas feronia* with 1.22% missing data) was retained in the final data set as it represented the only record for that species. Genetic distances were computed within and among species both as uncorrected divergences (p-distances) and using the Kimura 2-parameter (K2P) distance model [55]. Since results were almost identical with the two distance metrics and K2P is the standard substitution model applied in DNA barcoding studies, we report only the results obtained with K2P (except for the ABGD analyses; see below). In order to assess the existence of a separation between intra- and interspecific diversity (i.e. the barcode gap), we computed and compared the distance of each sequence to its furthest conspecific and to its nearest non-conspecific (i.e. nearest neighbor). All genetic distances were obtained with the package SPIDER [56] in R 3.3.1 [57]. For those species in which intraspecific variation could not be assessed because they were represented by a single specimen (i.e. singletons), we followed a “Tree Based Identification” approach to examine their distinctiveness [58]. Basically, a singleton was considered distinguishable from other species when showing unique (i.e not shared) DNA barcodes that allowed their separation from the nearest non-conspecific in the gene trees (see next).

We estimated a Neighbor-Joining (NJ) gene tree on BOLD (K2P and pairwise deletion options were selected). Node support was computed in MEGA through 1000 bootstrap pseudoreplicates. In addition to the NJ tree which we used as our reference topology, we built a maximum likelihood (ML) gene tree with RAxML 8.1.22 [59]. The latter analysis consisted of 100 independent ML tree searches under the GTRGAMMA model of evolution. Support values were derived from 1000 rapid bootstrap pseudoreplicates and printed on the best tree found among the ML searches. We emphasize that our goal was not to infer the phylogenetic relationships among the species analyzed, but to assess the distinctiveness of singletons and to obtain support values for terminal nodes and intraspecific clades.

### Sequence-based specimen identifications

To test the utility of the DNA barcode library for the discrimination of the butterflies of Argentina we simulated a sequence-based identification process [60]. Each sequence was treated as an unknown specimen and queried against our library to assign a species name based on three different criteria: Best Match (BM) and Best Close Match (BCM) as defined by Meier et al. [61], and the BOLD Identification Criterion (BIC) as implemented by the BOLD ID engine [49]. The BM criterion assigns a species name to the query according to the closest match available in the library regardless of the genetic divergence. The BCM approach incorporates a divergence threshold so that a species name is assigned to the query based on the closest match below the threshold. If a query has two or more equally close matches from different species within the threshold the identification is considered ambiguous, while if the closest match involves a sequence from another species the identification is incorrect. Queries will remain unidentified when the closest match lies outside the threshold (same for the BIC). The BIC is stricter as it considers all sequences within the threshold. Therefore, a correct identification is only recovered when all sequences below the threshold derive from the same species as the query, while the identification is considered as ambiguous when sequences from multiple species appear within the threshold. Finally, the identification is incorrect when all sequences below the threshold correspond to another species. All simulations were carried out in SPIDER.

We employed four different thresholds for the BCM and BIC criteria: 1) the 95^th^ percentile of all intraspecific distances [61], 2) BOLD’s ID engine threshold of 1% [49], 3) the genetic distance that minimizes the sum of false positive and false negative identifications (i.e. the cumulative error), and 4) the lowest value in a density plot of all genetic distances which should correspond to the transition between intra- and interspecific distances. The latter two values were obtained with SPIDER using the functions “threshVal” and “localMinima” respectively. Singletons were not used as queries but they remained as potential matches for other sequences. All analyses were performed using K2P and p-distances, but since results were identical we report only the former.

### MOTUs delineation analyses

To assess the presence of cryptic taxa, we implemented three different clustering algorithms commonly used for species delimitation in DNA barcoding studies [33,35]: Automatic Barcode Gap Discovery (ABGD, [32]), statistical parsimony networks [62] as implemented in TCS [63], and the Refined Single Linkage algorithm (RESL, [31]). Basically, the three methods partition the alignment of DNA barcodes into Molecular Operational Taxonomic Units (MOTUs) based on sequence similarity (for a more detailed explanation on these algorithms see the S1 Appendix and the literature cited above). ABGD was run on command line and using K2P and uncorrected pairwise distance matrices as inputs and testing two relative gap width values (X = 1.5, 1.0). We recorded all partitioning schemes for a range of prior intraspecific divergence (P) values between 0.001 (0.1%) and 0.1 (10%). For TCS we registered the results for ten different parsimony limit values (90%-99%). Lastly, RESL is the method employed to assign all COI barcode sequences on BOLD into genetic clusters (BINs), which are the basis of the Barcode Index Number system [31]. BINs are delineated based on all COI sequences uploaded to the platform, so they are not strictly comparable with the MOTUs obtained with the two other methods. Therefore, we applied the RESL algorithm exclusively to our data set using the Cluster Sequences Analysis tool available on BOLD (http://www.boldsystems.org). However, to assess how the addition of other sequences can modify the outcome of the RESL algorithm, we compared the standard BIN assignments available on BOLD with the results generated using RESL with our data only.

For all methods we compared MOTU counts to the number of reference species in our data set, and tested the correspondence between species and MOTU boundaries. Based on the latter, we assigned each species to one of four categories: MATCH, SPLIT, MERGE or MIXTURE [31]. A MATCH occurs when all the specimens from a particular species are grouped into a single MOTU. A SPLIT is registered when the sequences from a species are divided into two or more MOTUs, while a MERGE is recorded when the sequences from different species are grouped into the same MOTU. Lastly, when a species is involved in both a MERGE and a SPLIT, it is placed in the MIXTURE category.

### Geographic patterns of genetic variation

We examined a) species with high intraspecific distance (i.e. maximum divergence equal or higher than the 95^th^ percentile of all intraspecific distances), and b) species that were split by any of the clustering algorithms mentioned above. Additionally, we tested the correlation between genetic and geographic distances by performing a Mantel test for each of the 42 species represented by 10 or more individuals collected from sites at least 275 km apart (range of maximum distances: 275 km– 1280 km; Table F in S1 Supporting Information). These tests were performed in GenAlex 6.5 [64] and their significance was evaluated with 999 permutations.

## Results

### Sampling and final data set

We collected 2,161 specimens from central and northwestern Argentina (Fig 1) representing 252 genera and 429 species (Table 1, Tables A and B in S1 Supporting Information). A sequence could not be obtained for 134 specimens (6% of the total), and after discarding four low quality sequences and three cases of contamination, the final data set consisted of 2,020 sequences from 248 genera and 417 species (Table 1; Table A in S1 Supporting Information). On average, 4.8 sequences were analyzed per species (range 1–27), with 305 species represented by more than one specimen. Singletons (112) represented only 5.5% of the sequences and 27% of the species analyzed. No stop codons were found, and only one species (*Eryphanis reevesii*) possessed a deletion, but it was 3 bp in length and did not alter the reading frame.

**Table 1:** Summary of the specimens and species sampled and sequenced for the seven families of butterflies known from Argentina.

Family	Species in Argentina	Individuals/species sampled	Individuals/species sequenced	Sequencing success for individuals (%)/ species(%)	Species covered (%)
Hesperiidae	504	599/161	579/159	96.7/98.8	31.9
Lycaenidae	195	124/42	121/42	97.6/100.0	21.5
Nymphalidae	332	1047/157	959/151	91.6/96.2	47.3
Papilionidae	34	67/13	63/12	94.0/92.3	38.2
Pieridae	72	198/24	175/21	88.4/87.5	33.3
Riodinidae	115	126/32	123/32	97.6/100.0	27.8
Hedylidae	1	–	–	–	–
Total	1253	2161/429	2020/417	93.5/97.2	34.2

Sequencing success is based on the final data set used for the analyses.

### Genetic distances and gene trees

**Fig 2:**
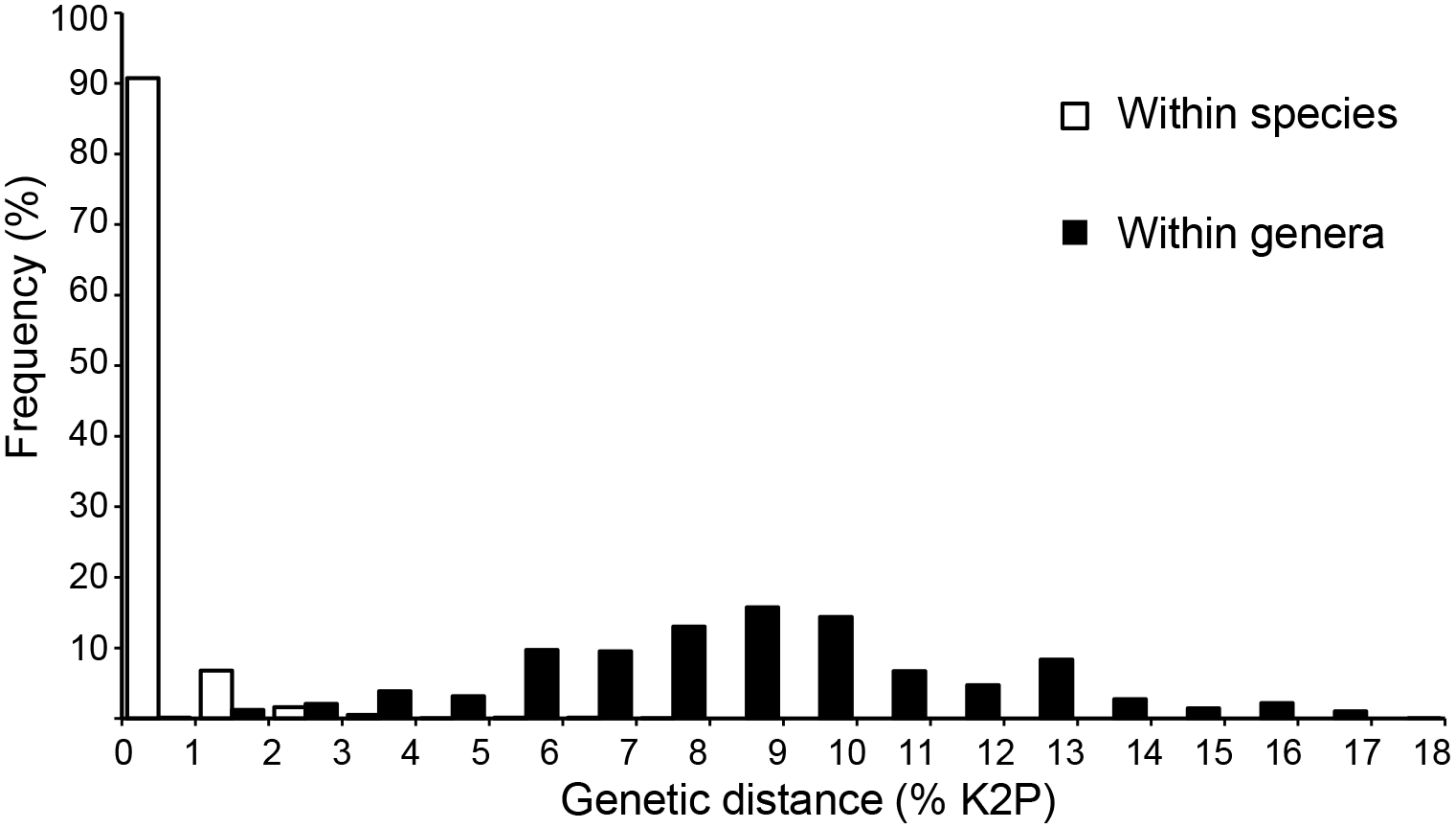
Frequency histogram of COI sequence divergence (K2P) within species and among congeneric species of butterflies of Argentina.

**Fig 3:**
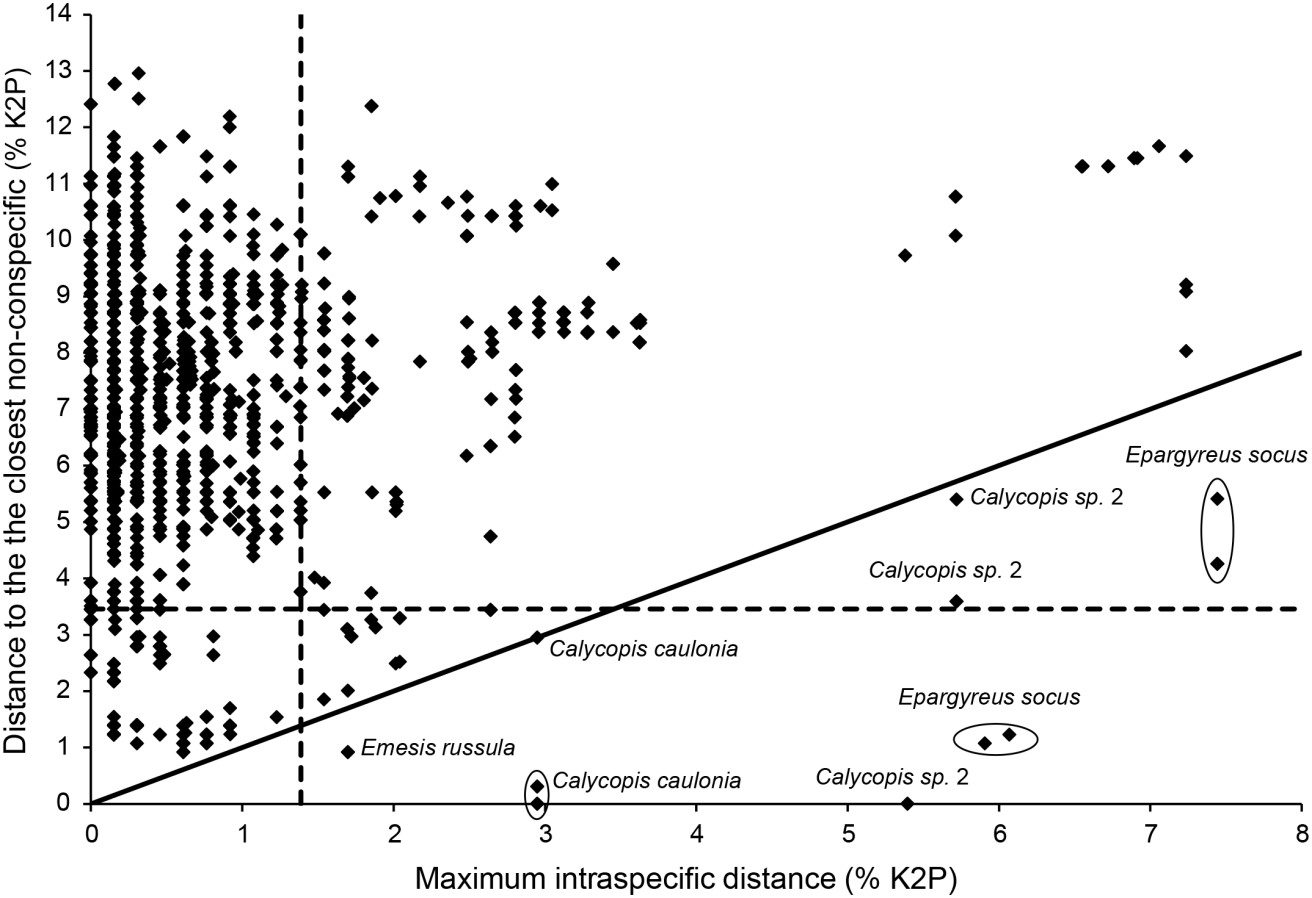
Barcode gap analysis for 305 butterfly species of Argentina represented by two or more COI sequences. Each dot represents a sequence. Dots below the diagonal correspond to specimens for which maximum intraspecific distance was higher than the distance to the nearest non-conspecific (i.e. nearest neighbor). The vertical dashed line shows the 95^th^ percentile of all intraspecific distances (1.39%), while the horizontal line corresponds to the lower 5% of all congeneric distances (3.46%).

Based on 7,983 comparisons performed among individuals of the 305 species (190 genera) represented by two or more sequences (6 individuals per species on average), the mean intraspecific divergence was 0.31% (range 0.00%–7.24%; Fig 2). By comparison, the average distance among congeneric species was 7.18% (range 2.84%–14.45%; Fig 2) based on 10,464 comparisons among 382 pairs of congeneric species (253 species from 84 genera). More importantly, the mean divergence to the nearest neighbor (i.e. minimum interspecific distance) was 6.91% (min. 0.00%, max. 13.14%), ten times larger than the mean distance (0.69%) to the furthest conspecific (min. 0.00%, max. 7.44%). As a consequence, a barcode gap was observed for all species with two or more individuals (Fig 3), with the exception of *Calycopis sp*. 2, *C*. *caulonia*, *Emesis russula* and *Epargyreus socus*. These species were paraphyletic in the NJ and ML gene trees (S1 and S2 Figs), and some individuals of *Calycopis sp*. 2 and *C*. *caulonia* even shared their barcode sequences (Fig 3). Excluding this single case of barcode sharing, the minimum interspecific distance was 0.92% between *E*. *russula* and *E*. *mandana*. In addition, three other species (*Actinote pellenea*, *Ministrymon cruenta* and *Cymaenes laureolus*) were also paraphyletic in the ML tree (but not in the NJ tree). Lastly, all singletons had unique DNA barcodes that made them clearly distinguishable from the nearest non-conspecific in the gene trees (S1 and S2 Figs). Consistently, the mean distance to the nearest neighbor averaged 6.83% across all singletons (min. 1.23%, max. 13.14%), with 94% of these species (105 taxa) showing minimum interspecific divergence above 3.46% (lower 5% of all congeneric distances; Table C in S1 Supporting Information).

### Sequence-based specimen identification simulation

Based on 1,908 queried sequences from the 305 species with two or more individuals, the BM criteria generated 99.42% of correct and 0.21% of incorrect identifications, while 0.37% of the queries received an ambiguous assignment (Table 2). For the BCM and BIC approaches we implemented four thresholds. The 95^th^ percentile of all intraspecific distances (1.39%) produced 98.43% correct, 0.10% incorrect and 0.37% ambiguous identifications with BCM, with 1.10% unidentified queries (Table 2). Results with the BIC approach varied only slightly, with a lower percentage (96.07%) of correct identifications, a higher rate (2.83%) of ambiguous identifications, no incorrect assignments, and the same amount of unidentified queries than with BCM (Table 2). For both BCM and BIC approaches, BOLD’s ID engine threshold of 1% produced no incorrect identifications, a low percentage of ambiguous identifications (0.37% with BCM and 0.58% with BIC), and a slightly higher percentage (1.62%) of unidentified queries than with the 1.39% threshold (Table 2). This resulted in around 98% of correct identifications, a percentage highly similar to that obtained with the 95^th^ percentile of all intraspecific distances. The “treshVal” function suggested a threshold between 0.8% and 0.9% (Fig A in S2 Supporting Information), so we used the mean value of 0.85% for the simulations. With this threshold, BCM and BIC returned results identical to those obtained with BCM using BOLD’s ID engine threshold (Table 2). The “localMinima” function returned a higher threshold of 2.06% (Fig B in S2 Supporting Information). Using this threshold and BCM, the percentage of correct identifications (99%) was the second highest after that of the BM criterion, while it was the lowest (95.59%) among all methods when using the more rigorous BIC (Table 2). With the latter approach and a 2.06% threshold, the number of ambiguous identifications increased to 3.88%, versus just 0.37% with BCM. Lastly, 0.53% of the queries remained unidentified using this threshold with both approaches (Table 2).

**Table 2:** Summary of the results from the sequence-based identification simulations.

Identification/Criterion	BM	95% percentile of intraspecific distances (1.39%)		"threshVal" (0.85%)		"localMinima" (2.06%)		BOLD's threshold (1.00%)	
		BCM	BIC	BCM	BIC	BCM	BIC	BCM	BIC (1.00%)
Correct	1897 (99.42%)	1878 (98.43%)	1833 (96.07%)	1870 (98.01%)	1870 (98.01%)	1889 (99%)	1824 (95.59%)	1870 (98.01%)	1866 (97.80%)
Incorrect	4 (0.21%)	2 (0.10%)	–	–	–	2 (0.10%)	–	–	–
Ambiguous	7 (0.37%)	7 (0.37%)	54 (2.83%)	7 (0.37%)	7 (0.37%)	7 (0.37%)	74 (3.88%)	7 (0.37%)	11 (0.58%)
No identification	–	21 (1.10%)	21 (1.10%)	31 (1.62%)	31 (1.62%)	10 (0.53%)	10 (0.53%)	31 (1.62%)	31 (1.62%)

A species name was assigned according to three criteria: Best Match (BM), Best Close Match (BCM) and BOLD Identification Criterion (BIC). Four threshold values (0.85%, 1.00%, 1.39% and 2.06%) were used for the BCM and BIC approaches. The total number of identifications within each category and the percentage they represent (values in parenthesis) are based on 1,908 queries (singletons were not used as queries but they remained as potential matches for other sequences).

### Assessment of cryptic diversity

**Fig 4:**
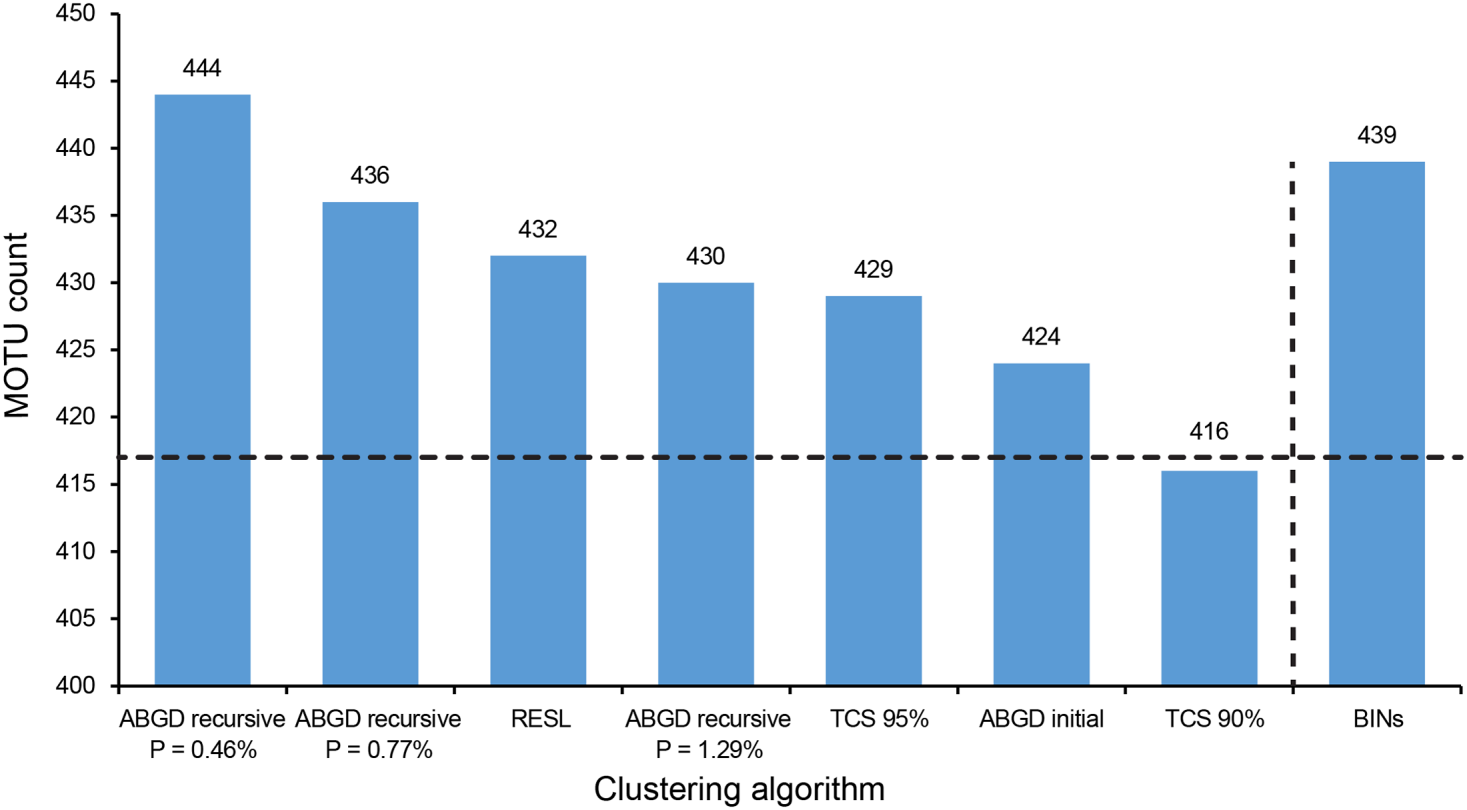
MOTU counts for eight partitioning schemes from the three clustering algorithms implemented. The horizontal line indicates the species count (417) based on current taxonomy.

The number of MOTUs delineated ranged from 416 to 444 depending on the method (Fig 4). The lowest count was delivered by TCS with the 90% cutoff value, while the recursive partitions of ABGD generated the maximum number of MOTUs. Since some methods produced a range of MOTU counts, we report all results (Tables C to E in S1 Supporting Information) but focus on certain partitioning schemes to simplify the comparisons.

**Fig 5:**
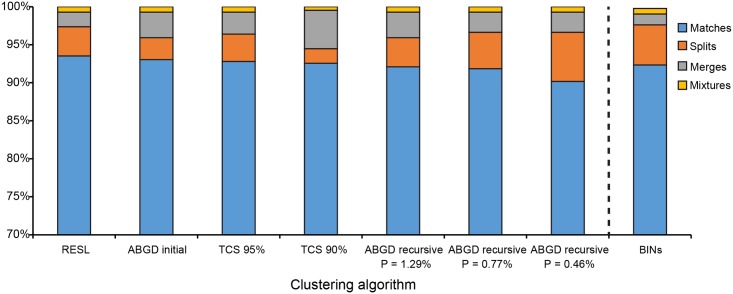
Congruence between species and MOTU boundaries for each of eight partitioning schemes from the three clustering algorithms implemented.

RESL produced 432 MOTUs (Fig 4, Table C in S1 Supporting Information) and 93.53% of MATCHES, with 16 species (3.84%) being split into two clusters (Table 3, Fig 5). Four pairs of species (1.92%) were merged into a single MOTU (Table 4), while three species (*Calycopis sp*. 2., *C*. *caulonia*, and *E*. *socus*) were involved in both merges and splits (i.e. MIXTURES). In comparison, our sequence records were assigned in June 2017 to 439 BINs on BOLD (Fig 4, Tables A and C in S1 Supporting Information). With the BIN system, MERGES (Table 4) decreased slightly (1.44%) while SPLITS (5.28%, Table 3) were higher, reducing the percentage of MATCHES (92.33%; Fig 5). MIXTURES remained the same (Fig 5).

**Table 3:** Species split into two or more MOTUs by one or more clustering algorithms.

Species	N	Max. distance	Geographic Pattern	RESL	TCS 90%	TCS 95%	ABGD initial	ABGD recursive P = 1.29	ABGD recursive P = 0.77	ABGD recursive P = 0.46	BIN
*Achlyodes busirus*	6	2.96	Split within Misiones	SP (2)	SP (2)	SP (2)	SP (2)	SP (2)	SP (2)	SP (2)	SP (2)
*Aphrissa statira*	8	0.62	Complex	MA	MA	MA	MA	MA	MA	SP (2)	MA
*Astraptes fulgerator*	3	1.23	Split within Misiones	MA	MA	MA	MA	MA	MA	MA	SP (2)
*Caeruleuptychia helena*	2	7.24	Split within Misiones	SP (2)	SP (2)	SP (2)	SP (2)	SP (2)	SP (2)	SP (2)	SP (2)
Calycopis caulonia*	7	2.95	Complex	MI (2)	ME	MI (2)	MI (2)	MI (2)	MI (2)	MI (2)	MI (2)
*Calycopis sp*. 2*	5	5.72	Split within Misiones	MI (3)	MI (3)	MI (3)	MI (3)	MI (3)	MI (3)	MI (3)	MI (3)
*Doxocopa agathina*	5	0.92	Split within Misiones	MA	MA	MA	MA	MA	SP (2)	SP (2)	MA
*Eantis thraso*	11	3.63	Complex	SP (2)	SP (2)	SP (2)	SP (2)	SP (3)	SP (3)	SP (3)	SP (3)
*Emesis diogenia*	7	0.61	Misiones vs Formosa	MA	MA	MA	MA	MA	MA	SP (2)	MA
Epargyreus socus*	5	7.44	Split within Misiones	MI (3)	MI (3)	MI (3)	MI (3)	MI (3)	MI (3)	MI (3)	MI (3)
*Eurema albula*	8	2.49	Complex	MA	MA	SP (2)	MA	SP (3)	SP (3)	SP (3)	MA
*Eurema elathea*	11	7.24	Complex	SP (2)	SP (2)	SP (2)	SP (2)	SP (2)	SP (2)	SP (2)	SP (2)
*Godartiana muscosa*	7	2.81	Split within Misiones	SP (2)	MA	SP (2)	SP (2)	SP (2)	SP (2)	SP (2)	SP (2)
*Gorgythion begga*	7	2.64	Misiones vs Entre Ríos	SP (2)	SP (2)	SP (2)	SP (2)	SP (2)	SP (2)	SP (2)	SP (2)
*Gorgythion beggina*	5	1.54	Complex	MA	MA	MA	MA	SP (2)	SP (2)	SP (2)	SP (2)
*Hamadryas amphinome*	5	1.39	Split within Misiones	SP (2)	MA	MA	MA	MA	MA	MA	SP (2)
*Heliconius erato*	16	3.45	Complex	SP (2)	MA	SP (2)	SP (2)	SP (2)	SP (2)	SP (2)	SP (2)
*Heliopetes omrina*	20	1.80	Complex	SP (2)	MA	MA	MA	SP (2)	SP (2)	SP (2)	SP (2)
*Heraclides androgeus*	6	1.08	Split within Misiones	MA	MA	MA	MA	MA	SP (2)	SP (2)	MA
*Lycas argentea*	4	3.28	Split within Misiones	SP (2)	SP (2)	SP (2)	SP (2)	SP (2)	SP (2)	SP (2)	SP (2)
*Mesosemia odice*	2	1.86	Split within Misiones	MA	MA	SP (2)	MA	MA	MA	MA	MA
*Ministrymon azia*	4	1.85	Misiones vs Formosa	MA	MA	MA	MA	MA	MA	MA	SP (2)
*Morys geisa*	3	2.81	Split within Misiones	SP (2)	SP (2)	SP (2)	SP (2)	SP (2)	SP (2)	SP (2)	SP (2)
*Notheme erota*	6	0.92	Split within Misiones	MA	MA	MA	MA	MA	MA	SP (2)	MA
*Ortilia ithra*	20	1.71	Complex	MA	MA	MA	MA	MA	MA	SP (3)	MA
*Paulogramma pygas*	8	1.25	Split within Misiones	SP (2)	MA	MA	MA	MA	SP (2)	SP (2)	SP (2)
*Pharneuptychia phares*	7	1.08	Complex	MA	MA	MA	MA	MA	MA	SP (2)	MA
*Phoebis argante*	10	2.04	Split within Misiones	SP (2)	ME	MA	MA	MA	MA	MA	SP (2)
*Polites vibex*	12	1.39	Complex	MA	MA	MA	MA	MA	SP (2)	SP (2)	MA
*Pyrisitia leuce*	13	3.05	Complex	MA	MA	SP (2)	SP (2)	SP (2)	SP (2)	SP (2)	MA
*Pyrrhogyra neaerea*	8	2.17	Complex	MA	MA	MA	MA	MA	MA	SP (2)	SP (2)
*Riodina lysippoides*	8	1.23	Complex	MA	MA	MA	MA	MA	MA	MA	SP (2)
*Strymon eurytulus*	9	0.98	Complex	MA	MA	MA	MA	MA	MA	SP (2)	MA
*Temenis laothoe*	8	1.71	Misiones vs Formosa	MA	MA	MA	MA	MA	MA	MA	SP (2)
*Trina geometrina*	12	2.65	Complex	SP (2)	MA	SP (2)	MA	SP (2)	SP (2)	SP (2)	SP (2)
*Vanessa braziliensis*	9	2.80	Complex	SP (2)	MA	SP (2)	SP (2)	SP (2)	SP (2)	SP (2)	SP (2)
*Zaretis strigosus*	4	5.71	Misiones vs Formosa	SP (2)	SP (2)	SP (2)	SP (2)	SP (2)	SP (2)	SP (2)	SP (2)

For each species, the number of sequences (N), the maximum intraspecific distance (K2P, %), and the congruence between MOTU and species boundaries for each clustering method (MA: MATCH, SP: SPLIT, ME: MERGE, MI: MIXTURE) are shown. The figures in parenthesis indicate the number of MOTUs into which each species was split. The geographic location of the split is also indicated for each species.

*also involved in cases of merging.

**Table 4:** Species merged with another taxon into a single MOTU by one or more of the clustering algorithms.

Species	N	Max. distance	Min. distance to NN	RESL	TCS 90%	TCS 95%	ABGD initial	ABGD recursive P = 1.29	ABGD recursive P = 0.77	ABGD recursive P = 0.46	BIN
*Actinote melanisans*	2	0.30	1.07	ME	ME	ME	ME	ME	ME	ME	ME
*Actinote pellenea*	27	0.92	1.07	ME	ME	ME	ME	ME	ME	ME	ME
*Aricoris indistincta*	2	0.00	2.33	MA	ME	MA	MA	MA	MA	MA	MA
*Aricoris signata*	6	0.15	2.33	MA	ME	MA	MA	MA	MA	MA	MA
Calycopis caulonia*	7	2.95	0.00	MI (2)	ME	MI (2)	MI (2)	MI (2)	MI (2)	MI (2)	MI (2)
*Calycopis sp*. 1	1	NA	2.00	MA	ME	MA	ME	ME	ME	ME	MA
*Calycopis sp*. 2*	5	5.72	0.00	MI (3)	MI (3)	MI (3)	MI (3)	MI (3)	MI (3)	MI (3)	MI (3)
*Cymaenes gisca*	11	0.15	2.17	MA	ME	MA	ME	ME	MA	MA	MA
*Cymaenes laureolus*	5	0.15	1.23	MA	ME	ME	ME	ME	MA	MA	MA
*Cymaenes lepta*	5	0.15	1.23	MA	ME	ME	ME	ME	MA	MA	MA
*Emesis mandana*	3	0.61	0.92	ME	ME	ME	ME	ME	ME	ME	ME
*Emesis russula*	6	1.70	0.92	ME	ME	ME	ME	ME	ME	ME	ME
*Epargyreus exadeus*	3	0.77	1.07	ME	ME	ME	ME	ME	ME	ME	ME
Epargyreus socus*	5	7.44	1.07	MI (3)	MI (3)	MI (3)	MI (3)	MI (3)	MI (3)	MI (3)	MI (3)
*Epargyreus tmolis*	5	0.30	1.23	ME	ME	ME	ME	ME	ME	ME	ME
*Eunica eburnea*	2	0.15	1.40	MA	ME	ME	ME	ME	ME	ME	MA
*Eunica margarita*	3	0.30	1.40	MA	ME	ME	ME	ME	ME	ME	MA
*Ministrymon cruenta*	2	0.16	1.23	ME	ME	ME	ME	ME	ME	ME	MA
*Ministrymon gamma*	1	NA	1.23	ME	ME	ME	ME	ME	ME	ME	MA
*Phoebis argante*	10	2.04	2.49	SP (2)	ME	MA	MA	MA	MA	MA	SP (2)
*Phoebis neocypris*	15	0.81	2.49	MA	ME	MA	MA	MA	MA	MA	MA
*Strymon lucena*	1	NA	2.56	MA	ME	MA	MA	MA	MA	MA	MA
*Strymon megarus*	2	0.15	2.56	MA	ME	MA	MA	MA	MA	MA	MA

For each species, the number of sequences (N), the maximum instraspecific distance (K2P, %), the minimum distance to the nearest non-conspecific (i.e. nearest neighbor, NN), and the congruence between MOTU and species boundaries for each clustering method are shown (acronyms and other details as in Table 3).

*also involved in cases of split.

TCS produced between 416 and 479 MOTUs (Tables C and D in S1 Supporting Information) depending on the cut-off value (i.e. parsimony limit). Because the 95% connection limit has been shown to produce good results across a broad range of taxa [65], we initially concentrated on this cut-off value. It generated 429 MOTUs with 92.81% of MATCHES and the same percentage of MIXTURES as RESL (Figs 4 and 5). Fifteen species (3.60%) were split into two MOTUs, 12 shared with RESL and only one exclusive to this partition (Table 3). MERGES (2.88%) increased with TCS in comparison to RESL, with six pairs of species being combined into a single genetic cluster (Table 4). Because the 90% partitioning scheme produced a MOTU count (416) that was closest to the number of reference species (Fig 4), this partition was included in the general comparison too. Interestingly, this cut-off value generated a percentage of MATCHES (92.57%) that was very similar to that obtained with the 95% limit, in spite of a higher proportion of MERGES (5.04%, Table 4, Fig 5). The incidence of SPLITS (1.92%, Table 3, Fig 5) was low, and only *Calycopis sp*. 2 and *E*. *socus* were involved in both merges and splits.

ABGD produced between 424 and 997 MOTUs depending on the settings (Tables C and E in S1 Supporting Information). The two X values produced almost identical results across all P values, with a slightly higher number of MOTUs found in the recursive partitions when X = 1.0. Regarding the distance model, K2P and uncorrected distances also behaved similarly across prior divergence values (Table E in S1 Supporting Information). Because of this, and in order to only capture large gaps [32], we focus on the partitions obtained with X = 1.5 and K2P divergences. We also discarded the partitioning schemes obtained from the lowest P values (0.1% to 0.28%) due to the extremely high MOTU counts generated (Table E in S1 Supporting Information).

ABGD’s initial partition produced 424 MOTUs (P values from 0.46% to 2.15%) and 93.05% of MATCHES (Figs 4 and 5). Twelve species (2.88%) were divided into two MOTUs (Table 3), and the percentage of MERGES (3.36%; Table 4) was the second highest after TCS 90% (Fig 5). The same three species mentioned above fell in the MIXTURE category. Excluding the one that matched the initial partition, recursive partitions delivered between 444 (P = 0.46%) and 430 (P = 1.29%) MOTUs (Fig 4, Table E in S1 Supporting Information). The percentage of MATCHES was similar across partitions, ranging from 90.17% (P = 0.46%) to 92.09% (P = 1.29%). The number of species that were split varied from 16 to 27 (Table 3), and MERGES (Table 4) ranged from 2.64% to 3.36% (Fig 5). Lastly, *Calycopis sp*. 2, *C*. *caulonia* and *E*. *socus* were again both split and merged.

Bootstrap support values for the species that were split into two or more MOTUs (Table 3) were generally high: 92% and 76% of these intraspecific clusters had support values ≥ 90% in the NJ and ML gene trees respectively (S1 and S2 Figs). On the other hand, all species pairs or triads that were merged into a single MOTU (Table 4) consisted of closely related species with minimum distance to the nearest non-conspecific always below the lower 5% of all congeneric distances (3.46%).

### Geographic patterns of intraspecific variation

In total, 46 species showed maximum intraspecific divergence values that were equal or higher than 1.39% (the 95^th^ percentile of intraspecific variation) and/or were split into two or more MOTUs by one or more of the clustering algorithms (Tables 3 and 5). Examination of these species revealed a clear geographic pattern: 40% (18 species; Tables 3 and 5) showed deep divergences between specimens collected within the Atlantic Forest in Misiones. Eight other species (17%) showed deep sequence variation among Argentinian regions. In particular, six species (Tables 3 and 5) evidenced a phylogeographic break between the Atlantic Forest in Misiones and the Humid Chaco eco-region in Formosa, while one species (*Gorgytion begga*) showed a split between Misiones and Entre Ríos (Espinal eco-region), and another (*Stegosatyrus periphas*) between Córdoba (central Argentina) and northeastern Argentina. The other 20 species (43%; Tables 3 and 5) showed no geographic pattern or a more complex scenario with divergence in sympatry and among regions. Finally, we found little evidence of isolation-by-distance: only 11 of the 42 species analyzed showed a statistically significant (*p* < 0.05) positive relationship between geographic and genetic distances, while the remaining 74% (31 species) did not (Table F in S1 Supporting Information).

**Table 5:** Species with high intraspecific variation that were not split by the clustering algorithms.

Species	N	Max. distance	Geographic pattern	RESL	TCS 90%	TCS 95%	ABGD initial	ABGD recursive P = 1.29	ABGD recursive P = 0.77	ABGD recursive P = 0.46	BIN
Emesis russula*	6	1.70	Misiones vs Formosa	ME	ME	ME	ME	ME	ME	ME	ME
*Doxocopa laurentia*	8	1.39	Complex	MA	MA	MA	MA	MA	MA	MA	MA
*Dryas iulia*	12	1.39	Complex	MA	MA	MA	MA	MA	MA	MA	MA
*Dynamine artemisia*	8	1.54	Split within Misiones	MA	MA	MA	MA	MA	MA	MA	MA
*Mechanitis lysimnia*	10	1.54	Split within Misiones	MA	MA	MA	MA	MA	MA	MA	MA
*Parides neophilus*	4	1.54	Misiones vs Formosa	MA	MA	MA	MA	MA	MA	MA	MA
*Perichares lotus*	2	1.39	Split within Misiones	MA	MA	MA	MA	MA	MA	MA	MA
*Pyrgus orcus*	22	2.02	Complex	MA	MA	MA	MA	MA	MA	MA	MA
*Stegosatyrus periphas*	11	1.86	Córdoba vs NEA	MA	MA	MA	MA	MA	MA	MA	MA

Details as in Table 3. NEA: Northeastern Argentina.

*merged with *E*. *russula* by the clustering algorithms (see Table 4)

## Discussion

This study has assembled a DNA barcode reference library for 417 butterfly species of Argentina, nearly 35% of the fauna [14]. It also provided the first documented occurrences for *Eurema agave*, *Mithras hannelore*, *Melanis hillapana* (more details in the S2 Appendix), and more new records for the country are likely to be included among the nine taxa that could only be identified to a generic level (*Calycopis sp*. 1 and 2, *Cobalopsis sp*. 1, *Corticea sp*., *Eutocus sp*. 1, *Kolana sp*. 1, *Paryphthimoides sp*. 1, *Virga sp*., *Zaretis sp*.; Table A in S1 Supporting Information).

Genetic divergence within species was much lower than among them as the mean distance to the nearest-neighbor (i.e. minimum interspecific divergence) was ten times larger than the mean maximum intraspecific distance. Consequently, the barcode gap was present in all but four of the species represented by two or more specimens (see below). These levels of intra- and interspecific variation are similar to those reported in earlier barcoding studies on Lepidoptera [25–27,29,66,67]. Intraspecific variation could not be assessed for 27% of the species in our database which were represented by a single individual. Nevertheless, all these singletons possessed unique DNA barcodes that made them clearly distinguishable from their closest non-conspecific in the gene trees (“Tree Based Identification” approach [58]).

In concordance to the above explained, the sequence-based identification simulations showed that our barcode library was very effective in the identification and discrimination of southern Neotropical butterflies. Identification success always exceeded 95% for all species represented by two or more individuals regardless of the identification criterion or sequence threshold implemented (Table 2). The BM criterion generated the highest percentage of correct identifications, illustrating the rarity of problematic cases within our data, since it makes a species assignment regardless of sequence divergence. However, the BM has a weakness: all newly encountered species (i.e. species without conspecific sequences in the database) will be incorrectly assigned to other species. The number of queries which failed to receive a species assignment was similar among the varied approaches, but they tended to increase when BIC and higher thresholds were implemented. This pattern reflects the fact that high thresholds (1.39%, 2.06%) lie near or within the narrow zone of overlap between intra- and interspecific divergences (Fig 2). However, high thresholds do have an advantage: they help to identify species with deep intraspecific divergence (Table 2), explaining why the “localMinima” threshold worked extremely well with the BCM. However, if species with deep divergence are actually two or more cryptic species, high thresholds will increase the number of ambiguous identifications when using strict approaches like BIC. In this context, we believe that the use of BIC (because of its more rigorous nature) with a lower threshold, like the 1% implemented by BOLD’s ID engine, or 0.85% as suggested by the “threshVal” function, is currently the best approach for the butterflies of Argentina as it produced 98% correct identifications, no incorrect assignments, and a low percentage of ambiguous calls.

All clustering algorithms, excepting TCS 90%, delivered MOTU counts that were greater than the number of reference species recognized based on current taxonomy, suggesting the existence of cryptic diversity. If the intraspecific splits detected in this study (see below) actually represent new species, the count for the butterflies of Argentina could be 3%–9% higher than currently recognized. There was an overall good correspondence between species and MOTUs boundaries across all methods, with MATCHES always exceeding 90%. RESL was the clustering algorithm that delivered the highest correspondence between MOTUs and reference species, followed closely by ABGD’s initial partition, as previously reported for the Lepidoptera of North America [31]. This earlier study found a stronger correspondence between species and MOTU counts, but the congruence between MOTUs and species boundaries was lower than for the butterflies of Argentina. As for ABGD, the high correspondence in the initial partition is not surprising since Puillandre et al. [32] showed that primary partitions are usually close to the number of described species. Congruence among partitioning schemes was also high, with 92% of the clusters (382 MOTUs) being recognized by all methods.

Ratnasingham & Hebert [31] described RESL as a more conservative approach in comparison to other clustering methods because of a higher tendency to merge taxa with low divergence rather than splitting them. This does not seem to be the case with the butterflies of Argentina, since we found that a) SPLITS were higher than MERGES with this method, b) the incidence of SPLITS with RESL was similar or even higher than that with other algorithms, and c) the percentage of MERGES was the lowest with RESL (Fig 5). This pattern is clearer under the BIN system due to an even higher and lower incidence of SPLITS and MERGES respectively (Fig 5). The latter shows that the outcome of the RESL can be influenced by the addition of other sequences uploaded to the platform, supporting our decision of excluding the BINs from the general comparison as they are not based on the same data used with the other algorithms (see Materials and methods). That being said, the combined analysis of our sequences and those available on BOLD through the BIN system allowed us to identify some intraspecific splits that were not detected by any of the other methods (including RESL applied to our data only; Table 3, but see below).

We found a clear pattern of sympatric divergence within the Atlantic Forest in Misiones province: 18 species (Tables 3 and 5) possessed two or more divergent lineages within the same sampling locality or between localities that are less than 30 km apart (Tables A and B in S1 Supporting Information). Based on their COI divergences and standard molecular rates [68], most of these splits occurred during the last two million years, a period of climatic fluctuations that appear to have fragmented the range of the Atlantic Forest, creating opportunities for isolation and speciation [69,70]. While there is an ongoing debate around the evolutionary impacts of the habitat fragmentations caused by Pleistocene glaciations in the Brazilian Atlantic Forest, much less is known about the past status of this forest in Argentina. However, it was recently suggested that the Atlantic Forest in Misiones was a refuge during the Last Glacial Maximum for the fire ant *Wasmannia auropunctata* [71]. As well, two new forest refugia in Paraná and Santa Catarina, two Brazilian states bordering Misiones, have been recently proposed [69]. Viewed from this perspective, the divergent barcode lineages found in Misiones may reflect secondary contact between populations that diverged in allopatry in forest refugia during the Pleistocene. Alternatively, these lineages could reflect sympatric, ecology-driven divergence [52]. Distinguishing between these two hypotheses is not possible without further studies.

Six species showed a phylogeographic break between their populations from the Atlantic Forest and those from the Humid Chaco eco-region in Formosa (Tables 3 and 5), while *G*. *begga* showed a split between the Atlantic Forest and the Espinal eco-region in Entre Ríos. One species (*Zaretis strigosus*) showed relatively deeper divergence dating to ca. 2.5 Ma, while the remaining splits were dated to between 300,000 and 1 million years. At first sight these cases of divergence within northeastern Argentina could be interpreted as isolation by distance, but our results revealed a weak relationship between genetic and geographic distances. As mentioned above, Pleistocene climatic fluctuations could have fragmented the geographic range of these species, enabling local adaptation of populations to different ecological niches. Because the Atlantic Forest, the Espinal and the Humid Chaco are markedly different, ecological divergence is a strong hypothesis. Alternatively, these six cases could reflect isolation created by the Del Plata Basin, and more precisely the Paraná-Paraguay fluvial Axis (Fig 1) which could restrict gene flow in species with limited dispersal ability [72,73]. Because the configuration of this Basin has shifted several times since its establishment in the Neogene, the variance on divergence dates among these species could reflect isolation events driven by current and past palaeochannels of the rivers [74,75]. More geographically comprehensive sampling, especially in Corrientes province, is needed to better understand the impact of these riverine barriers on population structure.

**Fig 6:**
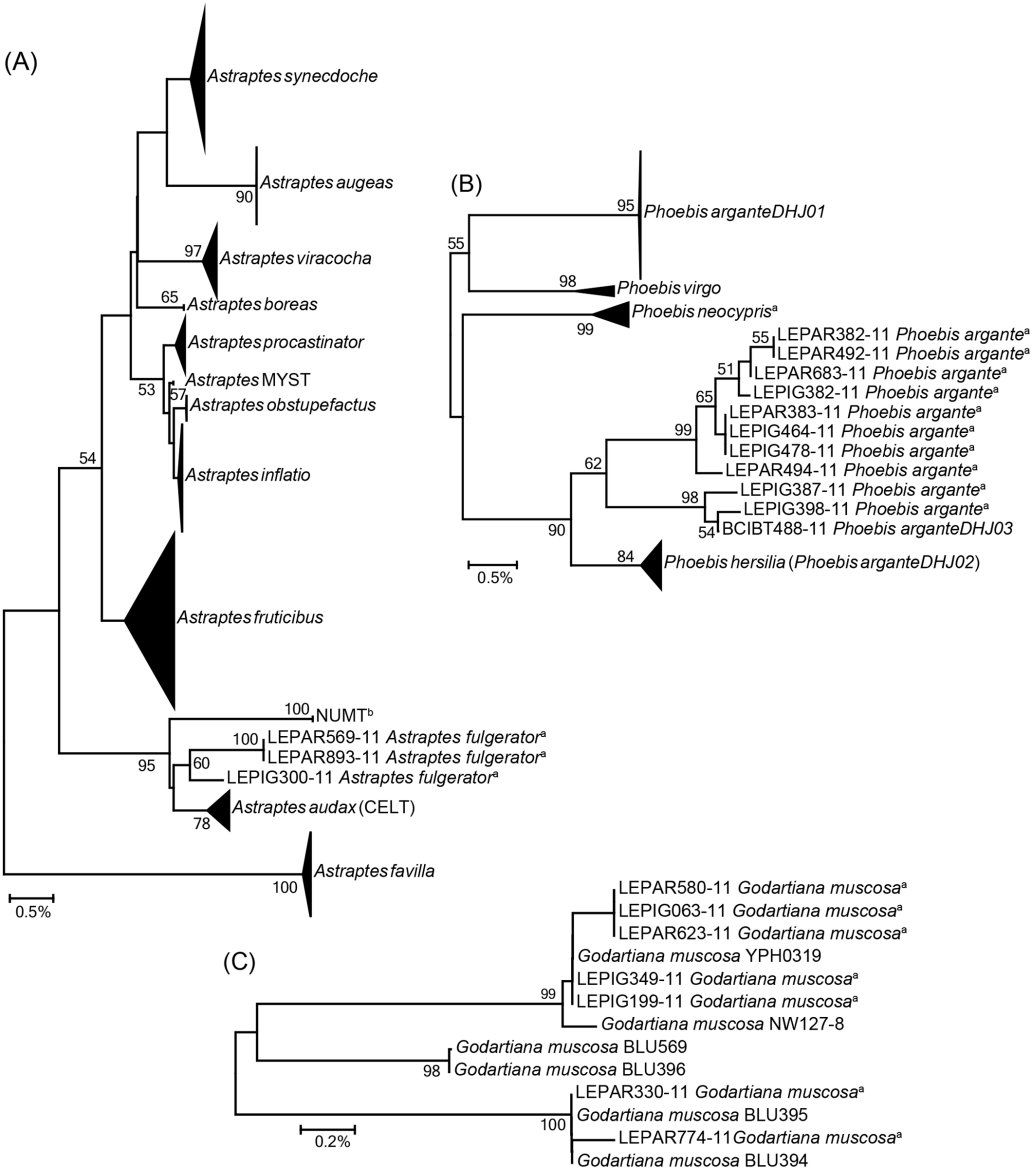
NJ trees for three of the species with divergence within the Atlantic Forest that have received previous investigation. (A) *Astraptes fulgerator* complex [52,77], (B) *Phoebis argante* [78], and (C) *Godartiana muscosa* [79]. Numbers above or below branches indicate bootstrap support based on 1,000 pseudoreplicates (only values ≥ 50% are shown). ^a^Sequences generated in this study. ^b^Brower [76] suggested that sequences within the clade “NUMT” are not pseudogenes.

Three of the species with divergence within the Atlantic Forest have received previous investigation. The best studied case involves *Astraptes fulgerator*, a taxon that has been shown to represent a complex of at least ten species with no genitalic divergence and only subtly differing adults [52,76]. Our study indicated the occurrence of two divergent lineages of *A*. *fulgerator* in Misiones which represent two distinct BINs on BOLD, and are closely related (Fig 6A) to the MOTU “CELT” described by Hebert et al [52], currently known as *A*. *audax* [77]. The mean divergence (1.23%) found between our two *A*. *fulgerator* lineages (S3 Appendix) and among these and *A*. *audax* (from 0.92% to 1.38%, Fig 6A) is similar to the genetic distances among some of the other species in the complex [52]. In this context, our results suggest the existence of at least one, and perhaps two, new species in the *A*. *fulgerator* complex within Argentina. Further studies to examine morphology of the caterpillars and food plant use are needed to clarify the status of these lineages.

Janzen et al. [78] reported the occurrence of two barcode clusters for *P*. *argante* in northwestern Costa Rica, which were assigned interim names *P*. *arganteDHJ01* and *P*. *arganteDHJ02* (the latter could correspond to *P*. *hersilia*). They suggested that a more geographically comprehensive sampling might uncover additional lineages of this species, and our study revealed two new lineages in Misiones that seem close in external morphology and barcode sequence to *P*. *arganteDHJ02* (Fig 6B). Moreover, one of these two lineages matched a third taxon from Costa Rica (*P*. *arganteDHJ03*, Fig 6B) which has not yet been described in the literature. We did not observe differences in wing colours or pattern in the two lineages found in Argentina (S3 Appendix), in contrast to differences apparent between DHJ01 and DHJ02 [78]. However, differences between Costa Rican taxa are clearer in females and all of our specimens were males. Further studies examining specimens of both sexes coupled with detailed assessment of variation in genitalia and wing colour patterns are needed to establish the status of these lineages [45]. Lastly, a recent revision of the genus *Godartiana* [79] evidenced the existence of three lineages within *G*. *muscosa* in southeastern Brazil with COI divergences of 2%–2.5%. However, the authors did not highlight this genetic variation within the species and, even though they examined over 100 males and 80 females, they did not report any differentiation in external or internal morphology either. We found two of these three lineages in the Atlantic Forest in Misiones (Fig 6C, S3 Appendix), confirming the results of Zacca et al. [79] and emphasizing the need for a deeper phylogeographic analysis of *G*. *muscosa*.

In total, 23 species were merged in at least one of the partitioning schemes discussed (Table 4). However, the barcode gap was only absent in the *Calycopis sp*. 2 and *C*. *caulonia* species pair, in *E*. *socus* (split and merged with other species of the genus *Epargyreus*), and in *E*. *russula* (merged with *E*. *mandana*). These species were paraphyletic in the COI gene trees and although it is tempting to suggest that they represent cases of incomplete lineage sorting or mitochondrial introgression, we cannot dismiss the possibility of erroneous identifications [80]. These can arise as a consequence of taxonomic inaccuracy (i.e. mistaking one species for another) and/or taxonomic limitations due to the existence of overlooked diversity [80]. The latter seems to be the case for the species of *Calycopis* analyzed here. The species-level taxonomy of this genus is largely unknown because of sexual dimorphism and high, poorly documented intraspecific variation [81,82]. Although we found *Calycopis sp*. 2 (S3 Appendix) to resemble *C*. *talama* [48] in external morphology, the latter is thought to be endemic to the Sierra do Mar in Brazil (R. Robbins, personal communication). Therefore, it is possible that *Calycopis sp*. 2 represents a species (or more than one; see next) that has not yet been described for Argentina. Beyond this, the specimens here identified as *Calycopis sp*. 2 were split into three distinct barcode clusters (with no clear external morphological differentiation; S3 Appendix) and some of them even shared their COI sequence with specimens of *C*. *caulonia*. This last species was also split into two MOTUs, being one of them often merged with *Calycopis sp*. 1, another taxon that probably represents an undescribed species for the country (S3 Appendix). Overall, these cases confirm that the species of *Calycopis* are difficult to sort using either external morphology or mitochondrial DNA [83], and emphasize the need for a revision of the genus.

The case of *E*. *russula* appears as complex as it is interesting. This species was always merged with its congeneric *E*. *mandana* (Table 4), and at the same time showed a deep split between Misiones and Formosa (Table 5). In fact, the individuals from Misiones appeared more closely related to that of *E*. *mandana* (from the same province) than to their conspecifics collected in Formosa, making *E*. *russula* paraphyletic as currently defined (S1 and S2 Figs). A possibility is that one of the two COI lineages found within *E*. *russula* actually represents a cryptic species (no differentiation was found in external morphology; S3 Appendix). At the same time, regardless of the taxonomic status of the intraspecific lineages found within *E*. *russula*, the divergence between the individuals of this species collected in Misiones and those of the congeneric *E*. *mandana* is only shallow. In fact, the distance between these two species was the lowest among non-conspecifics within our database (leaving aside the cases of barcode sharing). A more detailed study with a deeper sampling and a better evaluation of external and internal morphological differentiation is required to fully understand this case. Unfortunately, no literature was available for *Emesis* and *Epargyreus* to help further explain the case of *E*. *russula* and its relationship with *E*. *mandana*, and that of *E*. *socus*. As for *Calycopis*, a revision of these two genera is badly needed.

Future studies should revisit the cases highlighted here to examine the possible role of other factors that can confound the interpretation of mitochondrial divergence patterns. For example, maternally transmitted *Wolbachia* can affect the reproductive system of their hosts, leading to complex patterns of intraspecific COI variation and cases of barcode sharing [84]. Although *Wolbachia* sequences were rarely recovered with the amplification protocols employed in this study, the majority of infections will remain unnoticed until a more rigorous screening protocol using the *wsp* (*Wolbachia* surface protein) gene is performed [84]. Another potential confounding factor is the co-amplification of pseudogenes, but we found no in-frame stop codons or indels causing a frame shift, suggesting that the amplification of pseudogenes was unlikely [85]. However, because pseudogenes can also be cryptic their occurrence cannot be entirely dismissed without a more careful examination of sequence characteristics [85].

Zenker et al. [33] recently performed the first DNA barcoding study of Lepidoptera in southern South America with a fast census of moth diversity in Brazil. Our project extends this work by performing both the first extensive genetic assessment of the butterflies of this region and the pioneer large-scale DNA barcoding survey of the Lepidoptera of Argentina. Our work added 157 new barcode clusters (BINs) to the global COI library, increasing its geographical and taxonomic coverage and therefore contributing towards a better representation of butterfly diversity worldwide. At the same time, this study not only demonstrated the efficacy of DNA barcodes for the discrimination of the butterfly species of Argentina, but also uncovered several cases of cryptic diversity and revealed interesting geographic patterns of genetic variation. Future studies should benefit from these results by performing more detailed and geographically comprehensive evolutionary studies of the taxonomic cases unveiled here, using an integrative approach combining genetic data with the assessment of genitalic and external morphological differentiation. These studies will certainly contribute to a better understating of butterfly diversification in the poorly studied temperate regions of southern South America.

Finally, given the increasing rate of species loss and the fact that the number of undescribed insect species is far higher than that of taxonomic specialists, there is a need for a rapid, effective way of describing biological diversity before it disappears as a result of human activities [42,86,87]. In this context, biodiversity scans through DNA barcodes such as the one performed here can provide a rapid assessment of cryptic diversity and, in conjunction with traditional taxonomy, help to establish the direction of conservation actions for those areas of great importance, such as the Atlantic Forest in southern South America [18,20,42,86].

## Supporting information

S1 Fig BOLD NJ tree for all 2,020 COI sequences analyzed. Colours indicate different BINs. Numbers above or below branches indicate bootstrap support based on 1,000 pseudoreplicates.
(PDF)

S2 Fig Best ML tree for all 2,020 COI sequences analyzed. Numbers above or below branches correspond to bootstrap support based on 1,000 pseudoreplicates.
(PDF)

S1 Supporting Information Supplementary Tables A–F. Summary of collection and sequence data for the 2,161 specimens collected (including the geographic coordinates for all collection sites), the results of the TCS and ABGD analyses, the congruence between species and MOTUs boundaries, and the results of the 42 Mantel tests performed.
(PDF)

S2 Supporting Information Supplementary Figs A and B. Results from the “threshVal” and “localMinima” functions implemented in SPIDER. These functions were used to compute two of the four thresholds employed for the BCM and BIC criteria.
(PDF)

S1 Appendix Detailed explanation of the clustering algorithms implemented. Details on how ABGD, TCS and RESL partition the alignment of DNA barcodes into Molecular Operational Taxonomic Units (MOTUs).
(PDF)

S2 Appendix Three species of butterflies newly reported for Argentina. Details on three new records of butterflies for Argentina generated in the context of the field work carried out for this project.
(PDF)

S3 Appendix Photos of the specimens associated with the taxonomic cases discussed in more detail. We provide pictures of some specimens involved in the taxonomic cases discussed in more depth in the Discussion section: *A*. *fulgerator*, *P*. *argante*, *G*. *muscosa*, *Emesis russula/E*. *mandana* and *Calycopis sp*. *1*/*Calycopos sp*. 2/ *C*. *caulonia*.
(PDF)
