# Supplementary figures and images for: Barcoding the butterflies of southern South America: Species delimitation efficacy, cryptic diversity and geographic patterns of divergence
Source: PLoS One. 2017 Oct 19;12(10):e0186845. doi: 10.1371/journal.pone.0186845 (PMC5648246; doi:10.1371/journal.pone.0186845)

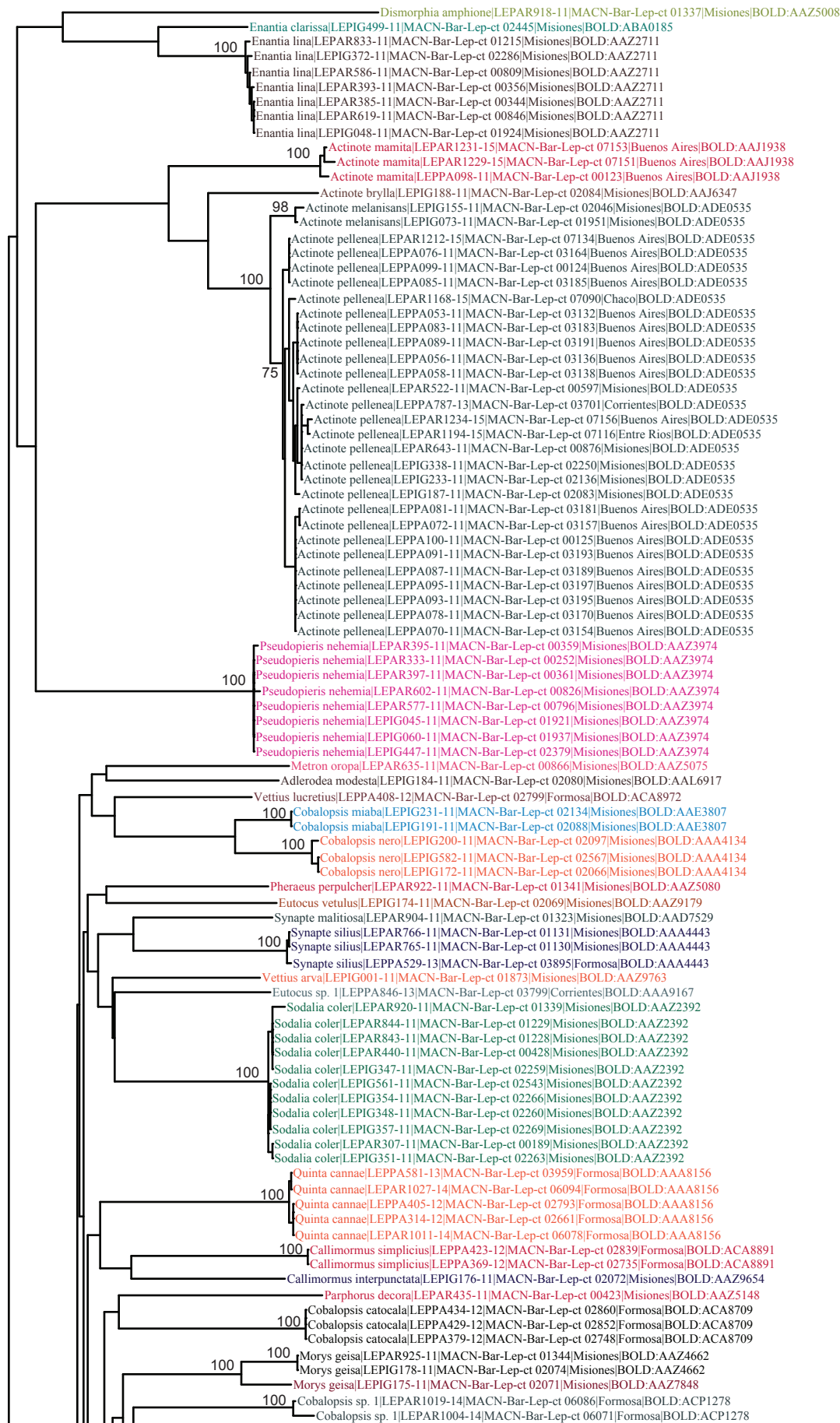

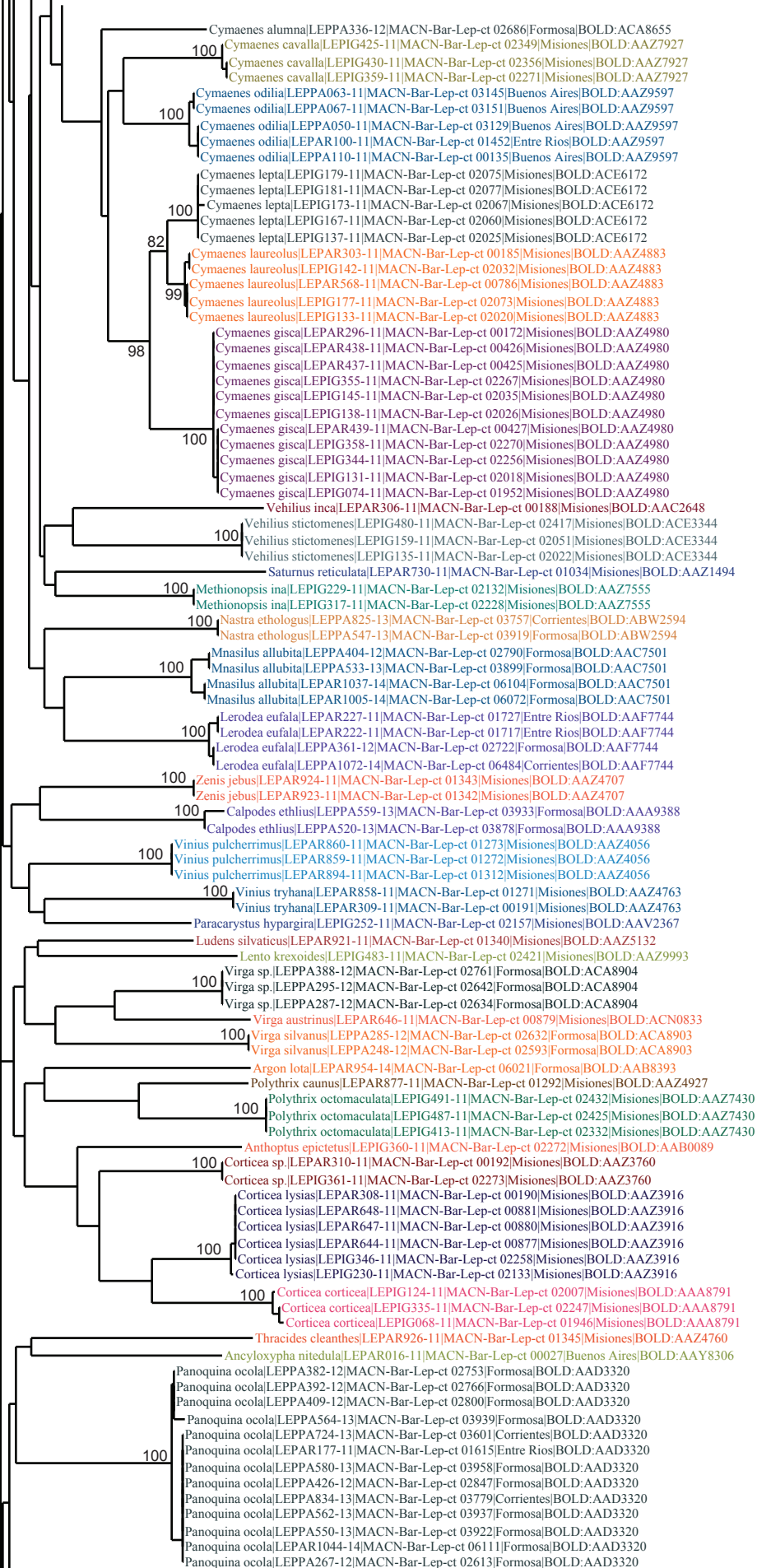

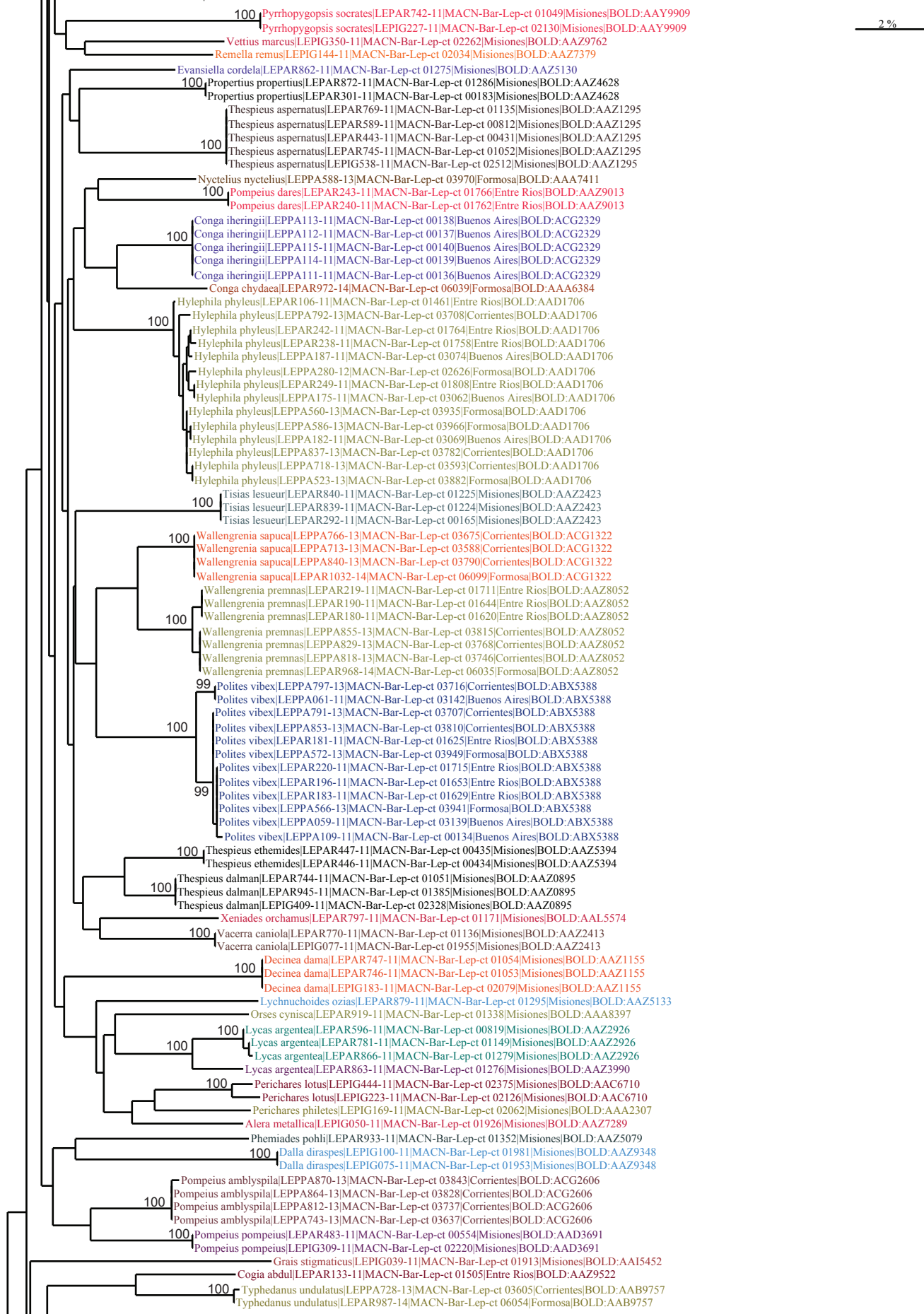

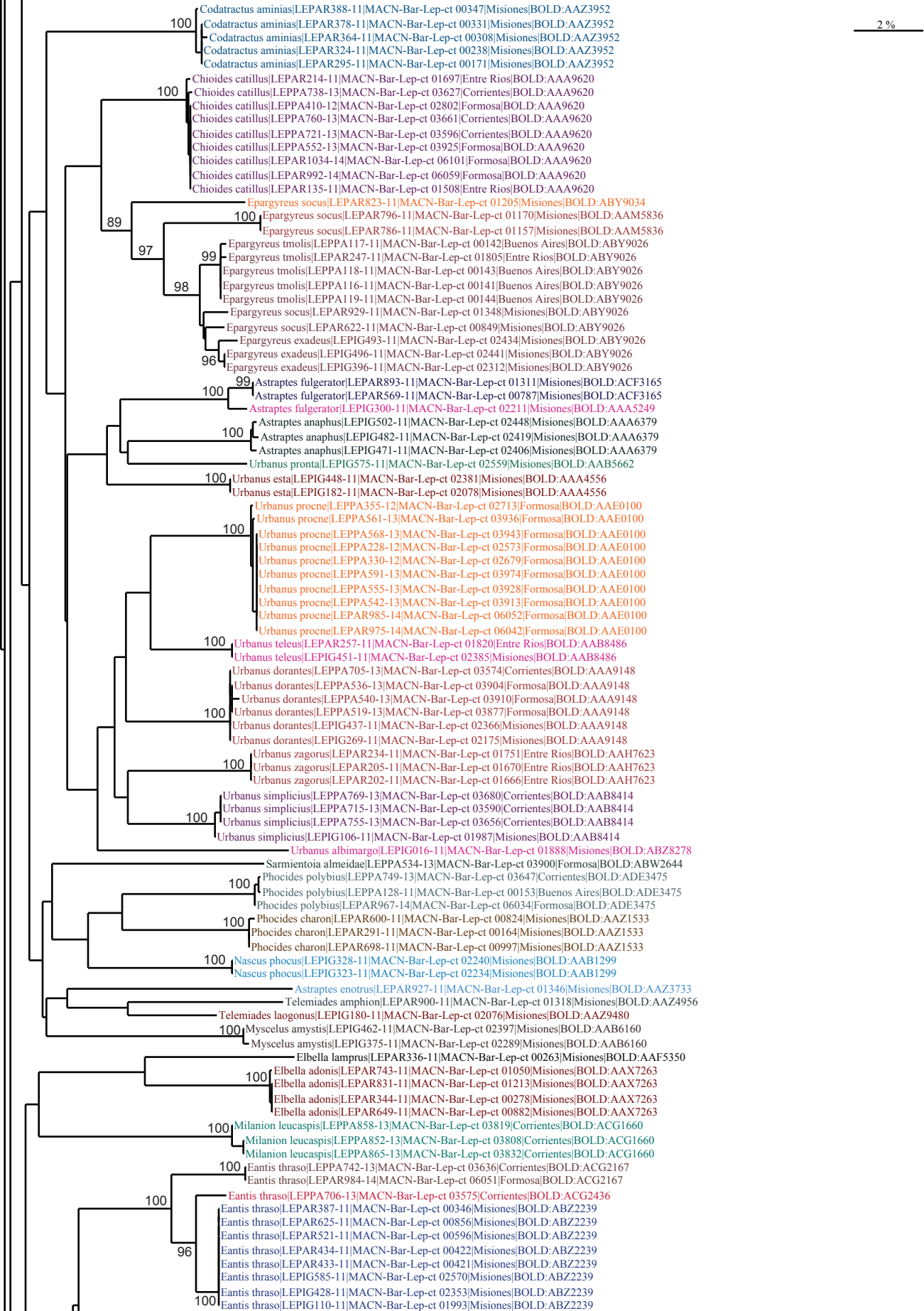

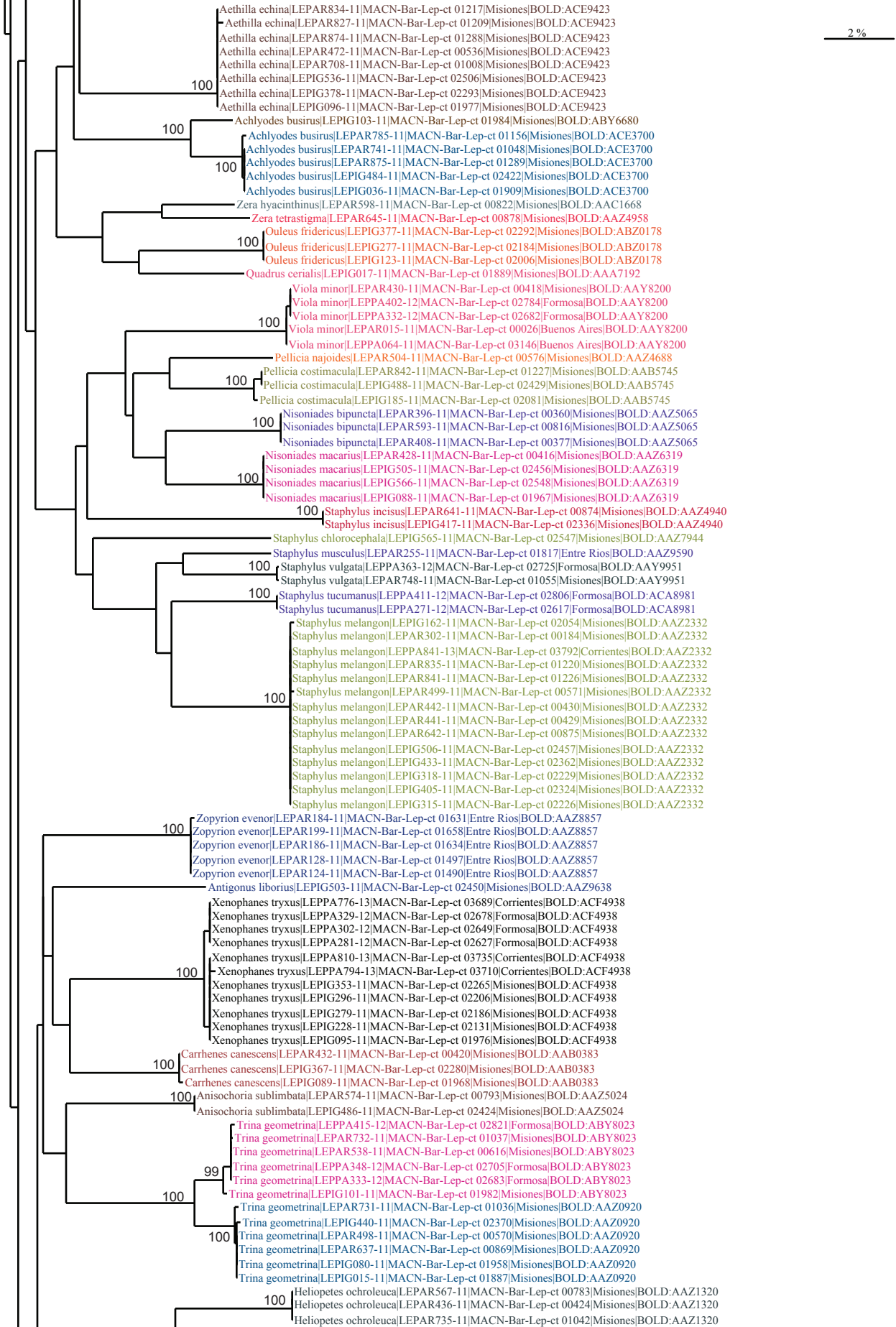

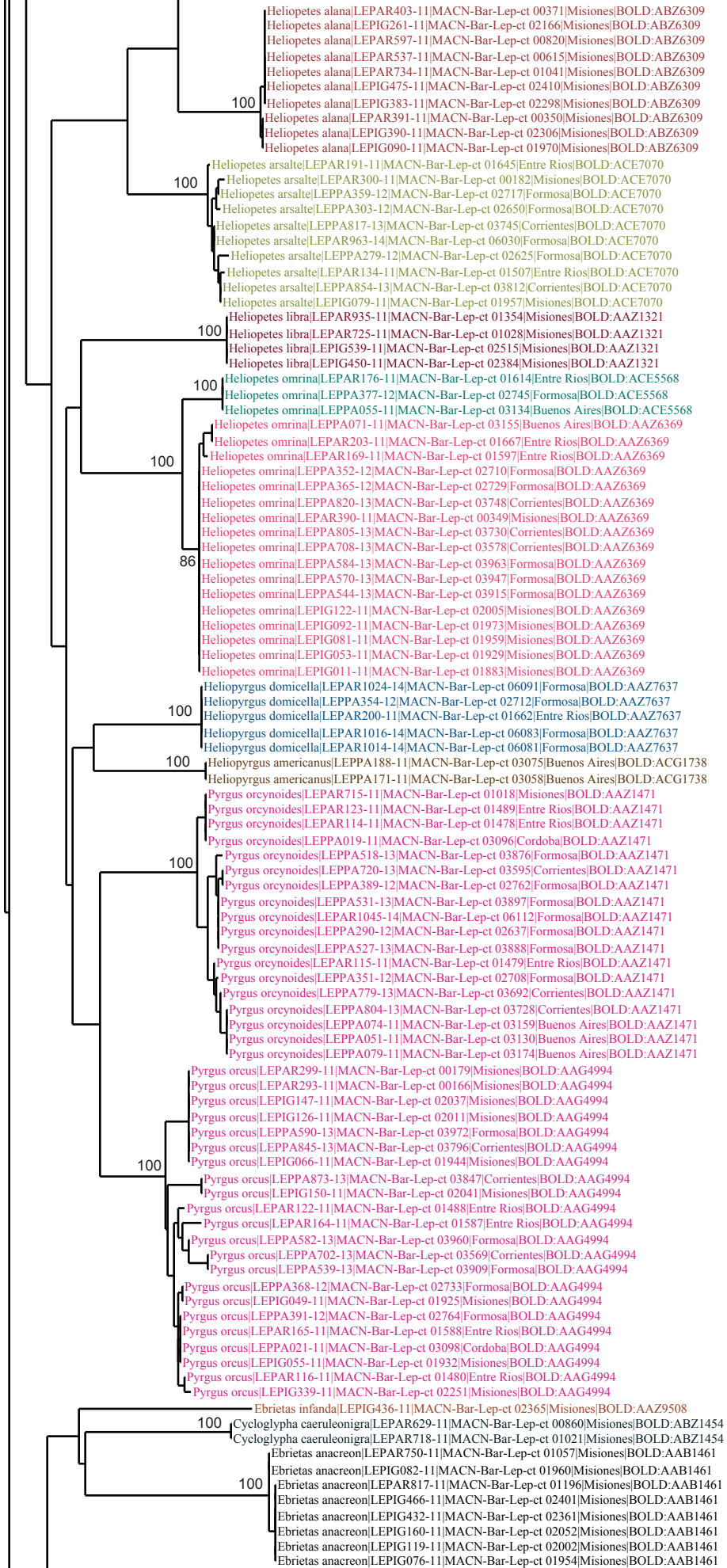

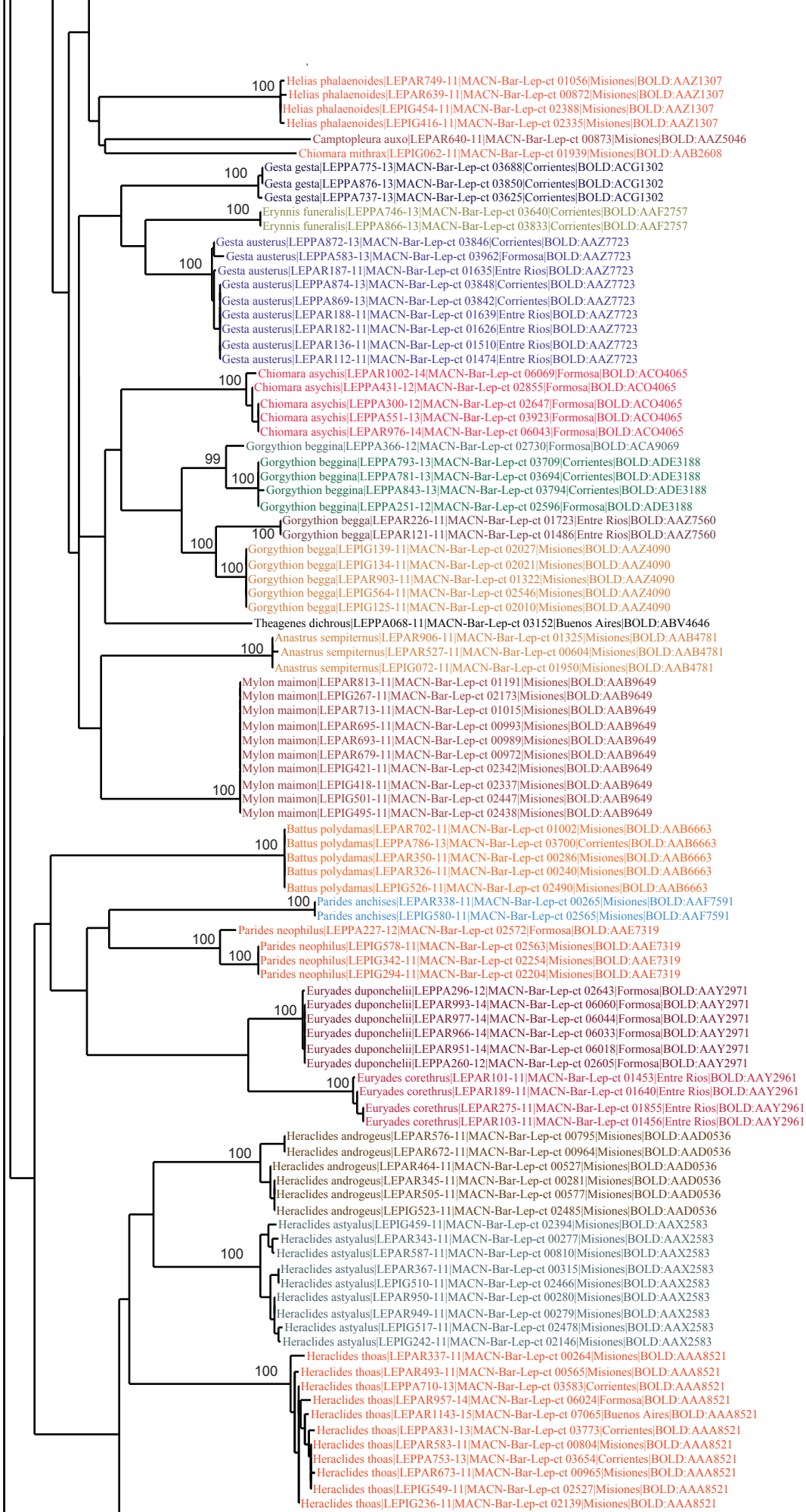

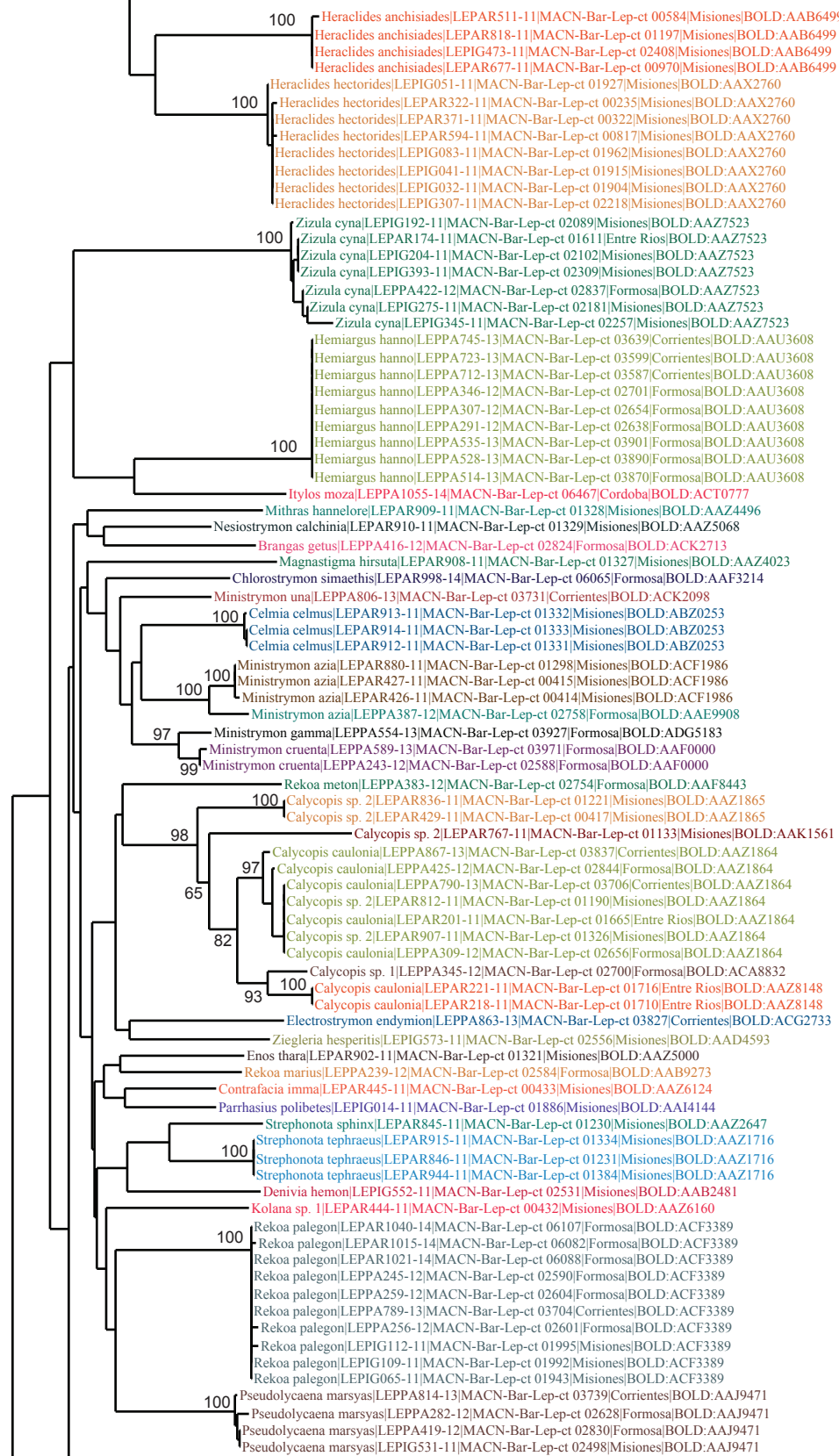

2 %

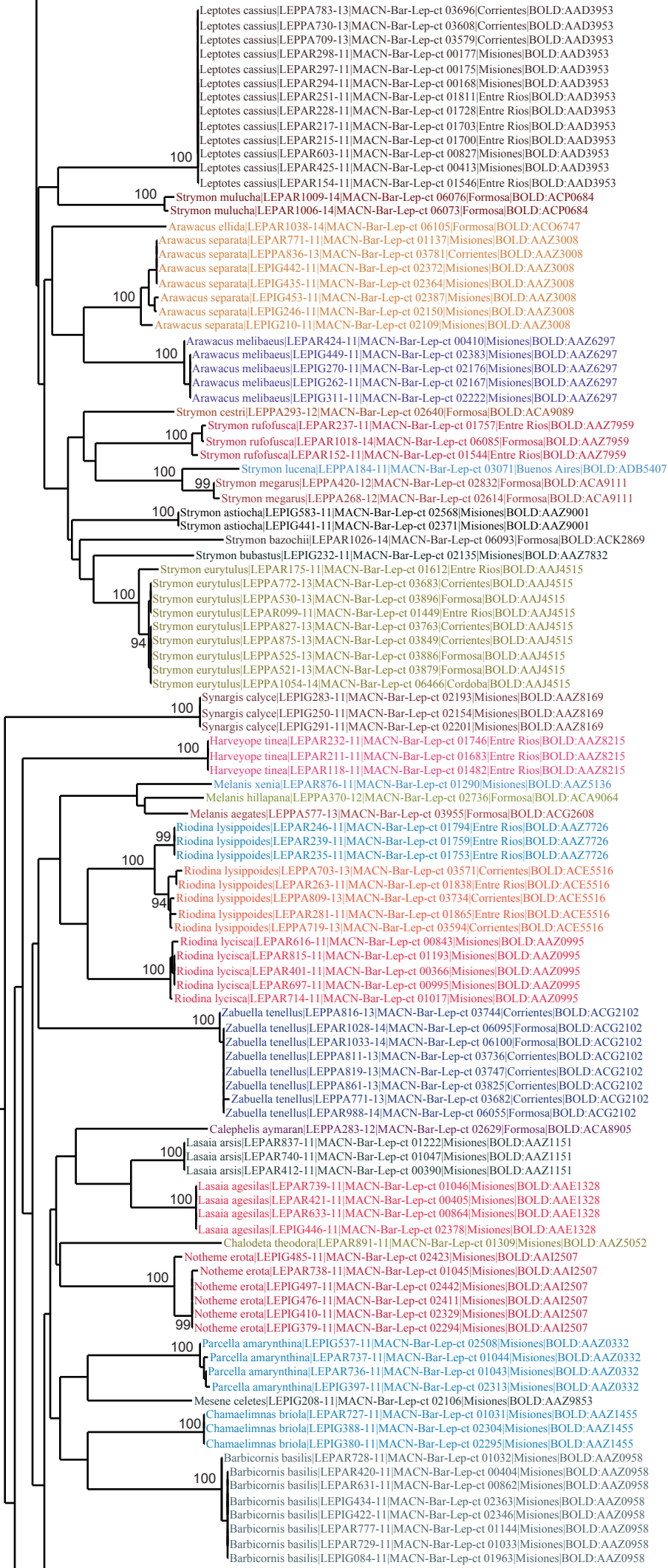

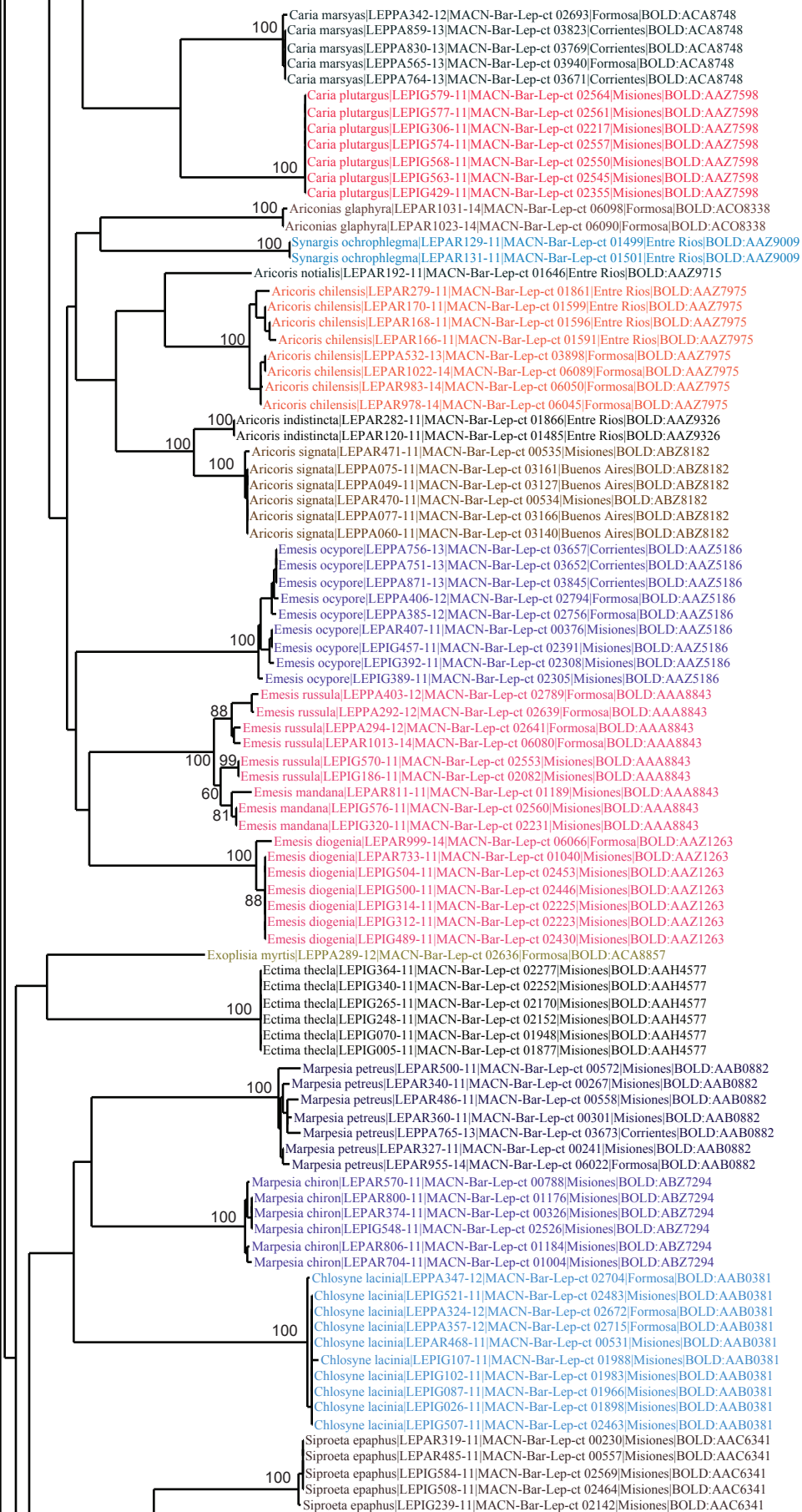

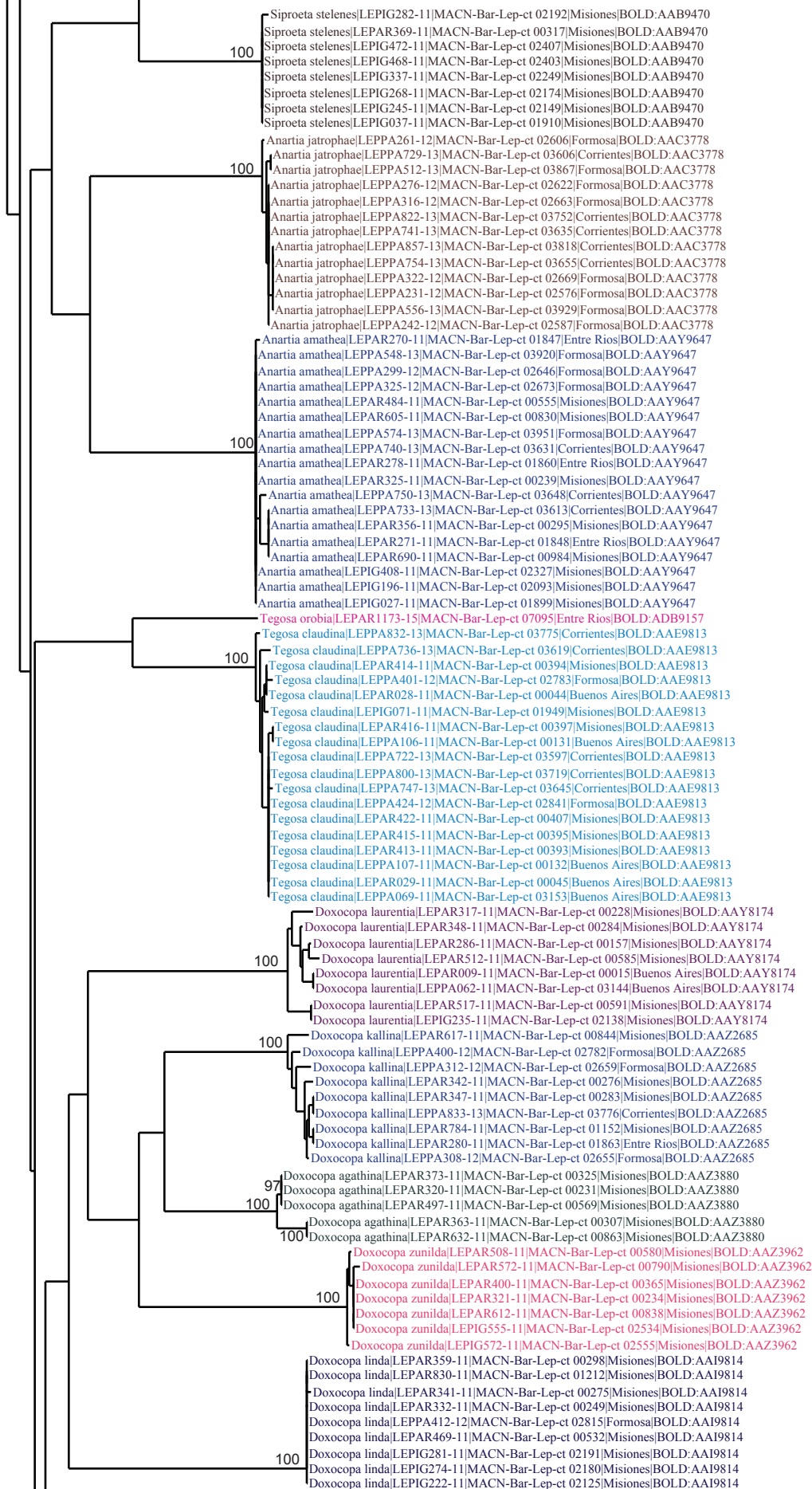

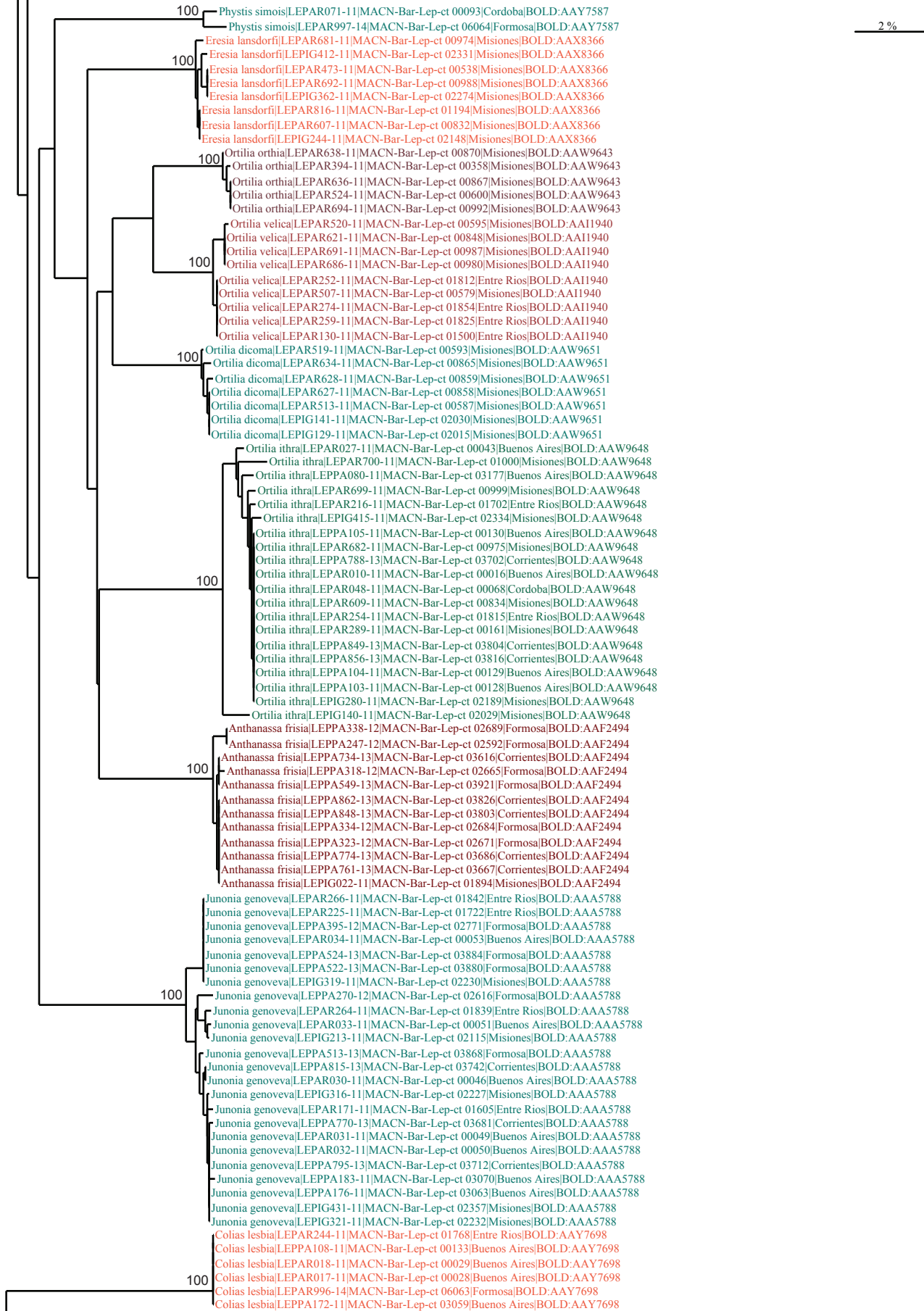

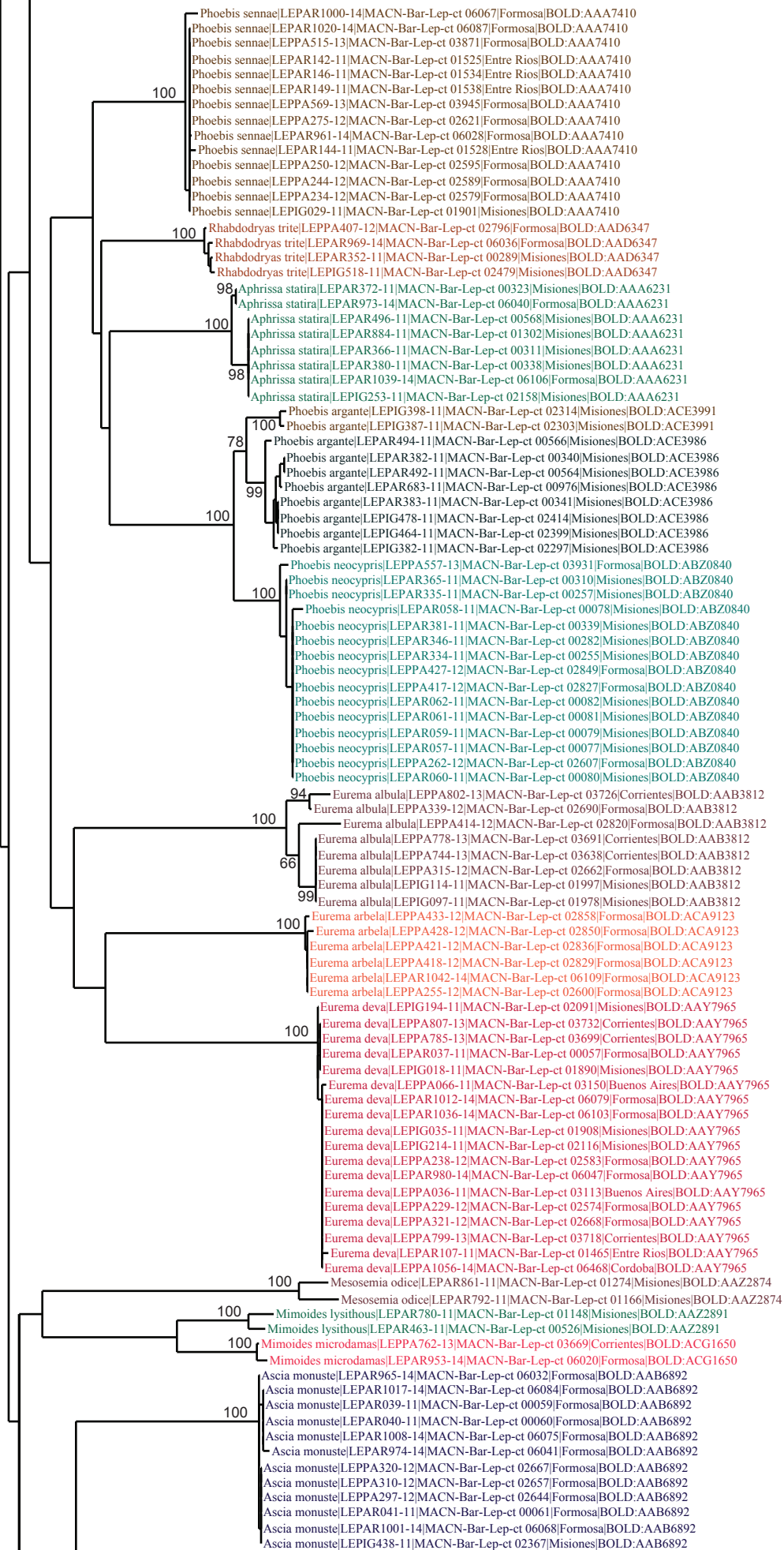

2 %

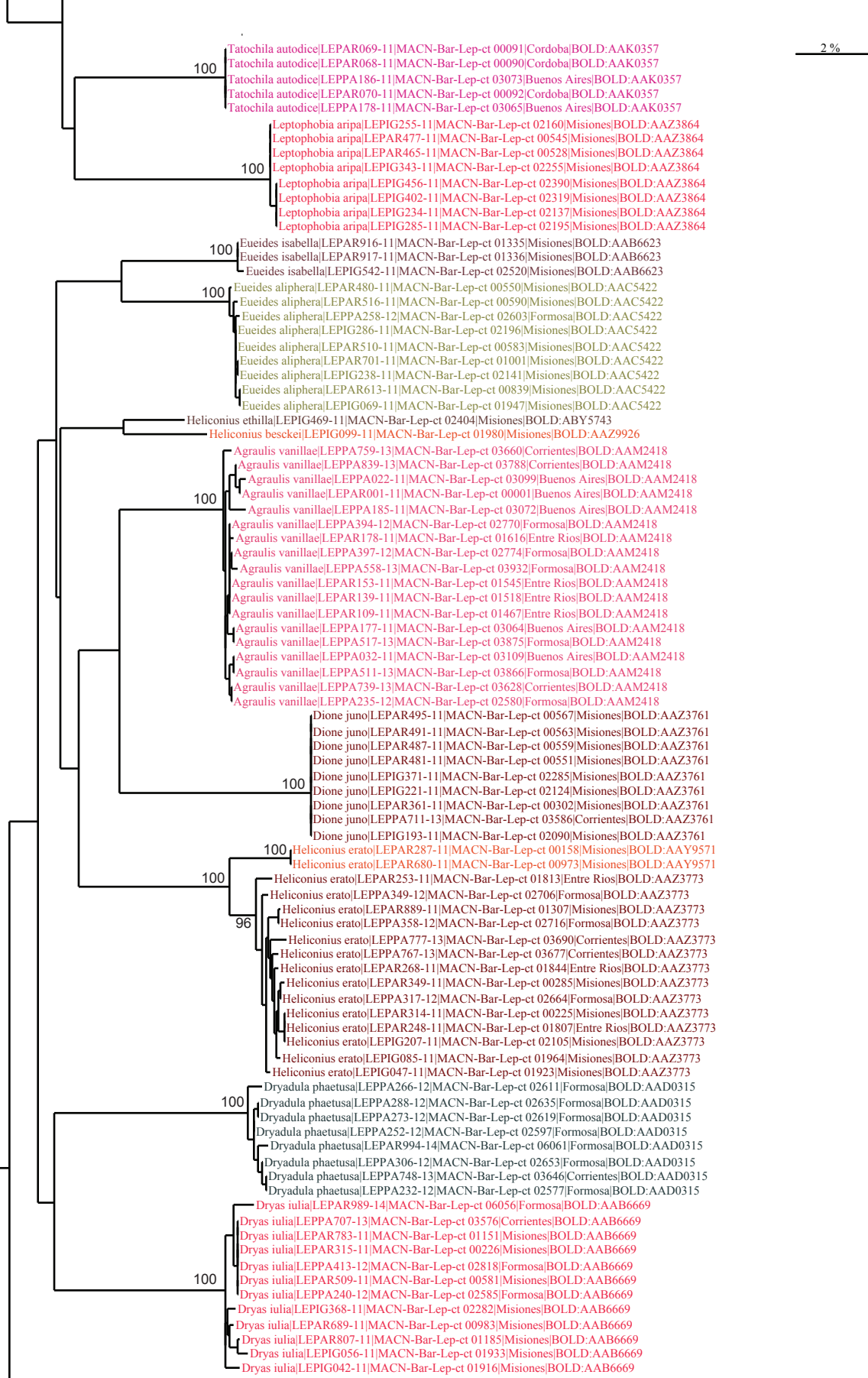

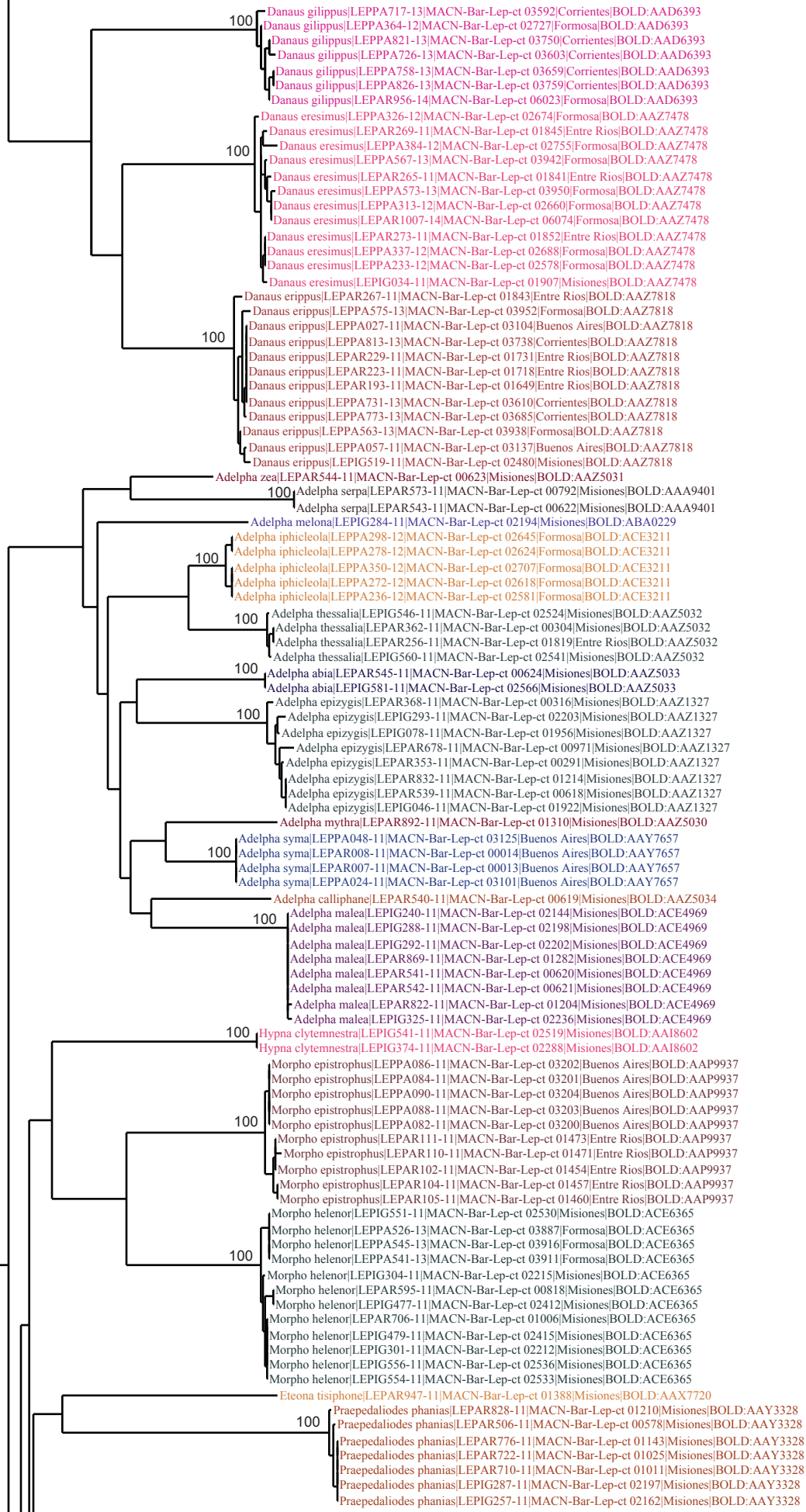

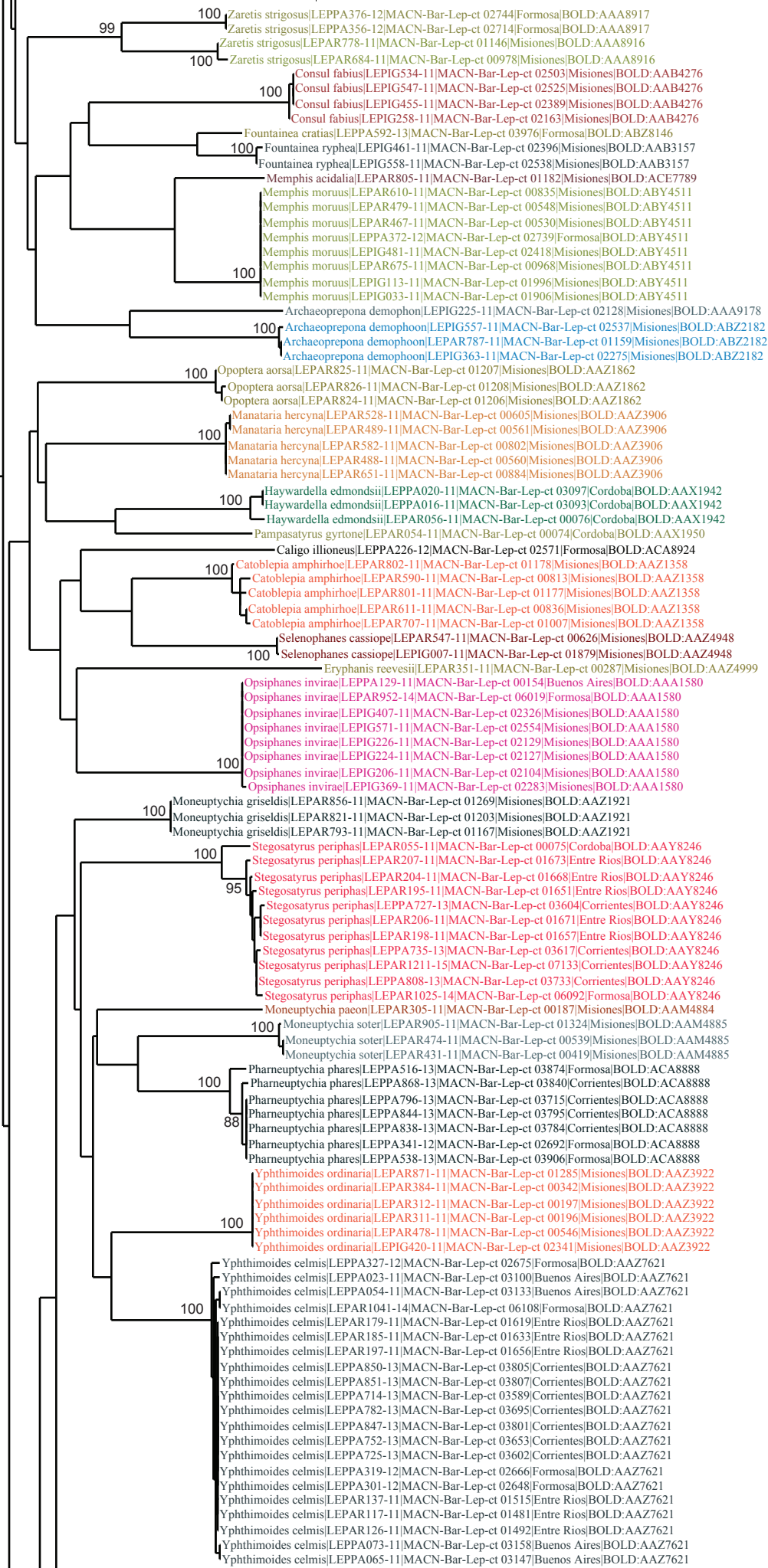

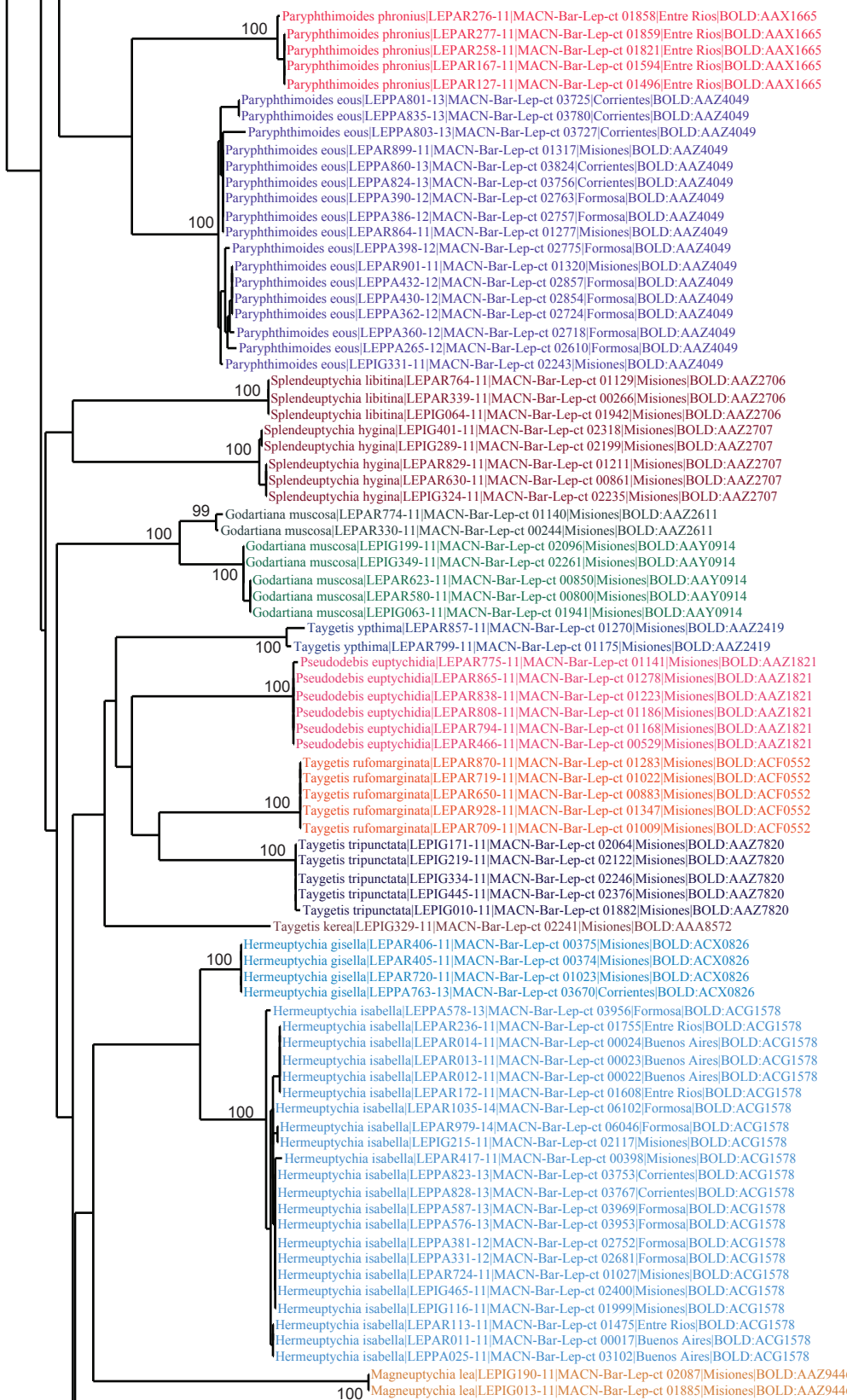

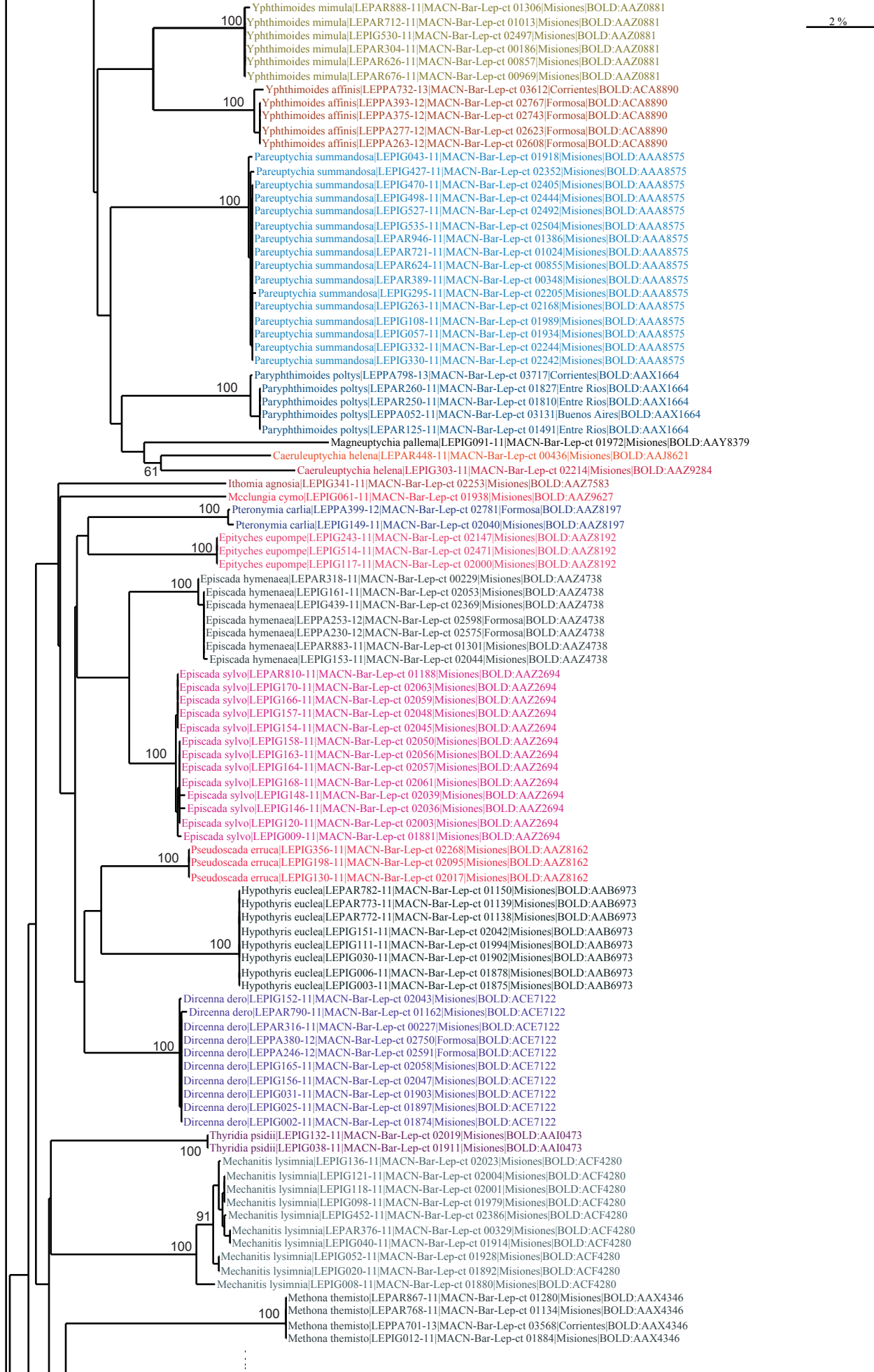

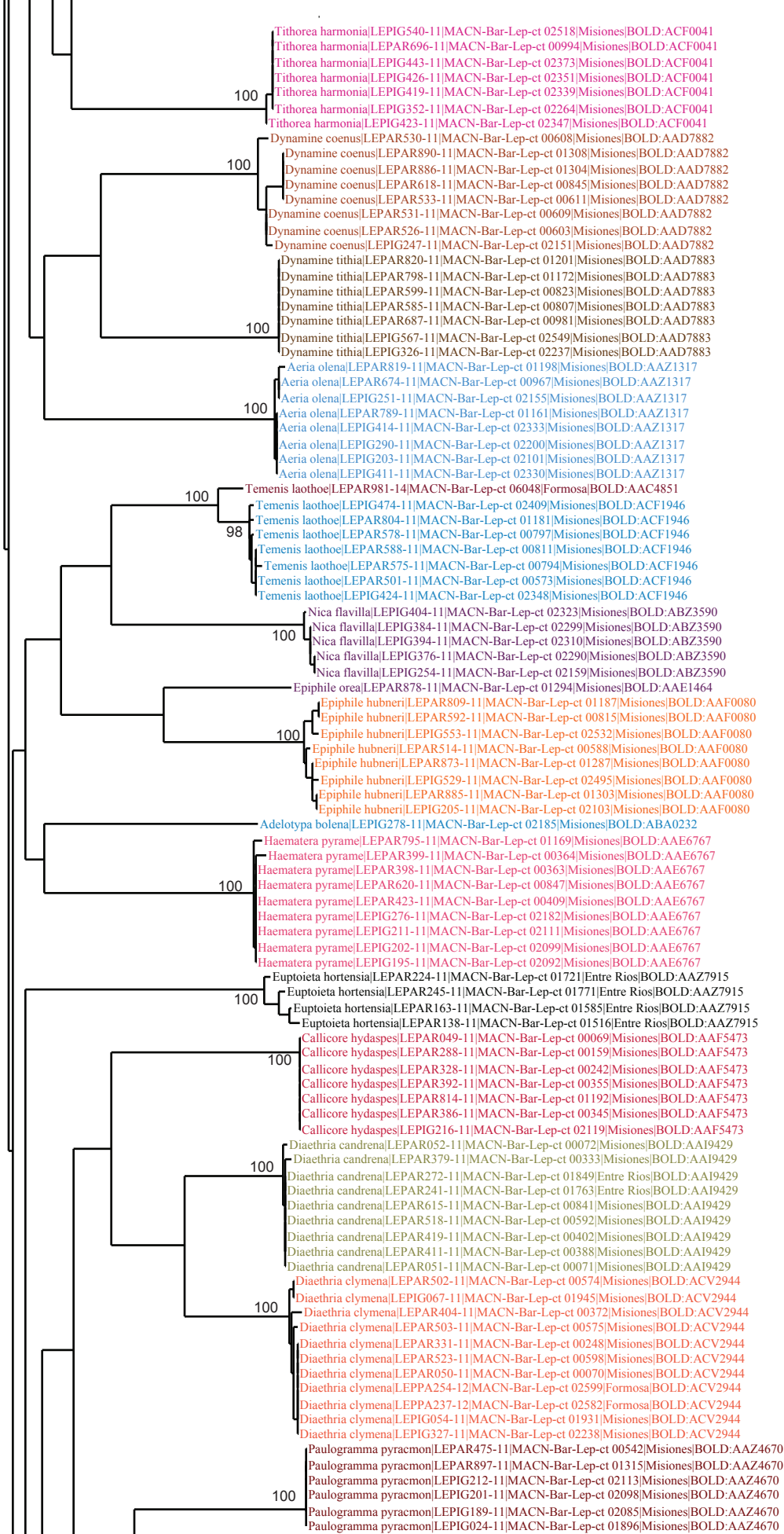



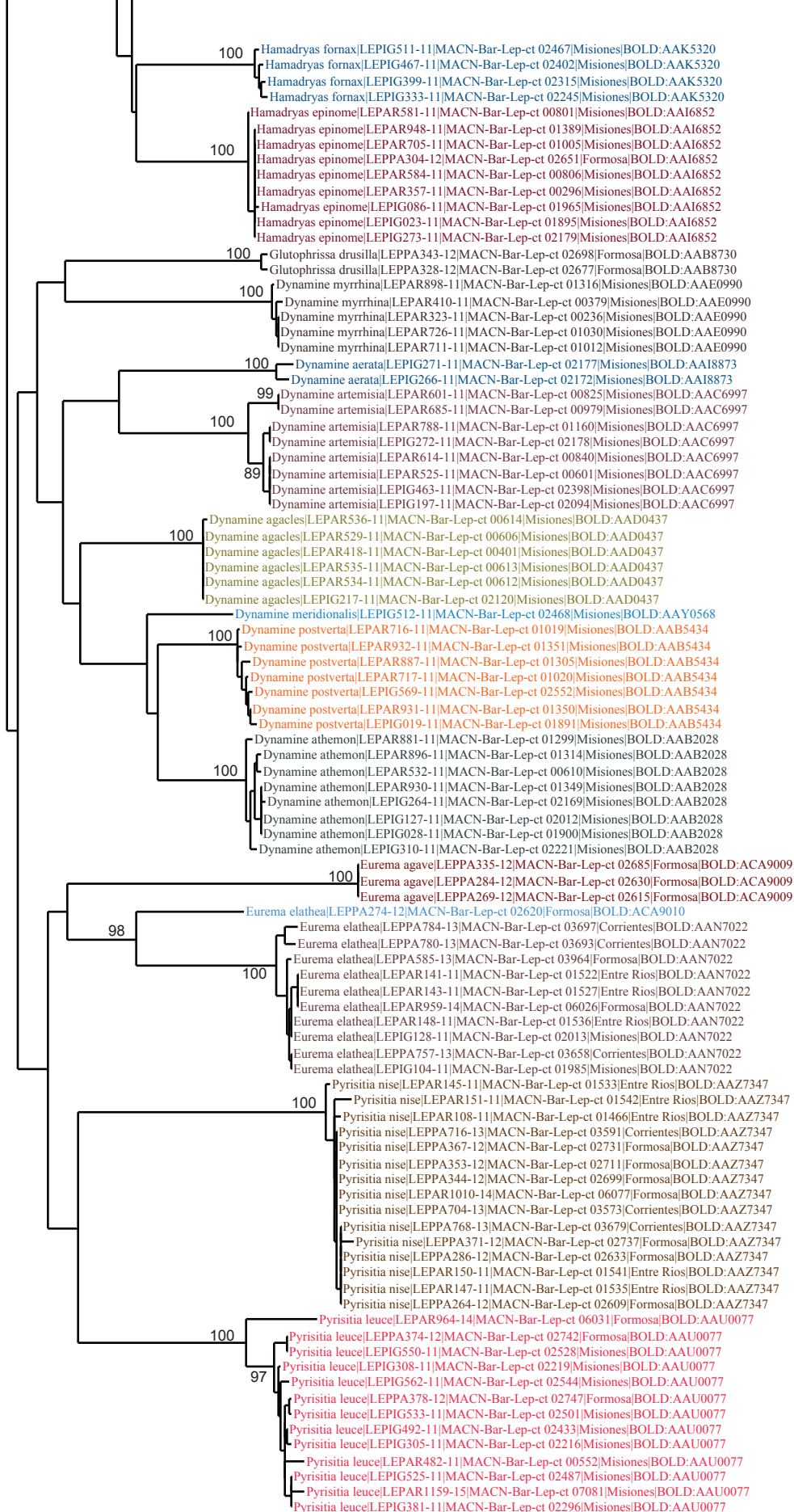

Supplement: S1 Fig — Colours indicate different BINs. Numbers above or below branches indicate bootstrap support based on 1,000 pseudoreplicates. (PDF) [file pone.0186845.s001.pdf]
