# Supplementary material for: Barcoding the butterflies of southern South America: Species delimitation efficacy, cryptic diversity and geographic patterns of divergence
Source: PLoS One. 2017 Oct 19;12(10):e0186845. doi: 10.1371/journal.pone.0186845 (PMC5648246; doi:10.1371/journal.pone.0186845)
Supplement: S1 Supporting Information — Summary of collection and sequence data for the 2,161 specimens collected (including the geographic coordinates for all collection sites), the results of the TCS and ABGD analyses, the congruence between species and MOTUs boundaries, and the results of the 42 Mantel tests performed. (PDF) [file pone.0186845.s003.pdf]

Table A. Summary of collection and sequence data for the 2,161 specimens collected. For more information check the public data set "DS-BUNEACAR" (dx.doi.org/10.5883/DS-BUNEACAR) on BOLD (www.boldsystems.org). For each specimen we specify the Sample and Process IDs, along with the year of collection and the locality where it was sampled (more detailed information on sampling localities is provided in Table B below). For those individuals that were successfully amplified we provide information on the length of the COI sequence together with the corresponding BIN information and GenBank accession number.

Identification	Sample ID	Process ID	BOLD's BIN	GenBank	COI-5P	Collection year	Locality
Achlyodes busirus	MACN-Bar-Lep-ct 01048	LEPAR741-11	BOLD:ACE3700	MF545708	658[0n]	2010	Misiones 2
Achlyodes busirus	MACN-Bar-Lep-ct 01156	LEPAR785-11	BOLD:ACE3700	MF545907	658[0n]	2010	Misiones 2
Achlyodes busirus	MACN-Bar-Lep-ct 01289	LEPAR875-11	BOLD:ACE3700	MF545434	658[0n]	2010	Misiones 2
Achlyodes busirus	MACN-Bar-Lep-ct 01909	LEPIG036-11	BOLD:ACE3700	MF545932	658[0n]	2011	Misiones 2
Achlyodes busirus	MACN-Bar-Lep-ct 01984	LEPIG103-11	BOLD:ABY6680	MF547172	658[0n]	2011	Misiones 2
Achlyodes busirus	MACN-Bar-Lep-ct 02422	LEPIG484-11	BOLD:ACE3700	MF545910	658[0n]	2011	Misiones 3
Actinote brylla	MACN-Bar-Lep-ct 02084	LEPIG188-11	BOLD:AAJ6347	MF546916	658[0n]	2011	Misiones 2
Actinote mamita	MACN-Bar-Lep-ct 00123	LEPPA098-11	BOLD:AAJ1938	MF546233	658[0n]	2010	Buenos Aires 21
Actinote mamita	MACN-Bar-Lep-ct 07151	LEPAR1229-15	BOLD:AAJ1938	MF546204	658[0n]	2012	Buenos Aires 24
Actinote mamita	MACN-Bar-Lep-ct 07153	LEPAR1231-15	BOLD:AAJ1938	MF546505	654[0n]	2010	Buenos Aires 29
Actinote melanisans	MACN-Bar-Lep-ct 01951	LEPIG073-11	BOLD:AAC7292	MF547094	658[0n]	2011	Misiones 2
Actinote melanisans	MACN-Bar-Lep-ct 02046	LEPIG155-11	BOLD:AAC7292	MF546994	658[0n]	2011	Misiones 2
Actinote melanisans	MACN-Bar-Lep-ct 07155	LEPAR1233-15			0	2007	Buenos Aires 20
Actinote pellenea	MACN-Bar-Lep-ct 00124	LEPPA099-11	BOLD:AAC7292	MF546533	658[0n]	2010	Buenos Aires 21
Actinote pellenea	MACN-Bar-Lep-ct 00125	LEPPA100-11	BOLD:AAC7292	MF546082	658[0n]	2010	Buenos Aires 21
Actinote pellenea	MACN-Bar-Lep-ct 00597	LEPAR522-11	BOLD:AAC7292	MF546825	658[0n]	2010	Misiones 2
Actinote pellenea	MACN-Bar-Lep-ct 00876	LEPAR643-11	BOLD:AAC7292	MF545885	658[0n]	2010	Misiones 2
Actinote pellenea	MACN-Bar-Lep-ct 02083	LEPIG187-11	BOLD:AAC7292	MF545951	658[0n]	2011	Misiones 2
Actinote pellenea	MACN-Bar-Lep-ct 02136	LEPIG233-11	BOLD:AAC7292	MF545558	658[0n]	2011	Misiones 2
Actinote pellenea	MACN-Bar-Lep-ct 02250	LEPIG338-11	BOLD:AAC7292	MF545579	658[0n]	2011	Misiones 3
Actinote pellenea	MACN-Bar-Lep-ct 03103	LEPPA026-11			0	2011	Buenos Aires 21
Actinote pellenea	MACN-Bar-Lep-ct 03106	LEPPA029-11			0	2011	Buenos Aires 21
Actinote pellenea	MACN-Bar-Lep-ct 03110	LEPPA033-11			0	2011	Buenos Aires 21
Actinote pellenea	MACN-Bar-Lep-ct 03116	LEPPA039-11			0	2011	Buenos Aires 21
Actinote pellenea	MACN-Bar-Lep-ct 03132	LEPPA053-11	BOLD:AAC7292	MF545562	658[0n]	2011	Buenos Aires 21
Actinote pellenea	MACN-Bar-Lep-ct 03136	LEPPA056-11	BOLD:AAC7292	MF546671	658[0n]	2011	Buenos Aires 21

Actinote pellenea	MACN-Bar-Lep-ct 03138	LEPPA058-11	BOLD:AAC7292	MF545711	658[0n]	2011	Buenos Aires 21
Actinote pellenea	MACN-Bar-Lep-ct 03154	LEPPA070-11	BOLD:AAC7292	MF547336	658[0n]	2011	Buenos Aires 21
Actinote pellenea	MACN-Bar-Lep-ct 03157	LEPPA072-11	BOLD:AAC7292	MF546922	658[0n]	2011	Buenos Aires 21
Actinote pellenea	MACN-Bar-Lep-ct 03164	LEPPA076-11	BOLD:AAC7292	MF546815	658[0n]	2011	Buenos Aires 21
Actinote pellenea	MACN-Bar-Lep-ct 03170	LEPPA078-11	BOLD:AAC7292	MF547050	658[0n]	2011	Buenos Aires 21
Actinote pellenea	MACN-Bar-Lep-ct 03181	LEPPA081-11	BOLD:AAC7292	MF546814	658[0n]	2011	Buenos Aires 21
Actinote pellenea	MACN-Bar-Lep-ct 03183	LEPPA083-11	BOLD:AAC7292	MF545612	658[0n]	2011	Buenos Aires 21
Actinote pellenea	MACN-Bar-Lep-ct 03185	LEPPA085-11	BOLD:AAC7292	MF545420	658[0n]	2011	Buenos Aires 21
Actinote pellenea	MACN-Bar-Lep-ct 03189	LEPPA087-11	BOLD:AAC7292	MF546829	658[0n]	2011	Buenos Aires 21
Actinote pellenea	MACN-Bar-Lep-ct 03191	LEPPA089-11	BOLD:AAC7292	MF547183	658[0n]	2011	Buenos Aires 21
Actinote pellenea	MACN-Bar-Lep-ct 03193	LEPPA091-11	BOLD:AAC7292	MF546005	658[0n]	2011	Buenos Aires 21
Actinote pellenea	MACN-Bar-Lep-ct 03195	LEPPA093-11	BOLD:AAC7292	MF545546	658[0n]	2011	Buenos Aires 21
Actinote pellenea	MACN-Bar-Lep-ct 03197	LEPPA095-11	BOLD:AAC7292	MF545842	658[0n]	2011	Buenos Aires 21
Actinote pellenea	MACN-Bar-Lep-ct 03701	LEPPA787-13	BOLD:AAC7292	MF547325	658[0n]	2011	Corrientes 1
Actinote pellenea	MACN-Bar-Lep-ct 07082	LEPAR1160-15			0	2005	Buenos Aires 13
Actinote pellenea	MACN-Bar-Lep-ct 07084	LEPAR1162-15			0	2002	Buenos Aires 23
Actinote pellenea	MACN-Bar-Lep-ct 07086	LEPAR1164-15			0	2010	Buenos Aires 29
Actinote pellenea	MACN-Bar-Lep-ct 07088	LEPAR1166-15			0	2010	Buenos Aires 29
Actinote pellenea	MACN-Bar-Lep-ct 07090	LEPAR1168-15	BOLD:AAC7292	MF546288	658[0n]	2014	Chaco 1
Actinote pellenea	MACN-Bar-Lep-ct 07092	LEPAR1170-15			0	2011	Entre Ríos 1
Actinote pellenea	MACN-Bar-Lep-ct 07094	LEPAR1172-15			0	2010	Buenos Aires 29
Actinote pellenea	MACN-Bar-Lep-ct 07096	LEPAR1174-15			0	2008	Buenos Aires 29
Actinote pellenea	MACN-Bar-Lep-ct 07098	LEPAR1176-15			0	2010	Buenos Aires 29
Actinote pellenea	MACN-Bar-Lep-ct 07100	LEPAR1178-15			0	2009	Buenos Aires 29
Actinote pellenea	MACN-Bar-Lep-ct 07102	LEPAR1180-15			0	2001	Buenos Aires 8
Actinote pellenea	MACN-Bar-Lep-ct 07104	LEPAR1182-15			0	2013	Entre Ríos 7
Actinote pellenea	MACN-Bar-Lep-ct 07106	LEPAR1184-15			0	2002	Buenos Aires 13
Actinote pellenea	MACN-Bar-Lep-ct 07108	LEPAR1186-15			0	2001	Buenos Aires 23
Actinote pellenea	MACN-Bar-Lep-ct 07110	LEPAR1188-15			0	2006	Buenos Aires 29
Actinote pellenea	MACN-Bar-Lep-ct 07112	LEPAR1190-15			0	2000	Buenos Aires 24
Actinote pellenea	MACN-Bar-Lep-ct 07114	LEPAR1192-15			0	2003	Buenos Aires 11
Actinote pellenea	MACN-Bar-Lep-ct 07116	LEPAR1194-15	BOLD:AAC7292	MF545644	658[0n]	2014	Entre Ríos 7
Actinote pellenea	MACN-Bar-Lep-ct 07118	LEPAR1196-15			0	2013	Buenos Aires 25

Actinote pellenea	MACN-Bar-Lep-ct 07120	LEPAR1198-15			0	2001	Buenos Aires 18
Actinote pellenea	MACN-Bar-Lep-ct 07122	LEPAR1200-15			0	2002	Misiones 7
Actinote pellenea	MACN-Bar-Lep-ct 07124	LEPAR1202-15			0	1996	Misiones 6
Actinote pellenea	MACN-Bar-Lep-ct 07126	LEPAR1204-15			0	2009	Buenos Aires 29
Actinote pellenea	MACN-Bar-Lep-ct 07128	LEPAR1206-15			0	2000	Buenos Aires 22
Actinote pellenea	MACN-Bar-Lep-ct 07130	LEPAR1208-15			0	2006	Buenos Aires 29
Actinote pellenea	MACN-Bar-Lep-ct 07132	LEPAR1210-15			0	2004	Buenos Aires 19
Actinote pellenea	MACN-Bar-Lep-ct 07134	LEPAR1212-15	BOLD:AAC7292	MF545917	644[0n]	2013	Buenos Aires 26
Actinote pellenea	MACN-Bar-Lep-ct 07136	LEPAR1214-15			0	2001	Buenos Aires 27
Actinote pellenea	MACN-Bar-Lep-ct 07138	LEPAR1216-15			0	2009	Córdoba 3
Actinote pellenea	MACN-Bar-Lep-ct 07140	LEPAR1218-15			0	2006	Buenos Aires 29
Actinote pellenea	MACN-Bar-Lep-ct 07142	LEPAR1220-15			0	2006	Buenos Aires 29
Actinote pellenea	MACN-Bar-Lep-ct 07144	LEPAR1222-15			0	2001	Buenos Aires 27
Actinote pellenea	MACN-Bar-Lep-ct 07146	LEPAR1224-15			0	2001	Buenos Aires 27
Actinote pellenea	MACN-Bar-Lep-ct 07148	LEPAR1226-15			0	2001	Buenos Aires 8
Actinote pellenea	MACN-Bar-Lep-ct 07156	LEPAR1234-15	BOLD:AAC7292	MF547035	636[0n]	2014	Buenos Aires 20
Actinote pyrrha	MACN-Bar-Lep-ct 07149	LEPAR1227-15			0	2011	Misiones 9
Actinote pyrrha	MACN-Bar-Lep-ct 07152	LEPAR1230-15			0	2010	Buenos Aires 8
Actinote pyrrha	MACN-Bar-Lep-ct 07154	LEPAR1232-15			0	2010	Buenos Aires 27
Adelotypa bolena	MACN-Bar-Lep-ct 02185	LEPIG278-11	BOLD:ABA0232	MF546320	658[0n]	2011	Misiones 3
Adelpha abia	MACN-Bar-Lep-ct 00624	LEPAR545-11	BOLD:AAZ5033	MF547327	658[0n]	2010	Misiones 2
Adelpha abia	MACN-Bar-Lep-ct 02566	LEPIG581-11	BOLD:AAZ5033	MF546151	658[0n]	2011	Misiones 3
Adelpha calliphane	MACN-Bar-Lep-ct 00619	LEPAR540-11	BOLD:AAZ5034	MF547128	658[0n]	2010	Misiones 2
Adelpha epizygis	MACN-Bar-Lep-ct 00291	LEPAR353-11	BOLD:AAZ1327	MF546901	658[0n]	2010	Misiones 2
Adelpha epizygis	MACN-Bar-Lep-ct 00316	LEPAR368-11	BOLD:AAZ1327	MF545519	658[0n]	2010	Misiones 2
Adelpha epizygis	MACN-Bar-Lep-ct 00618	LEPAR539-11	BOLD:AAZ1327	MF546226	658[0n]	2010	Misiones 2
Adelpha epizygis	MACN-Bar-Lep-ct 00971	LEPAR678-11	BOLD:AAZ1327	MF546928	658[0n]	2010	Misiones 2
Adelpha epizygis	MACN-Bar-Lep-ct 01214	LEPAR832-11	BOLD:AAZ1327	MF546037	658[0n]	2010	Misiones 2
Adelpha epizygis	MACN-Bar-Lep-ct 01922	LEPIG046-11	BOLD:AAZ1327	MF546523	658[0n]	2011	Misiones 2
Adelpha epizygis	MACN-Bar-Lep-ct 01956	LEPIG078-11	BOLD:AAZ1327	MF546118	658[0n]	2011	Misiones 2
Adelpha epizygis	MACN-Bar-Lep-ct 02203	LEPIG293-11	BOLD:AAZ1327	MF546450	658[0n]	2011	Misiones 3
Adelpha iphicleola	MACN-Bar-Lep-ct 02581	LEPPA236-12	BOLD:ACE3211	MF545714	658[0n]	2011	Formosa 2
Adelpha iphicleola	MACN-Bar-Lep-ct 02618	LEPPA272-12	BOLD:ACE3211	MF546985	658[0n]	2011	Formosa 2

Adelpha iphicleola	MACN-Bar-Lep-ct 02624	LEPPA278-12	BOLD:ACE3211	MF546680	658[0n]	2011	Formosa 2
Adelpha iphicleola	MACN-Bar-Lep-ct 02645	LEPPA298-12	BOLD:ACE3211	MF546846	658[0n]	2011	Formosa 2
Adelpha iphicleola	MACN-Bar-Lep-ct 02707	LEPPA350-12	BOLD:ACE3211	MF546179	658[0n]	2011	Formosa 3
Adelpha malea	MACN-Bar-Lep-ct 00620	LEPAR541-11	BOLD:ACE4969	MF546712	658[0n]	2010	Misiones 2
Adelpha malea	MACN-Bar-Lep-ct 00621	LEPAR542-11	BOLD:ACE4969	MF546793	658[0n]	2010	Misiones 2
Adelpha malea	MACN-Bar-Lep-ct 01204	LEPAR822-11	BOLD:ACE4969	MF546499	658[0n]	2010	Misiones 2
Adelpha malea	MACN-Bar-Lep-ct 01282	LEPAR869-11	BOLD:ACE4969	MF545672	658[0n]	2010	Misiones 2
Adelpha malea	MACN-Bar-Lep-ct 02144	LEPIG240-11	BOLD:ACE4969	MF545459	658[0n]	2011	Misiones 3
Adelpha malea	MACN-Bar-Lep-ct 02198	LEPIG288-11	BOLD:ACE4969	MF546606	658[0n]	2011	Misiones 3
Adelpha malea	MACN-Bar-Lep-ct 02202	LEPIG292-11	BOLD:ACE4969	MF547055	658[0n]	2011	Misiones 3
Adelpha malea	MACN-Bar-Lep-ct 02236	LEPIG325-11	BOLD:ACE4969	MF547254	658[0n]	2011	Misiones 3
Adelpha melona	MACN-Bar-Lep-ct 02194	LEPIG284-11	BOLD:ABA0229	MF546649	658[0n]	2011	Misiones 3
Adelpha mythra	MACN-Bar-Lep-ct 01310	LEPAR892-11	BOLD:AAZ5030	MF547293	658[0n]	2010	Misiones 2
Adelpha serpa	MACN-Bar-Lep-ct 00622	LEPAR543-11	BOLD:AAA9401	MF546638	658[0n]	2010	Misiones 2
Adelpha serpa	MACN-Bar-Lep-ct 00792	LEPAR573-11	BOLD:AAA9401	MF545942	658[0n]	2010	Misiones 2
Adelpha syma	MACN-Bar-Lep-ct 00013	LEPAR007-11	BOLD:AAY7657	MF546886	658[0n]	2010	Buenos Aires 21
Adelpha syma	MACN-Bar-Lep-ct 00014	LEPAR008-11	BOLD:AAY7657	MF546125	658[0n]	2010	Buenos Aires 21
Adelpha syma	MACN-Bar-Lep-ct 03101	LEPPA024-11	BOLD:AAY7657	MF546298	658[0n]	2011	Buenos Aires 21
Adelpha syma	MACN-Bar-Lep-ct 03108	LEPPA031-11			0	2011	Buenos Aires 21
Adelpha syma	MACN-Bar-Lep-ct 03125	LEPPA048-11	BOLD:AAY7657	MF546000	658[0n]	2011	Buenos Aires 21
Adelpha thessalia	MACN-Bar-Lep-ct 00304	LEPAR362-11	BOLD:AAZ5032	MF545408	658[0n]	2010	Misiones 2
Adelpha thessalia	MACN-Bar-Lep-ct 01819	LEPAR256-11	BOLD:AAZ5032	MF545753	658[0n]	2011	Entre Ríos 3
Adelpha thessalia	MACN-Bar-Lep-ct 02524	LEPIG546-11	BOLD:AAZ5032	MF546648	658[0n]	2011	Misiones 3
Adelpha thessalia	MACN-Bar-Lep-ct 02541	LEPIG560-11	BOLD:AAZ5032	MF545617	658[0n]	2011	Misiones 3
Adelpha zea	MACN-Bar-Lep-ct 00623	LEPAR544-11	BOLD:AAZ5031	MF545561	658[0n]	2010	Misiones 2
Adlerodea modesta	MACN-Bar-Lep-ct 02080	LEPIG184-11	BOLD:AAL6917	MF545580	658[0n]	2011	Misiones 2
Aeria olena	MACN-Bar-Lep-ct 00967	LEPAR674-11	BOLD:AAZ1317	MF547000	658[0n]	2010	Misiones 2
Aeria olena	MACN-Bar-Lep-ct 01161	LEPAR789-11	BOLD:AAZ1317	MF547158	658[0n]	2010	Misiones 2
Aeria olena	MACN-Bar-Lep-ct 01198	LEPAR819-11	BOLD:AAZ1317	MF546049	658[0n]	2010	Misiones 2
Aeria olena	MACN-Bar-Lep-ct 02101	LEPIG203-11	BOLD:AAZ1317	MF546696	658[0n]	2011	Misiones 2
Aeria olena	MACN-Bar-Lep-ct 02155	LEPIG251-11	BOLD:AAZ1317	MF545846	658[0n]	2011	Misiones 3
Aeria olena	MACN-Bar-Lep-ct 02200	LEPIG290-11	BOLD:AAZ1317	MF547379	658[0n]	2011	Misiones 3
Aeria olena	MACN-Bar-Lep-ct 02330	LEPIG411-11	BOLD:AAZ1317	MF546374	658[0n]	2011	Misiones 3

Aeria olena	MACN-Bar-Lep-ct 02333	LEPIG414-11	BOLD:AAZ1317	MF545589	658[0n]	2011	Misiones 3
Aethilla echina	MACN-Bar-Lep-ct 00536	LEPAR472-11	BOLD:ACE9423	MF545658	658[0n]	2010	Misiones 2
Aethilla echina	MACN-Bar-Lep-ct 01008	LEPAR708-11	BOLD:ACE9423	MF547354	658[0n]	2010	Misiones 2
Aethilla echina	MACN-Bar-Lep-ct 01209	LEPAR827-11	BOLD:ACE9423	MF545389	658[0n]	2010	Misiones 2
Aethilla echina	MACN-Bar-Lep-ct 01217	LEPAR834-11	BOLD:ACE9423	MF547066	658[0n]	2010	Misiones 2
Aethilla echina	MACN-Bar-Lep-ct 01288	LEPAR874-11	BOLD:ACE9423	MF545843	658[0n]	2010	Misiones 2
Aethilla echina	MACN-Bar-Lep-ct 01977	LEPIG096-11	BOLD:ACE9423	MF545692	658[0n]	2011	Misiones 2
Aethilla echina	MACN-Bar-Lep-ct 02293	LEPIG378-11	BOLD:ACE9423	MF546644	658[0n]	2011	Misiones 3
Aethilla echina	MACN-Bar-Lep-ct 02506	LEPIG536-11	BOLD:ACE9423	MF546811	658[0n]	2011	Misiones 3
Agraulis vanillae	MACN-Bar-Lep-ct 00001	LEPAR001-11	BOLD:AAM2418	MF545787	658[0n]	2010	Buenos Aires 21
Agraulis vanillae	MACN-Bar-Lep-ct 01467	LEPAR109-11	BOLD:AAM2418	MF546912	658[0n]	2011	Entre Ríos 3
Agraulis vanillae	MACN-Bar-Lep-ct 01518	LEPAR139-11	BOLD:AAM2418	MF547240	658[0n]	2011	Entre Ríos 4
Agraulis vanillae	MACN-Bar-Lep-ct 01545	LEPAR153-11	BOLD:AAM2418	MF545655	658[0n]	2011	Entre Ríos 3
Agraulis vanillae	MACN-Bar-Lep-ct 01616	LEPAR178-11	BOLD:AAM2418	MF545390	658[0n]	2011	Entre Ríos 5
Agraulis vanillae	MACN-Bar-Lep-ct 02580	LEPPA235-12	BOLD:AAM2418	MF546126	658[0n]	2011	Formosa 2
Agraulis vanillae	MACN-Bar-Lep-ct 02770	LEPPA394-12	BOLD:AAM2418	MF545948	658[0n]	2011	Formosa 3
Agraulis vanillae	MACN-Bar-Lep-ct 02774	LEPPA397-12	BOLD:AAM2418	MF546725	658[0n]	2011	Formosa 2
Agraulis vanillae	MACN-Bar-Lep-ct 03064	LEPPA177-11	BOLD:AAM2418	MF545756	658[0n]	2011	Buenos Aires 6
Agraulis vanillae	MACN-Bar-Lep-ct 03072	LEPPA185-11	BOLD:AAM2418	MF546808	631[0n]	2011	Buenos Aires 6
Agraulis vanillae	MACN-Bar-Lep-ct 03099	LEPPA022-11	BOLD:AAM2418	MF546576	658[0n]	2011	Buenos Aires 21
Agraulis vanillae	MACN-Bar-Lep-ct 03109	LEPPA032-11	BOLD:AAM2418	MF546410	658[0n]	2011	Buenos Aires 21
Agraulis vanillae	MACN-Bar-Lep-ct 03628	LEPPA739-13	BOLD:AAM2418	MF546894	658[0n]	2011	Corrientes 1
Agraulis vanillae	MACN-Bar-Lep-ct 03660	LEPPA759-13	BOLD:AAM2418	MF547188	658[0n]	2011	Corrientes 1
Agraulis vanillae	MACN-Bar-Lep-ct 03788	LEPPA839-13	BOLD:AAM2418	MF546413	658[0n]	2011	Corrientes 1
Agraulis vanillae	MACN-Bar-Lep-ct 03866	LEPPA511-13	BOLD:AAM2418	MF546963	658[0n]	2012	Formosa 4
Agraulis vanillae	MACN-Bar-Lep-ct 03875	LEPPA517-13	BOLD:AAM2418	MF546978	658[0n]	2012	Formosa 4
Agraulis vanillae	MACN-Bar-Lep-ct 03932	LEPPA558-13	BOLD:AAM2418	MF547181	621[0n]	2012	Formosa 4
Agraulis vanillae	MACN-Bar-Lep-ct 06463	LEPPA1051-14			0	2013	Córdoba 4
Alera metallica	MACN-Bar-Lep-ct 01926	LEPIG050-11	BOLD:AAZ7289	MF545694	658[0n]	2011	Misiones 2
Anartia amathea	MACN-Bar-Lep-ct 00239	LEPAR325-11	BOLD:AAY9647	MF546441	658[0n]	2010	Misiones 2
Anartia amathea	MACN-Bar-Lep-ct 00295	LEPAR356-11	BOLD:AAY9647	MF547064	658[0n]	2010	Misiones 2
Anartia amathea	MACN-Bar-Lep-ct 00555	LEPAR484-11	BOLD:AAY9647	MF546217	658[0n]	2010	Misiones 2
Anartia amathea	MACN-Bar-Lep-ct 00830	LEPAR605-11	BOLD:AAY9647	MF545804	658[0n]	2010	Misiones 2

Anartia amathea	MACN-Bar-Lep-ct 00984	LEPAR690-11	BOLD:AAY9647	MF546107	658[0n]	2010	Misiones 2
Anartia amathea	MACN-Bar-Lep-ct 01847	LEPAR270-11	BOLD:AAY9647	MF546974	658[0n]	2011	Entre Ríos 3
Anartia amathea	MACN-Bar-Lep-ct 01848	LEPAR271-11	BOLD:AAY9647	MF546140	658[0n]	2011	Entre Ríos 3
Anartia amathea	MACN-Bar-Lep-ct 01860	LEPAR278-11	BOLD:AAY9647	MF545523	658[0n]	2011	Entre Ríos 3
Anartia amathea	MACN-Bar-Lep-ct 01899	LEPIG027-11	BOLD:AAY9647	MF545501	658[0n]	2011	Misiones 2
Anartia amathea	MACN-Bar-Lep-ct 02093	LEPIG196-11	BOLD:AAY9647	MF545839	658[0n]	2011	Misiones 2
Anartia amathea	MACN-Bar-Lep-ct 02327	LEPIG408-11	BOLD:AAY9647	MF545569	658[0n]	2011	Misiones 3
Anartia amathea	MACN-Bar-Lep-ct 02646	LEPPA299-12	BOLD:AAY9647	MF545958	658[0n]	2011	Formosa 2
Anartia amathea	MACN-Bar-Lep-ct 02673	LEPPA325-12	BOLD:AAY9647	MF545432	658[0n]	2011	Formosa 2
Anartia amathea	MACN-Bar-Lep-ct 03613	LEPPA733-13	BOLD:AAY9647	MF547076	658[0n]	2011	Corrientes 1
Anartia amathea	MACN-Bar-Lep-ct 03631	LEPPA740-13	BOLD:AAY9647	MF545405	658[0n]	2011	Corrientes 1
Anartia amathea	MACN-Bar-Lep-ct 03648	LEPPA750-13	BOLD:AAY9647	MF547285	596[0n]	2011	Corrientes 1
Anartia amathea	MACN-Bar-Lep-ct 03914	LEPPA543-13			0	2012	Formosa 4
Anartia amathea	MACN-Bar-Lep-ct 03920	LEPPA548-13	BOLD:AAY9647	MF545551	658[0n]	2012	Formosa 4
Anartia amathea	MACN-Bar-Lep-ct 03951	LEPPA574-13	BOLD:AAY9647	MF546434	658[0n]	2012	Formosa 4
Anartia jatrophae	MACN-Bar-Lep-ct 02576	LEPPA231-12	BOLD:AAC3778	MF547261	658[0n]	2011	Formosa 2
Anartia jatrophae	MACN-Bar-Lep-ct 02587	LEPPA242-12	BOLD:AAC3778	MF545861	658[0n]	2011	Formosa 2
Anartia jatrophae	MACN-Bar-Lep-ct 02606	LEPPA261-12	BOLD:AAC3778	MF546031	658[0n]	2011	Formosa 2
Anartia jatrophae	MACN-Bar-Lep-ct 02622	LEPPA276-12	BOLD:AAC3778	MF547282	658[0n]	2011	Formosa 2
Anartia jatrophae	MACN-Bar-Lep-ct 02663	LEPPA316-12	BOLD:AAC3778	MF547308	658[0n]	2011	Formosa 2
Anartia jatrophae	MACN-Bar-Lep-ct 02669	LEPPA322-12	BOLD:AAC3778	MF546478	658[0n]	2011	Formosa 2
Anartia jatrophae	MACN-Bar-Lep-ct 03606	LEPPA729-13	BOLD:AAC3778	MF546738	658[0n]	2011	Corrientes 1
Anartia jatrophae	MACN-Bar-Lep-ct 03635	LEPPA741-13	BOLD:AAC3778	MF545895	658[0n]	2011	Corrientes 1
Anartia jatrophae	MACN-Bar-Lep-ct 03655	LEPPA754-13	BOLD:AAC3778	MF546749	658[0n]	2011	Corrientes 1
Anartia jatrophae	MACN-Bar-Lep-ct 03752	LEPPA822-13	BOLD:AAC3778	MF547307	658[0n]	2011	Corrientes 1
Anartia jatrophae	MACN-Bar-Lep-ct 03818	LEPPA857-13	BOLD:AAC3778	MF546583	658[0n]	2011	Corrientes 1
Anartia jatrophae	MACN-Bar-Lep-ct 03867	LEPPA512-13	BOLD:AAC3778	MF545474	621[0n]	2012	Formosa 4
Anartia jatrophae	MACN-Bar-Lep-ct 03929	LEPPA556-13	BOLD:AAC3778	MF546921	658[0n]	2012	Formosa 4
Anartia jatrophae	MACN-Bar-Lep-ct 03948	LEPPA571-13			0	2012	Formosa 4
Anastrus sempiternus	MACN-Bar-Lep-ct 00604	LEPAR527-11	BOLD:AAB4781	MF545588	658[0n]	2010	Misiones 2
Anastrus sempiternus	MACN-Bar-Lep-ct 01325	LEPAR906-11	BOLD:AAB4781	MF546745	658[0n]	2010	Misiones 2
Anastrus sempiternus	MACN-Bar-Lep-ct 01950	LEPIG072-11	BOLD:AAB4781	MF545872	658[0n]	2011	Misiones 2
Ancyloxypha nitedula	MACN-Bar-Lep-ct 00027	LEPAR016-11	BOLD:AAY8306	MF546199	658[0n]	2010	Buenos Aires 21

Anisochoria sublimbata	MACN-Bar-Lep-ct 00793	LEPAR574-11	BOLD:AAZ5024	MF546364	658[0n]	2010	Misiones 2
Anisochoria sublimbata	MACN-Bar-Lep-ct 02424	LEPIG486-11	BOLD:AAZ5024	MF545502	658[0n]	2011	Misiones 3
Anthanassa frisia	MACN-Bar-Lep-ct 01894	LEPIG022-11	BOLD:AAF2494	MF546279	658[0n]	2011	Misiones 2
Anthanassa frisia	MACN-Bar-Lep-ct 02592	LEPPA247-12	BOLD:AAF2494	MF545731	658[0n]	2011	Formosa 2
Anthanassa frisia	MACN-Bar-Lep-ct 02665	LEPPA318-12	BOLD:AAF2494	MF545657	658[0n]	2011	Formosa 2
Anthanassa frisia	MACN-Bar-Lep-ct 02671	LEPPA323-12	BOLD:AAF2494	MF546751	658[0n]	2011	Formosa 2
Anthanassa frisia	MACN-Bar-Lep-ct 02684	LEPPA334-12	BOLD:AAF2494	MF546129	658[0n]	2011	Formosa 2
Anthanassa frisia	MACN-Bar-Lep-ct 02689	LEPPA338-12	BOLD:AAF2494	MF547195	658[0n]	2011	Formosa 2
Anthanassa frisia	MACN-Bar-Lep-ct 03616	LEPPA734-13	BOLD:AAF2494	MF546150	658[0n]	2011	Corrientes 1
Anthanassa frisia	MACN-Bar-Lep-ct 03667	LEPPA761-13	BOLD:AAF2494	MF545673	658[0n]	2011	Corrientes 1
Anthanassa frisia	MACN-Bar-Lep-ct 03686	LEPPA774-13	BOLD:AAF2494	MF546470	658[0n]	2011	Corrientes 1
Anthanassa frisia	MACN-Bar-Lep-ct 03803	LEPPA848-13	BOLD:AAF2494	MF545911	658[0n]	2011	Corrientes 1
Anthanassa frisia	MACN-Bar-Lep-ct 03826	LEPPA862-13	BOLD:AAF2494	MF546324	618[0n]	2011	Corrientes 1
Anthanassa frisia	MACN-Bar-Lep-ct 03921	LEPPA549-13	BOLD:AAF2494	MF546596	658[0n]	2012	Formosa 4
Anthoptus epictetus	MACN-Bar-Lep-ct 02248	LEPIG336-11			0	2011	Misiones 3
Anthoptus epictetus	MACN-Bar-Lep-ct 02272	LEPIG360-11	BOLD:AAB0089	MF547011	658[0n]	2011	Misiones 3
Antigonus liborius	MACN-Bar-Lep-ct 02450	LEPIG503-11	BOLD:AAZ9638	MF546615	658[0n]	2011	Misiones 3
Aphrissa statira	MACN-Bar-Lep-ct 00311	LEPAR366-11	BOLD:AAA6231	MF546084	658[0n]	2010	Misiones 2
Aphrissa statira	MACN-Bar-Lep-ct 00323	LEPAR372-11	BOLD:AAA6231	MF545976	658[0n]	2010	Misiones 2
Aphrissa statira	MACN-Bar-Lep-ct 00338	LEPAR380-11	BOLD:AAA6231	MF546144	658[0n]	2010	Misiones 2
Aphrissa statira	MACN-Bar-Lep-ct 00568	LEPAR496-11	BOLD:AAA6231	MF545920	658[0n]	2010	Misiones 2
Aphrissa statira	MACN-Bar-Lep-ct 01302	LEPAR884-11	BOLD:AAA6231	MF546456	658[0n]	2010	Misiones 2
Aphrissa statira	MACN-Bar-Lep-ct 02158	LEPIG253-11	BOLD:AAA6231	MF545632	658[0n]	2011	Misiones 3
Aphrissa statira	MACN-Bar-Lep-ct 06040	LEPAR973-14	BOLD:AAA6231	MF545997	658[0n]	2013	Formosa 5
Aphrissa statira	MACN-Bar-Lep-ct 06106	LEPAR1039-14	BOLD:AAA6231	MF547251	658[4n]	2013	Formosa 5
Arawacus ellida	MACN-Bar-Lep-ct 06105	LEPAR1038-14	BOLD:ACO6747	MF546821	658[2n]	2013	Formosa 5
Arawacus melibaeus	MACN-Bar-Lep-ct 00410	LEPAR424-11	BOLD:AAZ6297	MF547237	658[0n]	2010	Misiones 2
Arawacus melibaeus	MACN-Bar-Lep-ct 02167	LEPIG262-11	BOLD:AAZ6297	MF545457	658[0n]	2011	Misiones 3
Arawacus melibaeus	MACN-Bar-Lep-ct 02176	LEPIG270-11	BOLD:AAZ6297	MF545770	658[0n]	2011	Misiones 3
Arawacus melibaeus	MACN-Bar-Lep-ct 02222	LEPIG311-11	BOLD:AAZ6297	MF546003	658[0n]	2011	Misiones 3
Arawacus melibaeus	MACN-Bar-Lep-ct 02383	LEPIG449-11	BOLD:AAZ6297	MF545849	658[0n]	2011	Misiones 3
Arawacus separata	MACN-Bar-Lep-ct 01137	LEPAR771-11	BOLD:AAZ3008	MF546244	658[0n]	2010	Misiones 2
Arawacus separata	MACN-Bar-Lep-ct 02109	LEPIG210-11	BOLD:AAZ3008	MF545402	658[0n]	2011	Misiones 2

Arawacus separata	MACN-Bar-Lep-ct 02150	LEPIG246-11	BOLD:AAZ3008	MF547177	658[0n]	2011	Misiones 3
Arawacus separata	MACN-Bar-Lep-ct 02364	LEPIG435-11	BOLD:AAZ3008	MF545560	658[0n]	2011	Misiones 3
Arawacus separata	MACN-Bar-Lep-ct 02372	LEPIG442-11	BOLD:AAZ3008	MF546455	658[0n]	2011	Misiones 3
Arawacus separata	MACN-Bar-Lep-ct 02387	LEPIG453-11	BOLD:AAZ3008	MF545841	658[0n]	2011	Misiones 3
Arawacus separata	MACN-Bar-Lep-ct 03781	LEPPA836-13	BOLD:AAZ3008	MF547175	658[0n]	2011	Corrientes 1
Archaeoprepona demophon	MACN-Bar-Lep-ct 02128	LEPIG225-11	BOLD:AAA9178	MF545988	658[0n]	2011	Misiones 2
Archaeoprepona demophoon	MACN-Bar-Lep-ct 01159	LEPAR787-11	BOLD:ABZ2182	MF545526	658[0n]	2010	Misiones 2
Archaeoprepona demophoon	MACN-Bar-Lep-ct 02275	LEPIG363-11	BOLD:ABZ2182	MF545934	658[0n]	2011	Misiones 3
Archaeoprepona demophoon	MACN-Bar-Lep-ct 02537	LEPIG557-11	BOLD:ABZ2182	MF546710	658[0n]	2011	Misiones 3
Argon lota	MACN-Bar-Lep-ct 06021	LEPAR954-14	BOLD:AAB8393	MF546908	658[2n]	2013	Formosa 5
Ariconias glaphyra	MACN-Bar-Lep-ct 06090	LEPAR1023-14	BOLD:ACO8338	MF545452	658[2n]	2013	Formosa 5
Ariconias glaphyra	MACN-Bar-Lep-ct 06098	LEPAR1031-14	BOLD:ACO8338	MF546669	658[0n]	2013	Formosa 5
Aricoris chilensis	MACN-Bar-Lep-ct 01591	LEPAR166-11	BOLD:AAZ7975	MF546143	658[0n]	2011	Entre Ríos 3
Aricoris chilensis	MACN-Bar-Lep-ct 01596	LEPAR168-11	BOLD:AAZ7975	MF546486	658[0n]	2011	Entre Ríos 3
Aricoris chilensis	MACN-Bar-Lep-ct 01599	LEPAR170-11	BOLD:AAZ7975	MF546117	658[0n]	2011	Entre Ríos 3
Aricoris chilensis	MACN-Bar-Lep-ct 01861	LEPAR279-11	BOLD:AAZ7975	MF546697	658[0n]	2011	Entre Ríos 3
Aricoris chilensis	MACN-Bar-Lep-ct 03898	LEPPA532-13	BOLD:AAZ7975	MF547368	618[0n]	2012	Formosa 4
Aricoris chilensis	MACN-Bar-Lep-ct 06045	LEPAR978-14	BOLD:AAZ7975	MF546950	658[1n]	2013	Formosa 5
Aricoris chilensis	MACN-Bar-Lep-ct 06050	LEPAR983-14	BOLD:AAZ7975	MF547317	658[4n]	2013	Formosa 5
Aricoris chilensis	MACN-Bar-Lep-ct 06089	LEPAR1022-14	BOLD:AAZ7975	MF547382	658[0n]	2013	Formosa 5
Aricoris indistincta	MACN-Bar-Lep-ct 01485	LEPAR120-11	BOLD:AAZ9326	MF546631	658[0n]	2011	Entre Ríos 3
Aricoris indistincta	MACN-Bar-Lep-ct 01866	LEPAR282-11	BOLD:AAZ9326	MF546184	658[0n]	2011	Entre Ríos 3
Aricoris notialis	MACN-Bar-Lep-ct 01646	LEPAR192-11	BOLD:AAZ9715	MF546819	658[0n]	2011	Entre Ríos 4
Aricoris signata	MACN-Bar-Lep-ct 00534	LEPAR470-11	BOLD:ABZ8182	MF546401	658[0n]	2010	Misiones 2
Aricoris signata	MACN-Bar-Lep-ct 00535	LEPAR471-11	BOLD:ABZ8182	MF545713	658[0n]	2010	Misiones 2
Aricoris signata	MACN-Bar-Lep-ct 03127	LEPPA049-11	BOLD:ABZ8182	MF546008	658[0n]	2011	Buenos Aires 21
Aricoris signata	MACN-Bar-Lep-ct 03140	LEPPA060-11	BOLD:ABZ8182	MF546227	658[0n]	2011	Buenos Aires 21
Aricoris signata	MACN-Bar-Lep-ct 03161	LEPPA075-11	BOLD:ABZ8182	MF547001	658[0n]	2011	Buenos Aires 21
Aricoris signata	MACN-Bar-Lep-ct 03166	LEPPA077-11	BOLD:ABZ8182	MF547008	658[0n]	2011	Buenos Aires 21
Ascia monuste	MACN-Bar-Lep-ct 00059	LEPAR039-11	BOLD:AAB6892	MF545675	658[0n]	2010	Formosa 1
Ascia monuste	MACN-Bar-Lep-ct 00060	LEPAR040-11	BOLD:AAB6892	MF547179	658[0n]	2010	Formosa 1
Ascia monuste	MACN-Bar-Lep-ct 00061	LEPAR041-11	BOLD:AAB6892	MF546120	658[0n]	2010	Formosa 1
Ascia monuste	MACN-Bar-Lep-ct 00087	LEPAR065-11			0	2006	Córdoba 1

Ascia monuste	MACN-Bar-Lep-ct 02367	LEPIG438-11	BOLD:AAB6892	MF547253	658[0n]	2011	Misiones 3
Ascia monuste	MACN-Bar-Lep-ct 02644	LEPPA297-12	BOLD:AAB6892	MF546636	658[0n]	2011	Formosa 2
Ascia monuste	MACN-Bar-Lep-ct 02657	LEPPA310-12	BOLD:AAB6892	MF546352	658[0n]	2011	Formosa 2
Ascia monuste	MACN-Bar-Lep-ct 02667	LEPPA320-12	BOLD:AAB6892	MF545806	658[0n]	2011	Formosa 2
Ascia monuste	MACN-Bar-Lep-ct 06032	LEPAR965-14	BOLD:AAB6892	MF546845	658[4n]	2013	Formosa 5
Ascia monuste	MACN-Bar-Lep-ct 06041	LEPAR974-14	BOLD:AAB6892	MF545587	626[0n]	2013	Formosa 5
Ascia monuste	MACN-Bar-Lep-ct 06068	LEPAR1001-14	BOLD:AAB6892	MF547129	658[0n]	2013	Formosa 5
Ascia monuste	MACN-Bar-Lep-ct 06075	LEPAR1008-14	BOLD:AAB6892	MF546747	658[1n]	2013	Formosa 5
Ascia monuste	MACN-Bar-Lep-ct 06084	LEPAR1017-14	BOLD:AAB6892	MF546822	658[0n]	2013	Formosa 5
Ascia monuste	MACN-Bar-Lep-ct 06464	LEPPA1052-14			0	2013	Córdoba 4
Ascia monuste	MACN-Bar-Lep-ct 06477	LEPPA1065-14			0	2013	Córdoba 4
Astraptes anaphus	MACN-Bar-Lep-ct 02406	LEPIG471-11	BOLD:AAA6379	MF546038	658[0n]	2011	Misiones 3
Astraptes anaphus	MACN-Bar-Lep-ct 02419	LEPIG482-11	BOLD:AAA6379	MF546284	658[0n]	2011	Misiones 3
Astraptes anaphus	MACN-Bar-Lep-ct 02448	LEPIG502-11	BOLD:AAA6379	MF547276	658[0n]	2011	Misiones 3
Astraptes enotrus	MACN-Bar-Lep-ct 01346	LEPAR927-11	BOLD:AAZ3733	MF545817	658[0n]	2010	Misiones 2
Astraptes fulgerator	MACN-Bar-Lep-ct 00787	LEPAR569-11	BOLD:ACF3165	MF545521	658[0n]	2010	Misiones 2
Astraptes fulgerator	MACN-Bar-Lep-ct 01311	LEPAR893-11	BOLD:ACF3165	MF547203	658[0n]	2010	Misiones 2
Astraptes fulgerator	MACN-Bar-Lep-ct 02211	LEPIG300-11	BOLD:AAA5249	MF547060	658[0n]	2011	Misiones 3
Barbicornis basilis	MACN-Bar-Lep-ct 00404	LEPAR420-11	BOLD:AAZ0958	MF546752	658[0n]	2010	Misiones 2
Barbicornis basilis	MACN-Bar-Lep-ct 00862	LEPAR631-11	BOLD:AAZ0958	MF547052	658[0n]	2010	Misiones 2
Barbicornis basilis	MACN-Bar-Lep-ct 01032	LEPAR728-11	BOLD:AAZ0958	MF545631	603[0n]	2010	Misiones 2
Barbicornis basilis	MACN-Bar-Lep-ct 01033	LEPAR729-11	BOLD:AAZ0958	MF545492	658[0n]	2010	Misiones 2
Barbicornis basilis	MACN-Bar-Lep-ct 01144	LEPAR777-11	BOLD:AAZ0958	MF545605	658[0n]	2010	Misiones 2
Barbicornis basilis	MACN-Bar-Lep-ct 01963	LEPIG084-11	BOLD:AAZ0958	MF547173	658[0n]	2011	Misiones 2
Barbicornis basilis	MACN-Bar-Lep-ct 02346	LEPIG422-11	BOLD:AAZ0958	MF546927	658[0n]	2011	Misiones 3
Barbicornis basilis	MACN-Bar-Lep-ct 02363	LEPIG434-11	BOLD:AAZ0958	MF546566	658[0n]	2011	Misiones 3
Battus polydamas	MACN-Bar-Lep-ct 00240	LEPAR326-11	BOLD:AAB6663	MF547140	658[0n]	2010	Misiones 2
Battus polydamas	MACN-Bar-Lep-ct 00286	LEPAR350-11	BOLD:AAB6663	MF547062	658[0n]	2010	Misiones 2
Battus polydamas	MACN-Bar-Lep-ct 01002	LEPAR702-11	BOLD:AAB6663	MF546308	658[0n]	2010	Misiones 2
Battus polydamas	MACN-Bar-Lep-ct 02490	LEPIG526-11	BOLD:AAB6663	MF545972	658[0n]	2011	Misiones 3
Battus polydamas	MACN-Bar-Lep-ct 03700	LEPPA786-13	BOLD:AAB6663	MF545638	658[0n]	2011	Corrientes 1
Biblis hyperia	MACN-Bar-Lep-ct 00799	LEPAR579-11	BOLD:ABY4877	MF546511	658[0n]	2010	Misiones 2
Biblis hyperia	MACN-Bar-Lep-ct 00831	LEPAR606-11	BOLD:ABY4877	MF545807	658[0n]	2010	Misiones 2

Biblis hyperia	MACN-Bar-Lep-ct 00833	LEPAR608-11	BOLD:ABY4877	MF547102	658[0n]	2010	Misiones 2
Biblis hyperia	MACN-Bar-Lep-ct 01026	LEPAR723-11	BOLD:ABY4877	MF547135	658[0n]	2010	Misiones 2
Biblis hyperia	MACN-Bar-Lep-ct 01147	LEPAR779-11	BOLD:ABY4877	MF545834	658[0n]	2010	Misiones 2
Biblis hyperia	MACN-Bar-Lep-ct 01483	LEPAR119-11	BOLD:ABY4877	MF545479	658[0n]	2011	Entre Ríos 3
Biblis hyperia	MACN-Bar-Lep-ct 01893	LEPIG021-11	BOLD:ABY4877	MF545510	658[0n]	2011	Misiones 2
Biblis hyperia	MACN-Bar-Lep-ct 02164	LEPIG259-11	BOLD:ABY4877	MF546498	658[0n]	2011	Misiones 3
Biblis hyperia	MACN-Bar-Lep-ct 02209	LEPIG298-11	BOLD:ABY4877	MF547355	658[0n]	2011	Misiones 3
Bolla atahuallpai	MACN-Bar-Lep-ct 00368	LEPAR402-11	*Wolbachia*		658[0n]	2010	Misiones 2
Bolla atahuallpai	MACN-Bar-Lep-ct 02033	LEPIG143-11	*Wolbachia*		658[0n]	2011	Misiones 2
Bolla atahuallpai	MACN-Bar-Lep-ct 02435	LEPIG494-11	*Wolbachia*		658[0n]	2011	Misiones 3
Brangas getus	MACN-Bar-Lep-ct 02824	LEPPA416-12	BOLD:ACK2713	MF546405	658[0n]	2011	Formosa 2
Caeruleuptychia helena	MACN-Bar-Lep-ct 00436	LEPAR448-11	BOLD:AAJ8621	MF546867	658[0n]	2010	Misiones 2
Caeruleuptychia helena	MACN-Bar-Lep-ct 02214	LEPIG303-11	BOLD:AAZ9284	MF546098	658[0n]	2011	Misiones 3
Calephelis aymaran	MACN-Bar-Lep-ct 02629	LEPPA283-12	BOLD:ACA8905	MF545926	658[0n]	2011	Formosa 2
Caligo illioneus	MACN-Bar-Lep-ct 02571	LEPPA226-12	BOLD:ACA8924	MF547002	658[0n]	2011	Formosa 2
Callicore hydaspes	MACN-Bar-Lep-ct 00069	LEPAR049-11	BOLD:AAF5473	MF546982	658[0n]		Misiones 16
Callicore hydaspes	MACN-Bar-Lep-ct 00159	LEPAR288-11	BOLD:AAF5473	MF546560	658[0n]	2010	Misiones 2
Callicore hydaspes	MACN-Bar-Lep-ct 00242	LEPAR328-11	BOLD:AAF5473	MF546026	658[0n]	2010	Misiones 2
Callicore hydaspes	MACN-Bar-Lep-ct 00345	LEPAR386-11	BOLD:AAF5473	MF546113	649[0n]	2010	Misiones 2
Callicore hydaspes	MACN-Bar-Lep-ct 00355	LEPAR392-11	BOLD:AAF5473	MF547099	658[0n]	2010	Misiones 2
Callicore hydaspes	MACN-Bar-Lep-ct 01192	LEPAR814-11	BOLD:AAF5473	MF545536	658[0n]	2010	Misiones 2
Callicore hydaspes	MACN-Bar-Lep-ct 02119	LEPIG216-11	BOLD:AAF5473	MF546251	649[0n]	2011	Misiones 2
Callimormus interpunctata	MACN-Bar-Lep-ct 02072	LEPIG176-11	BOLD:AAZ9654	MF546435	658[0n]	2011	Misiones 2
Callimormus simplicius	MACN-Bar-Lep-ct 02735	LEPPA369-12	BOLD:ACA8891	MF545975	658[0n]	2011	Formosa 3
Callimormus simplicius	MACN-Bar-Lep-ct 02839	LEPPA423-12	BOLD:ACA8891	MF545421	658[0n]	2011	Formosa 2
Calpodes ethlius	MACN-Bar-Lep-ct 03878	LEPPA520-13	BOLD:AAA9388	MF546417	658[0n]	2012	Formosa 4
Calpodes ethlius	MACN-Bar-Lep-ct 03933	LEPPA559-13	BOLD:AAA9388	MF547386	622[0n]	2012	Formosa 4
Calycopis caulonia	MACN-Bar-Lep-ct 01665	LEPAR201-11	BOLD:AAZ1864	MF545902	658[0n]	2011	Entre Ríos 4
Calycopis caulonia	MACN-Bar-Lep-ct 01710	LEPAR218-11	BOLD:AAZ8148	MF546043	658[0n]	2011	Entre Ríos 4
Calycopis caulonia	MACN-Bar-Lep-ct 01716	LEPAR221-11	BOLD:AAZ8148	MF547123	658[0n]	2011	Entre Ríos 4
Calycopis caulonia	MACN-Bar-Lep-ct 02656	LEPPA309-12	BOLD:AAZ1864	MF547364	658[0n]	2011	Formosa 2
Calycopis caulonia	MACN-Bar-Lep-ct 02844	LEPPA425-12	BOLD:AAZ1864	MF545779	658[0n]	2011	Formosa 2
Calycopis caulonia	MACN-Bar-Lep-ct 03706	LEPPA790-13	BOLD:AAZ1864	MF546986	658[0n]	2011	Corrientes 1

Calycopis caulonia	MACN-Bar-Lep-ct 03837	LEPPA867-13	BOLD:AAZ1864	MF546381	658[0n]	2011	Corrientes 1
Calycopis caulonia	MACN-Bar-Lep-ct 03918	LEPPA546-13			0	2012	Formosa 4
Calycopis sp. 1	MACN-Bar-Lep-ct 02700	LEPPA345-12	BOLD:ACA8832	MF547359	658[0n]	2011	Formosa 2
Calycopis sp. 2	MACN-Bar-Lep-ct 00417	LEPAR429-11	BOLD:AAZ1865	MF545498	658[0n]	2010	Misiones 2
Calycopis sp. 2	MACN-Bar-Lep-ct 01133	LEPAR767-11	BOLD:AAK1561	MF547057	658[0n]	2010	Misiones 2
Calycopis sp. 2	MACN-Bar-Lep-ct 01190	LEPAR812-11	BOLD:AAZ1864	MF547225	658[0n]	2010	Misiones 2
Calycopis sp. 2	MACN-Bar-Lep-ct 01221	LEPAR836-11	BOLD:AAZ1865	MF546261	658[0n]	2010	Misiones 2
Calycopis sp. 2	MACN-Bar-Lep-ct 01326	LEPAR907-11	BOLD:AAZ1864	MF546466	658[0n]	2010	Misiones 2
Camptopleura auxo	MACN-Bar-Lep-ct 00873	LEPAR640-11	BOLD:AAZ5046	MF546250	658[0n]	2010	Misiones 2
Caria marsyas	MACN-Bar-Lep-ct 02693	LEPPA342-12	BOLD:ACA8748	MF546988	658[0n]	2011	Formosa 2
Caria marsyas	MACN-Bar-Lep-ct 03671	LEPPA764-13	BOLD:ACA8748	MF545652	658[0n]	2011	Corrientes 1
Caria marsyas	MACN-Bar-Lep-ct 03769	LEPPA830-13	BOLD:ACA8748	MF546362	618[0n]	2011	Corrientes 1
Caria marsyas	MACN-Bar-Lep-ct 03823	LEPPA859-13	BOLD:ACA8748	MF546987	658[0n]	2011	Corrientes 1
Caria marsyas	MACN-Bar-Lep-ct 03940	LEPPA565-13	BOLD:ACA8748	MF546592	658[0n]	2012	Formosa 4
Caria plutargus	MACN-Bar-Lep-ct 02217	LEPIG306-11	BOLD:AAZ7598	MF545530	658[0n]	2011	Misiones 3
Caria plutargus	MACN-Bar-Lep-ct 02355	LEPIG429-11	BOLD:AAZ7598	MF546460	658[0n]	2011	Misiones 3
Caria plutargus	MACN-Bar-Lep-ct 02545	LEPIG563-11	BOLD:AAZ7598	MF545513	658[0n]	2011	Misiones 3
Caria plutargus	MACN-Bar-Lep-ct 02550	LEPIG568-11	BOLD:AAZ7598	MF546946	658[0n]	2011	Misiones 3
Caria plutargus	MACN-Bar-Lep-ct 02557	LEPIG574-11	BOLD:AAZ7598	MF546089	658[0n]	2011	Misiones 3
Caria plutargus	MACN-Bar-Lep-ct 02561	LEPIG577-11	BOLD:AAZ7598	MF547305	658[0n]	2011	Misiones 3
Caria plutargus	MACN-Bar-Lep-ct 02564	LEPIG579-11	BOLD:AAZ7598	MF546468	658[0n]	2011	Misiones 3
Carrhenes canescens	MACN-Bar-Lep-ct 00420	LEPAR432-11	BOLD:AAB0383	MF545473	658[0n]	2010	Misiones 2
Carrhenes canescens	MACN-Bar-Lep-ct 01968	LEPIG089-11	BOLD:AAB0383	MF545921	658[0n]	2011	Misiones 2
Carrhenes canescens	MACN-Bar-Lep-ct 02280	LEPIG367-11	BOLD:AAB0383	MF546687	658[0n]	2011	Misiones 3
Catoblepia amphirhoe	MACN-Bar-Lep-ct 00813	LEPAR590-11	BOLD:AAZ1358	MF546132	658[0n]	2010	Misiones 2
Catoblepia amphirhoe	MACN-Bar-Lep-ct 00836	LEPAR611-11	BOLD:AAZ1358	MF546797	658[0n]	2010	Misiones 2
Catoblepia amphirhoe	MACN-Bar-Lep-ct 01007	LEPAR707-11	BOLD:AAZ1358	MF546750	658[0n]	2010	Misiones 2
Catoblepia amphirhoe	MACN-Bar-Lep-ct 01177	LEPAR801-11	BOLD:AAZ1358	MF546504	658[0n]	2010	Misiones 2
Catoblepia amphirhoe	MACN-Bar-Lep-ct 01178	LEPAR802-11	BOLD:AAZ1358	MF546831	658[0n]	2010	Misiones 2
Catonephele numilia	MACN-Bar-Lep-ct 02465	LEPIG509-11	BOLD:AAA8583	MF545766	658[0n]	2011	Misiones 3
Catonephele numilia	MACN-Bar-Lep-ct 02476	LEPIG516-11	BOLD:AAA8583	MF545906	658[0n]	2011	Misiones 3
Catonephele numilia	MACN-Bar-Lep-ct 02484	LEPIG522-11	BOLD:AAA8583	MF547127	658[0n]	2011	Misiones 3
Celmia celmus	MACN-Bar-Lep-ct 01331	LEPAR912-11	BOLD:ABZ0253	MF546956	658[0n]	2010	Misiones 2

Celmia celmus	MACN-Bar-Lep-ct 01332	LEPAR913-11	BOLD:ABZ0253	MF546717	658[0n]	2010	Misiones 2
Celmia celmus	MACN-Bar-Lep-ct 01333	LEPAR914-11	BOLD:ABZ0253	MF547010	658[0n]	2010	Misiones 2
Chalodeta theodora	MACN-Bar-Lep-ct 01309	LEPAR891-11	BOLD:AAZ5052	MF546474	658[0n]	2010	Misiones 2
Chamaelimnas briola	MACN-Bar-Lep-ct 01031	LEPAR727-11	BOLD:AAZ1455	MF546826	658[0n]	2010	Misiones 2
Chamaelimnas briola	MACN-Bar-Lep-ct 02295	LEPIG380-11	BOLD:AAZ1455	MF545977	658[0n]	2011	Misiones 3
Chamaelimnas briola	MACN-Bar-Lep-ct 02304	LEPIG388-11	BOLD:AAZ1455	MF545666	658[0n]	2011	Misiones 3
Chioides catillus	MACN-Bar-Lep-ct 01508	LEPAR135-11	BOLD:AAA9620	MF546999	658[0n]	2011	Entre Ríos 4
Chioides catillus	MACN-Bar-Lep-ct 01697	LEPAR214-11	BOLD:AAA9620	MF546219	658[0n]	2011	Entre Ríos 4
Chioides catillus	MACN-Bar-Lep-ct 02802	LEPPA410-12	BOLD:AAA9620	MF546813	658[0n]	2011	Formosa 2
Chioides catillus	MACN-Bar-Lep-ct 03596	LEPPA721-13	BOLD:AAA9620	MF546110	658[0n]	2011	Corrientes 1
Chioides catillus	MACN-Bar-Lep-ct 03627	LEPPA738-13	BOLD:AAA9620	MF545643	658[0n]	2011	Corrientes 1
Chioides catillus	MACN-Bar-Lep-ct 03661	LEPPA760-13	BOLD:AAA9620	MF546510	658[0n]	2011	Corrientes 1
Chioides catillus	MACN-Bar-Lep-ct 03925	LEPPA552-13	BOLD:AAA9620	MF546952	651[0n]	2012	Formosa 4
Chioides catillus	MACN-Bar-Lep-ct 06059	LEPAR992-14	BOLD:AAA9620	MF547373	658[2n]	2013	Formosa 5
Chioides catillus	MACN-Bar-Lep-ct 06101	LEPAR1034-14	BOLD:AAA9620	MF546899	658[2n]	2013	Formosa 5
Chiomara asychis	MACN-Bar-Lep-ct 02647	LEPPA300-12	BOLD:ACO4065	MF545824	658[0n]	2011	Formosa 2
Chiomara asychis	MACN-Bar-Lep-ct 02855	LEPPA431-12	BOLD:ACO4065	MF545715	658[0n]	2011	Formosa 2
Chiomara asychis	MACN-Bar-Lep-ct 03923	LEPPA551-13	BOLD:ACO4065	MF546192	658[0n]	2012	Formosa 4
Chiomara asychis	MACN-Bar-Lep-ct 06043	LEPAR976-14	BOLD:ACO4065	MF546870	658[0n]	2013	Formosa 5
Chiomara asychis	MACN-Bar-Lep-ct 06069	LEPAR1002-14	BOLD:ACO4065	MF546698	658[2n]	2013	Formosa 5
Chiomara mithrax	MACN-Bar-Lep-ct 01939	LEPIG062-11	BOLD:AAB2608	MF545653	658[0n]	2011	Misiones 2
Chlorostrymon simaethis	MACN-Bar-Lep-ct 06065	LEPAR998-14	BOLD:AAF3214	MF545783	658[1n]	2013	Formosa 5
Chlosyne lacinia	MACN-Bar-Lep-ct 00531	LEPAR468-11	BOLD:AAB0381	MF545601	658[0n]	2010	Misiones 2
Chlosyne lacinia	MACN-Bar-Lep-ct 01898	LEPIG026-11	BOLD:AAB0381	MF546481	658[0n]	2011	Misiones 2
Chlosyne lacinia	MACN-Bar-Lep-ct 01966	LEPIG087-11	BOLD:AAB0381	MF545788	658[0n]	2011	Misiones 2
Chlosyne lacinia	MACN-Bar-Lep-ct 01983	LEPIG102-11	BOLD:AAB0381	MF545698	658[0n]	2011	Misiones 2
Chlosyne lacinia	MACN-Bar-Lep-ct 01988	LEPIG107-11	BOLD:AAB0381	MF546485	658[0n]	2011	Misiones 2
Chlosyne lacinia	MACN-Bar-Lep-ct 02463	LEPIG507-11	BOLD:AAB0381	MF545396	658[0n]	2011	Misiones 3
Chlosyne lacinia	MACN-Bar-Lep-ct 02483	LEPIG521-11	BOLD:AAB0381	MF545728	658[0n]	2011	Misiones 3
Chlosyne lacinia	MACN-Bar-Lep-ct 02672	LEPPA324-12	BOLD:AAB0381	MF546011	658[0n]	2011	Formosa 2
Chlosyne lacinia	MACN-Bar-Lep-ct 02704	LEPPA347-12	BOLD:AAB0381	MF546293	658[0n]	2011	Formosa 3
Chlosyne lacinia	MACN-Bar-Lep-ct 02715	LEPPA357-12	BOLD:AAB0381	MF546803	658[0n]	2011	Formosa 3
Cobalopsis catocala	MACN-Bar-Lep-ct 02748	LEPPA379-12	BOLD:ACA8709	MF545624	658[0n]	2011	Formosa 3

Cobalopsis catocala	MACN-Bar-Lep-ct 02852	LEPPA429-12	BOLD:ACA8709	MF546878	658[0n]	2011	Formosa 2
Cobalopsis catocala	MACN-Bar-Lep-ct 02860	LEPPA434-12	BOLD:ACA8709	MF545743	658[0n]	2011	Formosa 2
Cobalopsis miaba	MACN-Bar-Lep-ct 02088	LEPIG191-11	BOLD:AAE3807	MF546334	658[0n]	2011	Misiones 2
Cobalopsis miaba	MACN-Bar-Lep-ct 02134	LEPIG231-11	BOLD:AAE3807	MF546726	658[0n]	2011	Misiones 2
Cobalopsis nero	MACN-Bar-Lep-ct 02066	LEPIG172-11	BOLD:AAA4134	MF545732	658[0n]	2011	Misiones 2
Cobalopsis nero	MACN-Bar-Lep-ct 02097	LEPIG200-11	BOLD:AAA4134	MF547186	658[0n]	2011	Misiones 2
Cobalopsis nero	MACN-Bar-Lep-ct 02567	LEPIG582-11	BOLD:AAA4134	MF546254	658[0n]	2011	Misiones 3
Cobalopsis sp. 1	MACN-Bar-Lep-ct 06071	LEPAR1004-14	BOLD:ACP1278	MF546676	658[4n]	2013	Formosa 5
Cobalopsis sp. 1	MACN-Bar-Lep-ct 06086	LEPAR1019-14	BOLD:ACP1278	MF547124	658[0n]	2013	Formosa 5
Codatractus aminias	MACN-Bar-Lep-ct 00171	LEPAR295-11	BOLD:AAZ3952	MF546842	658[0n]	2010	Misiones 2
Codatractus aminias	MACN-Bar-Lep-ct 00238	LEPAR324-11	BOLD:AAZ3952	MF546966	658[0n]	2010	Misiones 2
Codatractus aminias	MACN-Bar-Lep-ct 00308	LEPAR364-11	BOLD:AAZ3952	MF547198	658[0n]	2010	Misiones 2
Codatractus aminias	MACN-Bar-Lep-ct 00331	LEPAR378-11	BOLD:AAZ3952	MF546532	658[0n]	2010	Misiones 2
Codatractus aminias	MACN-Bar-Lep-ct 00347	LEPAR388-11	BOLD:AAZ3952	MF545435	658[0n]	2010	Misiones 2
Cogia abdul	MACN-Bar-Lep-ct 01505	LEPAR133-11	BOLD:AAZ9522	MF546346	658[0n]	2011	Entre Ríos 4
Colias lesbia	MACN-Bar-Lep-ct 00028	LEPAR017-11	BOLD:AAY7698	MF547267	658[0n]	2010	Buenos Aires 21
Colias lesbia	MACN-Bar-Lep-ct 00029	LEPAR018-11	BOLD:AAY7698	MF546154	658[0n]	2010	Buenos Aires 21
Colias lesbia	MACN-Bar-Lep-ct 00133	LEPPA108-11	BOLD:AAY7698	MF545439	658[0n]	2010	Buenos Aires 21
Colias lesbia	MACN-Bar-Lep-ct 01768	LEPAR244-11	BOLD:AAY7698	MF547215	658[0n]	2011	Entre Ríos 6
Colias lesbia	MACN-Bar-Lep-ct 03059	LEPPA172-11	BOLD:AAY7698	MF545670	658[0n]	2011	Buenos Aires 4
Colias lesbia	MACN-Bar-Lep-ct 03105	LEPPA028-11			0	2011	Buenos Aires 21
Colias lesbia	MACN-Bar-Lep-ct 03118	LEPPA041-11			0	2011	Buenos Aires 21
Colias lesbia	MACN-Bar-Lep-ct 06063	LEPAR996-14	BOLD:AAY7698	MF546263	658[0n]	2013	Formosa 5
Colias lesbia	MACN-Bar-Lep-ct 06474	LEPPA1062-14			0	2013	Córdoba 4
Colobura dirce	MACN-Bar-Lep-ct 02121	LEPIG218-11	BOLD:ABZ2587	MF545863	658[0n]	2011	Misiones 2
Colobura dirce	MACN-Bar-Lep-ct 02140	LEPIG237-11	BOLD:ABZ2587	MF546737	658[0n]	2011	Misiones 3
Colobura dirce	MACN-Bar-Lep-ct 02207	LEPIG297-11	BOLD:ABZ2587	MF546545	658[0n]	2011	Misiones 3
Colobura dirce	MACN-Bar-Lep-ct 02213	LEPIG302-11	BOLD:ABZ2587	MF546883	658[0n]	2011	Misiones 3
Colobura dirce	MACN-Bar-Lep-ct 02307	LEPIG391-11	BOLD:ABZ2587	MF545400	658[0n]	2011	Misiones 3
Conga chydaea	MACN-Bar-Lep-ct 06039	LEPAR972-14	BOLD:AAA6384	MF545404	658[0n]	2013	Formosa 5
Conga iheringii	MACN-Bar-Lep-ct 00136	LEPPA111-11	BOLD:ACG2329	MF545541	658[0n]	2010	Buenos Aires 21
Conga iheringii	MACN-Bar-Lep-ct 00137	LEPPA112-11	BOLD:ACG2329	MF546969	658[0n]	2010	Buenos Aires 21
Conga iheringii	MACN-Bar-Lep-ct 00138	LEPPA113-11	BOLD:ACG2329	MF546347	658[0n]	2010	Buenos Aires 21

Conga iheringii	MACN-Bar-Lep-ct 00139	LEPPA114-11	BOLD:ACG2329	MF547231	658[0n]	2010	Buenos Aires 21
Conga iheringii	MACN-Bar-Lep-ct 00140	LEPPA115-11	BOLD:ACG2329	MF546984	658[0n]	2010	Buenos Aires 21
Consul fabius	MACN-Bar-Lep-ct 02163	LEPIG258-11	BOLD:AAB4276	MF546589	658[0n]	2011	Misiones 3
Consul fabius	MACN-Bar-Lep-ct 02389	LEPIG455-11	BOLD:AAB4276	MF546015	644[0n]	2011	Misiones 3
Consul fabius	MACN-Bar-Lep-ct 02503	LEPIG534-11	BOLD:AAB4276	MF546020	658[0n]	2011	Misiones 3
Consul fabius	MACN-Bar-Lep-ct 02525	LEPIG547-11	BOLD:AAB4276	MF546447	658[0n]	2011	Misiones 3
Contrafacia imma	MACN-Bar-Lep-ct 00433	LEPAR445-11	BOLD:AAZ6124	MF545556	658[0n]	2010	Misiones 2
Corticea corticea	MACN-Bar-Lep-ct 01946	LEPIG068-11	BOLD:AAA8791	MF546390	658[0n]	2011	Misiones 2
Corticea corticea	MACN-Bar-Lep-ct 02007	LEPIG124-11	BOLD:AAA8791	MF546138	658[0n]	2011	Misiones 2
Corticea corticea	MACN-Bar-Lep-ct 02247	LEPIG335-11	BOLD:AAA8791	MF545444	658[0n]	2011	Misiones 3
Corticea lysias	MACN-Bar-Lep-ct 00190	LEPAR308-11	BOLD:AAZ3916	MF546536	658[0n]	2010	Misiones 2
Corticea lysias	MACN-Bar-Lep-ct 00877	LEPAR644-11	BOLD:AAZ3916	MF546667	658[0n]	2010	Misiones 2
Corticea lysias	MACN-Bar-Lep-ct 00880	LEPAR647-11	BOLD:AAZ3916	MF545838	658[0n]	2010	Misiones 2
Corticea lysias	MACN-Bar-Lep-ct 00881	LEPAR648-11	BOLD:AAZ3916	MF546840	658[0n]	2010	Misiones 2
Corticea lysias	MACN-Bar-Lep-ct 02133	LEPIG230-11	BOLD:AAZ3916	MF546941	658[0n]	2011	Misiones 2
Corticea lysias	MACN-Bar-Lep-ct 02258	LEPIG346-11	BOLD:AAZ3916	MF545514	658[0n]	2011	Misiones 3
Corticea sp.	MACN-Bar-Lep-ct 00192	LEPAR310-11	BOLD:AAZ3760	MF545584	658[0n]	2010	Misiones 2
Corticea sp.	MACN-Bar-Lep-ct 02273	LEPIG361-11	BOLD:AAZ3760	MF547405	658[0n]	2011	Misiones 3
Cybdelis phaesyla	MACN-Bar-Lep-ct 01353	LEPAR934-11	BOLD:AAD3220	MF546073	658[0n]	2010	Misiones 2
Cycloglypha caeruleonigra	MACN-Bar-Lep-ct 00860	LEPAR629-11	BOLD:ABZ1454	MF546991	658[0n]	2010	Misiones 2
Cycloglypha caeruleonigra	MACN-Bar-Lep-ct 01021	LEPAR718-11	BOLD:ABZ1454	MF545778	658[0n]	2010	Misiones 2
Cymaenes alumna	MACN-Bar-Lep-ct 02686	LEPPA336-12	BOLD:ACA8655	MF545406	658[0n]	2011	Formosa 2
Cymaenes cavalla	MACN-Bar-Lep-ct 02271	LEPIG359-11	BOLD:AAZ7927	MF545886	658[0n]	2011	Misiones 3
Cymaenes cavalla	MACN-Bar-Lep-ct 02349	LEPIG425-11	BOLD:AAZ7927	MF546487	650[0n]	2011	Misiones 3
Cymaenes cavalla	MACN-Bar-Lep-ct 02356	LEPIG430-11	BOLD:AAZ7927	MF547290	658[0n]	2011	Misiones 3
Cymaenes gisca	MACN-Bar-Lep-ct 00172	LEPAR296-11	BOLD:AAZ4980	MF546349	658[0n]	2010	Misiones 2
Cymaenes gisca	MACN-Bar-Lep-ct 00425	LEPAR437-11	BOLD:AAZ4980	MF546783	658[0n]	2010	Misiones 2
Cymaenes gisca	MACN-Bar-Lep-ct 00426	LEPAR438-11	BOLD:AAZ4980	MF546933	658[0n]	2010	Misiones 2
Cymaenes gisca	MACN-Bar-Lep-ct 00427	LEPAR439-11	BOLD:AAZ4980	MF546614	658[0n]	2010	Misiones 2
Cymaenes gisca	MACN-Bar-Lep-ct 01952	LEPIG074-11	BOLD:AAZ4980	MF545547	658[0n]	2011	Misiones 2
Cymaenes gisca	MACN-Bar-Lep-ct 02018	LEPIG131-11	BOLD:AAZ4980	MF546496	658[0n]	2011	Misiones 2
Cymaenes gisca	MACN-Bar-Lep-ct 02026	LEPIG138-11	BOLD:AAZ4980	MF545607	658[0n]	2011	Misiones 2
Cymaenes gisca	MACN-Bar-Lep-ct 02035	LEPIG145-11	BOLD:AAZ4980	MF546075	658[0n]	2011	Misiones 2

Cymaenes gisca	MACN-Bar-Lep-ct 02256	LEPIG344-11	BOLD:AAZ4980	MF545878	658[0n]	2011	Misiones 3
Cymaenes gisca	MACN-Bar-Lep-ct 02267	LEPIG355-11	BOLD:AAZ4980	MF545748	658[0n]	2011	Misiones 3
Cymaenes gisca	MACN-Bar-Lep-ct 02270	LEPIG358-11	BOLD:AAZ4980	MF545811	658[0n]	2011	Misiones 3
Cymaenes gisca	MACN-Bar-Lep-ct 06587	LEPPA1175-14	COI < 500 bp		280[2n]	2012	Buenos Aires 3
Cymaenes laureolus	MACN-Bar-Lep-ct 00185	LEPAR303-11	BOLD:AAZ4883	MF545540	658[0n]	2010	Misiones 2
Cymaenes laureolus	MACN-Bar-Lep-ct 00786	LEPAR568-11	BOLD:AAZ4883	MF545472	658[0n]	2010	Misiones 2
Cymaenes laureolus	MACN-Bar-Lep-ct 02020	LEPIG133-11	BOLD:AAZ4883	MF545883	658[0n]	2011	Misiones 2
Cymaenes laureolus	MACN-Bar-Lep-ct 02032	LEPIG142-11	BOLD:AAZ4883	MF545871	658[0n]	2011	Misiones 2
Cymaenes laureolus	MACN-Bar-Lep-ct 02073	LEPIG177-11	BOLD:AAZ4883	MF545892	658[0n]	2011	Misiones 2
Cymaenes lepta	MACN-Bar-Lep-ct 02025	LEPIG137-11	BOLD:ACE6172	MF546954	658[0n]	2011	Misiones 2
Cymaenes lepta	MACN-Bar-Lep-ct 02060	LEPIG167-11	BOLD:ACE6172	MF545928	658[0n]	2011	Misiones 2
Cymaenes lepta	MACN-Bar-Lep-ct 02067	LEPIG173-11	BOLD:ACE6172	MF546633	658[0n]	2011	Misiones 2
Cymaenes lepta	MACN-Bar-Lep-ct 02075	LEPIG179-11	BOLD:ACE6172	MF546740	658[0n]	2011	Misiones 2
Cymaenes lepta	MACN-Bar-Lep-ct 02077	LEPIG181-11	BOLD:ACE6172	MF546527	658[0n]	2011	Misiones 2
Cymaenes lepta	MACN-Bar-Lep-ct 03851	LEPPA877-13			0	2011	Corrientes 1
Cymaenes odilia	MACN-Bar-Lep-ct 00135	LEPPA110-11	BOLD:AAZ9597	MF545429	658[0n]	2010	Buenos Aires 21
Cymaenes odilia	MACN-Bar-Lep-ct 01452	LEPAR100-11	BOLD:AAZ9597	MF546327	658[0n]	2011	Entre Ríos 3
Cymaenes odilia	MACN-Bar-Lep-ct 03129	LEPPA050-11	BOLD:AAZ9597	MF547309	658[0n]	2011	Buenos Aires 21
Cymaenes odilia	MACN-Bar-Lep-ct 03145	LEPPA063-11	BOLD:AAZ9597	MF546359	658[0n]	2011	Buenos Aires 21
Cymaenes odilia	MACN-Bar-Lep-ct 03151	LEPPA067-11	BOLD:AAZ9597	MF546887	658[0n]	2011	Buenos Aires 21
Cymaenes odilia	MACN-Bar-Lep-ct 06492	LEPPA1080-14			0	2013	Buenos Aires 28
Dalla diraspes	MACN-Bar-Lep-ct 01953	LEPIG075-11	BOLD:AAZ9348	MF545463	658[0n]	2011	Misiones 2
Dalla diraspes	MACN-Bar-Lep-ct 01981	LEPIG100-11	BOLD:AAZ9348	MF545426	658[0n]	2011	Misiones 2
Danaus eresimus	MACN-Bar-Lep-ct 01841	LEPAR265-11	BOLD:AAZ7478	MF546976	658[0n]	2011	Entre Ríos 3
Danaus eresimus	MACN-Bar-Lep-ct 01845	LEPAR269-11	BOLD:AAZ7478	MF547229	658[0n]	2011	Entre Ríos 3
Danaus eresimus	MACN-Bar-Lep-ct 01852	LEPAR273-11	BOLD:AAZ7478	MF546063	658[0n]	2011	Entre Ríos 3
Danaus eresimus	MACN-Bar-Lep-ct 01907	LEPIG034-11	BOLD:AAZ7478	MF545524	658[0n]	2011	Misiones 2
Danaus eresimus	MACN-Bar-Lep-ct 02578	LEPPA233-12	BOLD:AAZ7478	MF547150	658[0n]	2011	Formosa 2
Danaus eresimus	MACN-Bar-Lep-ct 02660	LEPPA313-12	BOLD:AAZ7478	MF547199	658[0n]	2011	Formosa 2
Danaus eresimus	MACN-Bar-Lep-ct 02674	LEPPA326-12	BOLD:AAZ7478	MF546876	658[0n]	2011	Formosa 2
Danaus eresimus	MACN-Bar-Lep-ct 02688	LEPPA337-12	BOLD:AAZ7478	MF545781	658[0n]	2011	Formosa 2
Danaus eresimus	MACN-Bar-Lep-ct 02755	LEPPA384-12	BOLD:AAZ7478	MF546588	658[0n]	2011	Formosa 2
Danaus eresimus	MACN-Bar-Lep-ct 03942	LEPPA567-13	BOLD:AAZ7478	MF546634	658[0n]	2012	Formosa 4

Danaus eresimus	MACN-Bar-Lep-ct 03950	LEPPA573-13	BOLD:AAZ7478	MF546360	658[0n]	2012	Formosa 4
Danaus eresimus	MACN-Bar-Lep-ct 06074	LEPAR1007-14	BOLD:AAZ7478	MF545410	658[0n]	2013	Formosa 5
Danaus erippus	MACN-Bar-Lep-ct 01649	LEPAR193-11	BOLD:AAZ7818	MF545722	658[0n]	2011	Entre Ríos 4
Danaus erippus	MACN-Bar-Lep-ct 01718	LEPAR223-11	BOLD:AAZ7818	MF547376	658[0n]	2011	Entre Ríos 4
Danaus erippus	MACN-Bar-Lep-ct 01731	LEPAR229-11	BOLD:AAZ7818	MF547205	658[0n]	2011	Entre Ríos 3
Danaus erippus	MACN-Bar-Lep-ct 01843	LEPAR267-11	BOLD:AAZ7818	MF546788	658[0n]	2011	Entre Ríos 3
Danaus erippus	MACN-Bar-Lep-ct 02480	LEPIG519-11	BOLD:AAZ7818	MF547103	658[0n]	2011	Misiones 3
Danaus erippus	MACN-Bar-Lep-ct 03104	LEPPA027-11	BOLD:AAZ7818	MF546463	658[0n]	2010	Buenos Aires 21
Danaus erippus	MACN-Bar-Lep-ct 03119	LEPPA042-11			0	2010	Buenos Aires 21
Danaus erippus	MACN-Bar-Lep-ct 03137	LEPPA057-11	BOLD:AAZ7818	MF545446	658[0n]	2011	Buenos Aires 21
Danaus erippus	MACN-Bar-Lep-ct 03610	LEPPA731-13	BOLD:AAZ7818	MF547289	658[0n]	2011	Corrientes 1
Danaus erippus	MACN-Bar-Lep-ct 03685	LEPPA773-13	BOLD:AAZ7818	MF547039	658[0n]	2011	Corrientes 1
Danaus erippus	MACN-Bar-Lep-ct 03738	LEPPA813-13	BOLD:AAZ7818	MF546304	658[0n]	2011	Corrientes 1
Danaus erippus	MACN-Bar-Lep-ct 03938	LEPPA563-13	BOLD:AAZ7818	MF546720	658[0n]	2012	Formosa 4
Danaus erippus	MACN-Bar-Lep-ct 03952	LEPPA575-13	BOLD:AAZ7818	MF547085	658[0n]	2012	Formosa 4
Danaus gilippus	MACN-Bar-Lep-ct 02727	LEPPA364-12	BOLD:AAD6393	MF547087	658[0n]	2011	Formosa 3
Danaus gilippus	MACN-Bar-Lep-ct 03592	LEPPA717-13	BOLD:AAD6393	MF545772	658[0n]	2011	Corrientes 1
Danaus gilippus	MACN-Bar-Lep-ct 03603	LEPPA726-13	BOLD:AAD6393	MF546830	585[0n]	2011	Corrientes 1
Danaus gilippus	MACN-Bar-Lep-ct 03659	LEPPA758-13	BOLD:AAD6393	MF546030	658[0n]	2011	Corrientes 1
Danaus gilippus	MACN-Bar-Lep-ct 03750	LEPPA821-13	BOLD:AAD6393	MF545590	658[0n]	2011	Corrientes 1
Danaus gilippus	MACN-Bar-Lep-ct 03759	LEPPA826-13	BOLD:AAD6393	MF546519	658[0n]	2011	Corrientes 1
Danaus gilippus	MACN-Bar-Lep-ct 06023	LEPAR956-14	BOLD:AAD6393	MF546544	658[5n]	2013	Formosa 5
Decinea dama	MACN-Bar-Lep-ct 01053	LEPAR746-11	BOLD:AAZ1155	MF546513	658[0n]	2010	Misiones 2
Decinea dama	MACN-Bar-Lep-ct 01054	LEPAR747-11	BOLD:AAZ1155	MF546535	658[0n]	2010	Misiones 2
Decinea dama	MACN-Bar-Lep-ct 02079	LEPIG183-11	BOLD:AAZ1155	MF546170	658[0n]	2011	Misiones 2
Denivia hemon	MACN-Bar-Lep-ct 02531	LEPIG552-11	BOLD:AAB2481	MF546307	658[1n]	2011	Misiones 3
Diaethria candrena	MACN-Bar-Lep-ct 00071	LEPAR051-11	BOLD:AAI9429	MF547046	658[0n]	2007	Misiones 1
Diaethria candrena	MACN-Bar-Lep-ct 00072	LEPAR052-11	BOLD:AAI9429	MF546562	658[0n]		Misiones 16
Diaethria candrena	MACN-Bar-Lep-ct 00333	LEPAR379-11	BOLD:AAI9429	MF547283	658[0n]	2010	Misiones 2
Diaethria candrena	MACN-Bar-Lep-ct 00388	LEPAR411-11	BOLD:AAI9429	MF547185	658[0n]	2010	Misiones 2
Diaethria candrena	MACN-Bar-Lep-ct 00402	LEPAR419-11	BOLD:AAI9429	MF546601	658[0n]	2010	Misiones 2
Diaethria candrena	MACN-Bar-Lep-ct 00592	LEPAR518-11	BOLD:AAI9429	MF546553	658[0n]	2010	Misiones 2
Diaethria candrena	MACN-Bar-Lep-ct 00841	LEPAR615-11	BOLD:AAI9429	MF546223	658[0n]	2010	Misiones 2

Diaethria candrena	MACN-Bar-Lep-ct 01763	LEPAR241-11	BOLD:AAI9429	MF547122	658[0n]	2011	Entre Ríos 6
Diaethria candrena	MACN-Bar-Lep-ct 01849	LEPAR272-11	BOLD:AAI9429	MF546291	658[0n]	2011	Entre Ríos 3
Diaethria clymena	MACN-Bar-Lep-ct 00070	LEPAR050-11	BOLD:ACV2944	MF545597	658[0n]	2007	Misiones 1
Diaethria clymena	MACN-Bar-Lep-ct 00248	LEPAR331-11	BOLD:ACV2944	MF546197	658[0n]	2010	Misiones 2
Diaethria clymena	MACN-Bar-Lep-ct 00372	LEPAR404-11	BOLD:ACV2944	MF545777	658[0n]	2010	Misiones 2
Diaethria clymena	MACN-Bar-Lep-ct 00574	LEPAR502-11	BOLD:ACV2944	MF546798	658[0n]	2010	Misiones 2
Diaethria clymena	MACN-Bar-Lep-ct 00575	LEPAR503-11	BOLD:ACV2944	MF546864	658[0n]	2010	Misiones 2
Diaethria clymena	MACN-Bar-Lep-ct 00598	LEPAR523-11	BOLD:ACV2944	MF546446	658[0n]	2010	Misiones 2
Diaethria clymena	MACN-Bar-Lep-ct 01931	LEPIG054-11	BOLD:ACV2944	MF545654	658[0n]	2011	Misiones 2
Diaethria clymena	MACN-Bar-Lep-ct 01945	LEPIG067-11	BOLD:ACV2944	MF545586	658[0n]	2011	Misiones 2
Diaethria clymena	MACN-Bar-Lep-ct 02238	LEPIG327-11	BOLD:ACV2944	MF546700	658[0n]	2011	Misiones 3
Diaethria clymena	MACN-Bar-Lep-ct 02582	LEPPA237-12	BOLD:ACV2944	MF547295	658[0n]	2011	Formosa 2
Diaethria clymena	MACN-Bar-Lep-ct 02599	LEPPA254-12	BOLD:ACV2944	MF546061	658[0n]	2011	Formosa 2
Dione juno	MACN-Bar-Lep-ct 00302	LEPAR361-11	BOLD:AAZ3761	MF545656	658[0n]	2010	Misiones 2
Dione juno	MACN-Bar-Lep-ct 00551	LEPAR481-11	BOLD:AAZ3761	MF546398	658[0n]	2010	Misiones 2
Dione juno	MACN-Bar-Lep-ct 00559	LEPAR487-11	BOLD:AAZ3761	MF547323	658[0n]	2010	Misiones 2
Dione juno	MACN-Bar-Lep-ct 00563	LEPAR491-11	BOLD:AAZ3761	MF546260	658[0n]	2010	Misiones 2
Dione juno	MACN-Bar-Lep-ct 00567	LEPAR495-11	BOLD:AAZ3761	MF545411	658[0n]	2010	Misiones 2
Dione juno	MACN-Bar-Lep-ct 02090	LEPIG193-11	BOLD:AAZ3761	MF545554	658[0n]	2011	Misiones 2
Dione juno	MACN-Bar-Lep-ct 02124	LEPIG221-11	BOLD:AAZ3761	MF547152	658[0n]	2011	Misiones 2
Dione juno	MACN-Bar-Lep-ct 02285	LEPIG371-11	BOLD:AAZ3761	MF547005	658[0n]	2011	Misiones 3
Dione juno	MACN-Bar-Lep-ct 03586	LEPPA711-13	BOLD:AAZ3761	MF545965	658[0n]	2011	Corrientes 1
Dircenna dero	MACN-Bar-Lep-ct 00227	LEPAR316-11	BOLD:ACE7122	MF546755	658[0n]	2010	Misiones 2
Dircenna dero	MACN-Bar-Lep-ct 01162	LEPAR790-11	BOLD:ACE7122	MF547395	658[0n]	2010	Misiones 2
Dircenna dero	MACN-Bar-Lep-ct 01874	LEPIG002-11	BOLD:ACE7122	MF546779	658[0n]	2011	Misiones 2
Dircenna dero	MACN-Bar-Lep-ct 01897	LEPIG025-11	BOLD:ACE7122	MF546476	658[0n]	2011	Misiones 2
Dircenna dero	MACN-Bar-Lep-ct 01903	LEPIG031-11	BOLD:ACE7122	MF546943	658[0n]	2011	Misiones 2
Dircenna dero	MACN-Bar-Lep-ct 02043	LEPIG152-11	BOLD:ACE7122	MF546871	658[0n]	2011	Misiones 2
Dircenna dero	MACN-Bar-Lep-ct 02047	LEPIG156-11	BOLD:ACE7122	MF546609	658[0n]	2011	Misiones 2
Dircenna dero	MACN-Bar-Lep-ct 02058	LEPIG165-11	BOLD:ACE7122	MF545451	658[0n]	2011	Misiones 2
Dircenna dero	MACN-Bar-Lep-ct 02591	LEPPA246-12	BOLD:ACE7122	MF547207	658[0n]	2011	Formosa 2
Dircenna dero	MACN-Bar-Lep-ct 02750	LEPPA380-12	BOLD:ACE7122	MF546666	658[0n]	2011	Formosa 2
Dismorphia amphione	MACN-Bar-Lep-ct 01337	LEPAR918-11	BOLD:AAZ5008	MF547389	658[0n]	2010	Misiones 2

Doxocopa agathina	MACN-Bar-Lep-ct 00231	LEPAR320-11	BOLD:AAZ3880	MF545798	658[0n]	2010	Misiones 2
Doxocopa agathina	MACN-Bar-Lep-ct 00307	LEPAR363-11	BOLD:AAZ3880	MF545468	658[0n]	2010	Misiones 2
Doxocopa agathina	MACN-Bar-Lep-ct 00325	LEPAR373-11	BOLD:AAZ3880	MF546983	658[0n]	2010	Misiones 2
Doxocopa agathina	MACN-Bar-Lep-ct 00569	LEPAR497-11	BOLD:AAZ3880	MF546437	658[0n]	2010	Misiones 2
Doxocopa agathina	MACN-Bar-Lep-ct 00863	LEPAR632-11	BOLD:AAZ3880	MF547328	658[0n]	2010	Misiones 2
Doxocopa kallina	MACN-Bar-Lep-ct 00276	LEPAR342-11	BOLD:AAZ2685	MF546432	658[0n]	2010	Misiones 2
Doxocopa kallina	MACN-Bar-Lep-ct 00283	LEPAR347-11	BOLD:AAZ2685	MF546967	658[0n]	2010	Misiones 2
Doxocopa kallina	MACN-Bar-Lep-ct 00844	LEPAR617-11	BOLD:AAZ2685	MF546387	658[0n]	2010	Misiones 2
Doxocopa kallina	MACN-Bar-Lep-ct 01152	LEPAR784-11	BOLD:AAZ2685	MF546528	658[0n]	2010	Misiones 2
Doxocopa kallina	MACN-Bar-Lep-ct 01863	LEPAR280-11	BOLD:AAZ2685	MF546408	658[0n]	2011	Entre Ríos 3
Doxocopa kallina	MACN-Bar-Lep-ct 02655	LEPPA308-12	BOLD:AAZ2685	MF545450	658[0n]	2011	Formosa 2
Doxocopa kallina	MACN-Bar-Lep-ct 02659	LEPPA312-12	BOLD:AAZ2685	MF546342	658[0n]	2011	Formosa 2
Doxocopa kallina	MACN-Bar-Lep-ct 02782	LEPPA400-12	BOLD:AAZ2685	MF545585	658[0n]	2011	Formosa 2
Doxocopa kallina	MACN-Bar-Lep-ct 03776	LEPPA833-13	BOLD:AAZ2685	MF545742	658[0n]	2011	Corrientes 1
Doxocopa laurentia	MACN-Bar-Lep-ct 00015	LEPAR009-11	BOLD:AAY8174	MF546480	658[0n]	2010	Buenos Aires 21
Doxocopa laurentia	MACN-Bar-Lep-ct 00157	LEPAR286-11	BOLD:AAY8174	MF546843	658[0n]	2010	Misiones 2
Doxocopa laurentia	MACN-Bar-Lep-ct 00228	LEPAR317-11	BOLD:AAY8174	MF545884	658[0n]	2010	Misiones 2
Doxocopa laurentia	MACN-Bar-Lep-ct 00284	LEPAR348-11	BOLD:AAY8174	MF546685	658[0n]	2010	Misiones 2
Doxocopa laurentia	MACN-Bar-Lep-ct 00585	LEPAR512-11	BOLD:AAY8174	MF546339	658[0n]	2010	Misiones 2
Doxocopa laurentia	MACN-Bar-Lep-ct 00591	LEPAR517-11	BOLD:AAY8174	MF546214	658[0n]	2010	Misiones 2
Doxocopa laurentia	MACN-Bar-Lep-ct 02138	LEPIG235-11	BOLD:AAY8174	MF547343	658[0n]	2011	Misiones 3
Doxocopa laurentia	MACN-Bar-Lep-ct 03144	LEPPA062-11	BOLD:AAY8174	MF545606	658[0n]	2011	Buenos Aires 21
Doxocopa linda	MACN-Bar-Lep-ct 00065	LEPAR045-11			0	1989	Misiones 4
Doxocopa linda	MACN-Bar-Lep-ct 00066	LEPAR046-11			0		Misiones 1
Doxocopa linda	MACN-Bar-Lep-ct 00249	LEPAR332-11	BOLD:AAI9814	MF546662	658[0n]	2010	Misiones 2
Doxocopa linda	MACN-Bar-Lep-ct 00275	LEPAR341-11	BOLD:AAI9814	MF546767	658[0n]	2010	Misiones 2
Doxocopa linda	MACN-Bar-Lep-ct 00298	LEPAR359-11	BOLD:AAI9814	MF545598	658[0n]	2010	Misiones 2
Doxocopa linda	MACN-Bar-Lep-ct 00532	LEPAR469-11	BOLD:AAI9814	MF546880	658[0n]	2010	Misiones 2
Doxocopa linda	MACN-Bar-Lep-ct 01212	LEPAR830-11	BOLD:AAI9814	MF547266	658[0n]	2010	Misiones 2
Doxocopa linda	MACN-Bar-Lep-ct 02125	LEPIG222-11	BOLD:AAI9814	MF546938	658[0n]	2011	Misiones 2
Doxocopa linda	MACN-Bar-Lep-ct 02180	LEPIG274-11	BOLD:AAI9814	MF545395	658[0n]	2011	Misiones 3
Doxocopa linda	MACN-Bar-Lep-ct 02191	LEPIG281-11	BOLD:AAI9814	MF546518	658[0n]	2011	Misiones 3
Doxocopa linda	MACN-Bar-Lep-ct 02815	LEPPA412-12	BOLD:AAI9814	MF547281	658[0n]	2011	Formosa 2

Doxocopa zunilda	MACN-Bar-Lep-ct 00234	LEPAR321-11	BOLD:AAZ3962	MF546980	658[0n]	2010	Misiones 2
Doxocopa zunilda	MACN-Bar-Lep-ct 00365	LEPAR400-11	BOLD:AAZ3962	MF546027	658[0n]	2010	Misiones 2
Doxocopa zunilda	MACN-Bar-Lep-ct 00580	LEPAR508-11	BOLD:AAZ3962	MF546958	658[0n]	2010	Misiones 2
Doxocopa zunilda	MACN-Bar-Lep-ct 00790	LEPAR572-11	BOLD:AAZ3962	MF546507	658[0n]	2010	Misiones 2
Doxocopa zunilda	MACN-Bar-Lep-ct 00838	LEPAR612-11	BOLD:AAZ3962	MF546968	658[0n]	2010	Misiones 2
Doxocopa zunilda	MACN-Bar-Lep-ct 02534	LEPIG555-11	BOLD:AAZ3962	MF545968	658[0n]	2011	Misiones 3
Doxocopa zunilda	MACN-Bar-Lep-ct 02555	LEPIG572-11	BOLD:AAZ3962	MF546690	649[0n]	2011	Misiones 3
Dryadula phaetusa	MACN-Bar-Lep-ct 02577	LEPPA232-12	BOLD:AAD0315	MF545532	658[0n]	2011	Formosa 2
Dryadula phaetusa	MACN-Bar-Lep-ct 02597	LEPPA252-12	BOLD:AAD0315	MF547014	658[0n]	2011	Formosa 2
Dryadula phaetusa	MACN-Bar-Lep-ct 02611	LEPPA266-12	BOLD:AAD0315	MF547268	658[0n]	2011	Formosa 2
Dryadula phaetusa	MACN-Bar-Lep-ct 02619	LEPPA273-12	BOLD:AAD0315	MF545999	658[0n]	2011	Formosa 2
Dryadula phaetusa	MACN-Bar-Lep-ct 02635	LEPPA288-12	BOLD:AAD0315	MF545441	658[0n]	2011	Formosa 2
Dryadula phaetusa	MACN-Bar-Lep-ct 02653	LEPPA306-12	BOLD:AAD0315	MF547038	658[0n]	2011	Formosa 2
Dryadula phaetusa	MACN-Bar-Lep-ct 03646	LEPPA748-13	BOLD:AAD0315	MF545491	658[0n]	2011	Corrientes 1
Dryadula phaetusa	MACN-Bar-Lep-ct 06061	LEPAR994-14	BOLD:AAD0315	MF545710	658[0n]	2013	Formosa 5
Dryas iulia	MACN-Bar-Lep-ct 00226	LEPAR315-11	BOLD:AAB6669	MF546705	658[0n]	2010	Misiones 2
Dryas iulia	MACN-Bar-Lep-ct 00581	LEPAR509-11	BOLD:AAB6669	MF546009	658[0n]	2010	Misiones 2
Dryas iulia	MACN-Bar-Lep-ct 00983	LEPAR689-11	BOLD:AAB6669	MF545724	658[0n]	2010	Misiones 2
Dryas iulia	MACN-Bar-Lep-ct 01151	LEPAR783-11	BOLD:AAB6669	MF545464	658[0n]	2010	Misiones 2
Dryas iulia	MACN-Bar-Lep-ct 01185	LEPAR807-11	BOLD:AAB6669	MF546465	658[0n]	2010	Misiones 2
Dryas iulia	MACN-Bar-Lep-ct 01916	LEPIG042-11	BOLD:AAB6669	MF547029	658[0n]	2011	Misiones 2
Dryas iulia	MACN-Bar-Lep-ct 01933	LEPIG056-11	BOLD:AAB6669	MF547044	658[0n]	2011	Misiones 2
Dryas iulia	MACN-Bar-Lep-ct 02282	LEPIG368-11	BOLD:AAB6669	MF546290	658[0n]	2011	Misiones 3
Dryas iulia	MACN-Bar-Lep-ct 02585	LEPPA240-12	BOLD:AAB6669	MF546278	658[0n]	2011	Formosa 2
Dryas iulia	MACN-Bar-Lep-ct 02818	LEPPA413-12	BOLD:AAB6669	MF545896	658[0n]	2011	Formosa 2
Dryas iulia	MACN-Bar-Lep-ct 03576	LEPPA707-13	BOLD:AAB6669	MF546587	658[0n]	2011	Corrientes 1
Dryas iulia	MACN-Bar-Lep-ct 06056	LEPAR989-14	BOLD:AAB6669	MF547311	658[3n]	2013	Formosa 5
Dynamine aerata	MACN-Bar-Lep-ct 02172	LEPIG266-11	BOLD:AAI8873	MF547326	658[0n]	2011	Misiones 3
Dynamine aerata	MACN-Bar-Lep-ct 02177	LEPIG271-11	BOLD:AAI8873	MF546054	658[0n]	2011	Misiones 3
Dynamine agacles	MACN-Bar-Lep-ct 00401	LEPAR418-11	BOLD:AAD0437	MF546590	658[0n]	2010	Misiones 2
Dynamine agacles	MACN-Bar-Lep-ct 00606	LEPAR529-11	BOLD:AAD0437	MF547333	658[0n]	2010	Misiones 2
Dynamine agacles	MACN-Bar-Lep-ct 00612	LEPAR534-11	BOLD:AAD0437	MF545555	658[0n]	2010	Misiones 2
Dynamine agacles	MACN-Bar-Lep-ct 00613	LEPAR535-11	BOLD:AAD0437	MF546185	658[0n]	2010	Misiones 2

Dynamine agacles	MACN-Bar-Lep-ct 00614	LEPAR536-11	BOLD:AAD0437	MF545600	658[0n]	2010	Misiones 2
Dynamine agacles	MACN-Bar-Lep-ct 02120	LEPIG217-11	BOLD:AAD0437	MF545481	658[0n]	2011	Misiones 2
Dynamine artemisia	MACN-Bar-Lep-ct 00601	LEPAR525-11	BOLD:AAC6997	MF546396	658[0n]	2010	Misiones 2
Dynamine artemisia	MACN-Bar-Lep-ct 00825	LEPAR601-11	BOLD:AAC6997	MF547156	658[0n]	2010	Misiones 2
Dynamine artemisia	MACN-Bar-Lep-ct 00840	LEPAR614-11	BOLD:AAC6997	MF545844	658[0n]	2010	Misiones 2
Dynamine artemisia	MACN-Bar-Lep-ct 00979	LEPAR685-11	BOLD:AAC6997	MF546319	658[0n]	2010	Misiones 2
Dynamine artemisia	MACN-Bar-Lep-ct 01160	LEPAR788-11	BOLD:AAC6997	MF546213	658[0n]	2010	Misiones 2
Dynamine artemisia	MACN-Bar-Lep-ct 02094	LEPIG197-11	BOLD:AAC6997	MF546459	658[0n]	2011	Misiones 2
Dynamine artemisia	MACN-Bar-Lep-ct 02178	LEPIG272-11	BOLD:AAC6997	MF546981	658[0n]	2011	Misiones 3
Dynamine artemisia	MACN-Bar-Lep-ct 02398	LEPIG463-11	BOLD:AAC6997	MF547391	658[0n]	2011	Misiones 3
Dynamine athemon	MACN-Bar-Lep-ct 00610	LEPAR532-11	BOLD:AAB2028	MF546992	658[0n]	2010	Misiones 2
Dynamine athemon	MACN-Bar-Lep-ct 01299	LEPAR881-11	BOLD:AAB2028	MF547351	658[0n]	2010	Misiones 2
Dynamine athemon	MACN-Bar-Lep-ct 01314	LEPAR896-11	BOLD:AAB2028	MF547243	658[0n]	2010	Misiones 2
Dynamine athemon	MACN-Bar-Lep-ct 01349	LEPAR930-11	BOLD:AAB2028	MF546106	658[0n]	2010	Misiones 2
Dynamine athemon	MACN-Bar-Lep-ct 01900	LEPIG028-11	BOLD:AAB2028	MF547089	658[0n]	2011	Misiones 2
Dynamine athemon	MACN-Bar-Lep-ct 02012	LEPIG127-11	BOLD:AAB2028	MF546858	658[0n]	2011	Misiones 2
Dynamine athemon	MACN-Bar-Lep-ct 02169	LEPIG264-11	BOLD:AAB2028	MF547184	658[0n]	2011	Misiones 3
Dynamine athemon	MACN-Bar-Lep-ct 02221	LEPIG310-11	BOLD:AAB2028	MF547278	658[0n]	2011	Misiones 3
Dynamine coenus	MACN-Bar-Lep-ct 00603	LEPAR526-11	BOLD:AAD7882	MF546375	658[0n]	2010	Misiones 2
Dynamine coenus	MACN-Bar-Lep-ct 00608	LEPAR530-11	BOLD:AAD7882	MF546332	658[0n]	2010	Misiones 2
Dynamine coenus	MACN-Bar-Lep-ct 00609	LEPAR531-11	BOLD:AAD7882	MF545387	658[0n]	2010	Misiones 2
Dynamine coenus	MACN-Bar-Lep-ct 00611	LEPAR533-11	BOLD:AAD7882	MF546882	658[0n]	2010	Misiones 2
Dynamine coenus	MACN-Bar-Lep-ct 00845	LEPAR618-11	BOLD:AAD7882	MF546045	658[0n]	2010	Misiones 2
Dynamine coenus	MACN-Bar-Lep-ct 01304	LEPAR886-11	BOLD:AAD7882	MF546048	658[0n]	2010	Misiones 2
Dynamine coenus	MACN-Bar-Lep-ct 01308	LEPAR890-11	BOLD:AAD7882	MF545681	658[0n]	2010	Misiones 2
Dynamine coenus	MACN-Bar-Lep-ct 02151	LEPIG247-11	BOLD:AAD7882	MF547063	658[0n]	2011	Misiones 3
Dynamine coenus	MACN-Bar-Lep-ct 07083	LEPAR1161-15			0	1998	Misiones 12
Dynamine meridionalis	MACN-Bar-Lep-ct 02468	LEPIG512-11	BOLD:AAY0568	MF546555	658[0n]	2011	Misiones 3
Dynamine myrrhina	MACN-Bar-Lep-ct 00236	LEPAR323-11	BOLD:AAE0990	MF547071	658[0n]	2010	Misiones 2
Dynamine myrrhina	MACN-Bar-Lep-ct 00379	LEPAR410-11	BOLD:AAE0990	MF546931	658[0n]	2010	Misiones 2
Dynamine myrrhina	MACN-Bar-Lep-ct 01012	LEPAR711-11	BOLD:AAE0990	MF546654	658[0n]	2010	Misiones 2
Dynamine myrrhina	MACN-Bar-Lep-ct 01030	LEPAR726-11	BOLD:AAE0990	MF546273	658[0n]	2010	Misiones 2
Dynamine myrrhina	MACN-Bar-Lep-ct 01316	LEPAR898-11	BOLD:AAE0990	MF547077	658[0n]	2010	Misiones 2

Dynamine postverta	MACN-Bar-Lep-ct 01019	LEPAR716-11	BOLD:AAB5434	MF546277	658[0n]	2010	Misiones 2
Dynamine postverta	MACN-Bar-Lep-ct 01020	LEPAR717-11	BOLD:AAB5434	MF546001	658[0n]	2010	Misiones 2
Dynamine postverta	MACN-Bar-Lep-ct 01305	LEPAR887-11	BOLD:AAB5434	MF547091	658[0n]	2010	Misiones 2
Dynamine postverta	MACN-Bar-Lep-ct 01350	LEPAR931-11	BOLD:AAB5434	MF546597	658[0n]	2010	Misiones 2
Dynamine postverta	MACN-Bar-Lep-ct 01351	LEPAR932-11	BOLD:AAB5434	MF545593	658[0n]	2010	Misiones 2
Dynamine postverta	MACN-Bar-Lep-ct 01891	LEPIG019-11	BOLD:AAB5434	MF545627	658[0n]	2011	Misiones 2
Dynamine postverta	MACN-Bar-Lep-ct 02552	LEPIG569-11	BOLD:AAB5434	MF546884	658[0n]	2011	Misiones 3
Dynamine tithia	MACN-Bar-Lep-ct 00807	LEPAR585-11	BOLD:AAD7883	MF546270	658[0n]	2010	Misiones 2
Dynamine tithia	MACN-Bar-Lep-ct 00823	LEPAR599-11	BOLD:AAD7883	MF545701	658[0n]	2010	Misiones 2
Dynamine tithia	MACN-Bar-Lep-ct 00981	LEPAR687-11	BOLD:AAD7883	MF546356	658[0n]	2010	Misiones 2
Dynamine tithia	MACN-Bar-Lep-ct 01172	LEPAR798-11	BOLD:AAD7883	MF547314	658[0n]	2010	Misiones 2
Dynamine tithia	MACN-Bar-Lep-ct 01201	LEPAR820-11	BOLD:AAD7883	MF546409	658[0n]	2010	Misiones 2
Dynamine tithia	MACN-Bar-Lep-ct 02237	LEPIG326-11	BOLD:AAD7883	MF546935	658[0n]	2011	Misiones 3
Dynamine tithia	MACN-Bar-Lep-ct 02549	LEPIG567-11	BOLD:AAD7883	MF545531	658[0n]	2011	Misiones 3
Eantis thraso	MACN-Bar-Lep-ct 00346	LEPAR387-11	BOLD:ABZ2239	MF547006	658[0n]	2010	Misiones 2
Eantis thraso	MACN-Bar-Lep-ct 00421	LEPAR433-11	BOLD:ABZ2239	MF546355	658[0n]	2010	Misiones 2
Eantis thraso	MACN-Bar-Lep-ct 00422	LEPAR434-11	BOLD:ABZ2239	MF546259	658[0n]	2010	Misiones 2
Eantis thraso	MACN-Bar-Lep-ct 00596	LEPAR521-11	BOLD:ABZ2239	MF545792	658[0n]	2010	Misiones 2
Eantis thraso	MACN-Bar-Lep-ct 00856	LEPAR625-11	BOLD:ABZ2239	MF545680	658[0n]	2010	Misiones 2
Eantis thraso	MACN-Bar-Lep-ct 01993	LEPIG110-11	BOLD:ABZ2239	MF547096	658[0n]	2011	Misiones 2
Eantis thraso	MACN-Bar-Lep-ct 02353	LEPIG428-11	BOLD:ABZ2239	MF546910	658[0n]	2011	Misiones 3
Eantis thraso	MACN-Bar-Lep-ct 02570	LEPIG585-11	BOLD:ABZ2239	MF546318	658[0n]	2011	Misiones 3
Eantis thraso	MACN-Bar-Lep-ct 03575	LEPPA706-13	BOLD:ACG2436	MF545412	658[0n]	2011	Corrientes 1
Eantis thraso	MACN-Bar-Lep-ct 03636	LEPPA742-13	BOLD:ACG2167	MF546756	658[0n]	2011	Corrientes 1
Eantis thraso	MACN-Bar-Lep-ct 06051	LEPAR984-14	BOLD:ACG2167	MF546145	658[4n]	2013	Formosa 5
Ebrietas anacreon	MACN-Bar-Lep-ct 01057	LEPAR750-11	BOLD:AAB1461	MF545816	658[0n]	2010	Misiones 2
Ebrietas anacreon	MACN-Bar-Lep-ct 01196	LEPAR817-11	BOLD:AAB1461	MF545628	658[0n]	2010	Misiones 2
Ebrietas anacreon	MACN-Bar-Lep-ct 01954	LEPIG076-11	BOLD:AAB1461	MF547269	658[0n]	2011	Misiones 2
Ebrietas anacreon	MACN-Bar-Lep-ct 01960	LEPIG082-11	BOLD:AAB1461	MF546744	658[0n]	2011	Misiones 2
Ebrietas anacreon	MACN-Bar-Lep-ct 02002	LEPIG119-11	BOLD:AAB1461	MF547078	658[0n]	2011	Misiones 2
Ebrietas anacreon	MACN-Bar-Lep-ct 02052	LEPIG160-11	BOLD:AAB1461	MF545764	658[0n]	2011	Misiones 2
Ebrietas anacreon	MACN-Bar-Lep-ct 02361	LEPIG432-11	BOLD:AAB1461	MF545485	658[0n]	2011	Misiones 3
Ebrietas anacreon	MACN-Bar-Lep-ct 02401	LEPIG466-11	BOLD:AAB1461	MF546949	658[0n]	2011	Misiones 3

Ebrietas infanda	MACN-Bar-Lep-ct 02365	LEPIG436-11	BOLD:AAZ9508	MF546225	658[0n]	2011	Misiones 3
Ectima thecla	MACN-Bar-Lep-ct 01877	LEPIG005-11	BOLD:AAH4577	MF547385	658[0n]	2011	Misiones 2
Ectima thecla	MACN-Bar-Lep-ct 01948	LEPIG070-11	BOLD:AAH4577	MF546051	658[0n]	2011	Misiones 2
Ectima thecla	MACN-Bar-Lep-ct 02152	LEPIG248-11	BOLD:AAH4577	MF545645	658[0n]	2011	Misiones 3
Ectima thecla	MACN-Bar-Lep-ct 02170	LEPIG265-11	BOLD:AAH4577	MF545437	658[0n]	2011	Misiones 3
Ectima thecla	MACN-Bar-Lep-ct 02252	LEPIG340-11	BOLD:AAH4577	MF546333	658[0n]	2011	Misiones 3
Ectima thecla	MACN-Bar-Lep-ct 02277	LEPIG364-11	BOLD:AAH4577	MF545695	658[0n]	2011	Misiones 3
Elbella adonis	MACN-Bar-Lep-ct 00278	LEPAR344-11	BOLD:AAX7263	MF545768	658[0n]	2010	Misiones 2
Elbella adonis	MACN-Bar-Lep-ct 00882	LEPAR649-11	BOLD:AAX7263	MF547322	658[0n]	2010	Misiones 2
Elbella adonis	MACN-Bar-Lep-ct 01050	LEPAR743-11	BOLD:AAX7263	MF546620	658[0n]	2010	Misiones 2
Elbella adonis	MACN-Bar-Lep-ct 01213	LEPAR831-11	BOLD:AAX7263	MF545462	658[0n]	2010	Misiones 2
Elbella lamprus	MACN-Bar-Lep-ct 00263	LEPAR336-11	BOLD:AAF5350	MF546104	658[0n]	2010	Misiones 2
Electrostrymon endymion	MACN-Bar-Lep-ct 03827	LEPPA863-13	BOLD:ACG2733	MF545903	602[0n]	2011	Corrientes 1
Emesis diogenia	MACN-Bar-Lep-ct 01040	LEPAR733-11	BOLD:AAZ1263	MF546164	658[0n]	2010	Misiones 2
Emesis diogenia	MACN-Bar-Lep-ct 02223	LEPIG312-11	BOLD:AAZ1263	MF547241	658[0n]	2011	Misiones 3
Emesis diogenia	MACN-Bar-Lep-ct 02225	LEPIG314-11	BOLD:AAZ1263	MF546018	658[0n]	2011	Misiones 3
Emesis diogenia	MACN-Bar-Lep-ct 02430	LEPIG489-11	BOLD:AAZ1263	MF546313	658[0n]	2011	Misiones 3
Emesis diogenia	MACN-Bar-Lep-ct 02446	LEPIG500-11	BOLD:AAZ1263	MF546642	658[0n]	2011	Misiones 3
Emesis diogenia	MACN-Bar-Lep-ct 02453	LEPIG504-11	BOLD:AAZ1263	MF546802	658[0n]	2011	Misiones 3
Emesis diogenia	MACN-Bar-Lep-ct 06066	LEPAR999-14	BOLD:AAZ1263	MF546763	658[0n]	2013	Formosa 5
Emesis mandana	MACN-Bar-Lep-ct 01189	LEPAR811-11	BOLD:AAA8843	MF545943	658[0n]	2010	Misiones 2
Emesis mandana	MACN-Bar-Lep-ct 02231	LEPIG320-11	BOLD:AAA8843	MF545674	658[0n]	2011	Misiones 3
Emesis mandana	MACN-Bar-Lep-ct 02233	LEPIG322-11			0	2011	Misiones 3
Emesis mandana	MACN-Bar-Lep-ct 02560	LEPIG576-11	BOLD:AAA8843	MF546326	658[0n]	2011	Misiones 3
Emesis ocypore	MACN-Bar-Lep-ct 00376	LEPAR407-11	BOLD:AAZ5186	MF545987	658[0n]	2010	Misiones 2
Emesis ocypore	MACN-Bar-Lep-ct 02305	LEPIG389-11	BOLD:AAZ5186	MF547239	643[0n]	2011	Misiones 3
Emesis ocypore	MACN-Bar-Lep-ct 02308	LEPIG392-11	BOLD:AAZ5186	MF545565	658[0n]	2011	Misiones 3
Emesis ocypore	MACN-Bar-Lep-ct 02391	LEPIG457-11	BOLD:AAZ5186	MF545827	658[0n]	2011	Misiones 3
Emesis ocypore	MACN-Bar-Lep-ct 02756	LEPPA385-12	BOLD:AAZ5186	MF546546	658[0n]	2011	Formosa 2
Emesis ocypore	MACN-Bar-Lep-ct 02794	LEPPA406-12	BOLD:AAZ5186	MF546264	658[0n]	2011	Formosa 2
Emesis ocypore	MACN-Bar-Lep-ct 03652	LEPPA751-13	BOLD:AAZ5186	MF547160	658[0n]	2011	Corrientes 1
Emesis ocypore	MACN-Bar-Lep-ct 03657	LEPPA756-13	BOLD:AAZ5186	MF546955	658[0n]	2011	Corrientes 1
Emesis ocypore	MACN-Bar-Lep-ct 03845	LEPPA871-13	BOLD:AAZ5186	MF546361	658[0n]	2011	Corrientes 1

Emesis russula	MACN-Bar-Lep-ct 02082	LEPIG186-11	BOLD:AAA8843	MF545899	658[0n]	2011	Misiones 2
Emesis russula	MACN-Bar-Lep-ct 02553	LEPIG570-11	BOLD:AAA8843	MF547221	658[0n]	2011	Misiones 3
Emesis russula	MACN-Bar-Lep-ct 02639	LEPPA292-12	BOLD:AAA8843	MF546877	658[0n]	2011	Formosa 2
Emesis russula	MACN-Bar-Lep-ct 02641	LEPPA294-12	BOLD:AAA8843	MF546311	658[0n]	2011	Formosa 2
Emesis russula	MACN-Bar-Lep-ct 02789	LEPPA403-12	BOLD:AAA8843	MF547291	658[0n]	2011	Formosa 2
Emesis russula	MACN-Bar-Lep-ct 06080	LEPAR1013-14	BOLD:AAA8843	MF546418	658[2n]	2013	Formosa 5
Enantia clarissa	MACN-Bar-Lep-ct 02445	LEPIG499-11	BOLD:ABA0185	MF545705	658[0n]	2011	Misiones 3
Enantia lina	MACN-Bar-Lep-ct 00344	LEPAR385-11	BOLD:AAZ2711	MF546247	658[0n]	2010	Misiones 2
Enantia lina	MACN-Bar-Lep-ct 00356	LEPAR393-11	BOLD:AAZ2711	MF545939	658[0n]	2010	Misiones 2
Enantia lina	MACN-Bar-Lep-ct 00809	LEPAR586-11	BOLD:AAZ2711	MF546253	636[0n]	2010	Misiones 2
Enantia lina	MACN-Bar-Lep-ct 00846	LEPAR619-11	BOLD:AAZ2711	MF547003	658[0n]	2010	Misiones 2
Enantia lina	MACN-Bar-Lep-ct 01215	LEPAR833-11	BOLD:AAZ2711	MF545623	658[0n]	2010	Misiones 2
Enantia lina	MACN-Bar-Lep-ct 01924	LEPIG048-11	BOLD:AAZ2711	MF545904	658[0n]	2011	Misiones 2
Enantia lina	MACN-Bar-Lep-ct 02286	LEPIG372-11	BOLD:AAZ2711	MF547236	658[0n]	2011	Misiones 3
Enos thara	MACN-Bar-Lep-ct 01321	LEPAR902-11	BOLD:AAZ5000	MF545486	658[0n]	2010	Misiones 2
Epargyreus exadeus	MACN-Bar-Lep-ct 02312	LEPIG396-11	BOLD:ABY9026	MF545448	658[0n]	2011	Misiones 3
Epargyreus exadeus	MACN-Bar-Lep-ct 02434	LEPIG493-11	BOLD:ABY9026	MF545889	658[0n]	2011	Misiones 3
Epargyreus exadeus	MACN-Bar-Lep-ct 02441	LEPIG496-11	BOLD:ABY9026	MF547104	658[0n]	2011	Misiones 3
Epargyreus socus	MACN-Bar-Lep-ct 00849	LEPAR622-11	BOLD:ABY9026	MF545735	658[0n]	2010	Misiones 2
Epargyreus socus	MACN-Bar-Lep-ct 01157	LEPAR786-11	BOLD:AAM5836	MF546907	658[0n]	2010	Misiones 2
Epargyreus socus	MACN-Bar-Lep-ct 01170	LEPAR796-11	BOLD:AAM5836	MF547073	658[0n]	2010	Misiones 2
Epargyreus socus	MACN-Bar-Lep-ct 01205	LEPAR823-11	BOLD:ABY9034	MF546739	658[0n]	2010	Misiones 2
Epargyreus socus	MACN-Bar-Lep-ct 01348	LEPAR929-11	BOLD:ABY9026	MF547220	658[0n]	2010	Misiones 2
Epargyreus tmolis	MACN-Bar-Lep-ct 00141	LEPPA116-11	BOLD:ABY9026	MF546961	658[0n]	2010	Buenos Aires 21
Epargyreus tmolis	MACN-Bar-Lep-ct 00142	LEPPA117-11	BOLD:ABY9026	MF545916	658[0n]	2010	Buenos Aires 21
Epargyreus tmolis	MACN-Bar-Lep-ct 00143	LEPPA118-11	BOLD:ABY9026	MF546578	658[0n]	2010	Buenos Aires 21
Epargyreus tmolis	MACN-Bar-Lep-ct 00144	LEPPA119-11	BOLD:ABY9026	MF546613	658[0n]	2010	Buenos Aires 21
Epargyreus tmolis	MACN-Bar-Lep-ct 01805	LEPAR247-11	BOLD:ABY9026	MF546835	658[0n]	2011	Entre Ríos 3
Epargyreus tmolis	MACN-Bar-Lep-ct 03123	LEPPA046-11			0	2011	Buenos Aires 21
Epiphile hubneri	MACN-Bar-Lep-ct 00588	LEPAR514-11	BOLD:AAF0080	MF545947	658[0n]	2010	Misiones 2
Epiphile hubneri	MACN-Bar-Lep-ct 00815	LEPAR592-11	BOLD:AAF0080	MF547264	658[0n]	2010	Misiones 2
Epiphile hubneri	MACN-Bar-Lep-ct 01187	LEPAR809-11	BOLD:AAF0080	MF546328	658[0n]	2010	Misiones 2
Epiphile hubneri	MACN-Bar-Lep-ct 01287	LEPAR873-11	BOLD:AAF0080	MF547027	658[0n]	2010	Misiones 2

Epiphile hubneri	MACN-Bar-Lep-ct 01303	LEPAR885-11	BOLD:AAF0080	MF547110	658[0n]	2010	Misiones 2
Epiphile hubneri	MACN-Bar-Lep-ct 02103	LEPIG205-11	BOLD:AAF0080	MF545493	658[0n]	2011	Misiones 2
Epiphile hubneri	MACN-Bar-Lep-ct 02495	LEPIG529-11	BOLD:AAF0080	MF545791	658[0n]	2011	Misiones 3
Epiphile hubneri	MACN-Bar-Lep-ct 02532	LEPIG553-11	BOLD:AAF0080	MF546668	658[0n]	2011	Misiones 3
Epiphile orea	MACN-Bar-Lep-ct 01294	LEPAR878-11	BOLD:AAE1464	MF547141	658[0n]	2010	Misiones 2
Episcada hymenaea	MACN-Bar-Lep-ct 00229	LEPAR318-11	BOLD:AAZ4738	MF545663	658[0n]	2010	Misiones 2
Episcada hymenaea	MACN-Bar-Lep-ct 01301	LEPAR883-11	BOLD:AAZ4738	MF547197	658[0n]	2010	Misiones 2
Episcada hymenaea	MACN-Bar-Lep-ct 02044	LEPIG153-11	BOLD:AAZ4738	MF545973	658[0n]	2011	Misiones 2
Episcada hymenaea	MACN-Bar-Lep-ct 02053	LEPIG161-11	BOLD:AAZ4738	MF545819	658[0n]	2011	Misiones 2
Episcada hymenaea	MACN-Bar-Lep-ct 02369	LEPIG439-11	BOLD:AAZ4738	MF546838	658[0n]	2011	Misiones 3
Episcada hymenaea	MACN-Bar-Lep-ct 02575	LEPPA230-12	BOLD:AAZ4738	MF545852	658[0n]	2011	Formosa 2
Episcada hymenaea	MACN-Bar-Lep-ct 02598	LEPPA253-12	BOLD:AAZ4738	MF545610	658[0n]	2011	Formosa 2
Episcada hymenaea	MACN-Bar-Lep-ct 06399	LEPPA987-14			0	2014	Chaco 3
Episcada sylvo	MACN-Bar-Lep-ct 01188	LEPAR810-11	BOLD:AAZ2694	MF547042	658[0n]	2010	Misiones 2
Episcada sylvo	MACN-Bar-Lep-ct 01881	LEPIG009-11	BOLD:AAZ2694	MF545771	658[0n]	2011	Misiones 2
Episcada sylvo	MACN-Bar-Lep-ct 02003	LEPIG120-11	BOLD:AAZ2694	MF546467	658[0n]	2011	Misiones 2
Episcada sylvo	MACN-Bar-Lep-ct 02036	LEPIG146-11	BOLD:AAZ2694	MF547059	658[0n]	2011	Misiones 2
Episcada sylvo	MACN-Bar-Lep-ct 02039	LEPIG148-11	BOLD:AAZ2694	MF546229	658[0n]	2011	Misiones 2
Episcada sylvo	MACN-Bar-Lep-ct 02045	LEPIG154-11	BOLD:AAZ2694	MF547393	658[0n]	2011	Misiones 2
Episcada sylvo	MACN-Bar-Lep-ct 02048	LEPIG157-11	BOLD:AAZ2694	MF546693	658[0n]	2011	Misiones 2
Episcada sylvo	MACN-Bar-Lep-ct 02050	LEPIG158-11	BOLD:AAZ2694	MF545667	658[0n]	2011	Misiones 2
Episcada sylvo	MACN-Bar-Lep-ct 02056	LEPIG163-11	BOLD:AAZ2694	MF546354	658[0n]	2011	Misiones 2
Episcada sylvo	MACN-Bar-Lep-ct 02057	LEPIG164-11	BOLD:AAZ2694	MF546741	658[0n]	2011	Misiones 2
Episcada sylvo	MACN-Bar-Lep-ct 02059	LEPIG166-11	BOLD:AAZ2694	MF545929	658[0n]	2011	Misiones 2
Episcada sylvo	MACN-Bar-Lep-ct 02061	LEPIG168-11	BOLD:AAZ2694	MF546036	658[0n]	2011	Misiones 2
Episcada sylvo	MACN-Bar-Lep-ct 02063	LEPIG170-11	BOLD:AAZ2694	MF546224	658[0n]	2011	Misiones 2
Epityches eupompe	MACN-Bar-Lep-ct 02000	LEPIG117-11	BOLD:AAZ8192	MF546742	658[0n]	2011	Misiones 2
Epityches eupompe	MACN-Bar-Lep-ct 02147	LEPIG243-11	BOLD:AAZ8192	MF546820	658[0n]	2011	Misiones 3
Epityches eupompe	MACN-Bar-Lep-ct 02471	LEPIG514-11	BOLD:AAZ8192	MF546652	658[0n]	2011	Misiones 3
Eresia lansdorfi	MACN-Bar-Lep-ct 00538	LEPAR473-11	BOLD:AAX8366	MF545458	658[0n]	2010	Misiones 2
Eresia lansdorfi	MACN-Bar-Lep-ct 00832	LEPAR607-11	BOLD:AAX8366	MF547048	658[0n]	2010	Misiones 2
Eresia lansdorfi	MACN-Bar-Lep-ct 00974	LEPAR681-11	BOLD:AAX8366	MF545445	658[0n]	2010	Misiones 2
Eresia lansdorfi	MACN-Bar-Lep-ct 00988	LEPAR692-11	BOLD:AAX8366	MF546130	627[0n]	2010	Misiones 2

Eresia lansdorfi	MACN-Bar-Lep-ct 01194	LEPAR816-11	BOLD:AAX8366	MF545793	658[0n]	2010	Misiones 2
Eresia lansdorfi	MACN-Bar-Lep-ct 02148	LEPIG244-11	BOLD:AAX8366	MF545870	658[0n]	2011	Misiones 3
Eresia lansdorfi	MACN-Bar-Lep-ct 02274	LEPIG362-11	BOLD:AAX8366	MF546047	658[0n]	2011	Misiones 3
Eresia lansdorfi	MACN-Bar-Lep-ct 02331	LEPIG412-11	BOLD:AAX8366	MF545875	658[0n]	2011	Misiones 3
Erynnis funeralis	MACN-Bar-Lep-ct 03640	LEPPA746-13	BOLD:AAF2757	MF547081	658[0n]	2011	Corrientes 1
Erynnis funeralis	MACN-Bar-Lep-ct 03833	LEPPA866-13	BOLD:AAF2757	MF545496	618[0n]	2011	Corrientes 1
Eryphanis reevesii	MACN-Bar-Lep-ct 00287	LEPAR351-11	BOLD:AAZ4999	MF546004	658[2n]	2010	Misiones 2
Eteona tisiphone	MACN-Bar-Lep-ct 01388	LEPAR947-11	BOLD:AAX7720	MF547374	658[0n]	2010	Misiones 2
Eueides aliphera	MACN-Bar-Lep-ct 00550	LEPAR480-11	BOLD:AAC5422	MF546889	658[0n]	2010	Misiones 2
Eueides aliphera	MACN-Bar-Lep-ct 00583	LEPAR510-11	BOLD:AAC5422	MF547153	658[0n]	2010	Misiones 2
Eueides aliphera	MACN-Bar-Lep-ct 00590	LEPAR516-11	BOLD:AAC5422	MF545634	658[0n]	2010	Misiones 2
Eueides aliphera	MACN-Bar-Lep-ct 00839	LEPAR613-11	BOLD:AAC5422	MF545553	658[0n]	2010	Misiones 2
Eueides aliphera	MACN-Bar-Lep-ct 01001	LEPAR701-11	BOLD:AAC5422	MF546006	654[0n]	2010	Misiones 2
Eueides aliphera	MACN-Bar-Lep-ct 01947	LEPIG069-11	BOLD:AAC5422	MF545833	658[0n]	2011	Misiones 2
Eueides aliphera	MACN-Bar-Lep-ct 02141	LEPIG238-11	BOLD:AAC5422	MF546686	649[0n]	2011	Misiones 3
Eueides aliphera	MACN-Bar-Lep-ct 02196	LEPIG286-11	BOLD:AAC5422	MF547125	658[0n]	2011	Misiones 3
Eueides aliphera	MACN-Bar-Lep-ct 02603	LEPPA258-12	BOLD:AAC5422	MF545901	658[0n]	2011	Formosa 2
Eueides isabella	MACN-Bar-Lep-ct 01335	LEPAR916-11	BOLD:AAB6623	MF546497	658[0n]	2010	Misiones 2
Eueides isabella	MACN-Bar-Lep-ct 01336	LEPAR917-11	BOLD:AAB6623	MF547280	658[0n]	2010	Misiones 2
Eueides isabella	MACN-Bar-Lep-ct 02520	LEPIG542-11	BOLD:AAB6623	MF545646	658[0n]	2011	Misiones 3
Eunica eburnea	MACN-Bar-Lep-ct 01300	LEPAR882-11	BOLD:AAE4564	MF546540	654[0n]	2010	Misiones 2
Eunica eburnea	MACN-Bar-Lep-ct 01504	LEPAR132-11	BOLD:AAE4564	MF546795	658[0n]	2011	Entre Ríos 4
Eunica margarita	MACN-Bar-Lep-ct 00543	LEPAR476-11	BOLD:ACF1588	MF546799	658[0n]	2010	Misiones 2
Eunica margarita	MACN-Bar-Lep-ct 00625	LEPAR546-11	BOLD:ACF1588	MF546262	658[0n]	2010	Misiones 2
Eunica margarita	MACN-Bar-Lep-ct 01163	LEPAR791-11	BOLD:ACF1588	MF545636	658[0n]	2010	Misiones 2
Eunica tatila	MACN-Bar-Lep-ct 00327	LEPAR375-11	BOLD:AAZ1656	MF546616	658[0n]	2010	Misiones 2
Eunica tatila	MACN-Bar-Lep-ct 00562	LEPAR490-11	BOLD:AAZ1656	MF545953	658[0n]	2010	Misiones 2
Eunica tatila	MACN-Bar-Lep-ct 01128	LEPAR763-11	BOLD:AAZ1656	MF546857	658[0n]	2010	Misiones 2
Euptoieta hegesia	MACN-Bar-Lep-ct 03957	LEPPA579-13			0	2012	Formosa 4
Euptoieta hegesia	MACN-Bar-Lep-ct 06027	LEPAR960-14			0	2013	Formosa 5
Euptoieta hortensia	MACN-Bar-Lep-ct 01516	LEPAR138-11	BOLD:AAZ7915	MF545542	658[0n]	2011	Entre Ríos 4
Euptoieta hortensia	MACN-Bar-Lep-ct 01585	LEPAR163-11	BOLD:AAZ7915	MF545900	658[0n]	2011	Entre Ríos 3
Euptoieta hortensia	MACN-Bar-Lep-ct 01721	LEPAR224-11	BOLD:AAZ7915	MF546383	658[0n]	2011	Entre Ríos 3

Euptoieta hortensia	MACN-Bar-Lep-ct 01771	LEPAR245-11	BOLD:AAZ7915	MF546768	658[0n]	2011	Entre Ríos 6
Euptoieta hortensia	MACN-Bar-Lep-ct 03060	LEPPA173-11	> 1% Ns		658[11n]	2011	Buenos Aires 16
Eurema agave	MACN-Bar-Lep-ct 02615	LEPPA269-12	BOLD:ACA9009	MF547138	658[0n]	2011	Formosa 2
Eurema agave	MACN-Bar-Lep-ct 02630	LEPPA284-12	BOLD:ACA9009	MF546577	658[0n]	2011	Formosa 2
Eurema agave	MACN-Bar-Lep-ct 02685	LEPPA335-12	BOLD:ACA9009	MF546959	658[0n]	2011	Formosa 2
Eurema albula	MACN-Bar-Lep-ct 01978	LEPIG097-11	BOLD:AAB3812	MF545578	658[0n]	2011	Misiones 2
Eurema albula	MACN-Bar-Lep-ct 01997	LEPIG114-11	BOLD:AAB3812	MF545684	658[0n]	2011	Misiones 2
Eurema albula	MACN-Bar-Lep-ct 02662	LEPPA315-12	BOLD:AAB3812	MF545499	658[0n]	2011	Formosa 2
Eurema albula	MACN-Bar-Lep-ct 02690	LEPPA339-12	BOLD:AAB3812	MF546402	658[0n]	2011	Formosa 2
Eurema albula	MACN-Bar-Lep-ct 02820	LEPPA414-12	BOLD:AAB3812	MF547036	658[0n]	2011	Formosa 2
Eurema albula	MACN-Bar-Lep-ct 03638	LEPPA744-13	BOLD:AAB3812	MF545563	658[0n]	2011	Corrientes 1
Eurema albula	MACN-Bar-Lep-ct 03691	LEPPA778-13	BOLD:AAB3812	MF547347	658[0n]	2011	Corrientes 1
Eurema albula	MACN-Bar-Lep-ct 03726	LEPPA802-13	BOLD:AAB3812	MF546678	658[0n]	2011	Corrientes 1
Eurema arbela	MACN-Bar-Lep-ct 02600	LEPPA255-12	BOLD:ACA9123	MF546605	658[0n]	2011	Formosa 2
Eurema arbela	MACN-Bar-Lep-ct 02829	LEPPA418-12	BOLD:ACA9123	MF545799	658[0n]	2011	Formosa 2
Eurema arbela	MACN-Bar-Lep-ct 02836	LEPPA421-12	BOLD:ACA9123	MF545564	658[0n]	2011	Formosa 2
Eurema arbela	MACN-Bar-Lep-ct 02850	LEPPA428-12	BOLD:ACA9123	MF547022	658[0n]	2011	Formosa 2
Eurema arbela	MACN-Bar-Lep-ct 02858	LEPPA433-12	BOLD:ACA9123	MF546183	658[0n]	2011	Formosa 2
Eurema arbela	MACN-Bar-Lep-ct 06109	LEPAR1042-14	BOLD:ACA9123	MF545469	658[0n]	2013	Formosa 5
Eurema deva	MACN-Bar-Lep-ct 00057	LEPAR037-11	BOLD:AAY7965	MF546268	658[0n]	2010	Formosa 1
Eurema deva	MACN-Bar-Lep-ct 00083	LEPAR063-11			0	1991	Córdoba 1
Eurema deva	MACN-Bar-Lep-ct 00084	LEPAR064-11			0	1990	Córdoba 1
Eurema deva	MACN-Bar-Lep-ct 01465	LEPAR107-11	BOLD:AAY7965	MF546059	658[0n]	2011	Entre Ríos 3
Eurema deva	MACN-Bar-Lep-ct 01890	LEPIG018-11	BOLD:AAY7965	MF545704	658[0n]	2011	Misiones 2
Eurema deva	MACN-Bar-Lep-ct 01908	LEPIG035-11	BOLD:AAY7965	MF545470	658[0n]	2011	Misiones 2
Eurema deva	MACN-Bar-Lep-ct 02091	LEPIG194-11	BOLD:AAY7965	MF545876	650[0n]	2011	Misiones 2
Eurema deva	MACN-Bar-Lep-ct 02116	LEPIG214-11	BOLD:AAY7965	MF545725	658[0n]	2011	Misiones 2
Eurema deva	MACN-Bar-Lep-ct 02574	LEPPA229-12	BOLD:AAY7965	MF545417	658[0n]	2011	Formosa 2
Eurema deva	MACN-Bar-Lep-ct 02583	LEPPA238-12	BOLD:AAY7965	MF547394	658[0n]	2011	Formosa 2
Eurema deva	MACN-Bar-Lep-ct 02668	LEPPA321-12	BOLD:AAY7965	MF546622	658[0n]	2011	Formosa 2
Eurema deva	MACN-Bar-Lep-ct 03113	LEPPA036-11	BOLD:AAY7965	MF546353	658[0n]	2010	Buenos Aires 21
Eurema deva	MACN-Bar-Lep-ct 03117	LEPPA040-11			0	2010	Buenos Aires 21
Eurema deva	MACN-Bar-Lep-ct 03150	LEPPA066-11	BOLD:AAY7965	MF546399	658[0n]	2010	Buenos Aires 21

Eurema deva	MACN-Bar-Lep-ct 03699	LEPPA785-13	BOLD:AAY7965	MF547271	658[0n]	2011	Corrientes 1
Eurema deva	MACN-Bar-Lep-ct 03718	LEPPA799-13	BOLD:AAY7965	MF545393	658[0n]	2011	Corrientes 1
Eurema deva	MACN-Bar-Lep-ct 03732	LEPPA807-13	BOLD:AAY7965	MF546888	658[0n]	2011	Corrientes 1
Eurema deva	MACN-Bar-Lep-ct 06047	LEPAR980-14	BOLD:AAY7965	MF545909	658[0n]	2013	Formosa 5
Eurema deva	MACN-Bar-Lep-ct 06079	LEPAR1012-14	BOLD:AAY7965	MF546753	658[4n]	2013	Formosa 5
Eurema deva	MACN-Bar-Lep-ct 06103	LEPAR1036-14	BOLD:AAY7965	MF545478	658[1n]	2013	Formosa 5
Eurema deva	MACN-Bar-Lep-ct 06468	LEPPA1056-14	BOLD:AAY7965	MF545677	658[1n]	2013	Córdoba 4
Eurema deva	MACN-Bar-Lep-ct 06471	LEPPA1059-14			0	2013	Córdoba 4
Eurema deva	MACN-Bar-Lep-ct 06479	LEPPA1067-14			0	2013	Córdoba 4
Eurema elathea	MACN-Bar-Lep-ct 01522	LEPAR141-11	BOLD:AAN7022	MF546694	658[0n]	2011	Entre Ríos 4
Eurema elathea	MACN-Bar-Lep-ct 01527	LEPAR143-11	BOLD:AAN7022	MF547130	658[0n]	2011	Entre Ríos 4
Eurema elathea	MACN-Bar-Lep-ct 01536	LEPAR148-11	BOLD:AAN7022	MF546369	658[0n]	2011	Entre Ríos 4
Eurema elathea	MACN-Bar-Lep-ct 01985	LEPIG104-11	BOLD:AAN7022	MF546188	658[0n]	2011	Misiones 2
Eurema elathea	MACN-Bar-Lep-ct 02013	LEPIG128-11	BOLD:AAN7022	MF547216	658[0n]	2011	Misiones 2
Eurema elathea	MACN-Bar-Lep-ct 02620	LEPPA274-12	BOLD:ACA9010	MF546773	658[0n]	2011	Formosa 2
Eurema elathea	MACN-Bar-Lep-ct 03658	LEPPA757-13	BOLD:AAN7022	MF546715	658[0n]	2011	Corrientes 1
Eurema elathea	MACN-Bar-Lep-ct 03693	LEPPA780-13	BOLD:AAN7022	MF546212	658[0n]	2011	Corrientes 1
Eurema elathea	MACN-Bar-Lep-ct 03697	LEPPA784-13	BOLD:AAN7022	MF545887	658[0n]	2011	Corrientes 1
Eurema elathea	MACN-Bar-Lep-ct 03964	LEPPA585-13	BOLD:AAN7022	MF546314	658[0n]	2012	Formosa 4
Eurema elathea	MACN-Bar-Lep-ct 06026	LEPAR959-14	BOLD:AAN7022	MF546841	658[2n]	2013	Formosa 5
Euryades corethrus	MACN-Bar-Lep-ct 01453	LEPAR101-11	BOLD:AAY2961	MF546866	658[0n]	2011	Entre Ríos 3
Euryades corethrus	MACN-Bar-Lep-ct 01456	LEPAR103-11	BOLD:AAY2961	MF546962	658[0n]	2011	Entre Ríos 3
Euryades corethrus	MACN-Bar-Lep-ct 01640	LEPAR189-11	BOLD:AAY2961	MF546239	658[0n]	2011	Entre Ríos 4
Euryades corethrus	MACN-Bar-Lep-ct 01855	LEPAR275-11	BOLD:AAY2961	MF545801	658[0n]	2011	Entre Ríos 3
Euryades duponchelii	MACN-Bar-Lep-ct 02605	LEPPA260-12	BOLD:AAY2971	MF546898	658[0n]	2011	Formosa 2
Euryades duponchelii	MACN-Bar-Lep-ct 02643	LEPPA296-12	BOLD:AAY2971	MF546657	658[0n]	2011	Formosa 2
Euryades duponchelii	MACN-Bar-Lep-ct 06018	LEPAR951-14	BOLD:AAY2971	MF546818	658[0n]	2013	Formosa 5
Euryades duponchelii	MACN-Bar-Lep-ct 06033	LEPAR966-14	BOLD:AAY2971	MF547170	658[0n]	2013	Formosa 5
Euryades duponchelii	MACN-Bar-Lep-ct 06044	LEPAR977-14	BOLD:AAY2971	MF546411	658[2n]	2013	Formosa 5
Euryades duponchelii	MACN-Bar-Lep-ct 06060	LEPAR993-14	BOLD:AAY2971	MF545483	658[0n]	2013	Formosa 5
Eutocus sp. 1	MACN-Bar-Lep-ct 03799	LEPPA846-13	BOLD:AAA9167	MF545621	658[0n]	2011	Corrientes 1
Eutocus vetulus	MACN-Bar-Lep-ct 02069	LEPIG174-11	BOLD:AAZ9179	MF545507	658[0n]	2011	Misiones 2
Evansiella cordela	MACN-Bar-Lep-ct 01275	LEPAR862-11	BOLD:AAZ5130	MF546258	658[0n]	2010	Misiones 2

Exoplisia myrtis	MACN-Bar-Lep-ct 02636	LEPPA289-12	BOLD:ACA8857	MF545423	658[0n]	2011	Formosa 2
Fountainea cratias	MACN-Bar-Lep-ct 03976	LEPPA592-13	BOLD:ABZ8146	MF546124	658[0n]	2012	Formosa 4
Fountainea ryphea	MACN-Bar-Lep-ct 02396	LEPIG461-11	BOLD:AAB3157	MF546090	658[0n]	2011	Misiones 3
Fountainea ryphea	MACN-Bar-Lep-ct 02538	LEPIG558-11	BOLD:AAB3157	MF547189	658[0n]	2011	Misiones 3
Gesta austerus	MACN-Bar-Lep-ct 01474	LEPAR112-11	BOLD:AAZ7723	MF547115	658[0n]	2011	Entre Ríos 3
Gesta austerus	MACN-Bar-Lep-ct 01510	LEPAR136-11	BOLD:AAZ7723	MF546176	658[0n]	2011	Entre Ríos 4
Gesta austerus	MACN-Bar-Lep-ct 01626	LEPAR182-11	BOLD:AAZ7723	MF546283	658[0n]	2011	Entre Ríos 4
Gesta austerus	MACN-Bar-Lep-ct 01635	LEPAR187-11	BOLD:AAZ7723	MF546604	658[0n]	2011	Entre Ríos 4
Gesta austerus	MACN-Bar-Lep-ct 01639	LEPAR188-11	BOLD:AAZ7723	MF545520	658[0n]	2011	Entre Ríos 4
Gesta austerus	MACN-Bar-Lep-ct 03842	LEPPA869-13	BOLD:AAZ7723	MF545430	658[0n]	2011	Corrientes 2
Gesta austerus	MACN-Bar-Lep-ct 03846	LEPPA872-13	BOLD:AAZ7723	MF547356	658[0n]	2011	Corrientes 2
Gesta austerus	MACN-Bar-Lep-ct 03848	LEPPA874-13	BOLD:AAZ7723	MF546469	658[0n]	2011	Corrientes 2
Gesta austerus	MACN-Bar-Lep-ct 03962	LEPPA583-13	BOLD:AAZ7723	MF546736	658[0n]	2012	Formosa 4
Gesta gesta	MACN-Bar-Lep-ct 03625	LEPPA737-13	BOLD:ACG1302	MF546854	658[0n]	2011	Corrientes 1
Gesta gesta	MACN-Bar-Lep-ct 03688	LEPPA775-13	BOLD:ACG1302	MF545802	658[0n]	2011	Corrientes 1
Gesta gesta	MACN-Bar-Lep-ct 03850	LEPPA876-13	BOLD:ACG1302	MF546419	658[0n]	2011	Corrientes 2
Glutophrissa drusilla	MACN-Bar-Lep-ct 02677	LEPPA328-12	BOLD:AAB8730	MF546993	658[0n]	2011	Formosa 2
Glutophrissa drusilla	MACN-Bar-Lep-ct 02698	LEPPA343-12	BOLD:AAB8730	MF547088	654[0n]	2011	Formosa 2
Godartiana muscosa	MACN-Bar-Lep-ct 00244	LEPAR330-11	BOLD:AAZ2611	MF546804	658[0n]	2010	Misiones 2
Godartiana muscosa	MACN-Bar-Lep-ct 00800	LEPAR580-11	BOLD:AAY0914	MF546414	658[0n]	2010	Misiones 2
Godartiana muscosa	MACN-Bar-Lep-ct 00850	LEPAR623-11	BOLD:AAY0914	MF546520	658[0n]	2010	Misiones 2
Godartiana muscosa	MACN-Bar-Lep-ct 01140	LEPAR774-11	BOLD:AAZ2611	MF545707	658[0n]	2010	Misiones 2
Godartiana muscosa	MACN-Bar-Lep-ct 01941	LEPIG063-11	BOLD:AAY0914	MF547334	658[0n]	2011	Misiones 2
Godartiana muscosa	MACN-Bar-Lep-ct 02096	LEPIG199-11	BOLD:AAY0914	MF547228	658[0n]	2011	Misiones 2
Godartiana muscosa	MACN-Bar-Lep-ct 02261	LEPIG349-11	BOLD:AAY0914	MF547316	658[0n]	2011	Misiones 3
Gorgythion begga	MACN-Bar-Lep-ct 01322	LEPAR903-11	BOLD:AAZ4090	MF546341	658[0n]	2010	Misiones 2
Gorgythion begga	MACN-Bar-Lep-ct 01486	LEPAR121-11	BOLD:AAZ7560	MF546095	658[0n]	2011	Entre Ríos 3
Gorgythion begga	MACN-Bar-Lep-ct 01723	LEPAR226-11	BOLD:AAZ7560	MF545509	658[0n]	2011	Entre Ríos 3
Gorgythion begga	MACN-Bar-Lep-ct 02010	LEPIG125-11	BOLD:AAZ4090	MF545641	658[0n]	2011	Misiones 2
Gorgythion begga	MACN-Bar-Lep-ct 02021	LEPIG134-11	BOLD:AAZ4090	MF547019	658[0n]	2011	Misiones 2
Gorgythion begga	MACN-Bar-Lep-ct 02027	LEPIG139-11	BOLD:AAZ4090	MF545854	658[0n]	2011	Misiones 2
Gorgythion begga	MACN-Bar-Lep-ct 02546	LEPIG564-11	BOLD:AAZ4090	MF546972	658[0n]	2011	Misiones 3
Gorgythion beggina	MACN-Bar-Lep-ct 02596	LEPPA251-12	BOLD:ACA9069	MF546044	658[0n]	2011	Formosa 2

Gorgythion beggina	MACN-Bar-Lep-ct 02730	LEPPA366-12	BOLD:ACA9069	MF546076	658[0n]	2011	Formosa 3
Gorgythion beggina	MACN-Bar-Lep-ct 03694	LEPPA781-13	BOLD:ACA9069	MF545836	658[0n]	2011	Corrientes 1
Gorgythion beggina	MACN-Bar-Lep-ct 03709	LEPPA793-13	BOLD:ACA9069	MF545933	658[0n]	2011	Corrientes 1
Gorgythion beggina	MACN-Bar-Lep-ct 03794	LEPPA843-13	BOLD:ACA9069	MF546190	618[0n]	2011	Corrientes 1
Grais stigmaticus	MACN-Bar-Lep-ct 01913	LEPIG039-11	BOLD:AAI5452	MF546265	658[0n]	2011	Misiones 2
Haematera pyrame	MACN-Bar-Lep-ct 00363	LEPAR398-11	BOLD:AAE6767	MF547040	658[0n]	2010	Misiones 2
Haematera pyrame	MACN-Bar-Lep-ct 00364	LEPAR399-11	BOLD:AAE6767	MF546306	658[0n]	2010	Misiones 2
Haematera pyrame	MACN-Bar-Lep-ct 00409	LEPAR423-11	BOLD:AAE6767	MF546483	658[0n]	2010	Misiones 2
Haematera pyrame	MACN-Bar-Lep-ct 00847	LEPAR620-11	BOLD:AAE6767	MF545908	658[0n]	2010	Misiones 2
Haematera pyrame	MACN-Bar-Lep-ct 01169	LEPAR795-11	BOLD:AAE6767	MF545609	642[0n]	2010	Misiones 2
Haematera pyrame	MACN-Bar-Lep-ct 02092	LEPIG195-11	BOLD:AAE6767	MF547255	658[0n]	2011	Misiones 2
Haematera pyrame	MACN-Bar-Lep-ct 02099	LEPIG202-11	BOLD:AAE6767	MF545826	658[0n]	2011	Misiones 2
Haematera pyrame	MACN-Bar-Lep-ct 02111	LEPIG211-11	BOLD:AAE6767	MF545913	658[0n]	2011	Misiones 2
Haematera pyrame	MACN-Bar-Lep-ct 02182	LEPIG276-11	BOLD:AAE6767	MF546608	658[0n]	2011	Misiones 3
Hamadryas amphinome	MACN-Bar-Lep-ct 01876	LEPIG004-11	BOLD:ACF1134	MF546147	658[0n]	2011	Misiones 2
Hamadryas amphinome	MACN-Bar-Lep-ct 01986	LEPIG105-11	BOLD:ACE4966	MF546368	658[0n]	2011	Misiones 2
Hamadryas amphinome	MACN-Bar-Lep-ct 02278	LEPIG365-11	BOLD:ACF1134	MF546305	658[0n]	2011	Misiones 3
Hamadryas amphinome	MACN-Bar-Lep-ct 02325	LEPIG406-11	BOLD:ACE4966	MF546421	658[0n]	2011	Misiones 3
Hamadryas amphinome	MACN-Bar-Lep-ct 02431	LEPIG490-11	BOLD:ACF1134	MF546055	658[0n]	2011	Misiones 3
Hamadryas epinome	MACN-Bar-Lep-ct 00296	LEPAR357-11	BOLD:AAI6852	MF546175	658[0n]	2010	Misiones 2
Hamadryas epinome	MACN-Bar-Lep-ct 00801	LEPAR581-11	BOLD:AAI6852	MF545930	658[0n]	2010	Misiones 2
Hamadryas epinome	MACN-Bar-Lep-ct 00806	LEPAR584-11	BOLD:AAI6852	MF546732	658[0n]	2010	Misiones 2
Hamadryas epinome	MACN-Bar-Lep-ct 01005	LEPAR705-11	BOLD:AAI6852	MF545436	658[0n]	2010	Misiones 2
Hamadryas epinome	MACN-Bar-Lep-ct 01389	LEPAR948-11	BOLD:AAI6852	MF546083	658[0n]	2010	Misiones 2
Hamadryas epinome	MACN-Bar-Lep-ct 01895	LEPIG023-11	BOLD:AAI6852	MF546336	658[0n]	2011	Misiones 2
Hamadryas epinome	MACN-Bar-Lep-ct 01965	LEPIG086-11	BOLD:AAI6852	MF545664	658[0n]	2011	Misiones 2
Hamadryas epinome	MACN-Bar-Lep-ct 02179	LEPIG273-11	BOLD:AAI6852	MF546142	658[0n]	2011	Misiones 3
Hamadryas epinome	MACN-Bar-Lep-ct 02651	LEPPA304-12	BOLD:AAI6852	MF546133	658[0n]	2011	Formosa 2
Hamadryas februa	MACN-Bar-Lep-ct 01935	LEPIG058-11	BOLD:ABZ5790	MF546538	658[0n]	2011	Misiones 2
Hamadryas februa	MACN-Bar-Lep-ct 01998	LEPIG115-11	BOLD:ABZ5790	MF546148	658[0n]	2011	Misiones 2
Hamadryas februa	MACN-Bar-Lep-ct 02145	LEPIG241-11	BOLD:ABZ5790	MF545500	658[0n]	2011	Misiones 3
Hamadryas februa	MACN-Bar-Lep-ct 02153	LEPIG249-11	BOLD:ABZ5790	MF546971	658[0n]	2011	Misiones 3
Hamadryas februa	MACN-Bar-Lep-ct 02165	LEPIG260-11	BOLD:ABZ5790	MF546202	658[0n]	2011	Misiones 3

*Hamadryas feronia**	MACN-Bar-Lep-ct 02486	LEPIG524-11	NONE	MF546177	658[8n]	2011	Misiones 3
Hamadryas fornax	MACN-Bar-Lep-ct 02245	LEPIG333-11	BOLD:AAK5320	MF547217	658[0n]	2011	Misiones 3
Hamadryas fornax	MACN-Bar-Lep-ct 02315	LEPIG399-11	BOLD:AAK5320	MF547383	658[0n]	2011	Misiones 3
Hamadryas fornax	MACN-Bar-Lep-ct 02402	LEPIG467-11	BOLD:AAK5320	MF546792	658[0n]	2011	Misiones 3
Hamadryas fornax	MACN-Bar-Lep-ct 02467	LEPIG511-11	BOLD:AAK5320	MF546780	658[0n]	2011	Misiones 3
Harveyope tinea	MACN-Bar-Lep-ct 01482	LEPAR118-11	BOLD:AAZ8215	MF547147	658[0n]	2011	Entre Ríos 3
Harveyope tinea	MACN-Bar-Lep-ct 01683	LEPAR211-11	BOLD:AAZ8215	MF547157	658[0n]	2011	Entre Ríos 3
Harveyope tinea	MACN-Bar-Lep-ct 01746	LEPAR232-11	BOLD:AAZ8215	MF546196	658[0n]	2011	Entre Ríos 3
Haywardella edmondsii	MACN-Bar-Lep-ct 00076	LEPAR056-11	BOLD:AAX1942	MF545488	658[0n]	2010	Córdoba 1
Haywardella edmondsii	MACN-Bar-Lep-ct 03093	LEPPA016-11	BOLD:AAX1942	MF545490	658[0n]	2011	Córdoba 2
Haywardella edmondsii	MACN-Bar-Lep-ct 03095	LEPPA018-11			0	2011	Córdoba 2
Haywardella edmondsii	MACN-Bar-Lep-ct 03097	LEPPA020-11	BOLD:AAX1942	MF546860	658[0n]	2011	Córdoba 2
Helias phalaenoides	MACN-Bar-Lep-ct 00872	LEPAR639-11	BOLD:AAZ1307	MF545751	658[0n]	2010	Misiones 2
Helias phalaenoides	MACN-Bar-Lep-ct 01056	LEPAR749-11	BOLD:AAZ1307	MF546575	658[0n]	2010	Misiones 2
Helias phalaenoides	MACN-Bar-Lep-ct 02335	LEPIG416-11	BOLD:AAZ1307	MF546077	658[0n]	2011	Misiones 3
Helias phalaenoides	MACN-Bar-Lep-ct 02388	LEPIG454-11	BOLD:AAZ1307	MF546787	658[0n]	2011	Misiones 3
Heliconius besckei	MACN-Bar-Lep-ct 01980	LEPIG099-11	BOLD:AAZ9926	MF546173	658[0n]	2011	Misiones 2
Heliconius erato	MACN-Bar-Lep-ct 00158	LEPAR287-11	BOLD:AAY9571	MF545613	658[0n]	2010	Misiones 2
Heliconius erato	MACN-Bar-Lep-ct 00225	LEPAR314-11	BOLD:AAZ3773	MF546167	658[0n]	2010	Misiones 2
Heliconius erato	MACN-Bar-Lep-ct 00285	LEPAR349-11	BOLD:AAZ3773	MF546292	658[0n]	2010	Misiones 2
Heliconius erato	MACN-Bar-Lep-ct 00973	LEPAR680-11	BOLD:AAY9571	MF545633	658[0n]	2010	Misiones 2
Heliconius erato	MACN-Bar-Lep-ct 01307	LEPAR889-11	BOLD:AAZ3773	MF547208	658[0n]	2010	Misiones 2
Heliconius erato	MACN-Bar-Lep-ct 01807	LEPAR248-11	BOLD:AAZ3773	MF547126	658[0n]	2011	Entre Ríos 3
Heliconius erato	MACN-Bar-Lep-ct 01813	LEPAR253-11	BOLD:AAZ3773	MF547021	658[0n]	2011	Entre Ríos 3
Heliconius erato	MACN-Bar-Lep-ct 01844	LEPAR268-11	BOLD:AAZ3773	MF546230	658[0n]	2011	Entre Ríos 3
Heliconius erato	MACN-Bar-Lep-ct 01923	LEPIG047-11	BOLD:AAZ3773	MF547256	658[0n]	2011	Misiones 2
Heliconius erato	MACN-Bar-Lep-ct 01964	LEPIG085-11	BOLD:AAZ3773	MF546211	658[0n]	2011	Misiones 2
Heliconius erato	MACN-Bar-Lep-ct 02105	LEPIG207-11	BOLD:AAZ3773	MF546216	658[0n]	2011	Misiones 2
Heliconius erato	MACN-Bar-Lep-ct 02664	LEPPA317-12	BOLD:AAZ3773	MF546897	658[0n]	2011	Formosa 2
Heliconius erato	MACN-Bar-Lep-ct 02706	LEPPA349-12	BOLD:AAZ3773	MF545730	658[0n]	2011	Formosa 3
Heliconius erato	MACN-Bar-Lep-ct 02716	LEPPA358-12	BOLD:AAZ3773	MF545637	658[0n]	2011	Formosa 3
Heliconius erato	MACN-Bar-Lep-ct 03677	LEPPA767-13	BOLD:AAZ3773	MF546445	658[0n]	2011	Corrientes 1
Heliconius erato	MACN-Bar-Lep-ct 03690	LEPPA777-13	BOLD:AAZ3773	MF546746	658[0n]	2011	Corrientes 1

Heliconius ethilla	MACN-Bar-Lep-ct 02404	LEPIG469-11	BOLD:ABY5743	MF547159	658[0n]	2011	Misiones 3
Heliconius ethilla	MACN-Bar-Lep-ct 07139	LEPAR1217-15			0	2007	Misiones 11
Heliconius ethilla	MACN-Bar-Lep-ct 07141	LEPAR1219-15			0	2002	Misiones 11
Heliopetes alana	MACN-Bar-Lep-ct 00350	LEPAR391-11	BOLD:ABZ6309	MF546701	658[0n]	2010	Misiones 2
Heliopetes alana	MACN-Bar-Lep-ct 00371	LEPAR403-11	BOLD:ABZ6309	MF545466	658[0n]	2010	Misiones 2
Heliopetes alana	MACN-Bar-Lep-ct 00615	LEPAR537-11	BOLD:ABZ6309	MF547024	658[0n]	2010	Misiones 2
Heliopetes alana	MACN-Bar-Lep-ct 00820	LEPAR597-11	BOLD:ABZ6309	MF545449	658[0n]	2010	Misiones 2
Heliopetes alana	MACN-Bar-Lep-ct 01041	LEPAR734-11	BOLD:ABZ6309	MF546702	658[0n]	2010	Misiones 2
Heliopetes alana	MACN-Bar-Lep-ct 01970	LEPIG090-11	BOLD:ABZ6309	MF546475	658[0n]	2011	Misiones 2
Heliopetes alana	MACN-Bar-Lep-ct 02166	LEPIG261-11	BOLD:ABZ6309	MF545767	658[0n]	2011	Misiones 3
Heliopetes alana	MACN-Bar-Lep-ct 02298	LEPIG383-11	BOLD:ABZ6309	MF547180	658[0n]	2011	Misiones 3
Heliopetes alana	MACN-Bar-Lep-ct 02306	LEPIG390-11	BOLD:ABZ6309	MF546198	658[0n]	2011	Misiones 3
Heliopetes alana	MACN-Bar-Lep-ct 02410	LEPIG475-11	BOLD:ABZ6309	MF546294	658[0n]	2011	Misiones 3
Heliopetes arsalte	MACN-Bar-Lep-ct 00182	LEPAR300-11	BOLD:ACE7070	MF546322	658[0n]	2010	Misiones 2
Heliopetes arsalte	MACN-Bar-Lep-ct 01507	LEPAR134-11	BOLD:ACE7070	MF546549	658[0n]	2011	Entre Ríos 4
Heliopetes arsalte	MACN-Bar-Lep-ct 01645	LEPAR191-11	BOLD:ACE7070	MF546156	658[0n]	2011	Entre Ríos 4
Heliopetes arsalte	MACN-Bar-Lep-ct 01957	LEPIG079-11	BOLD:ACE7070	MF545822	658[0n]	2011	Misiones 2
Heliopetes arsalte	MACN-Bar-Lep-ct 02625	LEPPA279-12	BOLD:ACE7070	MF546380	658[0n]	2011	Formosa 2
Heliopetes arsalte	MACN-Bar-Lep-ct 02650	LEPPA303-12	BOLD:ACE7070	MF546624	658[0n]	2011	Formosa 2
Heliopetes arsalte	MACN-Bar-Lep-ct 02717	LEPPA359-12	BOLD:ACE7070	MF545517	658[0n]	2011	Formosa 3
Heliopetes arsalte	MACN-Bar-Lep-ct 03745	LEPPA817-13	BOLD:ACE7070	MF546201	658[0n]	2011	Corrientes 1
Heliopetes arsalte	MACN-Bar-Lep-ct 03812	LEPPA854-13	BOLD:ACE7070	MF547095	658[0n]	2011	Corrientes 1
Heliopetes arsalte	MACN-Bar-Lep-ct 06030	LEPAR963-14	BOLD:ACE7070	MF547009	658[0n]	2013	Formosa 5
Heliopetes libra	MACN-Bar-Lep-ct 01028	LEPAR725-11	BOLD:AAZ1321	MF547065	658[0n]	2010	Misiones 2
Heliopetes libra	MACN-Bar-Lep-ct 01354	LEPAR935-11	BOLD:AAZ1321	MF546733	658[0n]	2010	Misiones 2
Heliopetes libra	MACN-Bar-Lep-ct 02384	LEPIG450-11	BOLD:AAZ1321	MF545691	658[0n]	2011	Misiones 3
Heliopetes libra	MACN-Bar-Lep-ct 02515	LEPIG539-11	BOLD:AAZ1321	MF546157	658[0n]	2011	Misiones 3
Heliopetes ochroleuca	MACN-Bar-Lep-ct 00424	LEPAR436-11	BOLD:AAZ1320	MF546060	658[0n]	2010	Misiones 2
Heliopetes ochroleuca	MACN-Bar-Lep-ct 00783	LEPAR567-11	BOLD:AAZ1320	MF545527	658[0n]	2010	Misiones 2
Heliopetes ochroleuca	MACN-Bar-Lep-ct 01042	LEPAR735-11	BOLD:AAZ1320	MF547375	658[0n]	2010	Misiones 2
Heliopetes omrina	MACN-Bar-Lep-ct 00349	LEPAR390-11	BOLD:AAZ6369	MF545516	658[0n]	2010	Misiones 2
Heliopetes omrina	MACN-Bar-Lep-ct 01597	LEPAR169-11	BOLD:AAZ6369	MF546186	658[0n]	2011	Entre Ríos 3
Heliopetes omrina	MACN-Bar-Lep-ct 01614	LEPAR176-11	BOLD:ACE5568	MF545832	658[0n]	2011	Entre Ríos 5

Heliopetes omrina	MACN-Bar-Lep-ct 01667	LEPAR203-11	BOLD:AAZ6369	MF545660	658[0n]	2011	Entre Ríos 4
Heliopetes omrina	MACN-Bar-Lep-ct 01883	LEPIG011-11	BOLD:AAZ6369	MF546530	658[0n]	2011	Misiones 2
Heliopetes omrina	MACN-Bar-Lep-ct 01929	LEPIG053-11	BOLD:AAZ6369	MF546494	658[0n]	2011	Misiones 2
Heliopetes omrina	MACN-Bar-Lep-ct 01959	LEPIG081-11	BOLD:AAZ6369	MF546672	658[0n]	2011	Misiones 2
Heliopetes omrina	MACN-Bar-Lep-ct 01973	LEPIG092-11	BOLD:AAZ6369	MF547148	658[0n]	2011	Misiones 2
Heliopetes omrina	MACN-Bar-Lep-ct 02005	LEPIG122-11	BOLD:AAZ6369	MF547361	658[0n]	2011	Misiones 2
Heliopetes omrina	MACN-Bar-Lep-ct 02710	LEPPA352-12	BOLD:AAZ6369	MF546856	658[0n]	2011	Formosa 3
Heliopetes omrina	MACN-Bar-Lep-ct 02729	LEPPA365-12	BOLD:AAZ6369	MF546088	658[0n]	2011	Formosa 3
Heliopetes omrina	MACN-Bar-Lep-ct 02745	LEPPA377-12	BOLD:ACE5568	MF545750	658[0n]	2011	Formosa 3
Heliopetes omrina	MACN-Bar-Lep-ct 03134	LEPPA055-11	BOLD:ACE5568	MF546920	658[0n]	2011	Buenos Aires 21
Heliopetes omrina	MACN-Bar-Lep-ct 03155	LEPPA071-11	BOLD:AAZ6369	MF546777	658[0n]	2011	Buenos Aires 21
Heliopetes omrina	MACN-Bar-Lep-ct 03578	LEPPA708-13	BOLD:AAZ6369	MF546241	621[0n]	2011	Corrientes 1
Heliopetes omrina	MACN-Bar-Lep-ct 03730	LEPPA805-13	BOLD:AAZ6369	MF547299	658[0n]	2011	Corrientes 1
Heliopetes omrina	MACN-Bar-Lep-ct 03748	LEPPA820-13	BOLD:AAZ6369	MF545993	658[0n]	2011	Corrientes 1
Heliopetes omrina	MACN-Bar-Lep-ct 03915	LEPPA544-13	BOLD:AAZ6369	MF545970	658[0n]	2012	Formosa 4
Heliopetes omrina	MACN-Bar-Lep-ct 03947	LEPPA570-13	BOLD:AAZ6369	MF546296	619[0n]	2012	Formosa 4
Heliopetes omrina	MACN-Bar-Lep-ct 03963	LEPPA584-13	BOLD:AAZ6369	MF545979	583[0n]	2012	Formosa 4
Heliopyrgus americanus	MACN-Bar-Lep-ct 03058	LEPPA171-11	BOLD:ACG1738	MF546029	658[0n]	2011	Buenos Aires 6
Heliopyrgus americanus	MACN-Bar-Lep-ct 03066	LEPPA179-11			0	2011	Buenos Aires 6
Heliopyrgus americanus	MACN-Bar-Lep-ct 03075	LEPPA188-11	BOLD:ACG1738	MF546425	658[0n]	2011	Buenos Aires 6
Heliopyrgus domicella	MACN-Bar-Lep-ct 01662	LEPAR200-11	BOLD:AAZ7637	MF545678	658[0n]	2011	Entre Ríos 4
Heliopyrgus domicella	MACN-Bar-Lep-ct 02712	LEPPA354-12	BOLD:AAZ7637	MF546653	658[0n]	2011	Formosa 3
Heliopyrgus domicella	MACN-Bar-Lep-ct 06081	LEPAR1014-14	BOLD:AAZ7637	MF545440	658[0n]	2013	Formosa 5
Heliopyrgus domicella	MACN-Bar-Lep-ct 06083	LEPAR1016-14	BOLD:AAZ7637	MF545418	658[4n]	2013	Formosa 5
Heliopyrgus domicella	MACN-Bar-Lep-ct 06091	LEPAR1024-14	BOLD:AAZ7637	MF547056	658[0n]	2013	Formosa 5
Hemiargus hanno	MACN-Bar-Lep-ct 02638	LEPPA291-12	BOLD:AAU3608	MF546708	658[0n]	2011	Formosa 2
Hemiargus hanno	MACN-Bar-Lep-ct 02654	LEPPA307-12	BOLD:AAU3608	MF546774	658[0n]	2011	Formosa 2
Hemiargus hanno	MACN-Bar-Lep-ct 02701	LEPPA346-12	BOLD:AAU3608	MF546586	658[0n]	2011	Formosa 2
Hemiargus hanno	MACN-Bar-Lep-ct 03587	LEPPA712-13	BOLD:AAU3608	MF547262	658[0n]	2011	Corrientes 1
Hemiargus hanno	MACN-Bar-Lep-ct 03599	LEPPA723-13	BOLD:AAU3608	MF547107	658[0n]	2011	Corrientes 1
Hemiargus hanno	MACN-Bar-Lep-ct 03639	LEPPA745-13	BOLD:AAU3608	MF546942	658[0n]	2011	Corrientes 1
Hemiargus hanno	MACN-Bar-Lep-ct 03870	LEPPA514-13	BOLD:AAU3608	MF546309	658[0n]	2012	Formosa 4
Hemiargus hanno	MACN-Bar-Lep-ct 03890	LEPPA528-13	BOLD:AAU3608	MF545570	658[0n]	2012	Formosa 4

Hemiargus hanno	MACN-Bar-Lep-ct 03901	LEPPA535-13	BOLD:AAU3608	MF545602	658[0n]	2012	Formosa 4
Heraclides anchisiades	MACN-Bar-Lep-ct 00584	LEPAR511-11	BOLD:AAB6499	MF546939	658[0n]	2010	Misiones 2
Heraclides anchisiades	MACN-Bar-Lep-ct 00970	LEPAR677-11	BOLD:AAB6499	MF547082	658[0n]	2010	Misiones 2
Heraclides anchisiades	MACN-Bar-Lep-ct 01197	LEPAR818-11	BOLD:AAB6499	MF546600	658[0n]	2010	Misiones 2
Heraclides anchisiades	MACN-Bar-Lep-ct 02408	LEPIG473-11	BOLD:AAB6499	MF545640	658[0n]	2011	Misiones 3
Heraclides androgeus	MACN-Bar-Lep-ct 00281	LEPAR345-11	BOLD:AAD0536	MF546492	658[0n]	2010	Misiones 2
Heraclides androgeus	MACN-Bar-Lep-ct 00527	LEPAR464-11	BOLD:AAD0536	MF546561	658[0n]	2010	Misiones 2
Heraclides androgeus	MACN-Bar-Lep-ct 00577	LEPAR505-11	BOLD:AAD0536	MF547358	658[0n]	2010	Misiones 2
Heraclides androgeus	MACN-Bar-Lep-ct 00795	LEPAR576-11	BOLD:AAD0536	MF547028	658[0n]	2010	Misiones 2
Heraclides androgeus	MACN-Bar-Lep-ct 00964	LEPAR672-11	BOLD:AAD0536	MF546948	658[0n]	2010	Misiones 2
Heraclides androgeus	MACN-Bar-Lep-ct 02485	LEPIG523-11	BOLD:AAD0536	MF547086	658[0n]	2011	Misiones 3
Heraclides astyalus	MACN-Bar-Lep-ct 00277	LEPAR343-11	BOLD:AAX2583	MF545699	658[0n]	2010	Misiones 2
Heraclides astyalus	MACN-Bar-Lep-ct 00279	LEPAR949-11	BOLD:AAX2583	MF546080	658[0n]	2010	Misiones 2
Heraclides astyalus	MACN-Bar-Lep-ct 00280	LEPAR950-11	BOLD:AAX2583	MF546249	658[0n]	2010	Misiones 2
Heraclides astyalus	MACN-Bar-Lep-ct 00315	LEPAR367-11	BOLD:AAX2583	MF547145	658[0n]	2010	Misiones 2
Heraclides astyalus	MACN-Bar-Lep-ct 00810	LEPAR587-11	BOLD:AAX2583	MF545484	658[0n]	2010	Misiones 2
Heraclides astyalus	MACN-Bar-Lep-ct 02146	LEPIG242-11	BOLD:AAX2583	MF545413	658[0n]	2011	Misiones 3
Heraclides astyalus	MACN-Bar-Lep-ct 02394	LEPIG459-11	BOLD:AAX2583	MF545891	658[0n]	2011	Misiones 3
Heraclides astyalus	MACN-Bar-Lep-ct 02466	LEPIG510-11	BOLD:AAX2583	MF546623	649[0n]	2011	Misiones 3
Heraclides astyalus	MACN-Bar-Lep-ct 02478	LEPIG517-11	BOLD:AAX2583	MF546582	658[0n]	2011	Misiones 3
Heraclides hectorides	MACN-Bar-Lep-ct 00235	LEPAR322-11	BOLD:AAX2760	MF545700	658[0n]	2010	Misiones 2
Heraclides hectorides	MACN-Bar-Lep-ct 00322	LEPAR371-11	BOLD:AAX2760	MF545719	658[0n]	2010	Misiones 2
Heraclides hectorides	MACN-Bar-Lep-ct 00817	LEPAR594-11	BOLD:AAX2760	MF547318	658[0n]	2010	Misiones 2
Heraclides hectorides	MACN-Bar-Lep-ct 01904	LEPIG032-11	BOLD:AAX2760	MF547341	658[0n]	2011	Misiones 2
Heraclides hectorides	MACN-Bar-Lep-ct 01915	LEPIG041-11	BOLD:AAX2760	MF547116	658[0n]	2011	Misiones 2
Heraclides hectorides	MACN-Bar-Lep-ct 01927	LEPIG051-11	BOLD:AAX2760	MF546895	658[0n]	2011	Misiones 2
Heraclides hectorides	MACN-Bar-Lep-ct 01962	LEPIG083-11	BOLD:AAX2760	MF545952	658[0n]	2011	Misiones 2
Heraclides hectorides	MACN-Bar-Lep-ct 02218	LEPIG307-11	BOLD:AAX2760	MF547004	658[0n]	2011	Misiones 3
Heraclides thoas	MACN-Bar-Lep-ct 00264	LEPAR337-11	BOLD:AAA8521	MF547043	658[0n]	2010	Misiones 2
Heraclides thoas	MACN-Bar-Lep-ct 00565	LEPAR493-11	BOLD:AAA8521	MF545583	658[0n]	2010	Misiones 2
Heraclides thoas	MACN-Bar-Lep-ct 00804	LEPAR583-11	BOLD:AAA8521	MF545727	658[0n]	2010	Misiones 2
Heraclides thoas	MACN-Bar-Lep-ct 00965	LEPAR673-11	BOLD:AAA8521	MF546794	644[0n]	2010	Misiones 2
Heraclides thoas	MACN-Bar-Lep-ct 02139	LEPIG236-11	BOLD:AAA8521	MF546997	658[0n]	2011	Misiones 3

Heraclides thoas	MACN-Bar-Lep-ct 02527	LEPIG549-11	BOLD:AAA8521	MF546458	658[0n]	2011	Misiones 3
Heraclides thoas	MACN-Bar-Lep-ct 03583	LEPPA710-13	BOLD:AAA8521	MF546085	658[0n]	2011	Corrientes 1
Heraclides thoas	MACN-Bar-Lep-ct 03654	LEPPA753-13	BOLD:AAA8521	MF545615	658[0n]	2011	Corrientes 1
Heraclides thoas	MACN-Bar-Lep-ct 03773	LEPPA831-13	BOLD:AAA8521	MF546673	603[0n]	2011	Corrientes 1
Heraclides thoas	MACN-Bar-Lep-ct 06024	LEPAR957-14	BOLD:AAA8521	MF547348	658[4n]	2013	Formosa 5
Heraclides thoas	MACN-Bar-Lep-ct 07065	LEPAR1143-15	BOLD:AAA8521	MF545774	658[0n]	2012	Buenos Aires 24
Hermeuptychia gisella	MACN-Bar-Lep-ct 00374	LEPAR405-11	BOLD:ACX0826	MF547037	658[0n]	2010	Misiones 2
Hermeuptychia gisella	MACN-Bar-Lep-ct 00375	LEPAR406-11	BOLD:ACX0826	MF546370	658[0n]	2010	Misiones 2
Hermeuptychia gisella	MACN-Bar-Lep-ct 01023	LEPAR720-11	BOLD:ACX0826	MF547162	658[0n]	2010	Misiones 2
Hermeuptychia gisella	MACN-Bar-Lep-ct 03670	LEPPA763-13	BOLD:ACX0826	MF546851	658[0n]	2011	Corrientes 1
Hermeuptychia isabella	MACN-Bar-Lep-ct 00017	LEPAR011-11	BOLD:ACG1578	MF546547	658[0n]	2010	Buenos Aires 21
Hermeuptychia isabella	MACN-Bar-Lep-ct 00022	LEPAR012-11	BOLD:ACG1578	MF546442	658[0n]	2010	Buenos Aires 21
Hermeuptychia isabella	MACN-Bar-Lep-ct 00023	LEPAR013-11	BOLD:ACG1578	MF545475	658[0n]	2010	Buenos Aires 21
Hermeuptychia isabella	MACN-Bar-Lep-ct 00024	LEPAR014-11	BOLD:ACG1578	MF546852	658[0n]	2010	Buenos Aires 21
Hermeuptychia isabella	MACN-Bar-Lep-ct 00398	LEPAR417-11	BOLD:ACG1578	MF547013	658[0n]	2010	Misiones 2
Hermeuptychia isabella	MACN-Bar-Lep-ct 01027	LEPAR724-11	BOLD:ACG1578	MF545630	658[0n]	2010	Misiones 2
Hermeuptychia isabella	MACN-Bar-Lep-ct 01475	LEPAR113-11	BOLD:ACG1578	MF545465	658[0n]	2011	Entre Ríos 3
Hermeuptychia isabella	MACN-Bar-Lep-ct 01608	LEPAR172-11	BOLD:ACG1578	MF545543	658[0n]	2011	Entre Ríos 5
Hermeuptychia isabella	MACN-Bar-Lep-ct 01755	LEPAR236-11	BOLD:ACG1578	MF547302	658[0n]	2011	Entre Ríos 6
Hermeuptychia isabella	MACN-Bar-Lep-ct 01999	LEPIG116-11	BOLD:ACG1578	MF546757	658[0n]	2011	Misiones 2
Hermeuptychia isabella	MACN-Bar-Lep-ct 02117	LEPIG215-11	BOLD:ACG1578	MF546002	658[0n]	2011	Misiones 2
Hermeuptychia isabella	MACN-Bar-Lep-ct 02400	LEPIG465-11	BOLD:ACG1578	MF546162	658[0n]	2011	Misiones 3
Hermeuptychia isabella	MACN-Bar-Lep-ct 02681	LEPPA331-12	BOLD:ACG1578	MF545706	658[0n]	2011	Formosa 2
Hermeuptychia isabella	MACN-Bar-Lep-ct 02752	LEPPA381-12	BOLD:ACG1578	MF547174	658[0n]	2011	Formosa 2
Hermeuptychia isabella	MACN-Bar-Lep-ct 03102	LEPPA025-11	BOLD:ACG1578	MF547274	658[0n]	2011	Buenos Aires 21
Hermeuptychia isabella	MACN-Bar-Lep-ct 03753	LEPPA823-13	BOLD:ACG1578	MF546057	658[0n]	2011	Corrientes 1
Hermeuptychia isabella	MACN-Bar-Lep-ct 03767	LEPPA828-13	BOLD:ACG1578	MF546195	658[0n]	2011	Corrientes 1
Hermeuptychia isabella	MACN-Bar-Lep-ct 03953	LEPPA576-13	BOLD:ACG1578	MF546302	621[0n]	2012	Formosa 4
Hermeuptychia isabella	MACN-Bar-Lep-ct 03956	LEPPA578-13	BOLD:ACG1578	MF545734	658[0n]	2012	Formosa 4
Hermeuptychia isabella	MACN-Bar-Lep-ct 03969	LEPPA587-13	BOLD:ACG1578	MF547139	658[0n]	2012	Formosa 4
Hermeuptychia isabella	MACN-Bar-Lep-ct 06046	LEPAR979-14	BOLD:ACG1578	MF546868	658[0n]	2013	Formosa 5
Hermeuptychia isabella	MACN-Bar-Lep-ct 06102	LEPAR1035-14	BOLD:ACG1578	MF546873	658[2n]	2013	Formosa 5
Hesperocharis infrasignata	MACN-Bar-Lep-ct 07069	LEPAR1147-15			0	2006	Entre Ríos 9

Hesperocharis infrasignata	MACN-Bar-Lep-ct 07071	LEPAR1149-15			0	1998	Entre Ríos 8
Hesperocharis infrasignata	MACN-Bar-Lep-ct 07073	LEPAR1151-15			0	2008	Córdoba 5
Hesperocharis infrasignata	MACN-Bar-Lep-ct 07075	LEPAR1153-15			0	2008	Córdoba 5
Hesperocharis paranensis	MACN-Bar-Lep-ct 07067	LEPAR1145-15			0	2006	Buenos Aires 17
Hylephila phyleus	MACN-Bar-Lep-ct 01461	LEPAR106-11	BOLD:AAD1706	MF545890	658[0n]	2011	Entre Ríos 3
Hylephila phyleus	MACN-Bar-Lep-ct 01758	LEPAR238-11	BOLD:AAD1706	MF546193	658[0n]	2011	Entre Ríos 6
Hylephila phyleus	MACN-Bar-Lep-ct 01764	LEPAR242-11	BOLD:AAD1706	MF546617	658[0n]	2011	Entre Ríos 6
Hylephila phyleus	MACN-Bar-Lep-ct 01808	LEPAR249-11	BOLD:AAD1706	MF546180	658[0n]	2011	Entre Ríos 3
Hylephila phyleus	MACN-Bar-Lep-ct 02626	LEPPA280-12	BOLD:AAD1706	MF546028	658[0n]	2011	Formosa 2
Hylephila phyleus	MACN-Bar-Lep-ct 03062	LEPPA175-11	BOLD:AAD1706	MF546989	658[0n]	2011	Buenos Aires 9
Hylephila phyleus	MACN-Bar-Lep-ct 03069	LEPPA182-11	BOLD:AAD1706	MF546785	658[0n]	2011	Buenos Aires 6
Hylephila phyleus	MACN-Bar-Lep-ct 03074	LEPPA187-11	BOLD:AAD1706	MF547248	658[0n]	2011	Buenos Aires 6
Hylephila phyleus	MACN-Bar-Lep-ct 03593	LEPPA718-13	BOLD:AAD1706	MF547053	658[0n]	2011	Corrientes 1
Hylephila phyleus	MACN-Bar-Lep-ct 03708	LEPPA792-13	BOLD:AAD1706	MF546158	658[0n]	2011	Corrientes 1
Hylephila phyleus	MACN-Bar-Lep-ct 03782	LEPPA837-13	BOLD:AAD1706	MF546174	658[0n]	2011	Corrientes 1
Hylephila phyleus	MACN-Bar-Lep-ct 03882	LEPPA523-13	BOLD:AAD1706	MF546034	625[0n]	2012	Formosa 4
Hylephila phyleus	MACN-Bar-Lep-ct 03935	LEPPA560-13	BOLD:AAD1706	MF546438	658[0n]	2012	Formosa 4
Hylephila phyleus	MACN-Bar-Lep-ct 03966	LEPPA586-13	BOLD:AAD1706	MF547362	658[0n]	2012	Formosa 4
Hypanartia lethe	MACN-Bar-Lep-ct 00243	LEPAR329-11	BOLD:ACE8211	MF546115	658[0n]	2010	Misiones 2
Hypanartia lethe	MACN-Bar-Lep-ct 00292	LEPAR354-11	BOLD:ACE8211	MF546543	658[0n]	2010	Misiones 2
Hypanartia lethe	MACN-Bar-Lep-ct 00293	LEPAR355-11	BOLD:ACE8211	MF545428	658[0n]	2010	Misiones 2
Hypanartia lethe	MACN-Bar-Lep-ct 00320	LEPAR370-11	BOLD:ACE8211	MF546782	658[0n]	2010	Misiones 2
Hypanartia lethe	MACN-Bar-Lep-ct 01003	LEPAR703-11	BOLD:ACE8211	MF546072	658[0n]	2010	Misiones 2
Hypanartia lethe	MACN-Bar-Lep-ct 02658	LEPPA311-12	BOLD:ACE8211	MF546557	658[0n]	2011	Formosa 2
Hypanartia lethe	MACN-Bar-Lep-ct 02691	LEPPA340-12	BOLD:ACE8211	MF546713	658[0n]	2011	Formosa 2
Hypanartia lethe	MACN-Bar-Lep-ct 02740	LEPPA373-12	BOLD:ACE8211	MF546021	658[0n]	2011	Formosa 3
Hypna clytemnestra	MACN-Bar-Lep-ct 02288	LEPIG374-11	BOLD:AAI8602	MF546828	658[0n]	2011	Misiones 3
Hypna clytemnestra	MACN-Bar-Lep-ct 02519	LEPIG541-11	BOLD:AAI8602	MF545882	658[0n]	2011	Misiones 3
Hypothyris euclea	MACN-Bar-Lep-ct 01138	LEPAR772-11	BOLD:AAB6973	MF547360	658[0n]	2010	Misiones 2
Hypothyris euclea	MACN-Bar-Lep-ct 01139	LEPAR773-11	BOLD:AAB6973	MF546050	658[0n]	2010	Misiones 2
Hypothyris euclea	MACN-Bar-Lep-ct 01150	LEPAR782-11	BOLD:AAB6973	MF546572	658[0n]	2010	Misiones 2
Hypothyris euclea	MACN-Bar-Lep-ct 01875	LEPIG003-11	BOLD:AAB6973	MF547090	658[0n]	2011	Misiones 2
Hypothyris euclea	MACN-Bar-Lep-ct 01878	LEPIG006-11	BOLD:AAB6973	MF547275	658[0n]	2011	Misiones 2

Hypothyris euclea	MACN-Bar-Lep-ct 01902	LEPIG030-11	BOLD:AAB6973	MF545935	658[0n]	2011	Misiones 2
Hypothyris euclea	MACN-Bar-Lep-ct 01975	LEPIG094-11			0	2011	Misiones 2
Hypothyris euclea	MACN-Bar-Lep-ct 01994	LEPIG111-11	BOLD:AAB6973	MF545668	658[0n]	2011	Misiones 2
Hypothyris euclea	MACN-Bar-Lep-ct 02042	LEPIG151-11	BOLD:AAB6973	MF547030	658[0n]	2011	Misiones 2
Ithomia agnosia	MACN-Bar-Lep-ct 02253	LEPIG341-11	BOLD:AAZ7583	MF546007	658[0n]	2011	Misiones 3
Itylos moza	MACN-Bar-Lep-ct 06467	LEPPA1055-14	BOLD:ACT0777	MF546817	658[0n]	2013	Córdoba 4
Junonia genoveva	MACN-Bar-Lep-ct 00046	LEPAR030-11	BOLD:AAA5788	MF547155	658[0n]	1996	Buenos Aires 15
Junonia genoveva	MACN-Bar-Lep-ct 00049	LEPAR031-11	BOLD:AAA5788	MF546855	658[0n]	2007	Buenos Aires 1
Junonia genoveva	MACN-Bar-Lep-ct 00050	LEPAR032-11	BOLD:AAA5788	MF546429	658[0n]	2007	Buenos Aires 1
Junonia genoveva	MACN-Bar-Lep-ct 00051	LEPAR033-11	BOLD:AAA5788	MF545995	658[0n]	2007	Buenos Aires 1
Junonia genoveva	MACN-Bar-Lep-ct 00053	LEPAR034-11	BOLD:AAA5788	MF545455	658[0n]	2007	Buenos Aires 1
Junonia genoveva	MACN-Bar-Lep-ct 01605	LEPAR171-11	BOLD:AAA5788	MF545919	658[0n]	2011	Entre Ríos 5
Junonia genoveva	MACN-Bar-Lep-ct 01722	LEPAR225-11	BOLD:AAA5788	MF546424	658[0n]	2011	Entre Ríos 3
Junonia genoveva	MACN-Bar-Lep-ct 01839	LEPAR264-11	BOLD:AAA5788	MF546734	658[0n]	2011	Entre Ríos 3
Junonia genoveva	MACN-Bar-Lep-ct 01842	LEPAR266-11	BOLD:AAA5788	MF545765	658[0n]	2011	Entre Ríos 3
Junonia genoveva	MACN-Bar-Lep-ct 02115	LEPIG213-11	BOLD:AAA5788	MF546645	658[0n]	2011	Misiones 2
Junonia genoveva	MACN-Bar-Lep-ct 02227	LEPIG316-11	BOLD:AAA5788	MF545808	658[0n]	2011	Misiones 3
Junonia genoveva	MACN-Bar-Lep-ct 02230	LEPIG319-11	BOLD:AAA5788	MF546564	658[0n]	2011	Misiones 3
Junonia genoveva	MACN-Bar-Lep-ct 02232	LEPIG321-11	BOLD:AAA5788	MF546252	658[0n]	2011	Misiones 3
Junonia genoveva	MACN-Bar-Lep-ct 02357	LEPIG431-11	BOLD:AAA5788	MF546677	658[0n]	2011	Misiones 3
Junonia genoveva	MACN-Bar-Lep-ct 02616	LEPPA270-12	BOLD:AAA5788	MF545533	658[0n]	2011	Formosa 2
Junonia genoveva	MACN-Bar-Lep-ct 02771	LEPPA395-12	BOLD:AAA5788	MF546017	658[0n]	2011	Formosa 3
Junonia genoveva	MACN-Bar-Lep-ct 03063	LEPPA176-11	BOLD:AAA5788	MF545716	658[0n]	2011	Buenos Aires 6
Junonia genoveva	MACN-Bar-Lep-ct 03070	LEPPA183-11	BOLD:AAA5788	MF546344	658[0n]	2011	Buenos Aires 12
Junonia genoveva	MACN-Bar-Lep-ct 03681	LEPPA770-13	BOLD:AAA5788	MF546844	658[0n]	2011	Corrientes 1
Junonia genoveva	MACN-Bar-Lep-ct 03712	LEPPA795-13	BOLD:AAA5788	MF547121	658[0n]	2011	Corrientes 1
Junonia genoveva	MACN-Bar-Lep-ct 03742	LEPPA815-13	BOLD:AAA5788	MF546116	658[0n]	2011	Corrientes 1
Junonia genoveva	MACN-Bar-Lep-ct 03868	LEPPA513-13	BOLD:AAA5788	MF545604	658[0n]	2012	Formosa 4
Junonia genoveva	MACN-Bar-Lep-ct 03880	LEPPA522-13	BOLD:AAA5788	MF545784	658[0n]	2012	Formosa 4
Junonia genoveva	MACN-Bar-Lep-ct 03884	LEPPA524-13	BOLD:AAA5788	MF546242	658[0n]	2012	Formosa 4
Kolana sp. 1	MACN-Bar-Lep-ct 00432	LEPAR444-11	BOLD:AAZ6160	MF547396	658[0n]	2010	Misiones 2
Lasaia agesilas	MACN-Bar-Lep-ct 00405	LEPAR421-11	BOLD:AAE1328	MF547191	658[0n]	2010	Misiones 2
Lasaia agesilas	MACN-Bar-Lep-ct 00864	LEPAR633-11	BOLD:AAE1328	MF545805	658[0n]	2010	Misiones 2

Lasaia agesilas	MACN-Bar-Lep-ct 01046	LEPAR739-11	BOLD:AAE1328	MF546919	658[0n]	2010	Misiones 2
Lasaia agesilas	MACN-Bar-Lep-ct 02378	LEPIG446-11	BOLD:AAE1328	MF546392	658[0n]	2011	Misiones 3
Lasaia arsis	MACN-Bar-Lep-ct 00390	LEPAR412-11	BOLD:AAZ1151	MF545963	658[0n]	2010	Misiones 2
Lasaia arsis	MACN-Bar-Lep-ct 01047	LEPAR740-11	BOLD:AAZ1151	MF546206	658[0n]	2010	Misiones 2
Lasaia arsis	MACN-Bar-Lep-ct 01222	LEPAR837-11	BOLD:AAZ1151	MF547109	658[0n]	2010	Misiones 2
Lento krexoides	MACN-Bar-Lep-ct 02421	LEPIG483-11	BOLD:AAZ9993	MF547106	658[0n]	2011	Misiones 3
Leptophobia aripa	MACN-Bar-Lep-ct 00528	LEPAR465-11	BOLD:AAZ3864	MF545994	658[0n]	2010	Misiones 2
Leptophobia aripa	MACN-Bar-Lep-ct 00545	LEPAR477-11	BOLD:AAZ3864	MF545682	658[0n]	2010	Misiones 2
Leptophobia aripa	MACN-Bar-Lep-ct 02137	LEPIG234-11	BOLD:AAZ3864	MF547202	658[0n]	2011	Misiones 3
Leptophobia aripa	MACN-Bar-Lep-ct 02160	LEPIG255-11	BOLD:AAZ3864	MF546208	658[0n]	2011	Misiones 3
Leptophobia aripa	MACN-Bar-Lep-ct 02195	LEPIG285-11	BOLD:AAZ3864	MF546064	658[0n]	2011	Misiones 3
Leptophobia aripa	MACN-Bar-Lep-ct 02255	LEPIG343-11	BOLD:AAZ3864	MF545780	658[0n]	2011	Misiones 3
Leptophobia aripa	MACN-Bar-Lep-ct 02319	LEPIG402-11	BOLD:AAZ3864	MF545754	658[0n]	2011	Misiones 3
Leptophobia aripa	MACN-Bar-Lep-ct 02390	LEPIG456-11	BOLD:AAZ3864	MF546329	658[0n]	2011	Misiones 3
Leptotes cassius	MACN-Bar-Lep-ct 00168	LEPAR294-11	BOLD:AAD3953	MF547120	658[0n]	2010	Misiones 2
Leptotes cassius	MACN-Bar-Lep-ct 00175	LEPAR297-11	BOLD:AAD3953	MF546160	658[0n]	2010	Misiones 2
Leptotes cassius	MACN-Bar-Lep-ct 00177	LEPAR298-11	BOLD:AAD3953	MF547058	658[0n]	2010	Misiones 2
Leptotes cassius	MACN-Bar-Lep-ct 00413	LEPAR425-11	BOLD:AAD3953	MF546514	658[0n]	2010	Misiones 2
Leptotes cassius	MACN-Bar-Lep-ct 00827	LEPAR603-11	BOLD:AAD3953	MF546595	658[0n]	2010	Misiones 2
Leptotes cassius	MACN-Bar-Lep-ct 01546	LEPAR154-11	BOLD:AAD3953	MF546769	658[0n]	2011	Entre Ríos 3
Leptotes cassius	MACN-Bar-Lep-ct 01700	LEPAR215-11	BOLD:AAD3953	MF545905	658[0n]	2011	Entre Ríos 4
Leptotes cassius	MACN-Bar-Lep-ct 01703	LEPAR217-11	BOLD:AAD3953	MF545592	658[0n]	2011	Entre Ríos 4
Leptotes cassius	MACN-Bar-Lep-ct 01728	LEPAR228-11	BOLD:AAD3953	MF545557	658[0n]	2011	Entre Ríos 3
Leptotes cassius	MACN-Bar-Lep-ct 01811	LEPAR251-11	BOLD:AAD3953	MF545693	658[0n]	2011	Entre Ríos 3
Leptotes cassius	MACN-Bar-Lep-ct 03579	LEPPA709-13	BOLD:AAD3953	MF546228	658[0n]	2011	Corrientes 1
Leptotes cassius	MACN-Bar-Lep-ct 03608	LEPPA730-13	BOLD:AAD3953	MF545956	618[0n]	2011	Corrientes 1
Leptotes cassius	MACN-Bar-Lep-ct 03696	LEPPA783-13	BOLD:AAD3953	MF546300	658[0n]	2011	Corrientes 1
Lerodea eufala	MACN-Bar-Lep-ct 01717	LEPAR222-11	BOLD:AAF7744	MF546315	658[0n]	2011	Entre Ríos 4
Lerodea eufala	MACN-Bar-Lep-ct 01727	LEPAR227-11	BOLD:AAF7744	MF546718	658[0n]	2011	Entre Ríos 3
Lerodea eufala	MACN-Bar-Lep-ct 02722	LEPPA361-12	BOLD:AAF7744	MF545689	658[0n]	2011	Formosa 3
Lerodea eufala	MACN-Bar-Lep-ct 06484	LEPPA1072-14	BOLD:AAF7744	MF547315	658[0n]	2010	Corrientes 3
Libytheana carinenta	MACN-Bar-Lep-ct 00056	LEPAR036-11	BOLD:AAC5274	MF547300	658[0n]	2010	Formosa 1
Libytheana carinenta	MACN-Bar-Lep-ct 00378	LEPAR409-11	BOLD:AAC5274	MF546925	658[0n]	2010	Misiones 2

Libytheana carinenta	MACN-Bar-Lep-ct 01609	LEPAR173-11	BOLD:AAC5274	MF545443	658[0n]	2011	Entre Ríos 5
Libytheana carinenta	MACN-Bar-Lep-ct 02475	LEPIG515-11	BOLD:AAC5274	MF546312	658[0n]	2011	Misiones 3
Libytheana carinenta	MACN-Bar-Lep-ct 02499	LEPIG532-11	BOLD:AAC5274	MF546584	658[0n]	2011	Misiones 3
Libytheana carinenta	MACN-Bar-Lep-ct 02586	LEPPA241-12	BOLD:AAC5274	MF546240	658[0n]	2011	Formosa 2
Libytheana carinenta	MACN-Bar-Lep-ct 02602	LEPPA257-12	BOLD:AAC5274	MF545823	658[0n]	2011	Formosa 2
Libytheana carinenta	MACN-Bar-Lep-ct 03094	LEPPA017-11			0	2011	Córdoba 2
Libytheana carinenta	MACN-Bar-Lep-ct 06057	LEPAR990-14	BOLD:AAC5274	MF546569	658[0n]	2013	Formosa 5
Libytheana carinenta	MACN-Bar-Lep-ct 06096	LEPAR1029-14	BOLD:AAC5274	MF547171	658[4n]	2013	Formosa 5
Libytheana carinenta	MACN-Bar-Lep-ct 06398	LEPPA986-14			0	2013	Chaco 2
Ludens silvaticus	MACN-Bar-Lep-ct 01340	LEPAR921-11	BOLD:AAZ5132	MF547224	658[0n]	2010	Misiones 2
Lycas argentea	MACN-Bar-Lep-ct 00819	LEPAR596-11	BOLD:AAZ2926	MF545898	658[0n]	2010	Misiones 2
Lycas argentea	MACN-Bar-Lep-ct 01149	LEPAR781-11	BOLD:AAZ2926	MF545386	658[0n]	2010	Misiones 2
Lycas argentea	MACN-Bar-Lep-ct 01276	LEPAR863-11	BOLD:AAZ3990	MF546772	658[0n]	2010	Misiones 2
Lycas argentea	MACN-Bar-Lep-ct 01279	LEPAR866-11	BOLD:AAZ2926	MF545567	658[0n]	2010	Misiones 2
Lychnuchoides ozias	MACN-Bar-Lep-ct 01295	LEPAR879-11	BOLD:AAZ5133	MF546203	658[0n]	2010	Misiones 2
Magnastigma hirsuta	MACN-Bar-Lep-ct 01327	LEPAR908-11	BOLD:AAZ4023	MF546909	658[0n]	2010	Misiones 2
Magneuptychia lea	MACN-Bar-Lep-ct 01885	LEPIG013-11	BOLD:AAZ9446	MF545572	658[0n]	2011	Misiones 2
Magneuptychia lea	MACN-Bar-Lep-ct 02087	LEPIG190-11	BOLD:AAZ9446	MF546153	658[0n]	2011	Misiones 2
Magneuptychia pallema	MACN-Bar-Lep-ct 01972	LEPIG091-11	BOLD:AAY8379	MF547303	658[0n]	2011	Misiones 2
Manataria hercyna	MACN-Bar-Lep-ct 00560	LEPAR488-11	BOLD:AAZ3906	MF546525	658[0n]	2010	Misiones 2
Manataria hercyna	MACN-Bar-Lep-ct 00561	LEPAR489-11	BOLD:AAZ3906	MF545794	658[0n]	2010	Misiones 2
Manataria hercyna	MACN-Bar-Lep-ct 00605	LEPAR528-11	BOLD:AAZ3906	MF545489	658[0n]	2010	Misiones 2
Manataria hercyna	MACN-Bar-Lep-ct 00802	LEPAR582-11	BOLD:AAZ3906	MF545937	658[0n]	2010	Misiones 2
Manataria hercyna	MACN-Bar-Lep-ct 00884	LEPAR651-11	BOLD:AAZ3906	MF546112	658[0n]	2010	Misiones 2
Marpesia chiron	MACN-Bar-Lep-ct 00326	LEPAR374-11	BOLD:ABZ7294	MF546345	658[0n]	2010	Misiones 2
Marpesia chiron	MACN-Bar-Lep-ct 00788	LEPAR570-11	BOLD:ABZ7294	MF546194	658[0n]	2010	Misiones 2
Marpesia chiron	MACN-Bar-Lep-ct 01004	LEPAR704-11	BOLD:ABZ7294	MF545425	658[0n]	2010	Misiones 2
Marpesia chiron	MACN-Bar-Lep-ct 01176	LEPAR800-11	BOLD:ABZ7294	MF545454	658[0n]	2010	Misiones 2
Marpesia chiron	MACN-Bar-Lep-ct 01184	LEPAR806-11	BOLD:ABZ7294	MF546885	658[0n]	2010	Misiones 2
Marpesia chiron	MACN-Bar-Lep-ct 02526	LEPIG548-11	BOLD:ABZ7294	MF545717	658[0n]	2011	Misiones 3
Marpesia petreus	MACN-Bar-Lep-ct 00241	LEPAR327-11	BOLD:AAB0882	MF545550	658[0n]	2010	Misiones 2
Marpesia petreus	MACN-Bar-Lep-ct 00267	LEPAR340-11	BOLD:AAB0882	MF547054	658[0n]	2010	Misiones 2
Marpesia petreus	MACN-Bar-Lep-ct 00301	LEPAR360-11	BOLD:AAB0882	MF546646	658[0n]	2010	Misiones 2

Marpesia petreus	MACN-Bar-Lep-ct 00558	LEPAR486-11	BOLD:AAB0882	MF547049	658[0n]	2010	Misiones 2
Marpesia petreus	MACN-Bar-Lep-ct 00572	LEPAR500-11	BOLD:AAB0882	MF546651	658[0n]	2010	Misiones 2
Marpesia petreus	MACN-Bar-Lep-ct 03673	LEPPA765-13	BOLD:AAB0882	MF546837	658[0n]	2011	Corrientes 1
Marpesia petreus	MACN-Bar-Lep-ct 06022	LEPAR955-14	BOLD:AAB0882	MF546541	658[0n]	2013	Formosa 5
Mcclungia cymo	MACN-Bar-Lep-ct 01938	LEPIG061-11	BOLD:AAZ9627	MF547301	658[0n]	2011	Misiones 2
Mechanitis lysimnia	MACN-Bar-Lep-ct 00329	LEPAR376-11	BOLD:ACF4280	MF546287	658[0n]	2010	Misiones 2
Mechanitis lysimnia	MACN-Bar-Lep-ct 01880	LEPIG008-11	BOLD:ACF4280	MF546119	658[0n]	2011	Misiones 2
Mechanitis lysimnia	MACN-Bar-Lep-ct 01892	LEPIG020-11	BOLD:ACF4280	MF546707	658[0n]	2011	Misiones 2
Mechanitis lysimnia	MACN-Bar-Lep-ct 01914	LEPIG040-11	BOLD:ACF4280	MF546875	658[0n]	2011	Misiones 2
Mechanitis lysimnia	MACN-Bar-Lep-ct 01928	LEPIG052-11	BOLD:ACF4280	MF546222	658[0n]	2011	Misiones 2
Mechanitis lysimnia	MACN-Bar-Lep-ct 01979	LEPIG098-11	BOLD:ACF4280	MF546178	658[0n]	2011	Misiones 2
Mechanitis lysimnia	MACN-Bar-Lep-ct 02001	LEPIG118-11	BOLD:ACF4280	MF546599	658[0n]	2011	Misiones 2
Mechanitis lysimnia	MACN-Bar-Lep-ct 02004	LEPIG121-11	BOLD:ACF4280	MF547144	658[0n]	2011	Misiones 2
Mechanitis lysimnia	MACN-Bar-Lep-ct 02023	LEPIG136-11	BOLD:ACF4280	MF545614	658[0n]	2011	Misiones 2
Mechanitis lysimnia	MACN-Bar-Lep-ct 02386	LEPIG452-11	BOLD:ACF4280	MF547017	658[0n]	2011	Misiones 3
Melanis aegates	MACN-Bar-Lep-ct 03955	LEPPA577-13	BOLD:ACG2608	MF546367	658[0n]	2012	Formosa 4
Melanis hillapana	MACN-Bar-Lep-ct 02736	LEPPA370-12	BOLD:ACA9064	MF545850	658[0n]	2011	Formosa 3
Melanis xenia	MACN-Bar-Lep-ct 01290	LEPAR876-11	BOLD:AAZ5136	MF547263	658[0n]	2010	Misiones 2
Memphis acidalia	MACN-Bar-Lep-ct 01182	LEPAR805-11	BOLD:ACE7789	MF545964	658[0n]	2010	Misiones 2
Memphis moruus	MACN-Bar-Lep-ct 00530	LEPAR467-11	BOLD:ABY4511	MF546730	658[0n]	2010	Misiones 2
Memphis moruus	MACN-Bar-Lep-ct 00548	LEPAR479-11	BOLD:ABY4511	MF547403	658[0n]	2010	Misiones 2
Memphis moruus	MACN-Bar-Lep-ct 00835	LEPAR610-11	BOLD:ABY4511	MF546719	658[0n]	2010	Misiones 2
Memphis moruus	MACN-Bar-Lep-ct 00968	LEPAR675-11	BOLD:ABY4511	MF546503	658[0n]	2010	Misiones 2
Memphis moruus	MACN-Bar-Lep-ct 01906	LEPIG033-11	BOLD:ABY4511	MF547025	658[0n]	2011	Misiones 2
Memphis moruus	MACN-Bar-Lep-ct 01996	LEPIG113-11	BOLD:ABY4511	MF547117	658[0n]	2011	Misiones 2
Memphis moruus	MACN-Bar-Lep-ct 02418	LEPIG481-11	BOLD:ABY4511	MF546428	658[0n]	2011	Misiones 3
Memphis moruus	MACN-Bar-Lep-ct 02739	LEPPA372-12	BOLD:ABY4511	MF545845	658[0n]	2011	Formosa 3
Mesene celetes	MACN-Bar-Lep-ct 02106	LEPIG208-11	BOLD:AAZ9853	MF545538	658[0n]	2011	Misiones 2
Mesosemia odice	MACN-Bar-Lep-ct 01166	LEPAR792-11	BOLD:AAZ2874	MF545847	658[0n]	2010	Misiones 2
Mesosemia odice	MACN-Bar-Lep-ct 01274	LEPAR861-11	BOLD:AAZ2874	MF547339	658[0n]	2010	Misiones 2
Methionopsis ina	MACN-Bar-Lep-ct 02132	LEPIG229-11	BOLD:AAZ7555	MF546012	658[0n]	2011	Misiones 2
Methionopsis ina	MACN-Bar-Lep-ct 02228	LEPIG317-11	BOLD:AAZ7555	MF546944	658[0n]	2011	Misiones 3
Methona themisto	MACN-Bar-Lep-ct 01134	LEPAR768-11	BOLD:AAX4346	MF545414	658[0n]	2010	Misiones 2

Methona themisto	MACN-Bar-Lep-ct 01280	LEPAR867-11	BOLD:AAX4346	MF546056	658[0n]	2010	Misiones 2
Methona themisto	MACN-Bar-Lep-ct 01884	LEPIG012-11	BOLD:AAX4346	MF546325	658[0n]	2011	Misiones 2
Methona themisto	MACN-Bar-Lep-ct 03568	LEPPA701-13	BOLD:AAX4346	MF546881	612[0n]		Corrientes 2
Metron oropa	MACN-Bar-Lep-ct 00866	LEPAR635-11	BOLD:AAZ5075	MF546502	658[0n]	2010	Misiones 2
Milanion leucaspis	MACN-Bar-Lep-ct 03808	LEPPA852-13	BOLD:ACG1660	MF547381	658[0n]	2011	Corrientes 1
Milanion leucaspis	MACN-Bar-Lep-ct 03819	LEPPA858-13	BOLD:ACG1660	MF545820	658[0n]	2011	Corrientes 1
Milanion leucaspis	MACN-Bar-Lep-ct 03832	LEPPA865-13	BOLD:ACG1660	MF546629	618[0n]	2011	Corrientes 1
Mimoides lysithous	MACN-Bar-Lep-ct 00526	LEPAR463-11	BOLD:AAZ2891	MF546682	658[0n]	2010	Misiones 2
Mimoides lysithous	MACN-Bar-Lep-ct 01148	LEPAR780-11	BOLD:AAZ2891	MF546436	658[0n]	2010	Misiones 2
Mimoides microdamas	MACN-Bar-Lep-ct 03669	LEPPA762-13	BOLD:ACG1650	MF546816	658[0n]	2011	Corrientes 1
Mimoides microdamas	MACN-Bar-Lep-ct 06020	LEPAR953-14	BOLD:ACG1650	MF547287	658[0n]	2013	Formosa 5
Ministrymon azia	MACN-Bar-Lep-ct 00414	LEPAR426-11	BOLD:ACF1986	MF545888	658[0n]	2010	Misiones 2
Ministrymon azia	MACN-Bar-Lep-ct 00415	LEPAR427-11	BOLD:ACF1986	MF545936	658[0n]	2010	Misiones 2
Ministrymon azia	MACN-Bar-Lep-ct 01298	LEPAR880-11	BOLD:ACF1986	MF546806	658[0n]	2010	Misiones 2
Ministrymon azia	MACN-Bar-Lep-ct 02758	LEPPA387-12	BOLD:AAE9908	MF547320	658[0n]	2011	Formosa 2
Ministrymon cruenta	MACN-Bar-Lep-ct 02588	LEPPA243-12	BOLD:AAF0000	MF545992	658[0n]	2011	Formosa 2
Ministrymon cruenta	MACN-Bar-Lep-ct 03971	LEPPA589-13	BOLD:AAF0000	MF547212	626[0n]	2012	Formosa 4
Ministrymon gamma	MACN-Bar-Lep-ct 03927	LEPPA554-13	BOLD:AAF0000	MF546479	658[0n]	2012	Formosa 4
Ministrymon una	MACN-Bar-Lep-ct 03731	LEPPA806-13	BOLD:ACK2098	MF546256	658[0n]	2011	Corrientes 1
Mithras hannelore	MACN-Bar-Lep-ct 01328	LEPAR909-11	BOLD:AAZ4496	MF545828	658[0n]	2010	Misiones 2
Mnasilus allubita	MACN-Bar-Lep-ct 02790	LEPPA404-12	BOLD:AAC7501	MF547238	658[0n]	2011	Formosa 2
Mnasilus allubita	MACN-Bar-Lep-ct 03899	LEPPA533-13	BOLD:AAC7501	MF546903	619[0n]	2012	Formosa 4
Mnasilus allubita	MACN-Bar-Lep-ct 06072	LEPAR1005-14	BOLD:AAC7501	MF546998	658[1n]	2013	Formosa 5
Mnasilus allubita	MACN-Bar-Lep-ct 06104	LEPAR1037-14	BOLD:AAC7501	MF546548	658[0n]	2013	Formosa 5
Moneuptychia griseldis	MACN-Bar-Lep-ct 01167	LEPAR793-11	BOLD:AAZ1921	MF546086	658[0n]	2010	Misiones 2
Moneuptychia griseldis	MACN-Bar-Lep-ct 01203	LEPAR821-11	BOLD:AAZ1921	MF547345	658[0n]	2010	Misiones 2
Moneuptychia griseldis	MACN-Bar-Lep-ct 01269	LEPAR856-11	BOLD:AAZ1921	MF546508	658[0n]	2010	Misiones 2
Moneuptychia griseldis	MACN-Bar-Lep-ct 07103	LEPAR1181-15			0	2004	Misiones 7
Moneuptychia griseldis	MACN-Bar-Lep-ct 07105	LEPAR1183-15			0	2004	Misiones 7
Moneuptychia paeon	MACN-Bar-Lep-ct 00187	LEPAR305-11	BOLD:AAM4884	MF545946	658[0n]	2010	Misiones 2
Moneuptychia soter	MACN-Bar-Lep-ct 00419	LEPAR431-11	BOLD:AAM4885	MF546415	658[0n]	2010	Misiones 2
Moneuptychia soter	MACN-Bar-Lep-ct 00539	LEPAR474-11	BOLD:AAM4885	MF546594	658[0n]	2010	Misiones 2
Moneuptychia soter	MACN-Bar-Lep-ct 01324	LEPAR905-11	BOLD:AAM4885	MF545759	658[0n]	2010	Misiones 2

Morpho epistrophus	MACN-Bar-Lep-ct 01454	LEPAR102-11	BOLD:AAP9937	MF547313	658[0n]	2011	Entre Ríos 3
Morpho epistrophus	MACN-Bar-Lep-ct 01457	LEPAR104-11	BOLD:AAP9937	MF545679	658[0n]	2011	Entre Ríos 3
Morpho epistrophus	MACN-Bar-Lep-ct 01460	LEPAR105-11	BOLD:AAP9937	MF547365	658[0n]	2011	Entre Ríos 3
Morpho epistrophus	MACN-Bar-Lep-ct 01471	LEPAR110-11	BOLD:AAP9937	MF547075	658[0n]	2011	Entre Ríos 3
Morpho epistrophus	MACN-Bar-Lep-ct 01473	LEPAR111-11	BOLD:AAP9937	MF547304	658[0n]	2011	Entre Ríos 3
Morpho epistrophus	MACN-Bar-Lep-ct 03200	LEPPA082-11	BOLD:AAP9937	MF547219	658[0n]	2011	Buenos Aires 21
Morpho epistrophus	MACN-Bar-Lep-ct 03201	LEPPA084-11	BOLD:AAP9937	MF546449	658[0n]	2011	Buenos Aires 21
Morpho epistrophus	MACN-Bar-Lep-ct 03202	LEPPA086-11	BOLD:AAP9937	MF547331	658[0n]	2011	Buenos Aires 21
Morpho epistrophus	MACN-Bar-Lep-ct 03203	LEPPA088-11	BOLD:AAP9937	MF547242	658[0n]	2011	Buenos Aires 21
Morpho epistrophus	MACN-Bar-Lep-ct 03204	LEPPA090-11	BOLD:AAP9937	MF545830	658[0n]	2011	Buenos Aires 21
Morpho helenor	MACN-Bar-Lep-ct 00818	LEPAR595-11	BOLD:ACE6365	MF545456	658[0n]	2010	Misiones 2
Morpho helenor	MACN-Bar-Lep-ct 01006	LEPAR706-11	BOLD:ACE6365	MF545983	658[0n]	2010	Misiones 2
Morpho helenor	MACN-Bar-Lep-ct 02212	LEPIG301-11	BOLD:ACE6365	MF547258	658[0n]	2011	Misiones 3
Morpho helenor	MACN-Bar-Lep-ct 02215	LEPIG304-11	BOLD:ACE6365	MF547169	658[0n]	2011	Misiones 3
Morpho helenor	MACN-Bar-Lep-ct 02412	LEPIG477-11	BOLD:ACE6365	MF547007	658[0n]	2011	Misiones 3
Morpho helenor	MACN-Bar-Lep-ct 02415	LEPIG479-11	BOLD:ACE6365	MF545482	658[0n]	2011	Misiones 3
Morpho helenor	MACN-Bar-Lep-ct 02530	LEPIG551-11	BOLD:ACE6365	MF545810	658[0n]	2011	Misiones 3
Morpho helenor	MACN-Bar-Lep-ct 02533	LEPIG554-11	BOLD:ACE6365	MF546171	658[0n]	2011	Misiones 3
Morpho helenor	MACN-Bar-Lep-ct 02536	LEPIG556-11	BOLD:ACE6365	MF545539	658[0n]	2011	Misiones 3
Morpho helenor	MACN-Bar-Lep-ct 03887	LEPPA526-13	BOLD:ACE6365	MF546062	658[0n]	2012	Formosa 4
Morpho helenor	MACN-Bar-Lep-ct 03911	LEPPA541-13	BOLD:ACE6365	MF546709	604[0n]	2012	Formosa 4
Morpho helenor	MACN-Bar-Lep-ct 03916	LEPPA545-13	BOLD:ACE6365	MF545893	611[0n]	2012	Formosa 4
Morpho iphitus	MACN-Bar-Lep-ct 07101	LEPAR1179-15			0	1998	Misiones 13
Morys geisa	MACN-Bar-Lep-ct 01344	LEPAR925-11	BOLD:AAZ4662	MF546906	658[0n]	2010	Misiones 2
Morys geisa	MACN-Bar-Lep-ct 02071	LEPIG175-11	BOLD:AAZ7848	MF546628	658[0n]	2011	Misiones 2
Morys geisa	MACN-Bar-Lep-ct 02074	LEPIG178-11	BOLD:AAZ4662	MF545635	658[0n]	2011	Misiones 2
Mylon maimon	MACN-Bar-Lep-ct 00972	LEPAR679-11	BOLD:AAB9649	MF547247	658[0n]	2010	Misiones 2
Mylon maimon	MACN-Bar-Lep-ct 00989	LEPAR693-11	BOLD:AAB9649	MF545574	658[0n]	2010	Misiones 2
Mylon maimon	MACN-Bar-Lep-ct 00993	LEPAR695-11	BOLD:AAB9649	MF547012	658[0n]	2010	Misiones 2
Mylon maimon	MACN-Bar-Lep-ct 01015	LEPAR713-11	BOLD:AAB9649	MF545686	658[0n]	2010	Misiones 2
Mylon maimon	MACN-Bar-Lep-ct 01191	LEPAR813-11	BOLD:AAB9649	MF547252	624[0n]	2010	Misiones 2
Mylon maimon	MACN-Bar-Lep-ct 02173	LEPIG267-11	BOLD:AAB9649	MF547329	658[0n]	2011	Misiones 3
Mylon maimon	MACN-Bar-Lep-ct 02337	LEPIG418-11	BOLD:AAB9649	MF546879	658[0n]	2011	Misiones 3

Mylon maimon	MACN-Bar-Lep-ct 02342	LEPIG421-11	BOLD:AAB9649	MF547100	658[0n]	2011	Misiones 3
Mylon maimon	MACN-Bar-Lep-ct 02438	LEPIG495-11	BOLD:AAB9649	MF546537	658[0n]	2011	Misiones 3
Mylon maimon	MACN-Bar-Lep-ct 02447	LEPIG501-11	BOLD:AAB9649	MF546551	658[0n]	2011	Misiones 3
Myscelia orsis	MACN-Bar-Lep-ct 00982	LEPAR688-11	BOLD:AAD6462	MF547230	658[0n]	2010	Misiones 2
Myscelia orsis	MACN-Bar-Lep-ct 01281	LEPAR868-11	BOLD:AAD6462	MF546181	658[0n]	2010	Misiones 2
Myscelia orsis	MACN-Bar-Lep-ct 01936	LEPIG059-11	BOLD:AAD6462	MF545746	658[0n]	2011	Misiones 2
Myscelia orsis	MACN-Bar-Lep-ct 01974	LEPIG093-11	BOLD:AAD6462	MF547015	658[0n]	2011	Misiones 2
Myscelus amystis	MACN-Bar-Lep-ct 02289	LEPIG375-11	BOLD:AAB6160	MF547034	658[0n]	2011	Misiones 3
Myscelus amystis	MACN-Bar-Lep-ct 02397	LEPIG462-11	BOLD:AAB6160	MF547369	650[0n]	2011	Misiones 3
Nascus phocus	MACN-Bar-Lep-ct 02234	LEPIG323-11	BOLD:AAB1299	MF547111	658[0n]	2011	Misiones 3
Nascus phocus	MACN-Bar-Lep-ct 02240	LEPIG328-11	BOLD:AAB1299	MF545954	658[0n]	2011	Misiones 3
Nastra ethologus	MACN-Bar-Lep-ct 03757	LEPPA825-13	BOLD:ABW2594	MF546114	618[0n]	2011	Corrientes 1
Nastra ethologus	MACN-Bar-Lep-ct 03919	LEPPA547-13	BOLD:ABW2594	MF546159	658[0n]	2012	Formosa 4
Nesiostrymon calchinia	MACN-Bar-Lep-ct 01329	LEPAR910-11	BOLD:AAZ5068	MF547200	658[0n]	2010	Misiones 2
Nesiostrymon calchinia	MACN-Bar-Lep-ct 01330	LEPAR911-11			0	2010	Misiones 2
Nica flavilla	MACN-Bar-Lep-ct 02159	LEPIG254-11	BOLD:ABZ3590	MF546896	658[0n]	2011	Misiones 3
Nica flavilla	MACN-Bar-Lep-ct 02290	LEPIG376-11	BOLD:ABZ3590	MF547118	658[0n]	2011	Misiones 3
Nica flavilla	MACN-Bar-Lep-ct 02299	LEPIG384-11	BOLD:ABZ3590	MF547265	658[0n]	2011	Misiones 3
Nica flavilla	MACN-Bar-Lep-ct 02310	LEPIG394-11	BOLD:ABZ3590	MF546099	658[0n]	2011	Misiones 3
Nica flavilla	MACN-Bar-Lep-ct 02323	LEPIG404-11	BOLD:ABZ3590	MF547161	654[0n]	2011	Misiones 3
Nisoniades bipuncta	MACN-Bar-Lep-ct 00360	LEPAR396-11	BOLD:AAZ5065	MF545752	658[0n]	2010	Misiones 2
Nisoniades bipuncta	MACN-Bar-Lep-ct 00377	LEPAR408-11	BOLD:AAZ5065	MF546602	658[0n]	2010	Misiones 2
Nisoniades bipuncta	MACN-Bar-Lep-ct 00816	LEPAR593-11	BOLD:AAZ5065	MF546722	658[0n]	2010	Misiones 2
Nisoniades macarius	MACN-Bar-Lep-ct 00416	LEPAR428-11	BOLD:AAZ6319	MF545775	658[0n]	2010	Misiones 2
Nisoniades macarius	MACN-Bar-Lep-ct 01967	LEPIG088-11	BOLD:AAZ6319	MF546386	658[0n]	2011	Misiones 2
Nisoniades macarius	MACN-Bar-Lep-ct 02456	LEPIG505-11	BOLD:AAZ6319	MF546135	658[0n]	2011	Misiones 3
Nisoniades macarius	MACN-Bar-Lep-ct 02548	LEPIG566-11	BOLD:AAZ6319	MF546321	658[0n]	2011	Misiones 3
Notheme erota	MACN-Bar-Lep-ct 01045	LEPAR738-11	BOLD:AAI2507	MF547270	658[0n]	2010	Misiones 2
Notheme erota	MACN-Bar-Lep-ct 02294	LEPIG379-11	BOLD:AAI2507	MF547244	658[0n]	2011	Misiones 3
Notheme erota	MACN-Bar-Lep-ct 02329	LEPIG410-11	BOLD:AAI2507	MF547154	658[0n]	2011	Misiones 3
Notheme erota	MACN-Bar-Lep-ct 02411	LEPIG476-11	BOLD:AAI2507	MF547041	658[0n]	2011	Misiones 3
Notheme erota	MACN-Bar-Lep-ct 02423	LEPIG485-11	BOLD:AAI2507	MF546389	658[0n]	2011	Misiones 3
Notheme erota	MACN-Bar-Lep-ct 02442	LEPIG497-11	BOLD:AAI2507	MF545840	658[0n]	2011	Misiones 3

Nyctelius nyctelius	MACN-Bar-Lep-ct 03970	LEPPA588-13	BOLD:AAA7411	MF545696	618[0n]	2012	Formosa 4
Opoptera aorsa	MACN-Bar-Lep-ct 01206	LEPAR824-11	BOLD:AAZ1862	MF545789	658[0n]	2010	Misiones 2
Opoptera aorsa	MACN-Bar-Lep-ct 01207	LEPAR825-11	BOLD:AAZ1862	MF545989	658[0n]	2010	Misiones 2
Opoptera aorsa	MACN-Bar-Lep-ct 01208	LEPAR826-11	BOLD:AAZ1862	MF545409	658[0n]	2010	Misiones 2
Opsiphanes invirae	MACN-Bar-Lep-ct 00073	LEPAR053-11			0	2009	Buenos Aires 2
Opsiphanes invirae	MACN-Bar-Lep-ct 00154	LEPPA129-11	BOLD:AAA1580	MF546403	658[0n]	2010	Buenos Aires 5
Opsiphanes invirae	MACN-Bar-Lep-ct 02104	LEPIG206-11	BOLD:AAA1580	MF546042	658[0n]	2011	Misiones 2
Opsiphanes invirae	MACN-Bar-Lep-ct 02127	LEPIG224-11	BOLD:AAA1580	MF547146	658[0n]	2011	Misiones 2
Opsiphanes invirae	MACN-Bar-Lep-ct 02129	LEPIG226-11	BOLD:AAA1580	MF547363	658[0n]	2011	Misiones 2
Opsiphanes invirae	MACN-Bar-Lep-ct 02283	LEPIG369-11	BOLD:AAA1580	MF546849	658[0n]	2011	Misiones 3
Opsiphanes invirae	MACN-Bar-Lep-ct 02326	LEPIG407-11	BOLD:AAA1580	MF545786	658[0n]	2011	Misiones 3
Opsiphanes invirae	MACN-Bar-Lep-ct 02554	LEPIG571-11	BOLD:AAA1580	MF545690	658[0n]	2011	Misiones 3
Opsiphanes invirae	MACN-Bar-Lep-ct 06019	LEPAR952-14	BOLD:AAA1580	MF545398	658[0n]	2013	Formosa 5
Orses cynisca	MACN-Bar-Lep-ct 01338	LEPAR919-11	BOLD:AAA8397	MF546661	658[0n]	2010	Misiones 2
Ortilia dicoma	MACN-Bar-Lep-ct 00587	LEPAR513-11	BOLD:AAW9651	MF546639	658[0n]	2010	Misiones 2
Ortilia dicoma	MACN-Bar-Lep-ct 00593	LEPAR519-11	BOLD:AAW9651	MF547149	658[0n]	2010	Misiones 2
Ortilia dicoma	MACN-Bar-Lep-ct 00858	LEPAR627-11	BOLD:AAW9651	MF545914	658[0n]	2010	Misiones 2
Ortilia dicoma	MACN-Bar-Lep-ct 00859	LEPAR628-11	BOLD:AAW9651	MF547112	658[0n]	2010	Misiones 2
Ortilia dicoma	MACN-Bar-Lep-ct 00865	LEPAR634-11	BOLD:AAW9651	MF545688	658[0n]	2010	Misiones 2
Ortilia dicoma	MACN-Bar-Lep-ct 02015	LEPIG129-11	BOLD:AAW9651	MF546699	658[0n]	2011	Misiones 2
Ortilia dicoma	MACN-Bar-Lep-ct 02030	LEPIG141-11	BOLD:AAW9651	MF547344	658[0n]	2011	Misiones 2
Ortilia ithra	MACN-Bar-Lep-ct 00016	LEPAR010-11	BOLD:AAW9648	MF545544	658[0n]	2010	Buenos Aires 21
Ortilia ithra	MACN-Bar-Lep-ct 00043	LEPAR027-11	BOLD:AAW9648	MF545687	658[3n]	2002	Buenos Aires 10
Ortilia ithra	MACN-Bar-Lep-ct 00068	LEPAR048-11	BOLD:AAW9648	MF546647	658[0n]	2009	Córdoba 1
Ortilia ithra	MACN-Bar-Lep-ct 00128	LEPPA103-11	BOLD:AAW9648	MF546979	658[0n]	2010	Buenos Aires 21
Ortilia ithra	MACN-Bar-Lep-ct 00129	LEPPA104-11	BOLD:AAW9648	MF545918	628[0n]	2010	Buenos Aires 21
Ortilia ithra	MACN-Bar-Lep-ct 00130	LEPPA105-11	BOLD:AAW9648	MF546166	658[0n]	2010	Buenos Aires 21
Ortilia ithra	MACN-Bar-Lep-ct 00161	LEPAR289-11	BOLD:AAW9648	MF545864	658[0n]	2010	Misiones 2
Ortilia ithra	MACN-Bar-Lep-ct 00834	LEPAR609-11	BOLD:AAW9648	MF547209	658[0n]	2010	Misiones 2
Ortilia ithra	MACN-Bar-Lep-ct 00975	LEPAR682-11	BOLD:AAW9648	MF545869	658[0n]	2010	Misiones 2
Ortilia ithra	MACN-Bar-Lep-ct 00999	LEPAR699-11	BOLD:AAW9648	MF547176	658[0n]	2010	Misiones 2
Ortilia ithra	MACN-Bar-Lep-ct 01000	LEPAR700-11	BOLD:AAW9648	MF546105	658[0n]	2010	Misiones 2
Ortilia ithra	MACN-Bar-Lep-ct 01702	LEPAR216-11	BOLD:AAW9648	MF546918	658[0n]	2011	Entre Ríos 4

Ortilia ithra	MACN-Bar-Lep-ct 01815	LEPAR254-11	BOLD:AAW9648	MF546805	658[0n]	2011	Entre Ríos 3
Ortilia ithra	MACN-Bar-Lep-ct 02029	LEPIG140-11	BOLD:AAW9648	MF547114	658[0n]	2011	Misiones 2
Ortilia ithra	MACN-Bar-Lep-ct 02189	LEPIG280-11	BOLD:AAW9648	MF546103	658[0n]	2011	Misiones 3
Ortilia ithra	MACN-Bar-Lep-ct 02334	LEPIG415-11	BOLD:AAW9648	MF546832	658[0n]	2011	Misiones 3
Ortilia ithra	MACN-Bar-Lep-ct 03120	LEPPA043-11			0	2011	Buenos Aires 21
Ortilia ithra	MACN-Bar-Lep-ct 03177	LEPPA080-11	BOLD:AAW9648	MF545460	658[0n]	2011	Buenos Aires 21
Ortilia ithra	MACN-Bar-Lep-ct 03702	LEPPA788-13	BOLD:AAW9648	MF545709	658[0n]	2011	Corrientes 1
Ortilia ithra	MACN-Bar-Lep-ct 03804	LEPPA849-13	BOLD:AAW9648	MF545795	658[0n]	2011	Corrientes 1
Ortilia ithra	MACN-Bar-Lep-ct 03816	LEPPA856-13	BOLD:AAW9648	MF545785	658[0n]	2011	Corrientes 1
Ortilia orthia	MACN-Bar-Lep-ct 00358	LEPAR394-11	BOLD:AAW9643	MF546834	658[0n]	2010	Misiones 2
Ortilia orthia	MACN-Bar-Lep-ct 00600	LEPAR524-11	BOLD:AAW9643	MF545548	658[0n]	2010	Misiones 2
Ortilia orthia	MACN-Bar-Lep-ct 00867	LEPAR636-11	BOLD:AAW9643	MF546376	636[0n]	2010	Misiones 2
Ortilia orthia	MACN-Bar-Lep-ct 00870	LEPAR638-11	BOLD:AAW9643	MF545650	658[0n]	2010	Misiones 2
Ortilia orthia	MACN-Bar-Lep-ct 00992	LEPAR694-11	BOLD:AAW9643	MF545659	658[0n]	2010	Misiones 2
Ortilia velica	MACN-Bar-Lep-ct 00579	LEPAR507-11	BOLD:AAI1940	MF545815	658[0n]	2010	Misiones 2
Ortilia velica	MACN-Bar-Lep-ct 00595	LEPAR520-11	BOLD:AAI1940	MF545855	658[0n]	2010	Misiones 2
Ortilia velica	MACN-Bar-Lep-ct 00848	LEPAR621-11	BOLD:AAI1940	MF546420	658[0n]	2010	Misiones 2
Ortilia velica	MACN-Bar-Lep-ct 00980	LEPAR686-11	BOLD:AAI1940	MF545938	658[0n]	2010	Misiones 2
Ortilia velica	MACN-Bar-Lep-ct 00987	LEPAR691-11	BOLD:AAI1940	MF546281	658[0n]	2010	Misiones 2
Ortilia velica	MACN-Bar-Lep-ct 01500	LEPAR130-11	BOLD:AAI1940	MF547357	658[0n]	2011	Entre Ríos 3
Ortilia velica	MACN-Bar-Lep-ct 01812	LEPAR252-11	BOLD:AAI1940	MF546299	658[0n]	2011	Entre Ríos 3
Ortilia velica	MACN-Bar-Lep-ct 01825	LEPAR259-11	BOLD:AAI1940	MF546397	658[0n]	2011	Entre Ríos 3
Ortilia velica	MACN-Bar-Lep-ct 01854	LEPAR274-11	BOLD:AAI1940	MF546932	658[0n]	2011	Entre Ríos 3
Ouleus fridericus	MACN-Bar-Lep-ct 02006	LEPIG123-11	BOLD:ABZ0178	MF546257	658[0n]	2011	Misiones 2
Ouleus fridericus	MACN-Bar-Lep-ct 02184	LEPIG277-11	BOLD:ABZ0178	MF545416	658[0n]	2011	Misiones 3
Ouleus fridericus	MACN-Bar-Lep-ct 02292	LEPIG377-11	BOLD:ABZ0178	MF545761	658[0n]	2011	Misiones 3
Pampasatyrus gyrtone	MACN-Bar-Lep-ct 00074	LEPAR054-11	BOLD:AAX1950	MF546515	658[0n]	2009	Córdoba 1
Pampasatyrus gyrtone	MACN-Bar-Lep-ct 06469	LEPPA1057-14			0	2013	Córdoba 4
Panoquina ocola	MACN-Bar-Lep-ct 01615	LEPAR177-11	BOLD:AAD3320	MF545912	658[0n]	2011	Entre Ríos 5
Panoquina ocola	MACN-Bar-Lep-ct 02613	LEPPA267-12	BOLD:AAD3320	MF547306	658[0n]	2011	Formosa 2
Panoquina ocola	MACN-Bar-Lep-ct 02753	LEPPA382-12	BOLD:AAD3320	MF547330	658[0n]	2011	Formosa 2
Panoquina ocola	MACN-Bar-Lep-ct 02766	LEPPA392-12	BOLD:AAD3320	MF546373	658[0n]	2011	Formosa 3
Panoquina ocola	MACN-Bar-Lep-ct 02800	LEPPA409-12	BOLD:AAD3320	MF546365	658[0n]	2011	Formosa 2

Panoquina ocola	MACN-Bar-Lep-ct 02847	LEPPA426-12	BOLD:AAD3320	MF545737	651[0n]	2011	Formosa 2
Panoquina ocola	MACN-Bar-Lep-ct 03601	LEPPA724-13	BOLD:AAD3320	MF547370	658[0n]	2011	Corrientes 1
Panoquina ocola	MACN-Bar-Lep-ct 03779	LEPPA834-13	BOLD:AAD3320	MF545982	618[0n]	2011	Corrientes 1
Panoquina ocola	MACN-Bar-Lep-ct 03922	LEPPA550-13	BOLD:AAD3320	MF545978	658[0n]	2012	Formosa 4
Panoquina ocola	MACN-Bar-Lep-ct 03937	LEPPA562-13	BOLD:AAD3320	MF547206	612[0n]	2012	Formosa 4
Panoquina ocola	MACN-Bar-Lep-ct 03939	LEPPA564-13	BOLD:AAD3320	MF546079	622[0n]	2012	Formosa 4
Panoquina ocola	MACN-Bar-Lep-ct 03958	LEPPA580-13	BOLD:AAD3320	MF546094	627[0n]	2012	Formosa 4
Panoquina ocola	MACN-Bar-Lep-ct 06111	LEPAR1044-14	BOLD:AAD3320	MF547167	658[0n]	2013	Formosa 5
Paracarystus hypargira	MACN-Bar-Lep-ct 02157	LEPIG252-11	BOLD:AAV2367	MF547192	658[0n]	2011	Misiones 3
Parcella amarynthina	MACN-Bar-Lep-ct 01043	LEPAR736-11	BOLD:AAZ0332	MF546905	658[0n]	2010	Misiones 2
Parcella amarynthina	MACN-Bar-Lep-ct 01044	LEPAR737-11	BOLD:AAZ0332	MF546384	658[0n]	2010	Misiones 2
Parcella amarynthina	MACN-Bar-Lep-ct 02313	LEPIG397-11	BOLD:AAZ0332	MF545950	658[0n]	2011	Misiones 3
Parcella amarynthina	MACN-Bar-Lep-ct 02508	LEPIG537-11	BOLD:AAZ0332	MF546243	658[0n]	2011	Misiones 3
Pareuptychia summandosa	MACN-Bar-Lep-ct 00348	LEPAR389-11	BOLD:AAA8575	MF547296	658[0n]	2010	Misiones 2
Pareuptychia summandosa	MACN-Bar-Lep-ct 00855	LEPAR624-11	BOLD:AAA8575	MF546058	658[0n]	2010	Misiones 2
Pareuptychia summandosa	MACN-Bar-Lep-ct 01024	LEPAR721-11	BOLD:AAA8575	MF546531	658[0n]	2010	Misiones 2
Pareuptychia summandosa	MACN-Bar-Lep-ct 01386	LEPAR946-11	BOLD:AAA8575	MF546627	658[0n]	2010	Misiones 2
Pareuptychia summandosa	MACN-Bar-Lep-ct 01918	LEPIG043-11	BOLD:AAA8575	MF546448	658[0n]	2011	Misiones 2
Pareuptychia summandosa	MACN-Bar-Lep-ct 01934	LEPIG057-11	BOLD:AAA8575	MF546097	658[0n]	2011	Misiones 2
Pareuptychia summandosa	MACN-Bar-Lep-ct 01989	LEPIG108-11	BOLD:AAA8575	MF546091	658[0n]	2011	Misiones 2
Pareuptychia summandosa	MACN-Bar-Lep-ct 02168	LEPIG263-11	BOLD:AAA8575	MF546187	658[0n]	2011	Misiones 3
Pareuptychia summandosa	MACN-Bar-Lep-ct 02205	LEPIG295-11	BOLD:AAA8575	MF545504	658[0n]	2011	Misiones 3
Pareuptychia summandosa	MACN-Bar-Lep-ct 02242	LEPIG330-11	BOLD:AAA8575	MF545669	658[0n]	2011	Misiones 3
Pareuptychia summandosa	MACN-Bar-Lep-ct 02244	LEPIG332-11	BOLD:AAA8575	MF546139	658[0n]	2011	Misiones 3
Pareuptychia summandosa	MACN-Bar-Lep-ct 02352	LEPIG427-11	BOLD:AAA8575	MF546406	658[0n]	2011	Misiones 3
Pareuptychia summandosa	MACN-Bar-Lep-ct 02405	LEPIG470-11	BOLD:AAA8575	MF546452	658[0n]	2011	Misiones 3
Pareuptychia summandosa	MACN-Bar-Lep-ct 02444	LEPIG498-11	BOLD:AAA8575	MF546559	658[0n]	2011	Misiones 3
Pareuptychia summandosa	MACN-Bar-Lep-ct 02492	LEPIG527-11	BOLD:AAA8575	MF545703	658[0n]	2011	Misiones 3
Pareuptychia summandosa	MACN-Bar-Lep-ct 02504	LEPIG535-11	BOLD:AAA8575	MF546404	658[0n]	2011	Misiones 3
Pareuptychia summandosa	MACN-Bar-Lep-ct 07107	LEPAR1185-15			0	2006	Misiones 7
Pareuptychia summandosa	MACN-Bar-Lep-ct 07109	LEPAR1187-15			0	2004	Misiones 7
Pareuptychia summandosa	MACN-Bar-Lep-ct 07111	LEPAR1189-15			0	2005	Misiones 7
Pareuptychia summandosa	MACN-Bar-Lep-ct 07113	LEPAR1191-15			0	2003	Misiones 7

Parides anchises	MACN-Bar-Lep-ct 00265	LEPAR338-11	BOLD:AAF7591	MF546874	658[0n]	2010	Misiones 2
Parides anchises	MACN-Bar-Lep-ct 02565	LEPIG580-11	BOLD:AAF7591	MF546070	658[0n]	2011	Misiones 3
Parides anchises	MACN-Bar-Lep-ct 06496	LEPPA1084-14			0	2010	Corrientes 4
Parides anchises	MACN-Bar-Lep-ct 06498	LEPPA1086-14			0	2010	Corrientes 4
Parides anchises	MACN-Bar-Lep-ct 06500	LEPPA1088-14			0	2010	Corrientes 4
Parides neophilus	MACN-Bar-Lep-ct 02204	LEPIG294-11	BOLD:AAE7319	MF545571	658[0n]	2011	Misiones 3
Parides neophilus	MACN-Bar-Lep-ct 02254	LEPIG342-11	BOLD:AAE7319	MF546658	658[0n]	2011	Misiones 3
Parides neophilus	MACN-Bar-Lep-ct 02563	LEPIG578-11	BOLD:AAE7319	MF546134	658[0n]	2011	Misiones 3
Parides neophilus	MACN-Bar-Lep-ct 02572	LEPPA227-12	BOLD:AAE7319	MF547151	658[0n]	2011	Formosa 2
Parphorus decora	MACN-Bar-Lep-ct 00423	LEPAR435-11	BOLD:AAZ5148	MF546323	658[0n]	2010	Misiones 2
Parrhasius polibetes	MACN-Bar-Lep-ct 01886	LEPIG014-11	BOLD:AAI4144	MF546558	658[0n]	2011	Misiones 2
Paryphthimoides eous	MACN-Bar-Lep-ct 01277	LEPAR864-11	BOLD:AAZ4049	MF547067	658[0n]	2010	Misiones 2
Paryphthimoides eous	MACN-Bar-Lep-ct 01317	LEPAR899-11	BOLD:AAZ4049	MF545461	650[0n]	2010	Misiones 2
Paryphthimoides eous	MACN-Bar-Lep-ct 01320	LEPAR901-11	BOLD:AAZ4049	MF545549	658[0n]	2010	Misiones 2
Paryphthimoides eous	MACN-Bar-Lep-ct 02243	LEPIG331-11	BOLD:AAZ4049	MF545702	658[0n]	2011	Misiones 3
Paryphthimoides eous	MACN-Bar-Lep-ct 02610	LEPPA265-12	BOLD:AAZ4049	MF547083	658[0n]	2011	Formosa 2
Paryphthimoides eous	MACN-Bar-Lep-ct 02718	LEPPA360-12	BOLD:AAZ4049	MF546574	658[0n]	2011	Formosa 3
Paryphthimoides eous	MACN-Bar-Lep-ct 02724	LEPPA362-12	BOLD:AAZ4049	MF545625	658[0n]	2011	Formosa 3
Paryphthimoides eous	MACN-Bar-Lep-ct 02757	LEPPA386-12	BOLD:AAZ4049	MF545981	658[0n]	2011	Formosa 2
Paryphthimoides eous	MACN-Bar-Lep-ct 02763	LEPPA390-12	BOLD:AAZ4049	MF546945	658[0n]	2011	Formosa 3
Paryphthimoides eous	MACN-Bar-Lep-ct 02775	LEPPA398-12	BOLD:AAZ4049	MF546234	658[0n]	2011	Formosa 2
Paryphthimoides eous	MACN-Bar-Lep-ct 02854	LEPPA430-12	BOLD:AAZ4049	MF545796	658[0n]	2011	Formosa 2
Paryphthimoides eous	MACN-Bar-Lep-ct 02857	LEPPA432-12	BOLD:AAZ4049	MF546379	658[0n]	2011	Formosa 2
Paryphthimoides eous	MACN-Bar-Lep-ct 03725	LEPPA801-13	BOLD:AAZ4049	MF545760	658[0n]	2011	Corrientes 1
Paryphthimoides eous	MACN-Bar-Lep-ct 03727	LEPPA803-13	BOLD:AAZ4049	MF546778	658[0n]	2011	Corrientes 1
Paryphthimoides eous	MACN-Bar-Lep-ct 03756	LEPPA824-13	BOLD:AAZ4049	MF546790	658[0n]	2011	Corrientes 1
Paryphthimoides eous	MACN-Bar-Lep-ct 03780	LEPPA835-13	BOLD:AAZ4049	MF547137	658[0n]	2011	Corrientes 1
Paryphthimoides eous	MACN-Bar-Lep-ct 03824	LEPPA860-13	BOLD:AAZ4049	MF545969	658[0n]	2011	Corrientes 1
Paryphthimoides phronius	MACN-Bar-Lep-ct 01496	LEPAR127-11	BOLD:AAX1665	MF547249	658[0n]	2011	Entre Ríos 3
Paryphthimoides phronius	MACN-Bar-Lep-ct 01594	LEPAR167-11	BOLD:AAX1665	MF546570	658[0n]	2011	Entre Ríos 3
Paryphthimoides phronius	MACN-Bar-Lep-ct 01821	LEPAR258-11	BOLD:AAX1665	MF545736	658[0n]	2011	Entre Ríos 3
Paryphthimoides phronius	MACN-Bar-Lep-ct 01858	LEPAR276-11	BOLD:AAX1665	MF545575	658[0n]	2011	Entre Ríos 3
Paryphthimoides phronius	MACN-Bar-Lep-ct 01859	LEPAR277-11	BOLD:AAX1665	MF546019	658[0n]	2011	Entre Ríos 3

Paryphthimoides poltys	MACN-Bar-Lep-ct 01491	LEPAR125-11	BOLD:AAX1664	MF546731	658[0n]	2011	Entre Ríos 3
Paryphthimoides poltys	MACN-Bar-Lep-ct 01810	LEPAR250-11	BOLD:AAX1664	MF545662	658[0n]	2011	Entre Ríos 3
Paryphthimoides poltys	MACN-Bar-Lep-ct 01827	LEPAR260-11	BOLD:AAX1664	MF547134	658[0n]	2011	Entre Ríos 3
Paryphthimoides poltys	MACN-Bar-Lep-ct 03111	LEPPA034-11			0	2010	Buenos Aires 21
Paryphthimoides poltys	MACN-Bar-Lep-ct 03114	LEPPA037-11			0	2011	Buenos Aires 21
Paryphthimoides poltys	MACN-Bar-Lep-ct 03131	LEPPA052-11	BOLD:AAX1664	MF546123	658[0n]	2011	Buenos Aires 21
Paryphthimoides poltys	MACN-Bar-Lep-ct 03717	LEPPA798-13	BOLD:AAX1664	MF546205	658[0n]	2011	Corrientes 1
Paryphthimoides sp. 1	MACN-Bar-Lep-ct 07135	LEPAR1213-15			0	2004	Misiones 7
Paryphthimoides sp. 1	MACN-Bar-Lep-ct 07137	LEPAR1215-15			0	2010	Misiones 15
Paulogramma pygas	MACN-Bar-Lep-ct 00330	LEPAR377-11	BOLD:ACE6723	MF545577	658[0n]	2010	Misiones 2
Paulogramma pygas	MACN-Bar-Lep-ct 02210	LEPIG299-11	BOLD:ACE6723	MF547018	658[0n]	2011	Misiones 3
Paulogramma pygas	MACN-Bar-Lep-ct 02284	LEPIG370-11	BOLD:ACE6723	MF546579	658[0n]	2011	Misiones 3
Paulogramma pygas	MACN-Bar-Lep-ct 02300	LEPIG385-11	BOLD:ACE6722	MF545611	658[0n]	2011	Misiones 3
Paulogramma pygas	MACN-Bar-Lep-ct 02311	LEPIG395-11	BOLD:ACE6722	MF545831	650[0n]	2011	Misiones 3
Paulogramma pygas	MACN-Bar-Lep-ct 02321	LEPIG403-11	BOLD:ACE6723	MF547284	658[0n]	2011	Misiones 3
Paulogramma pygas	MACN-Bar-Lep-ct 02470	LEPIG513-11	BOLD:ACE6723	MF546400	658[0n]	2011	Misiones 3
Paulogramma pygas	MACN-Bar-Lep-ct 02521	LEPIG543-11	BOLD:ACE6723	MF546973	658[0n]	2011	Misiones 3
Paulogramma pygas	MACN-Bar-Lep-ct 07085	LEPAR1163-15			0	2005	Misiones 7
Paulogramma pygas	MACN-Bar-Lep-ct 07087	LEPAR1165-15			0	2002	Misiones 7
Paulogramma pygas	MACN-Bar-Lep-ct 07089	LEPAR1167-15	COI < 500 bp		187[0n]	2005	Misiones 7
Paulogramma pyracmon	MACN-Bar-Lep-ct 00542	LEPAR475-11	BOLD:AAZ4670	MF547196	658[0n]	2010	Misiones 2
Paulogramma pyracmon	MACN-Bar-Lep-ct 01315	LEPAR897-11	BOLD:AAZ4670	MF547340	658[0n]	2010	Misiones 2
Paulogramma pyracmon	MACN-Bar-Lep-ct 01896	LEPIG024-11	BOLD:AAZ4670	MF547223	658[0n]	2011	Misiones 2
Paulogramma pyracmon	MACN-Bar-Lep-ct 02085	LEPIG189-11	BOLD:AAZ4670	MF547390	658[0n]	2011	Misiones 2
Paulogramma pyracmon	MACN-Bar-Lep-ct 02098	LEPIG201-11	BOLD:AAZ4670	MF546412	658[0n]	2011	Misiones 2
Paulogramma pyracmon	MACN-Bar-Lep-ct 02113	LEPIG212-11	BOLD:AAZ4670	MF547164	650[0n]	2011	Misiones 2
Pellicia costimacula	MACN-Bar-Lep-ct 01227	LEPAR842-11	BOLD:AAB5745	MF545971	658[0n]	2010	Misiones 2
Pellicia costimacula	MACN-Bar-Lep-ct 02081	LEPIG185-11	BOLD:AAB5745	MF546748	658[0n]	2011	Misiones 2
Pellicia costimacula	MACN-Bar-Lep-ct 02429	LEPIG488-11	BOLD:AAB5745	MF545769	658[0n]	2011	Misiones 3
Pellicia najoides	MACN-Bar-Lep-ct 00576	LEPAR504-11	BOLD:AAZ4688	MF547051	658[0n]	2010	Misiones 2
Perichares lotus	MACN-Bar-Lep-ct 02126	LEPIG223-11	BOLD:AAC6710	MF546728	658[0n]	2011	Misiones 2
Perichares lotus	MACN-Bar-Lep-ct 02375	LEPIG444-11	BOLD:AAC6710	MF546675	658[0n]	2011	Misiones 3
Perichares philetes	MACN-Bar-Lep-ct 02062	LEPIG169-11	BOLD:AAA2307	MF546593	658[0n]	2011	Misiones 2

Pharneuptychia phares	MACN-Bar-Lep-ct 02692	LEPPA341-12	BOLD:ACA8888	MF546786	658[0n]	2011	Formosa 2
Pharneuptychia phares	MACN-Bar-Lep-ct 03715	LEPPA796-13	BOLD:ACA8888	MF545685	658[0n]	2011	Corrientes 1
Pharneuptychia phares	MACN-Bar-Lep-ct 03784	LEPPA838-13	BOLD:ACA8888	MF546155	658[0n]	2011	Corrientes 1
Pharneuptychia phares	MACN-Bar-Lep-ct 03795	LEPPA844-13	BOLD:ACA8888	MF545525	658[0n]	2011	Corrientes 1
Pharneuptychia phares	MACN-Bar-Lep-ct 03840	LEPPA868-13	BOLD:ACA8888	MF545962	658[0n]	2011	Corrientes 1
Pharneuptychia phares	MACN-Bar-Lep-ct 03874	LEPPA516-13	BOLD:ACA8888	MF546275	658[0n]	2012	Formosa 4
Pharneuptychia phares	MACN-Bar-Lep-ct 03906	LEPPA538-13	BOLD:ACA8888	MF547093	658[0n]	2012	Formosa 4
Phemiades pohli	MACN-Bar-Lep-ct 01352	LEPAR933-11	BOLD:AAZ5079	MF546388	658[0n]	2010	Misiones 2
Pheraeus perpulcher	MACN-Bar-Lep-ct 01341	LEPAR922-11	BOLD:AAZ5080	MF545453	658[0n]	2010	Misiones 2
Phocides charon	MACN-Bar-Lep-ct 00164	LEPAR291-11	BOLD:AAZ1533	MF547310	658[0n]	2010	Misiones 2
Phocides charon	MACN-Bar-Lep-ct 00824	LEPAR600-11	BOLD:AAZ1533	MF546664	658[0n]	2010	Misiones 2
Phocides charon	MACN-Bar-Lep-ct 00997	LEPAR698-11	BOLD:AAZ1533	MF546915	658[0n]	2010	Misiones 2
Phocides polybius	MACN-Bar-Lep-ct 00153	LEPPA128-11	BOLD:AAE6688	MF546122	658[0n]	2010	Buenos Aires 7
Phocides polybius	MACN-Bar-Lep-ct 03647	LEPPA749-13	BOLD:AAE6688	MF547257	658[0n]	2011	Corrientes 1
Phocides polybius	MACN-Bar-Lep-ct 06034	LEPAR967-14	BOLD:AAE6688	MF546172	658[0n]	2013	Formosa 5
Phoebis argante	MACN-Bar-Lep-ct 00340	LEPAR382-11	BOLD:ACE3986	MF547294	658[0n]	2010	Misiones 2
Phoebis argante	MACN-Bar-Lep-ct 00341	LEPAR383-11	BOLD:ACE3986	MF546917	658[0n]	2010	Misiones 2
Phoebis argante	MACN-Bar-Lep-ct 00564	LEPAR492-11	BOLD:ACE3986	MF546836	658[0n]	2010	Misiones 2
Phoebis argante	MACN-Bar-Lep-ct 00566	LEPAR494-11	BOLD:ACE3986	MF546182	658[0n]	2010	Misiones 2
Phoebis argante	MACN-Bar-Lep-ct 00976	LEPAR683-11	BOLD:ACE3986	MF546100	658[0n]	2010	Misiones 2
Phoebis argante	MACN-Bar-Lep-ct 02297	LEPIG382-11	BOLD:ACE3986	MF545431	658[0n]	2011	Misiones 3
Phoebis argante	MACN-Bar-Lep-ct 02303	LEPIG387-11	BOLD:ACE3991	MF546809	658[0n]	2011	Misiones 3
Phoebis argante	MACN-Bar-Lep-ct 02314	LEPIG398-11	BOLD:ACE3991	MF545477	650[0n]	2011	Misiones 3
Phoebis argante	MACN-Bar-Lep-ct 02399	LEPIG464-11	BOLD:ACE3986	MF546571	658[0n]	2011	Misiones 3
Phoebis argante	MACN-Bar-Lep-ct 02414	LEPIG478-11	BOLD:ACE3986	MF546245	658[0n]	2011	Misiones 3
Phoebis neocypris	MACN-Bar-Lep-ct 00077	LEPAR057-11	BOLD:ABZ0840	MF545537	658[0n]	2008	Misiones 1
Phoebis neocypris	MACN-Bar-Lep-ct 00078	LEPAR058-11	BOLD:ABZ0840	MF546271	658[0n]	2008	Misiones 1
Phoebis neocypris	MACN-Bar-Lep-ct 00079	LEPAR059-11	BOLD:ABZ0840	MF546758	658[0n]	2008	Misiones 1
Phoebis neocypris	MACN-Bar-Lep-ct 00080	LEPAR060-11	BOLD:ABZ0840	MF546550	658[0n]	2008	Misiones 1
Phoebis neocypris	MACN-Bar-Lep-ct 00081	LEPAR061-11	BOLD:ABZ0840	MF546078	658[0n]	2008	Misiones 1
Phoebis neocypris	MACN-Bar-Lep-ct 00082	LEPAR062-11	BOLD:ABZ0840	MF546152	658[0n]	2008	Misiones 1
Phoebis neocypris	MACN-Bar-Lep-ct 00255	LEPAR334-11	BOLD:ABZ0840	MF545991	658[0n]	2010	Misiones 2
Phoebis neocypris	MACN-Bar-Lep-ct 00257	LEPAR335-11	BOLD:ABZ0840	MF545388	658[0n]	2010	Misiones 2

Phoebis neocypris	MACN-Bar-Lep-ct 00282	LEPAR346-11	BOLD:ABZ0840	MF547250	658[0n]	2010	Misiones 2
Phoebis neocypris	MACN-Bar-Lep-ct 00310	LEPAR365-11	BOLD:ABZ0840	MF546891	658[0n]	2010	Misiones 2
Phoebis neocypris	MACN-Bar-Lep-ct 00339	LEPAR381-11	BOLD:ABZ0840	MF545506	658[0n]	2010	Misiones 2
Phoebis neocypris	MACN-Bar-Lep-ct 02607	LEPPA262-12	BOLD:ABZ0840	MF547232	658[0n]	2011	Formosa 2
Phoebis neocypris	MACN-Bar-Lep-ct 02827	LEPPA417-12	BOLD:ABZ0840	MF545648	658[0n]	2011	Formosa 2
Phoebis neocypris	MACN-Bar-Lep-ct 02849	LEPPA427-12	BOLD:ABZ0840	MF545818	658[0n]	2011	Formosa 2
Phoebis neocypris	MACN-Bar-Lep-ct 03931	LEPPA557-13	BOLD:ABZ0840	MF545915	622[0n]	2012	Formosa 4
Phoebis sennae	MACN-Bar-Lep-ct 01525	LEPAR142-11	BOLD:AAA7410	MF545941	658[0n]	2011	Entre Ríos 4
Phoebis sennae	MACN-Bar-Lep-ct 01528	LEPAR144-11	BOLD:AAA7410	MF546724	658[0n]	2011	Entre Ríos 4
Phoebis sennae	MACN-Bar-Lep-ct 01534	LEPAR146-11	BOLD:AAA7410	MF545782	658[0n]	2011	Entre Ríos 4
Phoebis sennae	MACN-Bar-Lep-ct 01538	LEPAR149-11	BOLD:AAA7410	MF546393	658[0n]	2011	Entre Ríos 4
Phoebis sennae	MACN-Bar-Lep-ct 01901	LEPIG029-11	BOLD:AAA7410	MF546960	658[0n]	2011	Misiones 2
Phoebis sennae	MACN-Bar-Lep-ct 02579	LEPPA234-12	BOLD:AAA7410	MF547288	658[0n]	2011	Formosa 2
Phoebis sennae	MACN-Bar-Lep-ct 02589	LEPPA244-12	BOLD:AAA7410	MF546013	658[0n]	2011	Formosa 2
Phoebis sennae	MACN-Bar-Lep-ct 02595	LEPPA250-12	BOLD:AAA7410	MF546024	658[0n]	2011	Formosa 2
Phoebis sennae	MACN-Bar-Lep-ct 02621	LEPPA275-12	BOLD:AAA7410	MF546231	658[0n]	2011	Formosa 2
Phoebis sennae	MACN-Bar-Lep-ct 03871	LEPPA515-13	BOLD:AAA7410	MF546255	658[0n]	2012	Formosa 4
Phoebis sennae	MACN-Bar-Lep-ct 03945	LEPPA569-13	BOLD:AAA7410	MF545814	658[0n]	2012	Formosa 4
Phoebis sennae	MACN-Bar-Lep-ct 06028	LEPAR961-14	BOLD:AAA7410	MF546674	658[2n]	2013	Formosa 5
Phoebis sennae	MACN-Bar-Lep-ct 06067	LEPAR1000-14	BOLD:AAA7410	MF547337	658[0n]	2013	Formosa 5
Phoebis sennae	MACN-Bar-Lep-ct 06087	LEPAR1020-14	BOLD:AAA7410	MF546863	658[0n]	2013	Formosa 5
Phystis simois	MACN-Bar-Lep-ct 00093	LEPAR071-11	BOLD:AAY7587	MF546812	658[0n]	2010	Córdoba 1
Phystis simois	MACN-Bar-Lep-ct 06064	LEPAR997-14	BOLD:AAY7587	MF546137	658[4n]	2013	Formosa 5
Polites vibex	MACN-Bar-Lep-ct 00134	LEPPA109-11	BOLD:ACE5718	MF547235	658[0n]	2010	Buenos Aires 21
Polites vibex	MACN-Bar-Lep-ct 01625	LEPAR181-11	BOLD:ACE5718	MF546660	658[0n]	2011	Entre Ríos 4
Polites vibex	MACN-Bar-Lep-ct 01629	LEPAR183-11	BOLD:ACE5718	MF545874	658[0n]	2011	Entre Ríos 4
Polites vibex	MACN-Bar-Lep-ct 01653	LEPAR196-11	BOLD:ACE5718	MF545442	658[0n]	2011	Entre Ríos 4
Polites vibex	MACN-Bar-Lep-ct 01715	LEPAR220-11	BOLD:ACE5718	MF546301	658[0n]	2011	Entre Ríos 4
Polites vibex	MACN-Bar-Lep-ct 03124	LEPPA047-11			0	2011	Buenos Aires 21
Polites vibex	MACN-Bar-Lep-ct 03139	LEPPA059-11	BOLD:ACE5718	MF546765	658[0n]	2011	Buenos Aires 21
Polites vibex	MACN-Bar-Lep-ct 03142	LEPPA061-11	BOLD:ABX5388	MF545851	658[0n]	2011	Buenos Aires 21
Polites vibex	MACN-Bar-Lep-ct 03707	LEPPA791-13	BOLD:ACE5718	MF545596	658[0n]	2011	Corrientes 1
Polites vibex	MACN-Bar-Lep-ct 03716	LEPPA797-13	BOLD:ABX5388	MF546395	658[0n]	2011	Corrientes 1

Polites vibex	MACN-Bar-Lep-ct 03810	LEPPA853-13	BOLD:ACE5718	MF546286	658[0n]	2011	Corrientes 1
Polites vibex	MACN-Bar-Lep-ct 03941	LEPPA566-13	BOLD:ACE5718	MF546506	658[0n]	2012	Formosa 4
Polites vibex	MACN-Bar-Lep-ct 03949	LEPPA572-13	BOLD:ACE5718	MF545552	658[0n]	2012	Formosa 4
Polites vibex	MACN-Bar-Lep-ct 06502	LEPPA1090-14			0	2010	Corrientes 3
Polythrix caunus	MACN-Bar-Lep-ct 01292	LEPAR877-11	BOLD:AAZ4927	MF546801	658[0n]	2010	Misiones 2
Polythrix octomaculata	MACN-Bar-Lep-ct 02332	LEPIG413-11	BOLD:AAZ7430	MF547101	658[0n]	2011	Misiones 3
Polythrix octomaculata	MACN-Bar-Lep-ct 02425	LEPIG487-11	BOLD:AAZ7430	MF546766	658[0n]	2011	Misiones 3
Polythrix octomaculata	MACN-Bar-Lep-ct 02432	LEPIG491-11	BOLD:AAZ7430	MF545683	658[0n]	2011	Misiones 3
Pompeius amblyspila	MACN-Bar-Lep-ct 03637	LEPPA743-13	BOLD:ACG2606	MF545865	658[0n]	2011	Corrientes 1
Pompeius amblyspila	MACN-Bar-Lep-ct 03737	LEPPA812-13	BOLD:ACG2606	MF546295	658[0n]	2011	Corrientes 1
Pompeius amblyspila	MACN-Bar-Lep-ct 03828	LEPPA864-13	BOLD:ACG2606	MF546656	603[0n]	2011	Corrientes 1
Pompeius amblyspila	MACN-Bar-Lep-ct 03843	LEPPA870-13	BOLD:ACG2606	MF546433	618[0n]	2011	Corrientes 1
Pompeius dares	MACN-Bar-Lep-ct 01762	LEPAR240-11	BOLD:AAZ9013	MF545959	658[0n]	2011	Entre Ríos 6
Pompeius dares	MACN-Bar-Lep-ct 01766	LEPAR243-11	BOLD:AAZ9013	MF547297	658[0n]	2011	Entre Ríos 6
Pompeius pompeius	MACN-Bar-Lep-ct 00554	LEPAR483-11	BOLD:AAD3691	MF545566	658[0n]	2010	Misiones 2
Pompeius pompeius	MACN-Bar-Lep-ct 02220	LEPIG309-11	BOLD:AAD3691	MF546169	658[0n]	2011	Misiones 3
Praepedaliodes phanias	MACN-Bar-Lep-ct 00578	LEPAR506-11	BOLD:AAY3328	MF546046	636[0n]	2010	Misiones 2
Praepedaliodes phanias	MACN-Bar-Lep-ct 01011	LEPAR710-11	BOLD:AAY3328	MF545651	658[0n]	2010	Misiones 2
Praepedaliodes phanias	MACN-Bar-Lep-ct 01025	LEPAR722-11	BOLD:AAY3328	MF546081	658[0n]	2010	Misiones 2
Praepedaliodes phanias	MACN-Bar-Lep-ct 01143	LEPAR776-11	BOLD:AAY3328	MF547016	658[0n]	2010	Misiones 2
Praepedaliodes phanias	MACN-Bar-Lep-ct 01210	LEPAR828-11	BOLD:AAY3328	MF547133	658[0n]	2010	Misiones 2
Praepedaliodes phanias	MACN-Bar-Lep-ct 02162	LEPIG257-11	BOLD:AAY3328	MF545894	658[0n]	2011	Misiones 3
Praepedaliodes phanias	MACN-Bar-Lep-ct 02197	LEPIG287-11	BOLD:AAY3328	MF546556	658[0n]	2011	Misiones 3
Propertius propertius	MACN-Bar-Lep-ct 00183	LEPAR301-11	BOLD:AAZ4628	MF545867	658[0n]	2010	Misiones 2
Propertius propertius	MACN-Bar-Lep-ct 01286	LEPAR872-11	BOLD:AAZ4628	MF546643	658[0n]	2010	Misiones 2
Protesilaus helios	MACN-Bar-Lep-ct 07063	LEPAR1141-15			0	2007	Misiones 10
Pseudodebis euptychidia	MACN-Bar-Lep-ct 00529	LEPAR466-11	BOLD:AAZ1821	MF545476	658[0n]	2010	Misiones 2
Pseudodebis euptychidia	MACN-Bar-Lep-ct 01141	LEPAR775-11	BOLD:AAZ1821	MF545433	658[0n]	2010	Misiones 2
Pseudodebis euptychidia	MACN-Bar-Lep-ct 01168	LEPAR794-11	BOLD:AAZ1821	MF546443	658[0n]	2010	Misiones 2
Pseudodebis euptychidia	MACN-Bar-Lep-ct 01186	LEPAR808-11	BOLD:AAZ1821	MF547277	658[0n]	2010	Misiones 2
Pseudodebis euptychidia	MACN-Bar-Lep-ct 01223	LEPAR838-11	BOLD:AAZ1821	MF546366	658[0n]	2010	Misiones 2
Pseudodebis euptychidia	MACN-Bar-Lep-ct 01278	LEPAR865-11	BOLD:AAZ1821	MF546565	658[0n]	2010	Misiones 2
Pseudolycaena marsyas	MACN-Bar-Lep-ct 02498	LEPIG531-11	BOLD:AAJ9471	MF546068	658[1n]	2011	Misiones 3

Pseudolycaena marsyas	MACN-Bar-Lep-ct 02628	LEPPA282-12	BOLD:AAJ9471	MF545797	658[0n]	2011	Formosa 2
Pseudolycaena marsyas	MACN-Bar-Lep-ct 02830	LEPPA419-12	BOLD:AAJ9471	MF545397	658[0n]	2011	Formosa 2
Pseudolycaena marsyas	MACN-Bar-Lep-ct 03739	LEPPA814-13	BOLD:AAJ9471	MF546069	658[0n]	2011	Corrientes 1
Pseudopieris nehemia	MACN-Bar-Lep-ct 00252	LEPAR333-11	BOLD:AAZ3974	MF547352	658[0n]	2010	Misiones 2
Pseudopieris nehemia	MACN-Bar-Lep-ct 00359	LEPAR395-11	BOLD:AAZ3974	MF546246	658[0n]	2010	Misiones 2
Pseudopieris nehemia	MACN-Bar-Lep-ct 00361	LEPAR397-11	BOLD:AAZ3974	MF546810	658[0n]	2010	Misiones 2
Pseudopieris nehemia	MACN-Bar-Lep-ct 00796	LEPAR577-11	BOLD:AAZ3974	MF545438	658[0n]	2010	Misiones 2
Pseudopieris nehemia	MACN-Bar-Lep-ct 00826	LEPAR602-11	BOLD:AAZ3974	MF546267	658[0n]	2010	Misiones 2
Pseudopieris nehemia	MACN-Bar-Lep-ct 01921	LEPIG045-11	BOLD:AAZ3974	MF545505	658[0n]	2011	Misiones 2
Pseudopieris nehemia	MACN-Bar-Lep-ct 01937	LEPIG060-11	BOLD:AAZ3974	MF545812	658[0n]	2011	Misiones 2
Pseudopieris nehemia	MACN-Bar-Lep-ct 02379	LEPIG447-11	BOLD:AAZ3974	MF546679	658[0n]	2011	Misiones 3
Pseudoscada erruca	MACN-Bar-Lep-ct 02017	LEPIG130-11	BOLD:AAZ8162	MF546940	658[0n]	2011	Misiones 2
Pseudoscada erruca	MACN-Bar-Lep-ct 02095	LEPIG198-11	BOLD:AAZ8162	MF545619	658[0n]	2011	Misiones 2
Pseudoscada erruca	MACN-Bar-Lep-ct 02268	LEPIG356-11	BOLD:AAZ8162	MF546900	658[0n]	2011	Misiones 3
Pteronymia carlia	MACN-Bar-Lep-ct 02040	LEPIG149-11	BOLD:AAZ8197	MF545594	658[0n]	2011	Misiones 2
Pteronymia carlia	MACN-Bar-Lep-ct 02781	LEPPA399-12	BOLD:AAZ8197	MF546236	658[0n]	2011	Formosa 2
Pyrgus orcus	MACN-Bar-Lep-ct 00166	LEPAR293-11	BOLD:AAG4994	MF546444	658[0n]	2010	Misiones 2
Pyrgus orcus	MACN-Bar-Lep-ct 00179	LEPAR299-11	BOLD:AAG4994	MF547402	658[0n]	2010	Misiones 2
Pyrgus orcus	MACN-Bar-Lep-ct 01480	LEPAR116-11	BOLD:AAG4994	MF545857	658[0n]	2011	Entre Ríos 3
Pyrgus orcus	MACN-Bar-Lep-ct 01488	LEPAR122-11	BOLD:AAG4994	MF546580	658[0n]	2011	Entre Ríos 3
Pyrgus orcus	MACN-Bar-Lep-ct 01587	LEPAR164-11	BOLD:AAG4994	MF546014	658[0n]	2011	Entre Ríos 3
Pyrgus orcus	MACN-Bar-Lep-ct 01588	LEPAR165-11	BOLD:AAG4994	MF547401	658[0n]	2011	Entre Ríos 3
Pyrgus orcus	MACN-Bar-Lep-ct 01925	LEPIG049-11	BOLD:AAG4994	MF545497	658[0n]	2011	Misiones 2
Pyrgus orcus	MACN-Bar-Lep-ct 01932	LEPIG055-11	BOLD:AAG4994	MF546630	658[0n]	2011	Misiones 2
Pyrgus orcus	MACN-Bar-Lep-ct 01944	LEPIG066-11	BOLD:AAG4994	MF546509	658[0n]	2011	Misiones 2
Pyrgus orcus	MACN-Bar-Lep-ct 02011	LEPIG126-11	BOLD:AAG4994	MF547260	658[0n]	2011	Misiones 2
Pyrgus orcus	MACN-Bar-Lep-ct 02037	LEPIG147-11	BOLD:AAG4994	MF546032	658[0n]	2011	Misiones 2
Pyrgus orcus	MACN-Bar-Lep-ct 02041	LEPIG150-11	BOLD:AAG4994	MF545639	658[0n]	2011	Misiones 2
Pyrgus orcus	MACN-Bar-Lep-ct 02251	LEPIG339-11	BOLD:AAG4994	MF546853	658[0n]	2011	Misiones 3
Pyrgus orcus	MACN-Bar-Lep-ct 02652	LEPPA305-12			0	2011	Formosa 2
Pyrgus orcus	MACN-Bar-Lep-ct 02733	LEPPA368-12	BOLD:AAG4994	MF547023	658[0n]	2011	Formosa 3
Pyrgus orcus	MACN-Bar-Lep-ct 02764	LEPPA391-12	BOLD:AAG4994	MF546335	658[0n]	2011	Formosa 3
Pyrgus orcus	MACN-Bar-Lep-ct 03098	LEPPA021-11	BOLD:AAG4994	MF545603	658[0n]	2011	Córdoba 2

Pyrgus orcus	MACN-Bar-Lep-ct 03569	LEPPA702-13	BOLD:AAG4994	MF546567	606[0n]	2011	Corrientes 1
Pyrgus orcus	MACN-Bar-Lep-ct 03796	LEPPA845-13	BOLD:AAG4994	MF546847	658[0n]	2011	Corrientes 1
Pyrgus orcus	MACN-Bar-Lep-ct 03847	LEPPA873-13	BOLD:AAG4994	MF546781	658[0n]	2011	Corrientes 1
Pyrgus orcus	MACN-Bar-Lep-ct 03909	LEPPA539-13	BOLD:AAG4994	MF546610	658[0n]	2012	Formosa 4
Pyrgus orcus	MACN-Bar-Lep-ct 03960	LEPPA582-13	BOLD:AAG4994	MF545862	658[0n]	2012	Formosa 4
Pyrgus orcus	MACN-Bar-Lep-ct 03972	LEPPA590-13	BOLD:AAG4994	MF547187	658[0n]	2012	Formosa 4
Pyrgus orcynoides	MACN-Bar-Lep-ct 01018	LEPAR715-11	BOLD:AAZ1471	MF546423	658[0n]	2010	Misiones 2
Pyrgus orcynoides	MACN-Bar-Lep-ct 01478	LEPAR114-11	BOLD:AAZ1471	MF547378	658[0n]	2011	Entre Ríos 3
Pyrgus orcynoides	MACN-Bar-Lep-ct 01479	LEPAR115-11	BOLD:AAZ1471	MF547136	658[0n]	2011	Entre Ríos 3
Pyrgus orcynoides	MACN-Bar-Lep-ct 01489	LEPAR123-11	BOLD:AAZ1471	MF546704	658[0n]	2011	Entre Ríos 3
Pyrgus orcynoides	MACN-Bar-Lep-ct 02637	LEPPA290-12	BOLD:AAZ1471	MF546220	658[0n]	2011	Formosa 2
Pyrgus orcynoides	MACN-Bar-Lep-ct 02708	LEPPA351-12	BOLD:AAZ1471	MF546394	658[0n]	2011	Formosa 3
Pyrgus orcynoides	MACN-Bar-Lep-ct 02762	LEPPA389-12	BOLD:AAZ1471	MF546136	658[0n]	2011	Formosa 3
Pyrgus orcynoides	MACN-Bar-Lep-ct 03096	LEPPA019-11	BOLD:AAZ1471	MF546665	658[0n]	2011	Córdoba 2
Pyrgus orcynoides	MACN-Bar-Lep-ct 03121	LEPPA044-11			0	2011	Buenos Aires 21
Pyrgus orcynoides	MACN-Bar-Lep-ct 03130	LEPPA051-11	BOLD:AAZ1471	MF545721	658[0n]	2011	Buenos Aires 21
Pyrgus orcynoides	MACN-Bar-Lep-ct 03159	LEPPA074-11	BOLD:AAZ1471	MF546461	658[0n]	2011	Buenos Aires 21
Pyrgus orcynoides	MACN-Bar-Lep-ct 03174	LEPPA079-11	BOLD:AAZ1471	MF546035	658[0n]	2011	Buenos Aires 21
Pyrgus orcynoides	MACN-Bar-Lep-ct 03595	LEPPA720-13	BOLD:AAZ1471	MF545986	658[0n]	2011	Corrientes 1
Pyrgus orcynoides	MACN-Bar-Lep-ct 03692	LEPPA779-13	BOLD:AAZ1471	MF546796	658[0n]	2011	Corrientes 1
Pyrgus orcynoides	MACN-Bar-Lep-ct 03728	LEPPA804-13	BOLD:AAZ1471	MF546914	658[0n]	2011	Corrientes 1
Pyrgus orcynoides	MACN-Bar-Lep-ct 03876	LEPPA518-13	BOLD:AAZ1471	MF546238	658[0n]	2012	Formosa 4
Pyrgus orcynoides	MACN-Bar-Lep-ct 03888	LEPPA527-13	BOLD:AAZ1471	MF546521	613[0n]	2012	Formosa 4
Pyrgus orcynoides	MACN-Bar-Lep-ct 03897	LEPPA531-13	BOLD:AAZ1471	MF545427	658[0n]	2012	Formosa 4
Pyrgus orcynoides	MACN-Bar-Lep-ct 06112	LEPAR1045-14	BOLD:AAZ1471	MF546760	658[0n]	2013	Formosa 5
Pyrisitia leuce	MACN-Bar-Lep-ct 00552	LEPAR482-11	BOLD:AAU0077	MF546489	642[0n]	2010	Misiones 2
Pyrisitia leuce	MACN-Bar-Lep-ct 02216	LEPIG305-11	BOLD:AAU0077	MF547143	658[0n]	2011	Misiones 3
Pyrisitia leuce	MACN-Bar-Lep-ct 02219	LEPIG308-11	BOLD:AAU0077	MF546052	658[0n]	2011	Misiones 3
Pyrisitia leuce	MACN-Bar-Lep-ct 02296	LEPIG381-11	BOLD:AAU0077	MF547070	658[0n]	2011	Misiones 3
Pyrisitia leuce	MACN-Bar-Lep-ct 02433	LEPIG492-11	BOLD:AAU0077	MF546473	658[0n]	2011	Misiones 3
Pyrisitia leuce	MACN-Bar-Lep-ct 02487	LEPIG525-11	BOLD:AAU0077	MF545944	658[0n]	2011	Misiones 3
Pyrisitia leuce	MACN-Bar-Lep-ct 02501	LEPIG533-11	BOLD:AAU0077	MF545974	658[0n]	2011	Misiones 3
Pyrisitia leuce	MACN-Bar-Lep-ct 02528	LEPIG550-11	BOLD:AAU0077	MF545848	658[0n]	2011	Misiones 3

Pyrisitia leuce	MACN-Bar-Lep-ct 02544	LEPIG562-11	BOLD:AAU0077	MF545573	658[0n]	2011	Misiones 3
Pyrisitia leuce	MACN-Bar-Lep-ct 02742	LEPPA374-12	BOLD:AAU0077	MF546865	658[0n]	2011	Formosa 3
Pyrisitia leuce	MACN-Bar-Lep-ct 02747	LEPPA378-12	BOLD:AAU0077	MF546282	658[0n]	2011	Formosa 3
Pyrisitia leuce	MACN-Bar-Lep-ct 06031	LEPAR964-14	BOLD:AAU0077	MF547350	658[2n]	2013	Formosa 5
Pyrisitia leuce	MACN-Bar-Lep-ct 07077	LEPAR1155-15			0	2003	Misiones 7
Pyrisitia leuce	MACN-Bar-Lep-ct 07079	LEPAR1157-15			0	2002	Misiones 7
Pyrisitia leuce	MACN-Bar-Lep-ct 07081	LEPAR1159-15	BOLD:AAU0077	MF546850	564[0n]	2010	Misiones 14
Pyrisitia nise	MACN-Bar-Lep-ct 01466	LEPAR108-11	BOLD:AAZ7347	MF545877	658[0n]	2011	Entre Ríos 3
Pyrisitia nise	MACN-Bar-Lep-ct 01533	LEPAR145-11	BOLD:AAZ7347	MF547380	658[0n]	2011	Entre Ríos 4
Pyrisitia nise	MACN-Bar-Lep-ct 01535	LEPAR147-11	BOLD:AAZ7347	MF546023	658[0n]	2011	Entre Ríos 4
Pyrisitia nise	MACN-Bar-Lep-ct 01541	LEPAR150-11	BOLD:AAZ7347	MF546348	658[0n]	2011	Entre Ríos 4
Pyrisitia nise	MACN-Bar-Lep-ct 01542	LEPAR151-11	BOLD:AAZ7347	MF546754	658[0n]	2011	Entre Ríos 4
Pyrisitia nise	MACN-Bar-Lep-ct 02609	LEPPA264-12	BOLD:AAZ7347	MF545757	658[0n]	2011	Formosa 2
Pyrisitia nise	MACN-Bar-Lep-ct 02633	LEPPA286-12	BOLD:AAZ7347	MF546385	658[0n]	2011	Formosa 2
Pyrisitia nise	MACN-Bar-Lep-ct 02699	LEPPA344-12	BOLD:AAZ7347	MF546501	658[0n]	2011	Formosa 2
Pyrisitia nise	MACN-Bar-Lep-ct 02711	LEPPA353-12	BOLD:AAZ7347	MF545720	658[0n]	2011	Formosa 3
Pyrisitia nise	MACN-Bar-Lep-ct 02731	LEPPA367-12	BOLD:AAZ7347	MF547367	658[0n]	2011	Formosa 3
Pyrisitia nise	MACN-Bar-Lep-ct 02737	LEPPA371-12	BOLD:AAZ7347	MF546923	658[0n]	2011	Formosa 3
Pyrisitia nise	MACN-Bar-Lep-ct 03573	LEPPA704-13	BOLD:AAZ7347	MF545949	658[0n]	2011	Corrientes 1
Pyrisitia nise	MACN-Bar-Lep-ct 03591	LEPPA716-13	BOLD:AAZ7347	MF546833	658[0n]	2011	Corrientes 1
Pyrisitia nise	MACN-Bar-Lep-ct 03679	LEPPA768-13	BOLD:AAZ7347	MF546102	658[0n]	2011	Corrientes 1
Pyrisitia nise	MACN-Bar-Lep-ct 06077	LEPAR1010-14	BOLD:AAZ7347	MF546904	658[0n]	2013	Formosa 5
Pyrrhogyra neaerea	MACN-Bar-Lep-ct 01179	LEPAR803-11	BOLD:ACF4031	MF547319	658[0n]	2010	Misiones 2
Pyrrhogyra neaerea	MACN-Bar-Lep-ct 02279	LEPIG366-11	BOLD:AAI0521	MF545825	658[0n]	2011	Misiones 3
Pyrrhogyra neaerea	MACN-Bar-Lep-ct 02494	LEPIG528-11	BOLD:ACF4031	MF546681	658[0n]	2011	Misiones 3
Pyrrhogyra neaerea	MACN-Bar-Lep-ct 02522	LEPIG544-11	BOLD:AAI0521	MF545866	640[0n]	2011	Misiones 3
Pyrrhogyra neaerea	MACN-Bar-Lep-ct 02594	LEPPA249-12	BOLD:AAI0521	MF546109	658[0n]	2011	Formosa 2
Pyrrhogyra neaerea	MACN-Bar-Lep-ct 03905	LEPPA537-13	BOLD:ACF4031	MF546372	658[0n]	2012	Formosa 4
Pyrrhogyra neaerea	MACN-Bar-Lep-ct 06029	LEPAR962-14	BOLD:AAI0521	MF546861	658[0n]	2013	Formosa 5
Pyrrhogyra neaerea	MACN-Bar-Lep-ct 06053	LEPAR986-14	BOLD:ACF4031	MF547210	658[0n]	2013	Formosa 5
Pyrrhopygopsis socrates	MACN-Bar-Lep-ct 01049	LEPAR742-11	BOLD:AAY9909	MF546131	658[0n]	2010	Misiones 2
Pyrrhopygopsis socrates	MACN-Bar-Lep-ct 02130	LEPIG227-11	BOLD:AAY9909	MF546581	658[0n]	2011	Misiones 2
Quadrus cerialis	MACN-Bar-Lep-ct 01889	LEPIG017-11	BOLD:AAA7192	MF546625	658[0n]	2011	Misiones 2

Quinta cannae	MACN-Bar-Lep-ct 02661	LEPPA314-12	BOLD:AAA8156	MF545422	658[0n]	2011	Formosa 2
Quinta cannae	MACN-Bar-Lep-ct 02793	LEPPA405-12	BOLD:AAA8156	MF545835	658[0n]	2011	Formosa 2
Quinta cannae	MACN-Bar-Lep-ct 03959	LEPPA581-13	BOLD:AAA8156	MF547092	658[0n]	2012	Formosa 4
Quinta cannae	MACN-Bar-Lep-ct 06078	LEPAR1011-14	BOLD:AAA8156	MF546539	658[0n]	2013	Formosa 5
Quinta cannae	MACN-Bar-Lep-ct 06094	LEPAR1027-14	BOLD:AAA8156	MF546488	658[2n]	2013	Formosa 5
Rekoa marius	MACN-Bar-Lep-ct 02584	LEPPA239-12	BOLD:AAB9273	MF546127	658[0n]	2011	Formosa 2
Rekoa meton	MACN-Bar-Lep-ct 02754	LEPPA383-12	BOLD:AAF8443	MF546775	658[0n]	2011	Formosa 2
Rekoa palegon	MACN-Bar-Lep-ct 01943	LEPIG065-11	BOLD:ACF3389	MF546711	658[0n]	2011	Misiones 2
Rekoa palegon	MACN-Bar-Lep-ct 01992	LEPIG109-11	BOLD:ACF3389	MF547273	658[0n]	2011	Misiones 2
Rekoa palegon	MACN-Bar-Lep-ct 01995	LEPIG112-11	BOLD:ACF3389	MF547324	658[0n]	2011	Misiones 2
Rekoa palegon	MACN-Bar-Lep-ct 02590	LEPPA245-12	BOLD:ACF3389	MF546848	658[0n]	2011	Formosa 2
Rekoa palegon	MACN-Bar-Lep-ct 02601	LEPPA256-12	BOLD:ACF3389	MF546563	658[0n]	2011	Formosa 2
Rekoa palegon	MACN-Bar-Lep-ct 02604	LEPPA259-12	BOLD:ACF3389	MF547279	658[0n]	2011	Formosa 2
Rekoa palegon	MACN-Bar-Lep-ct 03704	LEPPA789-13	BOLD:ACF3389	MF546892	658[0n]	2011	Corrientes 1
Rekoa palegon	MACN-Bar-Lep-ct 06082	LEPAR1015-14	BOLD:ACF3389	MF546784	658[2n]	2013	Formosa 5
Rekoa palegon	MACN-Bar-Lep-ct 06088	LEPAR1021-14	BOLD:ACF3389	MF546472	658[2n]	2013	Formosa 5
Rekoa palegon	MACN-Bar-Lep-ct 06107	LEPAR1040-14	BOLD:ACF3389	MF545747	658[2n]	2013	Formosa 5
Remella remus	MACN-Bar-Lep-ct 02034	LEPIG144-11	BOLD:AAZ7379	MF546041	658[0n]	2011	Misiones 2
Rhabdodryas trite	MACN-Bar-Lep-ct 00289	LEPAR352-11	BOLD:AAD6347	MF546611	658[0n]	2010	Misiones 2
Rhabdodryas trite	MACN-Bar-Lep-ct 02479	LEPIG518-11	BOLD:AAD6347	MF545391	650[0n]	2011	Misiones 3
Rhabdodryas trite	MACN-Bar-Lep-ct 02796	LEPPA407-12	BOLD:AAD6347	MF545595	658[0n]	2011	Formosa 2
Rhabdodryas trite	MACN-Bar-Lep-ct 06036	LEPAR969-14	BOLD:AAD6347	MF546207	658[0n]	2013	Formosa 5
Riodina lycisca	MACN-Bar-Lep-ct 00366	LEPAR401-11	BOLD:AAZ0995	MF545763	658[0n]	2010	Misiones 2
Riodina lycisca	MACN-Bar-Lep-ct 00843	LEPAR616-11	BOLD:AAZ0995	MF546890	658[0n]	2010	Misiones 2
Riodina lycisca	MACN-Bar-Lep-ct 00995	LEPAR697-11	BOLD:AAZ0995	MF547218	658[0n]	2010	Misiones 2
Riodina lycisca	MACN-Bar-Lep-ct 01017	LEPAR714-11	BOLD:AAZ0995	MF546683	658[0n]	2010	Misiones 2
Riodina lycisca	MACN-Bar-Lep-ct 01193	LEPAR815-11	BOLD:AAZ0995	MF545755	658[0n]	2010	Misiones 2
Riodina lysippoides	MACN-Bar-Lep-ct 01747	LEPAR233-11			0	2011	Entre Ríos 6
Riodina lysippoides	MACN-Bar-Lep-ct 01753	LEPAR235-11	BOLD:AAZ7726	MF546936	658[0n]	2011	Entre Ríos 6
Riodina lysippoides	MACN-Bar-Lep-ct 01759	LEPAR239-11	BOLD:AAZ7726	MF546484	658[0n]	2011	Entre Ríos 6
Riodina lysippoides	MACN-Bar-Lep-ct 01794	LEPAR246-11	BOLD:AAZ7726	MF547201	658[0n]	2011	Entre Ríos 6
Riodina lysippoides	MACN-Bar-Lep-ct 01838	LEPAR263-11	BOLD:ACE5516	MF547182	658[0n]	2011	Entre Ríos 3
Riodina lysippoides	MACN-Bar-Lep-ct 01865	LEPAR281-11	BOLD:ACE5516	MF546493	658[0n]	2011	Entre Ríos 3

Riodina lysippoides	MACN-Bar-Lep-ct 03122	LEPPA045-11			0	2011	Buenos Aires 21
Riodina lysippoides	MACN-Bar-Lep-ct 03571	LEPPA703-13	BOLD:ACE5516	MF547342	638[0n]	2011	Corrientes 1
Riodina lysippoides	MACN-Bar-Lep-ct 03594	LEPPA719-13	BOLD:ACE5516	MF546289	658[0n]	2011	Corrientes 1
Riodina lysippoides	MACN-Bar-Lep-ct 03734	LEPPA809-13	BOLD:ACE5516	MF547031	658[0n]	2011	Corrientes 1
Sarmientoia almeidae	MACN-Bar-Lep-ct 03900	LEPPA534-13	BOLD:ABW2644	MF545661	658[0n]	2012	Formosa 4
Saturnus reticulata	MACN-Bar-Lep-ct 01034	LEPAR730-11	BOLD:AAZ1494	MF546512	658[0n]	2010	Misiones 2
Selenophanes cassiope	MACN-Bar-Lep-ct 00626	LEPAR547-11	BOLD:AAZ4948	MF546351	658[0n]	2010	Misiones 2
Selenophanes cassiope	MACN-Bar-Lep-ct 01879	LEPIG007-11	BOLD:AAZ4948	MF546626	658[0n]	2011	Misiones 2
Siproeta epaphus	MACN-Bar-Lep-ct 00230	LEPAR319-11	BOLD:ACF2703	MF546391	658[0n]	2010	Misiones 2
Siproeta epaphus	MACN-Bar-Lep-ct 00557	LEPAR485-11	BOLD:ACF2703	MF547259	658[0n]	2010	Misiones 2
Siproeta epaphus	MACN-Bar-Lep-ct 02142	LEPIG239-11	BOLD:ACF2703	MF546274	658[0n]	2011	Misiones 3
Siproeta epaphus	MACN-Bar-Lep-ct 02464	LEPIG508-11	BOLD:ACF2703	MF546659	658[0n]	2011	Misiones 3
Siproeta epaphus	MACN-Bar-Lep-ct 02569	LEPIG584-11	BOLD:ACF2703	MF545790	658[0n]	2011	Misiones 3
Siproeta stelenes	MACN-Bar-Lep-ct 00317	LEPAR369-11	BOLD:AAB9470	MF545718	658[0n]	2010	Misiones 2
Siproeta stelenes	MACN-Bar-Lep-ct 01910	LEPIG037-11	BOLD:AAB9470	MF545873	658[0n]	2011	Misiones 2
Siproeta stelenes	MACN-Bar-Lep-ct 02149	LEPIG245-11	BOLD:AAB9470	MF545856	658[0n]	2011	Misiones 3
Siproeta stelenes	MACN-Bar-Lep-ct 02174	LEPIG268-11	BOLD:AAB9470	MF547214	658[0n]	2011	Misiones 3
Siproeta stelenes	MACN-Bar-Lep-ct 02192	LEPIG282-11	BOLD:AAB9470	MF545749	658[0n]	2011	Misiones 3
Siproeta stelenes	MACN-Bar-Lep-ct 02249	LEPIG337-11	BOLD:AAB9470	MF547061	658[0n]	2011	Misiones 3
Siproeta stelenes	MACN-Bar-Lep-ct 02403	LEPIG468-11	BOLD:AAB9470	MF545401	658[0n]	2011	Misiones 3
Siproeta stelenes	MACN-Bar-Lep-ct 02407	LEPIG472-11	BOLD:AAB9470	MF545984	658[0n]	2011	Misiones 3
Smyrna blomfildia	MACN-Bar-Lep-ct 01313	LEPAR895-11	BOLD:AAC1408	MF545697	658[0n]	2010	Misiones 2
Smyrna blomfildia	MACN-Bar-Lep-ct 02161	LEPIG256-11	BOLD:AAC1408	MF545424	640[0n]	2011	Misiones 3
Smyrna blomfildia	MACN-Bar-Lep-ct 02287	LEPIG373-11	BOLD:AAC1408	MF545647	658[0n]	2011	Misiones 3
Smyrna blomfildia	MACN-Bar-Lep-ct 02301	LEPIG386-11	BOLD:AAC1408	MF545897	658[0n]	2011	Misiones 3
Smyrna blomfildia	MACN-Bar-Lep-ct 02393	LEPIG458-11	BOLD:AAC1408	MF546823	658[0n]	2011	Misiones 3
Smyrna blomfildia	MACN-Bar-Lep-ct 02482	LEPIG520-11	BOLD:AAC1408	MF546232	658[0n]	2011	Misiones 3
Smyrna blomfildia	MACN-Bar-Lep-ct 02523	LEPIG545-11	BOLD:AAC1408	MF546632	658[0n]	2011	Misiones 3
Smyrna blomfildia	MACN-Bar-Lep-ct 02540	LEPIG559-11	BOLD:AAC1408	MF545990	644[0n]	2011	Misiones 3
Sodalia coler	MACN-Bar-Lep-ct 00189	LEPAR307-11	BOLD:AAZ2392	MF546603	658[0n]	2010	Misiones 2
Sodalia coler	MACN-Bar-Lep-ct 00428	LEPAR440-11	BOLD:AAZ2392	MF545853	658[0n]	2010	Misiones 2
Sodalia coler	MACN-Bar-Lep-ct 01228	LEPAR843-11	BOLD:AAZ2392	MF545813	658[0n]	2010	Misiones 2
Sodalia coler	MACN-Bar-Lep-ct 01229	LEPAR844-11	BOLD:AAZ2392	MF547026	658[0n]	2010	Misiones 2

Sodalia coler	MACN-Bar-Lep-ct 01339	LEPAR920-11	BOLD:AAZ2392	MF545880	658[0n]	2010	Misiones 2
Sodalia coler	MACN-Bar-Lep-ct 02259	LEPIG347-11	BOLD:AAZ2392	MF546040	658[0n]	2011	Misiones 3
Sodalia coler	MACN-Bar-Lep-ct 02260	LEPIG348-11	BOLD:AAZ2392	MF546221	658[0n]	2011	Misiones 3
Sodalia coler	MACN-Bar-Lep-ct 02263	LEPIG351-11	BOLD:AAZ2392	MF546427	658[0n]	2011	Misiones 3
Sodalia coler	MACN-Bar-Lep-ct 02266	LEPIG354-11	BOLD:AAZ2392	MF546996	658[0n]	2011	Misiones 3
Sodalia coler	MACN-Bar-Lep-ct 02269	LEPIG357-11	BOLD:AAZ2392	MF545403	658[0n]	2011	Misiones 3
Sodalia coler	MACN-Bar-Lep-ct 02543	LEPIG561-11	BOLD:AAZ2392	MF546650	658[0n]	2011	Misiones 3
Splendeuptychia hygina	MACN-Bar-Lep-ct 00861	LEPAR630-11	BOLD:AAZ2707	MF546872	658[0n]	2010	Misiones 2
Splendeuptychia hygina	MACN-Bar-Lep-ct 01211	LEPAR829-11	BOLD:AAZ2707	MF547193	658[0n]	2010	Misiones 2
Splendeuptychia hygina	MACN-Bar-Lep-ct 02199	LEPIG289-11	BOLD:AAZ2707	MF546457	658[0n]	2011	Misiones 3
Splendeuptychia hygina	MACN-Bar-Lep-ct 02235	LEPIG324-11	BOLD:AAZ2707	MF547298	658[0n]	2011	Misiones 3
Splendeuptychia hygina	MACN-Bar-Lep-ct 02318	LEPIG401-11	BOLD:AAZ2707	MF547312	658[0n]	2011	Misiones 3
Splendeuptychia libitina	MACN-Bar-Lep-ct 00266	LEPAR339-11	BOLD:AAZ2706	MF545723	658[0n]	2010	Misiones 2
Splendeuptychia libitina	MACN-Bar-Lep-ct 01129	LEPAR764-11	BOLD:AAZ2706	MF547346	658[0n]	2010	Misiones 2
Splendeuptychia libitina	MACN-Bar-Lep-ct 01942	LEPIG064-11	BOLD:AAZ2706	MF547080	658[0n]	2011	Misiones 2
Staphylus chlorocephala	MACN-Bar-Lep-ct 02547	LEPIG565-11	BOLD:AAZ7944	MF546168	658[0n]	2011	Misiones 3
Staphylus incisus	MACN-Bar-Lep-ct 00874	LEPAR641-11	BOLD:AAZ4940	MF546641	658[0n]	2010	Misiones 2
Staphylus incisus	MACN-Bar-Lep-ct 02336	LEPIG417-11	BOLD:AAZ4940	MF546706	658[0n]	2011	Misiones 3
Staphylus melangon	MACN-Bar-Lep-ct 00184	LEPAR302-11	BOLD:AAZ2332	MF546371	658[0n]	2010	Misiones 2
Staphylus melangon	MACN-Bar-Lep-ct 00429	LEPAR441-11	BOLD:AAZ2332	MF547387	658[0n]	2010	Misiones 2
Staphylus melangon	MACN-Bar-Lep-ct 00430	LEPAR442-11	BOLD:AAZ2332	MF545821	658[0n]	2010	Misiones 2
Staphylus melangon	MACN-Bar-Lep-ct 00571	LEPAR499-11	BOLD:AAZ2332	MF546357	658[0n]	2010	Misiones 2
Staphylus melangon	MACN-Bar-Lep-ct 00875	LEPAR642-11	BOLD:AAZ2332	MF546439	658[0n]	2010	Misiones 2
Staphylus melangon	MACN-Bar-Lep-ct 01220	LEPAR835-11	BOLD:AAZ2332	MF546990	658[0n]	2010	Misiones 2
Staphylus melangon	MACN-Bar-Lep-ct 01226	LEPAR841-11	BOLD:AAZ2332	MF546827	658[0n]	2010	Misiones 2
Staphylus melangon	MACN-Bar-Lep-ct 02054	LEPIG162-11	BOLD:AAZ2332	MF545508	658[0n]	2011	Misiones 2
Staphylus melangon	MACN-Bar-Lep-ct 02226	LEPIG315-11	BOLD:AAZ2332	MF546451	658[0n]	2011	Misiones 3
Staphylus melangon	MACN-Bar-Lep-ct 02229	LEPIG318-11	BOLD:AAZ2332	MF546235	658[0n]	2011	Misiones 3
Staphylus melangon	MACN-Bar-Lep-ct 02324	LEPIG405-11	BOLD:AAZ2332	MF547097	658[0n]	2011	Misiones 3
Staphylus melangon	MACN-Bar-Lep-ct 02362	LEPIG433-11	BOLD:AAZ2332	MF545480	658[0n]	2011	Misiones 3
Staphylus melangon	MACN-Bar-Lep-ct 02457	LEPIG506-11	BOLD:AAZ2332	MF546723	658[0n]	2011	Misiones 3
Staphylus melangon	MACN-Bar-Lep-ct 03792	LEPPA841-13	BOLD:AAZ2332	MF546482	658[0n]	2011	Corrientes 1
Staphylus musculus	MACN-Bar-Lep-ct 01817	LEPAR255-11	BOLD:AAZ9590	MF546431	658[0n]	2011	Entre Ríos 3

Staphylus musculus	MACN-Bar-Lep-ct 06058	LEPAR991-14			0	2013	Formosa 5
Staphylus musculus	MACN-Bar-Lep-ct 06070	LEPAR1003-14			0	2013	Formosa 5
Staphylus tucumanus	MACN-Bar-Lep-ct 02617	LEPPA271-12	BOLD:ACA8981	MF547069	658[0n]	2011	Formosa 2
Staphylus tucumanus	MACN-Bar-Lep-ct 02806	LEPPA411-12	BOLD:ACA8981	MF545503	658[0n]	2011	Formosa 2
Staphylus vulgata	MACN-Bar-Lep-ct 01055	LEPAR748-11	BOLD:AAY9951	MF546957	658[0n]	2010	Misiones 2
Staphylus vulgata	MACN-Bar-Lep-ct 02725	LEPPA363-12	BOLD:AAY9951	MF545712	658[0n]	2011	Formosa 3
Stegosatyrus periphas	MACN-Bar-Lep-ct 00075	LEPAR055-11	BOLD:AAY8246	MF546378	658[0n]	2009	Córdoba 1
Stegosatyrus periphas	MACN-Bar-Lep-ct 01651	LEPAR195-11	BOLD:AAY8246	MF545608	658[0n]	2011	Entre Ríos 4
Stegosatyrus periphas	MACN-Bar-Lep-ct 01657	LEPAR198-11	BOLD:AAY8246	MF545529	658[0n]	2011	Entre Ríos 4
Stegosatyrus periphas	MACN-Bar-Lep-ct 01668	LEPAR204-11	BOLD:AAY8246	MF546729	658[0n]	2011	Entre Ríos 4
Stegosatyrus periphas	MACN-Bar-Lep-ct 01671	LEPAR206-11	BOLD:AAY8246	MF545616	658[0n]	2011	Entre Ríos 4
Stegosatyrus periphas	MACN-Bar-Lep-ct 01673	LEPAR207-11	BOLD:AAY8246	MF546970	658[0n]	2011	Entre Ríos 4
Stegosatyrus periphas	MACN-Bar-Lep-ct 03604	LEPPA727-13	BOLD:AAY8246	MF545740	658[0n]	2011	Corrientes 1
Stegosatyrus periphas	MACN-Bar-Lep-ct 03617	LEPPA735-13	BOLD:AAY8246	MF545487	658[0n]	2011	Corrientes 1
Stegosatyrus periphas	MACN-Bar-Lep-ct 03733	LEPPA808-13	BOLD:AAY8246	MF546248	658[0n]	2011	Corrientes 1
Stegosatyrus periphas	MACN-Bar-Lep-ct 06092	LEPAR1025-14	BOLD:AAY8246	MF546331	658[0n]	2013	Formosa 5
Stegosatyrus periphas	MACN-Bar-Lep-ct 07133	LEPAR1211-15	BOLD:AAY8246	MF546209	658[0n]	2015	Corrientes 5
Strephonota sphinx	MACN-Bar-Lep-ct 01230	LEPAR845-11	BOLD:AAZ2647	MF546477	658[0n]	2010	Misiones 2
Strephonota tephraeus	MACN-Bar-Lep-ct 01231	LEPAR846-11	BOLD:AAZ1716	MF546285	650[0n]	2010	Misiones 2
Strephonota tephraeus	MACN-Bar-Lep-ct 01334	LEPAR915-11	BOLD:AAZ1716	MF546995	658[0n]	2010	Misiones 2
Strephonota tephraeus	MACN-Bar-Lep-ct 01384	LEPAR944-11	BOLD:AAZ1716	MF545985	658[0n]	2010	Misiones 2
Strymon astiocha	MACN-Bar-Lep-ct 02371	LEPIG441-11	BOLD:AAZ9001	MF546237	658[0n]	2011	Misiones 3
Strymon astiocha	MACN-Bar-Lep-ct 02568	LEPIG583-11	BOLD:AAZ9001	MF546776	658[0n]	2011	Misiones 3
Strymon bazochii	MACN-Bar-Lep-ct 06093	LEPAR1026-14	BOLD:ACK2869	MF547366	658[0n]	2013	Formosa 5
Strymon bubastus	MACN-Bar-Lep-ct 02135	LEPIG232-11	BOLD:AAZ7832	MF545733	649[0n]	2011	Misiones 2
Strymon cestri	MACN-Bar-Lep-ct 02640	LEPPA293-12	BOLD:ACA9089	MF545762	658[0n]	2011	Formosa 2
Strymon eurytulus	MACN-Bar-Lep-ct 01449	LEPAR099-11	BOLD:AAJ4515	MF546108	658[0n]	2011	Entre Ríos 3
Strymon eurytulus	MACN-Bar-Lep-ct 01612	LEPAR175-11	BOLD:AAJ4515	MF546074	658[0n]	2011	Entre Ríos 5
Strymon eurytulus	MACN-Bar-Lep-ct 03068	LEPPA181-11			0	2011	Buenos Aires 6
Strymon eurytulus	MACN-Bar-Lep-ct 03683	LEPPA772-13	BOLD:AAJ4515	MF546022	658[0n]	2011	Corrientes 1
Strymon eurytulus	MACN-Bar-Lep-ct 03763	LEPPA827-13	BOLD:AAJ4515	MF546163	658[0n]	2011	Corrientes 1
Strymon eurytulus	MACN-Bar-Lep-ct 03849	LEPPA875-13	BOLD:AAJ4515	MF546691	658[0n]	2011	Corrientes 1
Strymon eurytulus	MACN-Bar-Lep-ct 03879	LEPPA521-13	BOLD:AAJ4515	MF547404	658[0n]	2012	Formosa 4

Strymon eurytulus	MACN-Bar-Lep-ct 03886	LEPPA525-13	BOLD:AAJ4515	MF545626	620[0n]	2012	Formosa 4
Strymon eurytulus	MACN-Bar-Lep-ct 03896	LEPPA530-13	BOLD:AAJ4515	MF545773	631[0n]	2012	Formosa 4
Strymon eurytulus	MACN-Bar-Lep-ct 06466	LEPPA1054-14	BOLD:AAJ4515	MF547045	658[0n]	2013	Córdoba 4
Strymon lucena	MACN-Bar-Lep-ct 03071	LEPPA184-11	BOLD:ADB5407	MF546517	561[1n]	2011	Buenos Aires 16
Strymon megarus	MACN-Bar-Lep-ct 02614	LEPPA268-12	BOLD:ACA9111	MF546210	658[0n]	2011	Formosa 2
Strymon megarus	MACN-Bar-Lep-ct 02832	LEPPA420-12	BOLD:ACA9111	MF547020	658[0n]	2011	Formosa 2
Strymon mulucha	MACN-Bar-Lep-ct 06073	LEPAR1006-14	BOLD:ACP0684	MF546495	658[2n]	2013	Formosa 5
Strymon mulucha	MACN-Bar-Lep-ct 06076	LEPAR1009-14	BOLD:ACP0684	MF547074	658[0n]	2013	Formosa 5
Strymon rufofusca	MACN-Bar-Lep-ct 01544	LEPAR152-11	BOLD:AAZ7959	MF545676	658[0n]	2011	Entre Ríos 3
Strymon rufofusca	MACN-Bar-Lep-ct 01757	LEPAR237-11	BOLD:AAZ7959	MF546542	658[0n]	2011	Entre Ríos 6
Strymon rufofusca	MACN-Bar-Lep-ct 06085	LEPAR1018-14	BOLD:AAZ7959	MF545591	658[0n]	2013	Formosa 5
Synale elana	MACN-Bar-Lep-ct 06495	LEPPA1083-14			0	2010	Corrientes 3
Synale elana	MACN-Bar-Lep-ct 06501	LEPPA1089-14			0	2010	Corrientes 3
Synale elana	MACN-Bar-Lep-ct 06505	LEPPA1093-14			0	2010	Corrientes 3
Synale elana	MACN-Bar-Lep-ct 06507	LEPPA1095-14			0	2010	Corrientes 3
Synapte malitiosa	MACN-Bar-Lep-ct 01323	LEPAR904-11	BOLD:AAD7529	MF546965	658[0n]	2010	Misiones 2
Synapte silius	MACN-Bar-Lep-ct 01130	LEPAR765-11	BOLD:AAA4443	MF545407	658[0n]	2010	Misiones 2
Synapte silius	MACN-Bar-Lep-ct 01131	LEPAR766-11	BOLD:AAA4443	MF546377	658[0n]	2010	Misiones 2
Synapte silius	MACN-Bar-Lep-ct 03895	LEPPA529-13	BOLD:AAA4443	MF547349	658[0n]	2012	Formosa 4
Synargis calyce	MACN-Bar-Lep-ct 02154	LEPIG250-11	BOLD:AAZ8169	MF546454	658[0n]	2011	Misiones 3
Synargis calyce	MACN-Bar-Lep-ct 02193	LEPIG283-11	BOLD:AAZ8169	MF547399	658[0n]	2011	Misiones 3
Synargis calyce	MACN-Bar-Lep-ct 02201	LEPIG291-11	BOLD:AAZ8169	MF547392	658[0n]	2011	Misiones 3
Synargis ochrophlegma	MACN-Bar-Lep-ct 01499	LEPAR129-11	BOLD:AAZ9009	MF546951	658[0n]	2011	Entre Ríos 3
Synargis ochrophlegma	MACN-Bar-Lep-ct 01501	LEPAR131-11	BOLD:AAZ9009	MF545931	658[0n]	2011	Entre Ríos 3
Tatochila autodice	MACN-Bar-Lep-ct 00088	LEPAR066-11			0	2004	Buenos Aires 10
Tatochila autodice	MACN-Bar-Lep-ct 00089	LEPAR067-11			0	2005	Entre Ríos 2
Tatochila autodice	MACN-Bar-Lep-ct 00090	LEPAR068-11	BOLD:AAK0357	MF547377	658[0n]	2010	Córdoba 1
Tatochila autodice	MACN-Bar-Lep-ct 00091	LEPAR069-11	BOLD:AAK0357	MF546462	658[0n]	2010	Córdoba 1
Tatochila autodice	MACN-Bar-Lep-ct 00092	LEPAR070-11	BOLD:AAK0357	MF546337	658[0n]	2010	Córdoba 1
Tatochila autodice	MACN-Bar-Lep-ct 03065	LEPPA178-11	BOLD:AAK0357	MF545868	658[0n]	2011	Buenos Aires 16
Tatochila autodice	MACN-Bar-Lep-ct 03073	LEPPA186-11	BOLD:AAK0357	MF546692	658[0n]	2011	Buenos Aires 16
Tatochila autodice	MACN-Bar-Lep-ct 06472	LEPPA1060-14			0	2013	Córdoba 4
Tatochila mercedis	MACN-Bar-Lep-ct 06470	LEPPA1058-14			0	2013	Córdoba 4

Tatochila mercedis	MACN-Bar-Lep-ct 06475	LEPPA1063-14			0	2013	Córdoba 4
Taygetis kerea	MACN-Bar-Lep-ct 02241	LEPIG329-11	BOLD:AAA8572	MF547113	658[0n]	2011	Misiones 3
Taygetis rufomarginata	MACN-Bar-Lep-ct 00883	LEPAR650-11	BOLD:ACF0552	MF545922	658[0n]	2010	Misiones 2
Taygetis rufomarginata	MACN-Bar-Lep-ct 01009	LEPAR709-11	BOLD:ACF0552	MF545726	658[0n]	2010	Misiones 2
Taygetis rufomarginata	MACN-Bar-Lep-ct 01022	LEPAR719-11	BOLD:ACF0552	MF546759	658[0n]	2010	Misiones 2
Taygetis rufomarginata	MACN-Bar-Lep-ct 01283	LEPAR870-11	BOLD:ACF0552	MF546490	658[0n]	2010	Misiones 2
Taygetis rufomarginata	MACN-Bar-Lep-ct 01347	LEPAR928-11	BOLD:ACF0552	MF546297	658[0n]	2010	Misiones 2
Taygetis rufomarginata	MACN-Bar-Lep-ct 07117	LEPAR1195-15			0	2003	Misiones 7
Taygetis tripunctata	MACN-Bar-Lep-ct 01882	LEPIG010-11	BOLD:AAZ7820	MF546065	658[0n]	2011	Misiones 2
Taygetis tripunctata	MACN-Bar-Lep-ct 02064	LEPIG171-11	BOLD:AAZ7820	MF546585	658[0n]	2011	Misiones 2
Taygetis tripunctata	MACN-Bar-Lep-ct 02122	LEPIG219-11	BOLD:AAZ7820	MF546303	658[0n]	2011	Misiones 2
Taygetis tripunctata	MACN-Bar-Lep-ct 02246	LEPIG334-11	BOLD:AAZ7820	MF545620	658[0n]	2011	Misiones 3
Taygetis tripunctata	MACN-Bar-Lep-ct 02376	LEPIG445-11	BOLD:AAZ7820	MF546516	658[0n]	2011	Misiones 3
Taygetis virgilia	MACN-Bar-Lep-ct 07115	LEPAR1193-15			0	1998	Misiones 12
Taygetis ypthima	MACN-Bar-Lep-ct 01175	LEPAR799-11	BOLD:AAZ2419	MF547131	658[0n]	2010	Misiones 2
Taygetis ypthima	MACN-Bar-Lep-ct 01270	LEPAR857-11	BOLD:AAZ2419	MF545837	658[0n]	2010	Misiones 2
Tegosa claudina	MACN-Bar-Lep-ct 00044	LEPAR028-11	BOLD:AAE9813	MF546762	658[0n]	2004	Buenos Aires 10
Tegosa claudina	MACN-Bar-Lep-ct 00045	LEPAR029-11	BOLD:AAE9813	MF546771	658[0n]	2005	Buenos Aires 14
Tegosa claudina	MACN-Bar-Lep-ct 00131	LEPPA106-11	BOLD:AAE9813	MF545545	658[0n]	2010	Buenos Aires 21
Tegosa claudina	MACN-Bar-Lep-ct 00132	LEPPA107-11	BOLD:AAE9813	MF546869	658[0n]	2010	Buenos Aires 21
Tegosa claudina	MACN-Bar-Lep-ct 00393	LEPAR413-11	BOLD:AAE9813	MF546500	658[0n]	2010	Misiones 2
Tegosa claudina	MACN-Bar-Lep-ct 00394	LEPAR414-11	BOLD:AAE9813	MF547338	658[0n]	2010	Misiones 2
Tegosa claudina	MACN-Bar-Lep-ct 00395	LEPAR415-11	BOLD:AAE9813	MF545745	658[0n]	2010	Misiones 2
Tegosa claudina	MACN-Bar-Lep-ct 00397	LEPAR416-11	BOLD:AAE9813	MF545415	658[0n]	2010	Misiones 2
Tegosa claudina	MACN-Bar-Lep-ct 00407	LEPAR422-11	BOLD:AAE9813	MF546218	658[0n]	2010	Misiones 2
Tegosa claudina	MACN-Bar-Lep-ct 01949	LEPIG071-11	BOLD:AAE9813	MF545940	658[0n]	2011	Misiones 2
Tegosa claudina	MACN-Bar-Lep-ct 02783	LEPPA401-12	BOLD:AAE9813	MF547226	658[0n]	2011	Formosa 2
Tegosa claudina	MACN-Bar-Lep-ct 02841	LEPPA424-12	BOLD:AAE9813	MF547166	658[0n]	2011	Formosa 2
Tegosa claudina	MACN-Bar-Lep-ct 03115	LEPPA038-11			0	2010	Buenos Aires 21
Tegosa claudina	MACN-Bar-Lep-ct 03153	LEPPA069-11	BOLD:AAE9813	MF545800	658[0n]	2010	Buenos Aires 21
Tegosa claudina	MACN-Bar-Lep-ct 03597	LEPPA722-13	BOLD:AAE9813	MF545961	658[0n]	2011	Corrientes 1
Tegosa claudina	MACN-Bar-Lep-ct 03619	LEPPA736-13	BOLD:AAE9813	MF545559	620[0n]	2011	Corrientes 1
Tegosa claudina	MACN-Bar-Lep-ct 03645	LEPPA747-13	BOLD:AAE9813	MF546924	658[0n]	2011	Corrientes 1

Tegosa claudina	MACN-Bar-Lep-ct 03719	LEPPA800-13	BOLD:AAE9813	MF546200	658[0n]	2011	Corrientes 1
Tegosa claudina	MACN-Bar-Lep-ct 03775	LEPPA832-13	BOLD:AAE9813	MF545881	658[0n]	2011	Corrientes 1
Tegosa orobia	MACN-Bar-Lep-ct 07093	LEPAR1171-15			0	2005	Entre Ríos 10
Tegosa orobia	MACN-Bar-Lep-ct 07095	LEPAR1173-15	BOLD:ADB9157	MF546839	647[0n]	2013	Entre Ríos 7
Telemiades amphion	MACN-Bar-Lep-ct 01318	LEPAR900-11	BOLD:AAZ4956	MF546964	658[0n]	2010	Misiones 2
Telemiades laogonus	MACN-Bar-Lep-ct 02076	LEPIG180-11	BOLD:AAZ9480	MF545622	658[0n]	2011	Misiones 2
Temenis laothoe	MACN-Bar-Lep-ct 00573	LEPAR501-11	BOLD:ACF1946	MF547098	658[0n]	2010	Misiones 2
Temenis laothoe	MACN-Bar-Lep-ct 00794	LEPAR575-11	BOLD:ACF1946	MF546607	658[0n]	2010	Misiones 2
Temenis laothoe	MACN-Bar-Lep-ct 00797	LEPAR578-11	BOLD:ACF1946	MF546093	658[0n]	2010	Misiones 2
Temenis laothoe	MACN-Bar-Lep-ct 00811	LEPAR588-11	BOLD:ACF1946	MF546947	658[0n]	2010	Misiones 2
Temenis laothoe	MACN-Bar-Lep-ct 01181	LEPAR804-11	BOLD:ACF1946	MF546937	658[0n]	2010	Misiones 2
Temenis laothoe	MACN-Bar-Lep-ct 02317	LEPIG400-11			0	2011	Misiones 3
Temenis laothoe	MACN-Bar-Lep-ct 02348	LEPIG424-11	BOLD:ACF1946	MF547142	658[0n]	2011	Misiones 3
Temenis laothoe	MACN-Bar-Lep-ct 02409	LEPIG474-11	BOLD:ACF1946	MF545618	658[0n]	2011	Misiones 3
Temenis laothoe	MACN-Bar-Lep-ct 06048	LEPAR981-14	BOLD:AAC4851	MF547397	658[2n]	2013	Formosa 5
Theagenes dichrous	MACN-Bar-Lep-ct 03152	LEPPA068-11	BOLD:ABV4646	MF547190	658[0n]	2011	Buenos Aires 21
Thespieus aspernatus	MACN-Bar-Lep-ct 00431	LEPAR443-11	BOLD:AAZ1295	MF546554	658[0n]	2010	Misiones 2
Thespieus aspernatus	MACN-Bar-Lep-ct 00812	LEPAR589-11	BOLD:AAZ1295	MF545980	658[0n]	2010	Misiones 2
Thespieus aspernatus	MACN-Bar-Lep-ct 01052	LEPAR745-11	BOLD:AAZ1295	MF547227	658[0n]	2010	Misiones 2
Thespieus aspernatus	MACN-Bar-Lep-ct 01135	LEPAR769-11	BOLD:AAZ1295	MF546926	658[0n]	2010	Misiones 2
Thespieus aspernatus	MACN-Bar-Lep-ct 02512	LEPIG538-11	BOLD:AAZ1295	MF547079	658[0n]	2011	Misiones 3
Thespieus dalman	MACN-Bar-Lep-ct 01051	LEPAR744-11	BOLD:AAZ0895	MF546591	658[0n]	2010	Misiones 2
Thespieus dalman	MACN-Bar-Lep-ct 01385	LEPAR945-11	BOLD:AAZ0895	MF546534	658[0n]	2010	Misiones 2
Thespieus dalman	MACN-Bar-Lep-ct 02328	LEPIG409-11	BOLD:AAZ0895	MF546092	658[0n]	2011	Misiones 3
Thespieus ethemides	MACN-Bar-Lep-ct 00434	LEPAR446-11	BOLD:AAZ5394	MF546789	658[0n]	2010	Misiones 2
Thespieus ethemides	MACN-Bar-Lep-ct 00435	LEPAR447-11	BOLD:AAZ5394	MF546141	658[0n]	2010	Misiones 2
Thracides cleanthes	MACN-Bar-Lep-ct 01345	LEPAR926-11	BOLD:AAZ4760	MF547211	658[0n]	2010	Misiones 2
Thyridia psidii	MACN-Bar-Lep-ct 01911	LEPIG038-11	BOLD:AAI0473	MF546016	658[0n]	2011	Misiones 2
Thyridia psidii	MACN-Bar-Lep-ct 02019	LEPIG132-11	BOLD:AAI0473	MF547165	658[0n]	2011	Misiones 2
Thyridia psidii	MACN-Bar-Lep-ct 07080	LEPAR1158-15			0	1996	Misiones 5
Tisias lesueur	MACN-Bar-Lep-ct 00165	LEPAR292-11	BOLD:AAZ2423	MF546929	658[0n]	2010	Misiones 2
Tisias lesueur	MACN-Bar-Lep-ct 01224	LEPAR839-11	BOLD:AAZ2423	MF546716	658[0n]	2010	Misiones 2
Tisias lesueur	MACN-Bar-Lep-ct 01225	LEPAR840-11	BOLD:AAZ2423	MF547032	658[0n]	2010	Misiones 2

Tithorea harmonia	MACN-Bar-Lep-ct 00994	LEPAR696-11	BOLD:ACF0041	MF545776	658[0n]	2010	Misiones 2
Tithorea harmonia	MACN-Bar-Lep-ct 02264	LEPIG352-11	BOLD:ACF0041	MF546382	658[0n]	2011	Misiones 3
Tithorea harmonia	MACN-Bar-Lep-ct 02339	LEPIG419-11	BOLD:ACF0041	MF547372	658[0n]	2011	Misiones 3
Tithorea harmonia	MACN-Bar-Lep-ct 02347	LEPIG423-11	BOLD:ACF0041	MF546655	658[0n]	2011	Misiones 3
Tithorea harmonia	MACN-Bar-Lep-ct 02351	LEPIG426-11	BOLD:ACF0041	MF546338	658[0n]	2011	Misiones 3
Tithorea harmonia	MACN-Bar-Lep-ct 02373	LEPIG443-11	BOLD:ACF0041	MF546953	658[0n]	2011	Misiones 3
Tithorea harmonia	MACN-Bar-Lep-ct 02518	LEPIG540-11	BOLD:ACF0041	MF546934	658[0n]	2011	Misiones 3
Trina geometrina	MACN-Bar-Lep-ct 00570	LEPAR498-11	BOLD:AAZ0920	MF545392	658[0n]	2010	Misiones 2
Trina geometrina	MACN-Bar-Lep-ct 00616	LEPAR538-11	BOLD:ABY8023	MF545925	658[0n]	2010	Misiones 2
Trina geometrina	MACN-Bar-Lep-ct 00869	LEPAR637-11	BOLD:AAZ0920	MF547400	658[0n]	2010	Misiones 2
Trina geometrina	MACN-Bar-Lep-ct 01036	LEPAR731-11	BOLD:AAZ0920	MF546573	658[0n]	2010	Misiones 2
Trina geometrina	MACN-Bar-Lep-ct 01037	LEPAR732-11	BOLD:ABY8023	MF546635	654[0n]	2010	Misiones 2
Trina geometrina	MACN-Bar-Lep-ct 01887	LEPIG015-11	BOLD:AAZ0920	MF545859	658[0n]	2011	Misiones 2
Trina geometrina	MACN-Bar-Lep-ct 01958	LEPIG080-11	BOLD:AAZ0920	MF545945	658[0n]	2011	Misiones 2
Trina geometrina	MACN-Bar-Lep-ct 01982	LEPIG101-11	BOLD:ABY8023	MF546800	658[0n]	2011	Misiones 2
Trina geometrina	MACN-Bar-Lep-ct 02370	LEPIG440-11	BOLD:AAZ0920	MF546316	658[0n]	2011	Misiones 3
Trina geometrina	MACN-Bar-Lep-ct 02683	LEPPA333-12	BOLD:ABY8023	MF547353	658[0n]	2011	Formosa 2
Trina geometrina	MACN-Bar-Lep-ct 02705	LEPPA348-12	BOLD:ABY8023	MF546280	658[0n]	2011	Formosa 3
Trina geometrina	MACN-Bar-Lep-ct 02821	LEPPA415-12	BOLD:ABY8023	MF545599	658[0n]	2011	Formosa 2
Typhedanus undulatus	MACN-Bar-Lep-ct 03605	LEPPA728-13	BOLD:AAB9757	MF547286	658[0n]	2011	Corrientes 1
Typhedanus undulatus	MACN-Bar-Lep-ct 06054	LEPAR987-14	BOLD:AAB9757	MF546067	658[0n]	2013	Formosa 5
Urbanus albimargo	MACN-Bar-Lep-ct 01888	LEPIG016-11	BOLD:ABZ8278	MF547084	658[0n]	2011	Misiones 2
Urbanus dorantes	MACN-Bar-Lep-ct 02175	LEPIG269-11	BOLD:AAA9148	MF545741	658[0n]	2011	Misiones 3
Urbanus dorantes	MACN-Bar-Lep-ct 02366	LEPIG437-11	BOLD:AAA9148	MF545447	658[0n]	2011	Misiones 3
Urbanus dorantes	MACN-Bar-Lep-ct 03574	LEPPA705-13	BOLD:AAA9148	MF546621	658[0n]	2011	Corrientes 1
Urbanus dorantes	MACN-Bar-Lep-ct 03877	LEPPA519-13	BOLD:AAA9148	MF545471	614[0n]	2012	Formosa 4
Urbanus dorantes	MACN-Bar-Lep-ct 03904	LEPPA536-13	BOLD:AAA9148	MF547245	658[0n]	2012	Formosa 4
Urbanus dorantes	MACN-Bar-Lep-ct 03910	LEPPA540-13	BOLD:AAA9148	MF546859	531[0n]	2012	Formosa 4
Urbanus esta	MACN-Bar-Lep-ct 02078	LEPIG182-11	BOLD:AAA4556	MF545535	658[0n]	2011	Misiones 2
Urbanus esta	MACN-Bar-Lep-ct 02381	LEPIG448-11	BOLD:AAA4556	MF546128	658[0n]	2011	Misiones 3
Urbanus procne	MACN-Bar-Lep-ct 02573	LEPPA228-12	BOLD:AAE0100	MF545729	658[0n]	2011	Formosa 2
Urbanus procne	MACN-Bar-Lep-ct 02679	LEPPA330-12	BOLD:AAE0100	MF546310	658[0n]	2011	Formosa 2
Urbanus procne	MACN-Bar-Lep-ct 02713	LEPPA355-12	BOLD:AAE0100	MF546930	658[0n]	2011	Formosa 3

Urbanus procne	MACN-Bar-Lep-ct 03913	LEPPA542-13	BOLD:AAE0100	MF546087	658[0n]	2012	Formosa 4
Urbanus procne	MACN-Bar-Lep-ct 03928	LEPPA555-13	BOLD:AAE0100	MF546619	601[0n]	2012	Formosa 4
Urbanus procne	MACN-Bar-Lep-ct 03936	LEPPA561-13	BOLD:AAE0100	MF545642	658[0n]	2012	Formosa 4
Urbanus procne	MACN-Bar-Lep-ct 03943	LEPPA568-13	BOLD:AAE0100	MF547388	658[0n]	2012	Formosa 4
Urbanus procne	MACN-Bar-Lep-ct 03974	LEPPA591-13	BOLD:AAE0100	MF546025	658[0n]	2012	Formosa 4
Urbanus procne	MACN-Bar-Lep-ct 06042	LEPAR975-14	BOLD:AAE0100	MF546770	658[0n]	2013	Formosa 5
Urbanus procne	MACN-Bar-Lep-ct 06052	LEPAR985-14	BOLD:AAE0100	MF545924	658[0n]	2013	Formosa 5
Urbanus pronta	MACN-Bar-Lep-ct 02559	LEPIG575-11	BOLD:AAB5662	MF546426	658[0n]	2011	Misiones 3
Urbanus simplicius	MACN-Bar-Lep-ct 01987	LEPIG106-11	BOLD:AAB8414	MF546272	658[0n]	2011	Misiones 2
Urbanus simplicius	MACN-Bar-Lep-ct 03590	LEPPA715-13	BOLD:AAB8414	MF547163	658[0n]	2011	Corrientes 1
Urbanus simplicius	MACN-Bar-Lep-ct 03656	LEPPA755-13	BOLD:AAB8414	MF545803	658[0n]	2011	Corrientes 1
Urbanus simplicius	MACN-Bar-Lep-ct 03680	LEPPA769-13	BOLD:AAB8414	MF546552	658[0n]	2011	Corrientes 1
Urbanus simplicius	MACN-Bar-Lep-ct 03926	LEPPA553-13			0	2012	Formosa 4
Urbanus teleus	MACN-Bar-Lep-ct 01820	LEPAR257-11	BOLD:AAB8486	MF545419	658[0n]	2011	Entre Ríos 3
Urbanus teleus	MACN-Bar-Lep-ct 02385	LEPIG451-11	BOLD:AAB8486	MF545923	658[0n]	2011	Misiones 3
Urbanus zagorus	MACN-Bar-Lep-ct 01666	LEPAR202-11	BOLD:AAH7623	MF546743	658[0n]	2011	Entre Ríos 4
Urbanus zagorus	MACN-Bar-Lep-ct 01670	LEPAR205-11	BOLD:AAH7623	MF545860	658[0n]	2011	Entre Ríos 4
Urbanus zagorus	MACN-Bar-Lep-ct 01751	LEPAR234-11	BOLD:AAH7623	MF546902	658[0n]	2011	Entre Ríos 6
Vacerra caniola	MACN-Bar-Lep-ct 01136	LEPAR770-11	BOLD:AAZ2413	MF546893	658[0n]	2010	Misiones 2
Vacerra caniola	MACN-Bar-Lep-ct 01955	LEPIG077-11	BOLD:AAZ2413	MF545858	658[0n]	2011	Misiones 2
Vanessa braziliensis	MACN-Bar-Lep-ct 00002	LEPAR002-11	BOLD:AAH7884	MF546526	658[0n]	2010	Buenos Aires 21
Vanessa braziliensis	MACN-Bar-Lep-ct 00003	LEPAR003-11	BOLD:AAH7884	MF546471	658[0n]	2010	Buenos Aires 21
Vanessa braziliensis	MACN-Bar-Lep-ct 00040	LEPAR026-11	BOLD:AAH7884	MF546663	658[0n]	2007	Misiones 1
Vanessa braziliensis	MACN-Bar-Lep-ct 00067	LEPAR047-11	BOLD:AAH7884	MF546407	658[0n]	2010	Buenos Aires 21
Vanessa braziliensis	MACN-Bar-Lep-ct 00126	LEPPA101-11	BOLD:AAH7884	MF546529	658[0n]	2010	Buenos Aires 21
Vanessa braziliensis	MACN-Bar-Lep-ct 00162	LEPAR290-11	BOLD:AAH7884	MF546524	658[0n]	2010	Misiones 2
Vanessa braziliensis	MACN-Bar-Lep-ct 01650	LEPAR194-11	BOLD:AAH7884	MF547033	658[0n]	2011	Entre Ríos 4
Vanessa braziliensis	MACN-Bar-Lep-ct 03061	LEPPA174-11	BOLD:ACD7120	MF547178	658[0n]	2011	Buenos Aires 16
Vanessa braziliensis	MACN-Bar-Lep-ct 03067	LEPPA180-11	BOLD:AAH7884	MF547068	658[0n]	2011	Buenos Aires 16
Vanessa braziliensis	MACN-Bar-Lep-ct 06476	LEPPA1064-14			0	2013	Córdoba 4
Vanessa carye	MACN-Bar-Lep-ct 00009	LEPAR004-11	BOLD:ABY9862	MF546791	637[0n]	2010	Buenos Aires 21
Vanessa carye	MACN-Bar-Lep-ct 00010	LEPAR005-11	BOLD:ABY9862	MF545960	637[0n]	2010	Buenos Aires 21
Vanessa carye	MACN-Bar-Lep-ct 00012	LEPAR006-11	BOLD:ABY9862	MF545809	658[0n]	2010	Buenos Aires 21

Vanessa carye	MACN-Bar-Lep-ct 00127	LEPPA102-11	BOLD:ABY9862	MF547292	658[0n]	2010	Buenos Aires 21
Vanessa carye	MACN-Bar-Lep-ct 03112	LEPPA035-11	BOLD:ABY9862	MF547105	658[0n]	2011	Buenos Aires 21
Vanessa carye	MACN-Bar-Lep-ct 06465	LEPPA1053-14			0	2013	Córdoba 4
Vanessa carye	MACN-Bar-Lep-ct 06473	LEPPA1061-14			0	2013	Córdoba 4
Vehilius inca	MACN-Bar-Lep-ct 00188	LEPAR306-11	BOLD:AAC2648	MF546862	658[0n]	2010	Misiones 2
Vehilius stictomenes	MACN-Bar-Lep-ct 02022	LEPIG135-11	BOLD:ACE3344	MF545744	658[0n]	2011	Misiones 2
Vehilius stictomenes	MACN-Bar-Lep-ct 02051	LEPIG159-11	BOLD:ACE3344	MF546703	658[0n]	2011	Misiones 2
Vehilius stictomenes	MACN-Bar-Lep-ct 02417	LEPIG480-11	BOLD:ACE3344	MF545739	658[0n]	2011	Misiones 3
Vettius arva	MACN-Bar-Lep-ct 01873	LEPIG001-11	BOLD:AAZ9763	MF547119	658[0n]	2011	Misiones 2
Vettius lucretius	MACN-Bar-Lep-ct 02799	LEPPA408-12	BOLD:ACA8972	MF546695	658[0n]	2011	Formosa 2
Vettius marcus	MACN-Bar-Lep-ct 02262	LEPIG350-11	BOLD:AAZ9762	MF546689	658[0n]	2011	Misiones 3
Vinius pulcherrimus	MACN-Bar-Lep-ct 01272	LEPAR859-11	BOLD:AAZ4056	MF546727	658[0n]	2010	Misiones 2
Vinius pulcherrimus	MACN-Bar-Lep-ct 01273	LEPAR860-11	BOLD:AAZ4056	MF545581	658[0n]	2010	Misiones 2
Vinius pulcherrimus	MACN-Bar-Lep-ct 01312	LEPAR894-11	BOLD:AAZ4056	MF545399	658[0n]	2010	Misiones 2
Vinius tryhana	MACN-Bar-Lep-ct 00191	LEPAR309-11	BOLD:AAZ4763	MF546066	658[0n]	2010	Misiones 2
Vinius tryhana	MACN-Bar-Lep-ct 01271	LEPAR858-11	BOLD:AAZ4763	MF545966	658[0n]	2010	Misiones 2
Viola minor	MACN-Bar-Lep-ct 00026	LEPAR015-11	BOLD:AAY8200	MF547398	658[0n]	2010	Buenos Aires 21
Viola minor	MACN-Bar-Lep-ct 00418	LEPAR430-11	BOLD:AAY8200	MF546453	658[0n]	2010	Misiones 2
Viola minor	MACN-Bar-Lep-ct 02682	LEPPA332-12	BOLD:AAY8200	MF545512	658[0n]	2011	Formosa 2
Viola minor	MACN-Bar-Lep-ct 02784	LEPPA402-12	BOLD:AAY8200	MF547272	658[0n]	2011	Formosa 2
Viola minor	MACN-Bar-Lep-ct 03146	LEPPA064-11	BOLD:AAY8200	MF545515	658[0n]	2011	Buenos Aires 21
Virga austrinus	MACN-Bar-Lep-ct 00879	LEPAR646-11	BOLD:ACN0833	MF546096	658[0n]	2010	Misiones 2
Virga silvanus	MACN-Bar-Lep-ct 02593	LEPPA248-12	BOLD:ACA8903	MF546688	658[0n]	2011	Formosa 2
Virga silvanus	MACN-Bar-Lep-ct 02632	LEPPA285-12	BOLD:ACA8903	MF546071	658[0n]	2011	Formosa 2
Virga sp.	MACN-Bar-Lep-ct 02634	LEPPA287-12	BOLD:ACA8904	MF546491	658[0n]	2011	Formosa 2
Virga sp.	MACN-Bar-Lep-ct 02642	LEPPA295-12	BOLD:ACA8904	MF545955	658[0n]	2011	Formosa 2
Virga sp.	MACN-Bar-Lep-ct 02761	LEPPA388-12	BOLD:ACA8904	MF546191	658[0n]	2011	Formosa 2
Wallengrenia premnas	MACN-Bar-Lep-ct 01620	LEPAR180-11	BOLD:AAZ8052	MF546340	658[0n]	2011	Entre Ríos 3
Wallengrenia premnas	MACN-Bar-Lep-ct 01644	LEPAR190-11	BOLD:AAZ8052	MF547332	658[0n]	2011	Entre Ríos 4
Wallengrenia premnas	MACN-Bar-Lep-ct 01711	LEPAR219-11	BOLD:AAZ8052	MF547321	658[0n]	2011	Entre Ríos 4
Wallengrenia premnas	MACN-Bar-Lep-ct 03746	LEPPA818-13	BOLD:AAZ8052	MF545394	658[0n]	2011	Corrientes 1
Wallengrenia premnas	MACN-Bar-Lep-ct 03768	LEPPA829-13	BOLD:AAZ8052	MF546612	658[0n]	2011	Corrientes 1
Wallengrenia premnas	MACN-Bar-Lep-ct 03815	LEPPA855-13	BOLD:AAZ8052	MF546430	658[0n]	2011	Corrientes 1

Wallengrenia premnas	MACN-Bar-Lep-ct 06035	LEPAR968-14	BOLD:AAZ8052	MF546317	658[0n]	2013	Formosa 5
Wallengrenia sapuca	MACN-Bar-Lep-ct 03588	LEPPA713-13	BOLD:ACG1322	MF546039	658[0n]	2011	Corrientes 1
Wallengrenia sapuca	MACN-Bar-Lep-ct 03675	LEPPA766-13	BOLD:ACG1322	MF546161	658[0n]	2011	Corrientes 1
Wallengrenia sapuca	MACN-Bar-Lep-ct 03790	LEPPA840-13	BOLD:ACG1322	MF547047	618[1n]	2011	Corrientes 1
Wallengrenia sapuca	MACN-Bar-Lep-ct 06099	LEPAR1032-14	BOLD:ACG1322	MF547168	658[2n]	2013	Formosa 5
Xeniades orchamus	MACN-Bar-Lep-ct 01171	LEPAR797-11	BOLD:AAL5574	MF547222	658[0n]	2010	Misiones 2
Xenophanes tryxus	MACN-Bar-Lep-ct 01976	LEPIG095-11	BOLD:ACF4938	MF546358	658[0n]	2011	Misiones 2
Xenophanes tryxus	MACN-Bar-Lep-ct 02131	LEPIG228-11	BOLD:ACF4938	MF545671	658[0n]	2011	Misiones 2
Xenophanes tryxus	MACN-Bar-Lep-ct 02186	LEPIG279-11	BOLD:ACF4938	MF546146	658[0n]	2011	Misiones 3
Xenophanes tryxus	MACN-Bar-Lep-ct 02206	LEPIG296-11	BOLD:ACF4938	MF546640	658[0n]	2011	Misiones 3
Xenophanes tryxus	MACN-Bar-Lep-ct 02265	LEPIG353-11	BOLD:ACF4938	MF547233	658[0n]	2011	Misiones 3
Xenophanes tryxus	MACN-Bar-Lep-ct 02627	LEPPA281-12	BOLD:ACF4938	MF545998	658[0n]	2011	Formosa 2
Xenophanes tryxus	MACN-Bar-Lep-ct 02649	LEPPA302-12	BOLD:ACF4938	MF545495	658[0n]	2011	Formosa 2
Xenophanes tryxus	MACN-Bar-Lep-ct 02678	LEPPA329-12	BOLD:ACF4938	MF545738	658[0n]	2011	Formosa 2
Xenophanes tryxus	MACN-Bar-Lep-ct 03689	LEPPA776-13	BOLD:ACF4938	MF547335	658[0n]	2011	Corrientes 1
Xenophanes tryxus	MACN-Bar-Lep-ct 03710	LEPPA794-13	BOLD:ACF4938	MF545829	658[0n]	2011	Corrientes 1
Xenophanes tryxus	MACN-Bar-Lep-ct 03735	LEPPA810-13	BOLD:ACF4938	MF545758	658[0n]	2011	Corrientes 1
Yphthimoides affinis	MACN-Bar-Lep-ct 02608	LEPPA263-12	BOLD:ACA8890	MF546276	658[0n]	2011	Formosa 2
Yphthimoides affinis	MACN-Bar-Lep-ct 02623	LEPPA277-12	BOLD:ACA8890	MF546330	658[0n]	2011	Formosa 2
Yphthimoides affinis	MACN-Bar-Lep-ct 02743	LEPPA375-12	BOLD:ACA8890	MF546807	658[0n]	2011	Formosa 3
Yphthimoides affinis	MACN-Bar-Lep-ct 02767	LEPPA393-12	BOLD:ACA8890	MF546522	658[0n]	2011	Formosa 3
Yphthimoides affinis	MACN-Bar-Lep-ct 02773	LEPPA396-12			0	2011	Formosa 3
Yphthimoides affinis	MACN-Bar-Lep-ct 03612	LEPPA732-13	BOLD:ACA8890	MF546266	658[0n]	2011	Corrientes 1
Yphthimoides affinis	MACN-Bar-Lep-ct 03793	LEPPA842-13	COI < 500 bp		362[1n]	2011	Corrientes 1
Yphthimoides celmis	MACN-Bar-Lep-ct 01481	LEPAR117-11	BOLD:AAZ7621	MF546350	658[0n]	2011	Entre Ríos 3
Yphthimoides celmis	MACN-Bar-Lep-ct 01492	LEPAR126-11	BOLD:AAZ7621	MF546568	658[0n]	2011	Entre Ríos 3
Yphthimoides celmis	MACN-Bar-Lep-ct 01515	LEPAR137-11	BOLD:AAZ7621	MF546714	658[0n]	2011	Entre Ríos 4
Yphthimoides celmis	MACN-Bar-Lep-ct 01619	LEPAR179-11	BOLD:AAZ7621	MF545518	658[0n]	2011	Entre Ríos 5
Yphthimoides celmis	MACN-Bar-Lep-ct 01633	LEPAR185-11	BOLD:AAZ7621	MF546343	658[0n]	2011	Entre Ríos 4
Yphthimoides celmis	MACN-Bar-Lep-ct 01656	LEPAR197-11	BOLD:AAZ7621	MF546721	658[0n]	2011	Entre Ríos 4
Yphthimoides celmis	MACN-Bar-Lep-ct 02648	LEPPA301-12	BOLD:AAZ7621	MF547371	658[0n]	2011	Formosa 2
Yphthimoides celmis	MACN-Bar-Lep-ct 02666	LEPPA319-12	BOLD:AAZ7621	MF547204	658[0n]	2011	Formosa 2
Yphthimoides celmis	MACN-Bar-Lep-ct 02675	LEPPA327-12	BOLD:AAZ7621	MF545957	658[0n]	2011	Formosa 2

Yphthimoides celmis	MACN-Bar-Lep-ct 03100	LEPPA023-11	BOLD:AAZ7621	MF546761	658[0n]	2011	Buenos Aires 21
Yphthimoides celmis	MACN-Bar-Lep-ct 03133	LEPPA054-11	BOLD:AAZ7621	MF546165	658[0n]	2011	Buenos Aires 21
Yphthimoides celmis	MACN-Bar-Lep-ct 03147	LEPPA065-11	BOLD:AAZ7621	MF546101	658[0n]	2011	Buenos Aires 21
Yphthimoides celmis	MACN-Bar-Lep-ct 03158	LEPPA073-11	BOLD:AAZ7621	MF546121	658[0n]	2011	Buenos Aires 21
Yphthimoides celmis	MACN-Bar-Lep-ct 03589	LEPPA714-13	BOLD:AAZ7621	MF546637	658[0n]	2011	Corrientes 1
Yphthimoides celmis	MACN-Bar-Lep-ct 03602	LEPPA725-13	BOLD:AAZ7621	MF546670	658[0n]	2011	Corrientes 1
Yphthimoides celmis	MACN-Bar-Lep-ct 03653	LEPPA752-13	BOLD:AAZ7621	MF547384	636[0n]	2011	Corrientes 1
Yphthimoides celmis	MACN-Bar-Lep-ct 03695	LEPPA782-13	BOLD:AAZ7621	MF545568	658[0n]	2011	Corrientes 1
Yphthimoides celmis	MACN-Bar-Lep-ct 03801	LEPPA847-13	BOLD:AAZ7621	MF547194	618[0n]	2011	Corrientes 1
Yphthimoides celmis	MACN-Bar-Lep-ct 03805	LEPPA850-13	BOLD:AAZ7621	MF546010	658[0n]	2011	Corrientes 1
Yphthimoides celmis	MACN-Bar-Lep-ct 03807	LEPPA851-13	BOLD:AAZ7621	MF546422	658[0n]	2011	Corrientes 1
Yphthimoides celmis	MACN-Bar-Lep-ct 06108	LEPAR1041-14	BOLD:AAZ7621	MF545649	658[5n]	2013	Formosa 5
Yphthimoides mimula	MACN-Bar-Lep-ct 00186	LEPAR304-11	BOLD:AAZ0881	MF546911	658[0n]	2010	Misiones 2
Yphthimoides mimula	MACN-Bar-Lep-ct 00857	LEPAR626-11	BOLD:AAZ0881	MF546033	658[0n]	2010	Misiones 2
Yphthimoides mimula	MACN-Bar-Lep-ct 00969	LEPAR676-11	BOLD:AAZ0881	MF546215	658[0n]	2010	Misiones 2
Yphthimoides mimula	MACN-Bar-Lep-ct 01013	LEPAR712-11	BOLD:AAZ0881	MF546975	658[0n]	2010	Misiones 2
Yphthimoides mimula	MACN-Bar-Lep-ct 01306	LEPAR888-11	BOLD:AAZ0881	MF545665	658[0n]	2010	Misiones 2
Yphthimoides mimula	MACN-Bar-Lep-ct 02497	LEPIG530-11	BOLD:AAZ0881	MF546913	658[0n]	2011	Misiones 3
Yphthimoides ordinaria	MACN-Bar-Lep-ct 00196	LEPAR311-11	BOLD:AAZ3922	MF547213	658[0n]	2010	Misiones 2
Yphthimoides ordinaria	MACN-Bar-Lep-ct 00197	LEPAR312-11	BOLD:AAZ3922	MF545494	658[0n]	2010	Misiones 2
Yphthimoides ordinaria	MACN-Bar-Lep-ct 00342	LEPAR384-11	BOLD:AAZ3922	MF547132	658[0n]	2010	Misiones 2
Yphthimoides ordinaria	MACN-Bar-Lep-ct 00546	LEPAR478-11	BOLD:AAZ3922	MF545576	658[0n]	2010	Misiones 2
Yphthimoides ordinaria	MACN-Bar-Lep-ct 01285	LEPAR871-11	BOLD:AAZ3922	MF546189	658[0n]	2010	Misiones 2
Yphthimoides ordinaria	MACN-Bar-Lep-ct 02341	LEPIG420-11	BOLD:AAZ3922	MF545629	658[0n]	2011	Misiones 3
Zabuella tenellus	MACN-Bar-Lep-ct 03682	LEPPA771-13	BOLD:ACG2102	MF545967	618[2n]	2011	Corrientes 1
Zabuella tenellus	MACN-Bar-Lep-ct 03736	LEPPA811-13	BOLD:ACG2102	MF546363	658[0n]	2011	Corrientes 1
Zabuella tenellus	MACN-Bar-Lep-ct 03744	LEPPA816-13	BOLD:ACG2102	MF546977	658[0n]	2011	Corrientes 1
Zabuella tenellus	MACN-Bar-Lep-ct 03747	LEPPA819-13	BOLD:ACG2102	MF545511	658[0n]	2011	Corrientes 1
Zabuella tenellus	MACN-Bar-Lep-ct 03825	LEPPA861-13	BOLD:ACG2102	MF546053	658[0n]	2011	Corrientes 1
Zabuella tenellus	MACN-Bar-Lep-ct 06055	LEPAR988-14	BOLD:ACG2102	MF546684	658[2n]	2013	Formosa 5
Zabuella tenellus	MACN-Bar-Lep-ct 06095	LEPAR1028-14	BOLD:ACG2102	MF545927	658[0n]	2013	Formosa 5
Zabuella tenellus	MACN-Bar-Lep-ct 06100	LEPAR1033-14	BOLD:ACG2102	MF547108	658[0n]	2013	Formosa 5
Zaretis sp.	MACN-Bar-Lep-ct 07099	LEPAR1177-15			0	1998	Misiones 12

Zaretis strigosus	MACN-Bar-Lep-ct 00978	LEPAR684-11	BOLD:AAA8916	MF546440	658[0n]	2010	Misiones 2
Zaretis strigosus	MACN-Bar-Lep-ct 01146	LEPAR778-11	BOLD:AAA8916	MF547246	658[0n]	2010	Misiones 2
Zaretis strigosus	MACN-Bar-Lep-ct 02714	LEPPA356-12	BOLD:AAA8917	MF546735	658[0n]	2011	Formosa 3
Zaretis strigosus	MACN-Bar-Lep-ct 02744	LEPPA376-12	BOLD:AAA8917	MF546111	658[0n]	2011	Formosa 3
Zaretis strigosus	MACN-Bar-Lep-ct 07091	LEPAR1169-15			0	1996	Misiones 8
Zaretis strigosus	MACN-Bar-Lep-ct 07097	LEPAR1175-15			0	1996	Misiones 8
Zenis jebus	MACN-Bar-Lep-ct 01342	LEPAR923-11	BOLD:AAZ4707	MF545522	658[0n]	2010	Misiones 2
Zenis jebus	MACN-Bar-Lep-ct 01343	LEPAR924-11	BOLD:AAZ4707	MF546764	658[0n]	2010	Misiones 2
Zera hyacinthinus	MACN-Bar-Lep-ct 00822	LEPAR598-11	BOLD:AAC1668	MF546416	658[0n]	2010	Misiones 2
Zera tetrastigma	MACN-Bar-Lep-ct 00878	LEPAR645-11	BOLD:AAZ4958	MF546824	658[0n]	2010	Misiones 2
Ziegleria hesperitis	MACN-Bar-Lep-ct 02556	LEPIG573-11	BOLD:AAD4593	MF546149	658[0n]	2011	Misiones 3
Zizula cyna	MACN-Bar-Lep-ct 01611	LEPAR174-11	BOLD:AAZ7523	MF545879	658[0n]	2011	Entre Ríos 5
Zizula cyna	MACN-Bar-Lep-ct 02089	LEPIG192-11	BOLD:AAZ7523	MF545534	658[0n]	2011	Misiones 2
Zizula cyna	MACN-Bar-Lep-ct 02102	LEPIG204-11	BOLD:AAZ7523	MF546598	658[0n]	2011	Misiones 2
Zizula cyna	MACN-Bar-Lep-ct 02181	LEPIG275-11	BOLD:AAZ7523	MF545528	658[0n]	2011	Misiones 3
Zizula cyna	MACN-Bar-Lep-ct 02257	LEPIG345-11	BOLD:AAZ7523	MF545467	658[0n]	2011	Misiones 3
Zizula cyna	MACN-Bar-Lep-ct 02309	LEPIG393-11	BOLD:AAZ7523	MF547234	658[0n]	2011	Misiones 3
Zizula cyna	MACN-Bar-Lep-ct 02837	LEPPA422-12	BOLD:AAZ7523	MF546618	658[0n]	2011	Formosa 2
Zopyrion evenor	MACN-Bar-Lep-ct 01490	LEPAR124-11	BOLD:AAZ8857	MF545582	658[0n]	2011	Entre Ríos 3
Zopyrion evenor	MACN-Bar-Lep-ct 01497	LEPAR128-11	BOLD:AAZ8857	MF546464	658[0n]	2011	Entre Ríos 3
Zopyrion evenor	MACN-Bar-Lep-ct 01631	LEPAR184-11	BOLD:AAZ8857	MF547072	658[0n]	2011	Entre Ríos 4
Zopyrion evenor	MACN-Bar-Lep-ct 01634	LEPAR186-11	BOLD:AAZ8857	MF545996	658[0n]	2011	Entre Ríos 4
Zopyrion evenor	MACN-Bar-Lep-ct 01658	LEPAR199-11	BOLD:AAZ8857	MF546269	658[0n]	2011	Entre Ríos 4

**H. feronia* has no BIN assignment because it has more than 1% of ambiguous calls.

Table B. Detailed information on the sampling localities mentioned in Table A.

Locality	Region/Department	Sector	Site	Lat.	Lon.
Buenos Aires 1	Capital Federal	Agronomía		-34.596	-58.480
Buenos Aires 2	Capital Federal	Agronomía		-34.593	-58.489
Buenos Aires 3	Capital Federal	Agronomía		-34.591	-58.481
Buenos Aires 4	Capital Federal	Barracas		-34.650	-58.383
Buenos Aires 5	Capital Federal	Flores		-34.633	-58.467
Buenos Aires 6	Capital Federal	Floresta		-34.636	-58.484
Buenos Aires 7	Capital Federal	Parque Avellaneda		-34.646	-58.479
Buenos Aires 8	Capital Federal	Reserva Ecológica Costanera Sur		-34.609	-58.357
Buenos Aires 9	Capital Federal	Villa Devoto		-34.600	-58.517
Buenos Aires 10	Partido de Campana	Reserva Natural Otamendi		-34.219	-58.899
Buenos Aires 11	Partido de Campana	Reserva Natural Otamendi		-34.227	-58.898
Buenos Aires 12	Partido de Chascomús	Chascomús		-35.596	-57.976
Buenos Aires 13	Partido de Escobar	El Cazador		-34.308	-58.758
Buenos Aires 14	Partido de Escobar	Escobar		-34.247	-58.731
Buenos Aires 15	Partido de Exaltación de la Cruz	Los Cardales		-34.331	-58.976
Buenos Aires 16	Partido de la Costa	San Bernardo		-36.687	-56.680
Buenos Aires 17	Partido de La Plata	Isla Martín García		-34.188	-58.246
Buenos Aires 18	Partido de La Plata	Isla Martín García		-34.184	-58.256
Buenos Aires 19	Partido de La Plata	Isla Martín García		-34.189	-58.255
Buenos Aires 20	Partido de La Plata	Isla Martín García		-34.190	-58.251
Buenos Aires 21	Partido de Magdalena	Estancia El Destino		-35.130	-57.380
Buenos Aires 22	Partido de Pilar	Fatima		-34.429	-58.990
Buenos Aires 23	Partido de Pilar	Zelaya		-34.366	-58.902
Buenos Aires 24	Partido de San Fernando	Río Carabelas		-34.158	-58.745
Buenos Aires 25	Partido de San Fernando	Victoria		-34.455	-58.552
Buenos Aires 26	Partido de San Isidro	Martínez		-34.499	-58.538
Buenos Aires 27	Partido de San Isidro	Reserva Municipal Ribera Norte		-34.470	-58.494
Buenos Aires 28	Partido de San Martin	Villa Lynch		-34.593	-58.536
Buenos Aires 29	Partido de Vicente López	Reserva Ecológica		-34.493	-58.479
Chaco 1	Departamento Bermejo	La Fidelidad	Río Bermejo	-24.836	-61.104

Chaco 2	Departamento General Güemes	La Fidelidad	Paraje La Armonía	-25.180	-61.094
Chaco 3	Departamento General Güemes	La Fidelidad	Paraje La Providencia	-25.032	-61.263
Córdoba 1	Departamento de Punilla	Capilla del Monte		-30.879	-64.534
Córdoba 2	Departamento de Punilla	Capilla del Monte		-30.865	-64.521
Córdoba 3	Departamento Río Seco	Cerro Colorado	Río Los Tartagos	-30.099	-63.933
Córdoba 4	Departamento San Alberto	Parque Nacional Quebrada del Condorito	Seccional Trinidad	-31.690	-64.854
Córdoba 5	Departamento Tulumba	San Jose de las Salinas		-29.998	-64.607
Corrientes 1	Departamento Capital	San Cayetano	Estación Biológica Corrientes	-27.553	-58.680
Corrientes 2	Departamento Concepción	Estancia El Transito		-28.422	-57.697
Corrientes 3	Departamento Mburucuyá	Parque Nacional Mburucuyá	Sendero Yatay	-28.020	-58.036
Corrientes 4	Departamento Mburucuyá	Parque Nacional Mburucuyá	Sendero Yatay	-28.014	-58.013
Corrientes 5	Departamento Santo Tome	Estancia Virocay	Casco	-28.272	-55.963
Entre Ríos 1	Departamento Colon	Parque Nacional El Palmar	Arroyo Palmar	-31.894	-58.237
Entre Ríos 2	Departamento de Colon	Liebig		-32.150	-58.189
Entre Ríos 3	Departamento de Colon	Parque Nacional El Palmar	Intendencia	-31.869	-58.208
Entre Ríos 4	Departamento de Colon	Parque Nacional El Palmar	La Glorieta	-31.879	-58.269
Entre Ríos 5	Departamento de Colon	Parque Nacional El Palmar		-31.888	-58.275
Entre Ríos 6	Departamento de Colon	Parque Nacional El Palmar	Area intangible	-31.893	-58.270
Entre Ríos 7	Departamento Gualeguaychú	Reserva Privada Malabrigo		-32.904	-58.765
Entre Ríos 8	Departamento Gualeguaychú	Salto de Mendez	Espinillar	-32.823	-58.499
Entre Ríos 9	Departamento Islas del Ibicuy	Ceibas	Arroyo Nancay	-33.439	-58.656
Entre Ríos 10	Departamento Islas del Ibicuy	Ceibas		-33.489	-58.710
Formosa 1	Departamento de Bermejo	Reserva Natural Formosa	Seccional Maradona	-24.180	-61.430
Formosa 2	Departamento de Pilcomayo	Parque Nacional Rio Pilcomayo	Seccional Estero Poi	-25.121	-58.172
Formosa 3	Departamento de Pilcomayo	Parque Nacional Rio Pilcomayo	Parador Yaguareté	-25.015	-58.131
Formosa 4	Departamento Laishi	Estancia El Bagual	Reserva Ecológica	-26.180	-58.940
Formosa 5	Departamento Laishi	Estancia El Bagual	Reserva Ecológica	-26.305	-58.830
Misiones 1	Departamento de Cainguas			-27.090	-54.922
Misiones 2	Departamento de Iguazú	Parque Nacional Iguazú	Seccional Yacuí	-25.680	-54.170
Misiones 3	Departamento de Iguazú	Parque Nacional Iguazú	Seccional Timbó	-25.715	-54.441
Misiones 4	Departamento de Iguazú	Puerto Iguazú		-25.594	-54.588
Misiones 5	Departamento Gral. Belgrano	Reserva Natural Estricta San Antonio	INTA San Antonio	-26.038	-53.779
Misiones 6	Departamento Gral. Belgrano	Reserva Natural Estricta San Antonio	San Antonio	-26.055	-53.751

Misiones 7	Departamento Gral. Belgrano	Reserva Privada Yacutinga		-25.589	-54.074
Misiones 8	Departamento de Iguazú	Parque Nacional Iguazú	Sendero Macuco	-25.655	-54.457
Misiones 9	Departamento de Iguazú	Parque Nacional Iguazú	Seccional Yacuí	-25.681	-54.161
Misiones 10	Departamento de Iguazú	Parque Provincial Urugua-í	Seccional Uruzú	-25.857	-54.168
Misiones 11	Departamento de Iguazú	Puerto Iguazú		-25.598	-54.570
Misiones 12	Departamento de Iguazú	Puerto Península		-25.667	-54.650
Misiones 13	Departamento de Iguazú	Puerto Península		-25.657	-54.568
Misiones 14	Departamento Oberá	Campo Ramón	El Bonito	-27.453	-54.929
Misiones 15	Departamento San Ignacio	Reserva Privada Osununú		-27.286	-55.570
Misiones 16	NA	NA	NA	NA	NA

Table C. Correspondence between species and MOTUs boundaries for each clustering algorithm for all species analyzed. MA: MATCH, SP: SPLIT, ME: MERGE, MI: MIXTURE. The numbers in parenthesis indicate the amount of MOTUs into which a reference species was split. We also inform for each species the number of sequences (N), the mean and maximum instraspecific distances, and the minimum distance (K2P, %) to the nearest non-conspecific (i.e. nearest neighbor, NN).

Species	N	Mean distance	Max. distance	Min. distance to NN	RESL	TCS 90%	TCS 95%	ABGD initial	ABGD recursive P = 1.29	ABGD recursive P = 0.77	ABGD recursive P = 0.46	BINs
Achlyodes busirus	6	0.99	2.96	8.52	SP (2)	SP (2)	SP (2)	SP (2)	SP (2)	SP (2)	SP (2)	SP (2)
Actinote brylla	1	NA	NA	4.66	MA	MA	MA	MA	MA	MA	MA	MA
Actinote mamita	3	0.41	0.46	7.45	MA	MA	MA	MA	MA	MA	MA	MA
Actinote melanisans	2	0.30	0.30	1.07	ME	ME	ME	ME	ME	ME	ME	ME
Actinote pellenea	27	0.43	0.92	1.07	ME	ME	ME	ME	ME	ME	ME	ME
Adelotypa bolena	1	NA	NA	9.72	MA	MA	MA	MA	MA	MA	MA	MA
Adelpha abia	2	0.00	0.00	6.54	MA	MA	MA	MA	MA	MA	MA	MA
Adelpha calliphane	1	NA	NA	6.88	MA	MA	MA	MA	MA	MA	MA	MA
Adelpha epizygis	8	0.39	0.76	7.04	MA	MA	MA	MA	MA	MA	MA	MA
Adelpha iphicleola	5	0.18	0.30	3.29	MA	MA	MA	MA	MA	MA	MA	MA
Adelpha malea	8	0.08	0.30	6.88	MA	MA	MA	MA	MA	MA	MA	MA
Adelpha melona	1	NA	NA	7.21	MA	MA	MA	MA	MA	MA	MA	MA
Adelpha mythra	1	NA	NA	4.89	MA	MA	MA	MA	MA	MA	MA	MA
Adelpha serpa	2	0.00	0.00	6.54	MA	MA	MA	MA	MA	MA	MA	MA
Adelpha syma	4	0.00	0.00	4.89	MA	MA	MA	MA	MA	MA	MA	MA
Adelpha thessalia	4	0.18	0.30	3.29	MA	MA	MA	MA	MA	MA	MA	MA
Adelpha zea	1	NA	NA	6.54	MA	MA	MA	MA	MA	MA	MA	MA
Adlerodea modesta	1	NA	NA	8.69	MA	MA	MA	MA	MA	MA	MA	MA
Aeria olena	8	0.12	0.30	8.71	MA	MA	MA	MA	MA	MA	MA	MA
Aethilla echina	8	0.04	0.15	8.19	MA	MA	MA	MA	MA	MA	MA	MA
Agraulis vanillae	18	0.38	0.96	7.85	MA	MA	MA	MA	MA	MA	MA	MA
Alera metallica	1	NA	NA	6.71	MA	MA	MA	MA	MA	MA	MA	MA
Anartia amathea	18	0.23	0.76	8.72	MA	MA	MA	MA	MA	MA	MA	MA
Anartia jatrophae	13	0.16	0.46	8.72	MA	MA	MA	MA	MA	MA	MA	MA
Anastrus sempiternus	3	0.10	0.15	7.85	MA	MA	MA	MA	MA	MA	MA	MA

Ancyloxypha nitedula	1	NA	NA	8.52	MA	MA	MA	MA	MA	MA	MA	MA
Anisochoria sublimbata	2	0.00	0.00	7.87	MA	MA	MA	MA	MA	MA	MA	MA
Anthanassa frisia	12	0.23	0.77	6.78	MA	MA	MA	MA	MA	MA	MA	MA
Anthoptus epictetus	1	NA	NA	7.68	MA	MA	MA	MA	MA	MA	MA	MA
Antigonus liborius	1	NA	NA	7.68	MA	MA	MA	MA	MA	MA	MA	MA
Aphrissa statira	8	0.26	0.62	6.01	MA	MA	MA	MA	MA	MA	SP (2)	MA
Arawacus ellida	1	NA	NA	5.87	MA	MA	MA	MA	MA	MA	MA	MA
Arawacus melibaeus	5	0.06	0.15	4.72	MA	MA	MA	MA	MA	MA	MA	MA
Arawacus separata	7	0.42	1.07	4.72	MA	MA	MA	MA	MA	MA	MA	MA
Archaeoprepona demophon	1	NA	NA	8.21	MA	MA	MA	MA	MA	MA	MA	MA
Archaeoprepona demophoon	3	0.10	0.15	8.21	MA	MA	MA	MA	MA	MA	MA	MA
Argon lota	1	NA	NA	8.96	MA	MA	MA	MA	MA	MA	MA	MA
Ariconias glaphyra	2	0.15	0.15	8.89	MA	MA	MA	MA	MA	MA	MA	MA
Aricoris chilensis	8	0.59	1.08	4.39	MA	MA	MA	MA	MA	MA	MA	MA
Aricoris indistincta	2	0.00	0.00	2.33	MA	ME	MA	MA	MA	MA	MA	MA
Aricoris notialis	1	NA	NA	4.39	MA	MA	MA	MA	MA	MA	MA	MA
Aricoris signata	6	0.05	0.15	2.33	MA	ME	MA	MA	MA	MA	MA	MA
Ascia monuste	12	0.11	0.32	9.04	MA	MA	MA	MA	MA	MA	MA	MA
Astraptes anaphus	3	0.30	0.30	7.18	MA	MA	MA	MA	MA	MA	MA	MA
Astraptes enotrus	1	NA	NA	10.42	MA	MA	MA	MA	MA	MA	MA	MA
Astraptes fulgerator	3	0.82	1.23	6.84	MA	MA	MA	MA	MA	MA	MA	SP (2)
Barbicornis basilis	8	0.04	0.17	6.95	MA	MA	MA	MA	MA	MA	MA	MA
Battus polydamas	5	0.00	0.00	11.13	MA	MA	MA	MA	MA	MA	MA	MA
Biblis hyperia	9	0.48	0.92	10.07	MA	MA	MA	MA	MA	MA	MA	MA
Brangas getus	1	NA	NA	6.18	MA	MA	MA	MA	MA	MA	MA	MA
Caeruleuptychia helena	2	7.24	7.24	8.03	SP (2)	SP (2)	SP (2)	SP (2)	SP (2)	SP (2)	SP (2)	SP (2)
Calephelis aymaran	1	NA	NA	6.51	MA	MA	MA	MA	MA	MA	MA	MA
Caligo illioneus	1	NA	NA	9.39	MA	MA	MA	MA	MA	MA	MA	MA
Callicore hydaspes	7	0.00	0.00	9.20	MA	MA	MA	MA	MA	MA	MA	MA
Callimormus interpunctata	1	NA	NA	8.52	MA	MA	MA	MA	MA	MA	MA	MA
Callimormus simplicius	2	0.00	0.00	8.86	MA	MA	MA	MA	MA	MA	MA	MA
Calpodes ethlius	2	0.48	0.48	8.01	MA	MA	MA	MA	MA	MA	MA	MA
Calycopis caulonia	7	1.52	2.95	0.00	MI (2)	ME	MI (2)	MI (2)	MI (2)	MI (2)	MI (2)	MI (2)

Calycopis sp. 1	1	NA	NA	2.00	MA	ME	MA	ME	ME	ME	ME	MA
Calycopis sp. 2	5	3.79	5.72	0.00	MI (3)	MI (3)	MI (3)	MI (3)	MI (3)	MI (3)	MI (3)	MI (3)
Camptopleura auxo	1	NA	NA	10.42	MA	MA	MA	MA	MA	MA	MA	MA
Caria marsyas	5	0.06	0.16	5.70	MA	MA	MA	MA	MA	MA	MA	MA
Caria plutargus	7	0.00	0.00	5.70	MA	MA	MA	MA	MA	MA	MA	MA
Carrhenes canescens	3	0.10	0.15	6.67	MA	MA	MA	MA	MA	MA	MA	MA
Catoblepia amphirhoe	5	0.46	0.61	6.88	MA	MA	MA	MA	MA	MA	MA	MA
Catonephele numilia	3	0.10	0.15	7.35	MA	MA	MA	MA	MA	MA	MA	MA
Celmia celmus	3	0.10	0.15	4.87	MA	MA	MA	MA	MA	MA	MA	MA
Chalodeta theodora	1	NA	NA	7.84	MA	MA	MA	MA	MA	MA	MA	MA
Chamaelimnas briola	3	0.00	0.00	6.95	MA	MA	MA	MA	MA	MA	MA	MA
Chioides catillus	9	0.07	0.30	6.20	MA	MA	MA	MA	MA	MA	MA	MA
Chiomara asychis	5	0.24	0.61	6.51	MA	MA	MA	MA	MA	MA	MA	MA
Chiomara mithrax	1	NA	NA	10.42	MA	MA	MA	MA	MA	MA	MA	MA
Chlorostrymon simaethis	1	NA	NA	4.71	MA	MA	MA	MA	MA	MA	MA	MA
Chlosyne lacinia	10	0.06	0.30	10.43	MA	MA	MA	MA	MA	MA	MA	MA
Cobalopsis catocala	3	0.00	0.00	8.20	MA	MA	MA	MA	MA	MA	MA	MA
Cobalopsis miaba	2	0.00	0.00	3.60	MA	MA	MA	MA	MA	MA	MA	MA
Cobalopsis nero	3	0.20	0.30	3.60	MA	MA	MA	MA	MA	MA	MA	MA
Cobalopsis sp. 1	2	0.62	0.62	6.84	MA	MA	MA	MA	MA	MA	MA	MA
Codatractus aminias	5	0.12	0.30	8.35	MA	MA	MA	MA	MA	MA	MA	MA
Cogia abdul	1	NA	NA	5.85	MA	MA	MA	MA	MA	MA	MA	MA
Colias lesbia	6	0.00	0.00	9.21	MA	MA	MA	MA	MA	MA	MA	MA
Colobura dirce	5	0.49	0.77	9.72	MA	MA	MA	MA	MA	MA	MA	MA
Conga chydaea	1	NA	NA	4.89	MA	MA	MA	MA	MA	MA	MA	MA
Conga iheringii	5	0.00	0.00	4.89	MA	MA	MA	MA	MA	MA	MA	MA
Consul fabius	4	0.08	0.16	9.41	MA	MA	MA	MA	MA	MA	MA	MA
Contrafacia imma	1	NA	NA	4.39	MA	MA	MA	MA	MA	MA	MA	MA
Corticea corticea	3	0.20	0.30	5.05	MA	MA	MA	MA	MA	MA	MA	MA
Corticea lysias	6	0.05	0.15	5.05	MA	MA	MA	MA	MA	MA	MA	MA
Corticea sp.	2	0.00	0.00	6.86	MA	MA	MA	MA	MA	MA	MA	MA
Cybdelis phaesyla	1	NA	NA	9.72	MA	MA	MA	MA	MA	MA	MA	MA
Cycloglypha caeruleonigra	2	0.00	0.00	8.53	MA	MA	MA	MA	MA	MA	MA	MA

Cymaenes alumna	1	NA	NA	4.40	MA	MA	MA	MA	MA	MA	MA	MA
Cymaenes cavalla	3	0.10	0.15	4.31	MA	MA	MA	MA	MA	MA	MA	MA
Cymaenes gisca	11	0.08	0.15	2.17	MA	ME	MA	ME	ME	MA	MA	MA
Cymaenes laureolus	5	0.09	0.15	1.23	MA	ME	ME	ME	ME	MA	MA	MA
Cymaenes lepta	5	0.06	0.15	1.23	MA	ME	ME	ME	ME	MA	MA	MA
Cymaenes odilia	5	0.18	0.30	3.76	MA	MA	MA	MA	MA	MA	MA	MA
Dalla diraspes	2	0.00	0.00	10.07	MA	MA	MA	MA	MA	MA	MA	MA
Danaus eresimus	12	0.34	0.76	6.86	MA	MA	MA	MA	MA	MA	MA	MA
Danaus erippus	12	0.27	0.76	6.86	MA	MA	MA	MA	MA	MA	MA	MA
Danaus gilippus	7	0.32	0.62	7.56	MA	MA	MA	MA	MA	MA	MA	MA
Decinea dama	3	0.00	0.00	9.29	MA	MA	MA	MA	MA	MA	MA	MA
Denivia hemon	1	NA	NA	6.09	MA	MA	MA	MA	MA	MA	MA	MA
Diaethria candrena	9	0.07	0.30	5.36	MA	MA	MA	MA	MA	MA	MA	MA
Diaethria clymena	11	0.13	0.46	5.36	MA	MA	MA	MA	MA	MA	MA	MA
Dione juno	9	0.00	0.00	8.20	MA	MA	MA	MA	MA	MA	MA	MA
Dircenna dero	10	0.06	0.30	5.38	MA	MA	MA	MA	MA	MA	MA	MA
Dismorphia amphione	1	NA	NA	13.14	MA	MA	MA	MA	MA	MA	MA	MA
Doxocopa agathina	5	0.55	0.92	6.91	MA	MA	MA	MA	MA	SP (2)	SP (2)	MA
Doxocopa kallina	9	0.52	1.08	6.91	MA	MA	MA	MA	MA	MA	MA	MA
Doxocopa laurentia	8	0.85	1.39	9.74	MA	MA	MA	MA	MA	MA	MA	MA
Doxocopa linda	9	0.03	0.15	10.96	MA	MA	MA	MA	MA	MA	MA	MA
Doxocopa zunilda	7	0.18	0.31	9.04	MA	MA	MA	MA	MA	MA	MA	MA
Dryadula phaetusa	8	0.45	1.23	9.38	MA	MA	MA	MA	MA	MA	MA	MA
Dryas iulia	12	0.55	1.39	8.52	MA	MA	MA	MA	MA	MA	MA	MA
Dynamine aerata	2	0.77	0.77	8.02	MA	MA	MA	MA	MA	MA	MA	MA
Dynamine agacles	6	0.05	0.15	7.17	MA	MA	MA	MA	MA	MA	MA	MA
Dynamine artemisia	8	0.70	1.54	8.23	MA	MA	MA	MA	MA	MA	MA	MA
Dynamine athemon	8	0.33	0.61	4.40	MA	MA	MA	MA	MA	MA	MA	MA
Dynamine coenus	8	0.42	0.92	9.04	MA	MA	MA	MA	MA	MA	MA	MA
Dynamine meridionalis	1	NA	NA	4.40	MA	MA	MA	MA	MA	MA	MA	MA
Dynamine myrrhina	5	0.12	0.30	9.74	MA	MA	MA	MA	MA	MA	MA	MA
Dynamine postverta	7	0.36	0.61	4.58	MA	MA	MA	MA	MA	MA	MA	MA
Dynamine tithia	7	0.00	0.00	9.05	MA	MA	MA	MA	MA	MA	MA	MA

Eantis thraso	11	1.41	3.63	8.19	SP (2)	SP (2)	SP (2)	SP (2)	SP (3)	SP (3)	SP (3)	SP (3)
Ebrietas anacreon	8	0.07	0.15	8.70	MA	MA	MA	MA	MA	MA	MA	MA
Ebrietas infanda	1	NA	NA	8.53	MA	MA	MA	MA	MA	MA	MA	MA
Ectima thecla	6	0.00	0.00	9.21	MA	MA	MA	MA	MA	MA	MA	MA
Elbella adonis	4	0.08	0.15	8.04	MA	MA	MA	MA	MA	MA	MA	MA
Elbella lamprus	1	NA	NA	8.04	MA	MA	MA	MA	MA	MA	MA	MA
Electrostrymon endymion	1	NA	NA	7.00	MA	MA	MA	MA	MA	MA	MA	MA
Emesis diogenia	7	0.17	0.61	7.68	MA	MA	MA	MA	MA	MA	SP (2)	MA
Emesis mandana	3	0.41	0.61	0.92	ME	ME	ME	ME	ME	ME	ME	ME
Emesis ocypore	9	0.54	1.10	8.56	MA	MA	MA	MA	MA	MA	MA	MA
Emesis russula	6	0.97	1.70	0.92	ME	ME	ME	ME	ME	ME	ME	ME
Enantia clarissa	1	NA	NA	4.89	MA	MA	MA	MA	MA	MA	MA	MA
Enantia lina	7	0.13	0.30	4.89	MA	MA	MA	MA	MA	MA	MA	MA
Enos thara	1	NA	NA	5.85	MA	MA	MA	MA	MA	MA	MA	MA
Epargyreus exadeus	3	0.51	0.77	1.07	ME	ME	ME	ME	ME	ME	ME	ME
Epargyreus socus	5	4.63	7.44	1.07	MI (3)	MI (3)	MI (3)	MI (3)	MI (3)	MI (3)	MI (3)	MI (3)
Epargyreus tmolis	5	0.12	0.30	1.23	ME	ME	ME	ME	ME	ME	ME	ME
Epiphile hubneri	8	0.38	0.92	7.22	MA	MA	MA	MA	MA	MA	MA	MA
Epiphile orea	1	NA	NA	7.22	MA	MA	MA	MA	MA	MA	MA	MA
Episcada hymenaea	7	0.09	0.30	3.60	MA	MA	MA	MA	MA	MA	MA	MA
Episcada sylvo	13	0.15	0.30	3.60	MA	MA	MA	MA	MA	MA	MA	MA
Epityches eupompe	3	0.00	0.00	7.18	MA	MA	MA	MA	MA	MA	MA	MA
Eresia lansdorfi	8	0.24	0.76	6.17	MA	MA	MA	MA	MA	MA	MA	MA
Erynnis funeralis	2	0.00	0.00	4.87	MA	MA	MA	MA	MA	MA	MA	MA
Eryphanis reevesii	1	NA	NA	11.23	MA	MA	MA	MA	MA	MA	MA	MA
Eteona tisiphone	1	NA	NA	10.95	MA	MA	MA	MA	MA	MA	MA	MA
Eueides aliphera	9	0.16	0.30	6.17	MA	MA	MA	MA	MA	MA	MA	MA
Eueides isabella	3	0.10	0.15	6.17	MA	MA	MA	MA	MA	MA	MA	MA
Eunica eburnea	2	0.15	0.15	1.40	MA	ME	ME	ME	ME	ME	ME	MA
Eunica margarita	3	0.30	0.30	1.40	MA	ME	ME	ME	ME	ME	ME	MA
Eunica tatila	3	0.10	0.15	5.24	MA	MA	MA	MA	MA	MA	MA	MA
Euptoieta hortensia	4	0.69	0.92	10.42	MA	MA	MA	MA	MA	MA	MA	MA
Eurema agave	3	0.00	0.00	12.41	MA	MA	MA	MA	MA	MA	MA	MA

Eurema albula	8	1.22	2.49	10.07	MA	MA	SP (2)	MA	SP (3)	SP (3)	SP (3)	MA
Eurema arbela	6	0.10	0.30	9.73	MA	MA	MA	MA	MA	MA	MA	MA
Eurema deva	18	0.10	0.31	10.86	MA	MA	MA	MA	MA	MA	MA	MA
Eurema elathea	11	1.61	7.24	9.21	SP (2)	SP (2)	SP (2)	SP (2)	SP (2)	SP (2)	SP (2)	SP (2)
Euryades corethrus	4	0.15	0.30	4.44	MA	MA	MA	MA	MA	MA	MA	MA
Euryades duponchelii	6	0.05	0.15	4.44	MA	MA	MA	MA	MA	MA	MA	MA
Eutocus sp. 1	1	NA	NA	8.19	MA	MA	MA	MA	MA	MA	MA	MA
Eutocus vetulus	1	NA	NA	7.68	MA	MA	MA	MA	MA	MA	MA	MA
Evansiella cordela	1	NA	NA	5.85	MA	MA	MA	MA	MA	MA	MA	MA
Exoplisia myrtis	1	NA	NA	7.34	MA	MA	MA	MA	MA	MA	MA	MA
Fountainea cratias	1	NA	NA	2.80	MA	MA	MA	MA	MA	MA	MA	MA
Fountainea ryphea	2	0.15	0.15	2.80	MA	MA	MA	MA	MA	MA	MA	MA
Gesta austerus	9	0.16	0.61	4.87	MA	MA	MA	MA	MA	MA	MA	MA
Gesta gesta	3	0.10	0.15	6.02	MA	MA	MA	MA	MA	MA	MA	MA
Glutophrissa drusilla	2	0.31	0.31	10.31	MA	MA	MA	MA	MA	MA	MA	MA
Godartiana muscosa	7	1.32	2.81	7.17	SP (2)	MA	SP (2)	SP (2)	SP (2)	SP (2)	SP (2)	SP (2)
Gorgythion begga	7	1.26	2.64	3.44	SP (2)	SP (2)	SP (2)	SP (2)	SP (2)	SP (2)	SP (2)	SP (2)
Gorgythion beggina	5	0.63	1.54	3.44	MA	MA	MA	MA	SP (2)	SP (2)	SP (2)	SP (2)
Grais stigmaticus	1	NA	NA	9.92	MA	MA	MA	MA	MA	MA	MA	MA
Haematera pyrame	9	0.14	0.63	9.79	MA	MA	MA	MA	MA	MA	MA	MA
Hamadryas amphinome	5	0.86	1.39	5.20	SP (2)	MA	MA	MA	MA	MA	MA	SP (2)
Hamadryas epinome	9	0.07	0.30	5.70	MA	MA	MA	MA	MA	MA	MA	MA
Hamadryas februa	5	0.58	1.08	5.09	MA	MA	MA	MA	MA	MA	MA	MA
Hamadryas feronia	1	NA	NA	5.09	MA	MA	MA	MA	MA	MA	MA	NA*
Hamadryas fornax	4	0.33	0.46	6.03	MA	MA	MA	MA	MA	MA	MA	MA
Harveyope tinea	3	0.00	0.00	8.69	MA	MA	MA	MA	MA	MA	MA	MA
Haywardella edmondsii	3	0.51	0.77	7.72	MA	MA	MA	MA	MA	MA	MA	MA
Helias phalaenoides	4	0.23	0.30	9.55	MA	MA	MA	MA	MA	MA	MA	MA
Heliconius besckei	1	NA	NA	3.93	MA	MA	MA	MA	MA	MA	MA	MA
Heliconius erato	16	1.12	3.45	8.37	SP (2)	MA	SP (2)	SP (2)	SP (2)	SP (2)	SP (2)	SP (2)
Heliconius ethilla	1	NA	NA	3.93	MA	MA	MA	MA	MA	MA	MA	MA
Heliopetes alana	10	0.07	0.15	5.60	MA	MA	MA	MA	MA	MA	MA	MA
Heliopetes arsalte	10	0.34	0.77	5.72	MA	MA	MA	MA	MA	MA	MA	MA

Heliopetes libra	4	0.00	0.00	6.68	MA	MA	MA	MA	MA	MA	MA	MA
Heliopetes ochroleuca	3	0.00	0.00	5.60	MA	MA	MA	MA	MA	MA	MA	MA
Heliopetes omrina	20	0.52	1.80	6.88	SP (2)	MA	MA	MA	SP (2)	SP (2)	SP (2)	SP (2)
Heliopyrgus americanus	2	0.00	0.00	5.86	MA	MA	MA	MA	MA	MA	MA	MA
Heliopyrgus domicella	5	0.00	0.00	5.86	MA	MA	MA	MA	MA	MA	MA	MA
Hemiargus hanno	9	0.00	0.00	7.85	MA	MA	MA	MA	MA	MA	MA	MA
Heraclides anchisiades	4	0.08	0.15	6.39	MA	MA	MA	MA	MA	MA	MA	MA
Heraclides androgeus	6	0.54	1.08	6.39	MA	MA	MA	MA	MA	SP (2)	SP (2)	MA
Heraclides astyalus	9	0.61	1.23	6.39	MA	MA	MA	MA	MA	MA	MA	MA
Heraclides hectorides	8	0.11	0.30	6.39	MA	MA	MA	MA	MA	MA	MA	MA
Heraclides thoas	11	0.33	0.62	7.52	MA	MA	MA	MA	MA	MA	MA	MA
Hermeuptychia gisella	4	0.00	0.00	2.64	MA	MA	MA	MA	MA	MA	MA	MA
Hermeuptychia isabella	22	0.17	0.46	2.64	MA	MA	MA	MA	MA	MA	MA	MA
Hylephila phyleus	14	0.43	0.81	5.52	MA	MA	MA	MA	MA	MA	MA	MA
Hypanartia lethe	8	0.00	0.00	6.85	MA	MA	MA	MA	MA	MA	MA	MA
Hypna clytemnestra	2	0.00	0.00	10.61	MA	MA	MA	MA	MA	MA	MA	MA
Hypothyris euclea	8	0.00	0.00	6.69	MA	MA	MA	MA	MA	MA	MA	MA
Ithomia agnosia	1	NA	NA	8.38	MA	MA	MA	MA	MA	MA	MA	MA
Itylos moza	1	NA	NA	7.85	MA	MA	MA	MA	MA	MA	MA	MA
Junonia genoveva	24	0.64	1.23	8.52	MA	MA	MA	MA	MA	MA	MA	MA
Kolana sp. 1	1	NA	NA	6.34	MA	MA	MA	MA	MA	MA	MA	MA
Lasaia agesilas	4	0.00	0.00	3.27	MA	MA	MA	MA	MA	MA	MA	MA
Lasaia arsis	3	0.00	0.00	3.27	MA	MA	MA	MA	MA	MA	MA	MA
Lento krexoides	1	NA	NA	9.03	MA	MA	MA	MA	MA	MA	MA	MA
Leptophobia aripa	8	0.09	0.15	9.40	MA	MA	MA	MA	MA	MA	MA	MA
Leptotes cassius	13	0.00	0.00	6.80	MA	MA	MA	MA	MA	MA	MA	MA
Lerodea eufala	4	0.20	0.30	6.85	MA	MA	MA	MA	MA	MA	MA	MA
Libytheana carinenta	9	0.07	0.31	7.95	MA	MA	MA	MA	MA	MA	MA	MA
Ludens silvaticus	1	NA	NA	8.19	MA	MA	MA	MA	MA	MA	MA	MA
Lycas argentea	4	1.66	3.28	7.52	SP (2)	SP (2)	SP (2)	SP (2)	SP (2)	SP (2)	SP (2)	SP (2)
Lychnuchoides ozias	1	NA	NA	8.46	MA	MA	MA	MA	MA	MA	MA	MA
Magnastigma hirsuta	1	NA	NA	7.17	MA	MA	MA	MA	MA	MA	MA	MA
Magneuptychia lea	2	0.00	0.00	10.62	MA	MA	MA	MA	MA	MA	MA	MA

Magneuptychia pallema	1	NA	NA	8.73	MA	MA	MA	MA	MA	MA	MA	MA
Manataria hercyna	5	0.09	0.15	9.73	MA	MA	MA	MA	MA	MA	MA	MA
Marpesia chiron	6	0.18	0.46	8.01	MA	MA	MA	MA	MA	MA	MA	MA
Marpesia petreus	7	0.60	0.92	8.36	MA	MA	MA	MA	MA	MA	MA	MA
Mcclungia cymo	1	NA	NA	7.38	MA	MA	MA	MA	MA	MA	MA	MA
Mechanitis lysimnia	10	0.51	1.54	7.34	MA	MA	MA	MA	MA	MA	MA	MA
Melanis aegates	1	NA	NA	2.96	MA	MA	MA	MA	MA	MA	MA	MA
Melanis hillapana	1	NA	NA	2.96	MA	MA	MA	MA	MA	MA	MA	MA
Melanis xenia	1	NA	NA	3.43	MA	MA	MA	MA	MA	MA	MA	MA
Memphis acidalia	1	NA	NA	4.76	MA	MA	MA	MA	MA	MA	MA	MA
Memphis moruus	8	0.00	0.00	4.76	MA	MA	MA	MA	MA	MA	MA	MA
Mesene celetes	1	NA	NA	6.18	MA	MA	MA	MA	MA	MA	MA	MA
Mesosemia odice	2	1.86	1.86	12.36	MA	MA	SP (2)	MA	MA	MA	MA	MA
Methionopsis ina	2	0.00	0.00	6.68	MA	MA	MA	MA	MA	MA	MA	MA
Methona themisto	4	0.00	0.00	9.96	MA	MA	MA	MA	MA	MA	MA	MA
Metron oropa	1	NA	NA	8.03	MA	MA	MA	MA	MA	MA	MA	MA
Milanion leucaspis	3	0.21	0.32	10.08	MA	MA	MA	MA	MA	MA	MA	MA
Mimoides lysithous	2	1.07	1.07	4.39	MA	MA	MA	MA	MA	MA	MA	MA
Mimoides microdamas	2	0.30	0.30	4.39	MA	MA	MA	MA	MA	MA	MA	MA
Ministrymon azia	4	0.92	1.85	3.10	MA	MA	MA	MA	MA	MA	MA	SP (2)
Ministrymon cruenta	2	0.16	0.16	1.23	ME	ME	ME	ME	ME	ME	ME	MA
Ministrymon gamma	1	NA	NA	1.23	ME	ME	ME	ME	ME	ME	ME	MA
Ministrymon una	1	NA	NA	4.22	MA	MA	MA	MA	MA	MA	MA	MA
Mithras hannelore	1	NA	NA	6.68	MA	MA	MA	MA	MA	MA	MA	MA
Mnasilus allubita	4	0.53	0.81	7.01	MA	MA	MA	MA	MA	MA	MA	MA
Moneuptychia griseldis	3	0.00	0.00	6.72	MA	MA	MA	MA	MA	MA	MA	MA
Moneuptychia paeon	1	NA	NA	7.56	MA	MA	MA	MA	MA	MA	MA	MA
Moneuptychia soter	3	0.10	0.15	6.71	MA	MA	MA	MA	MA	MA	MA	MA
Morpho epistrophus	10	0.27	0.61	7.53	MA	MA	MA	MA	MA	MA	MA	MA
Morpho helenor	12	0.27	0.67	7.53	MA	MA	MA	MA	MA	MA	MA	MA
Morys geisa	3	1.87	2.81	7.18	SP (2)	SP (2)	SP (2)	SP (2)	SP (2)	SP (2)	SP (2)	SP (2)
Mylon maimon	10	0.00	0.00	7.84	MA	MA	MA	MA	MA	MA	MA	MA
Myscelia orsis	4	0.23	0.46	7.35	MA	MA	MA	MA	MA	MA	MA	MA

Myscelus amystis	2	0.15	0.15	9.85	MA	MA	MA	MA	MA	MA	MA	MA
Nascus phocus	2	0.00	0.00	8.53	MA	MA	MA	MA	MA	MA	MA	MA
Nastra ethologus	2	0.00	0.00	7.02	MA	MA	MA	MA	MA	MA	MA	MA
Nesiostrymon calchinia	1	NA	NA	5.03	MA	MA	MA	MA	MA	MA	MA	MA
Nica flavilla	5	0.15	0.31	8.61	MA	MA	MA	MA	MA	MA	MA	MA
Nisoniades bipuncta	3	0.00	0.00	6.19	MA	MA	MA	MA	MA	MA	MA	MA
Nisoniades macarius	4	0.00	0.00	6.19	MA	MA	MA	MA	MA	MA	MA	MA
Notheme erota	6	0.31	0.92	6.67	MA	MA	MA	MA	MA	MA	SP (2)	MA
Nyctelius nyctelius	1	NA	NA	5.37	MA	MA	MA	MA	MA	MA	MA	MA
Opoptera aorsa	3	0.20	0.30	9.73	MA	MA	MA	MA	MA	MA	MA	MA
Opsiphanes invirae	8	0.04	0.15	9.58	MA	MA	MA	MA	MA	MA	MA	MA
Orses cynisca	1	NA	NA	7.52	MA	MA	MA	MA	MA	MA	MA	MA
Ortilia dicoma	7	0.17	0.46	5.21	MA	MA	MA	MA	MA	MA	MA	MA
Ortilia ithra	20	0.44	1.71	7.23	MA	MA	MA	MA	MA	MA	SP (3)	MA
Ortilia orthia	5	0.18	0.30	3.89	MA	MA	MA	MA	MA	MA	MA	MA
Ortilia velica	9	0.29	0.61	3.89	MA	MA	MA	MA	MA	MA	MA	MA
Ouleus fridericus	3	0.00	0.00	6.86	MA	MA	MA	MA	MA	MA	MA	MA
Pampasatyrus gyrtone	1	NA	NA	7.72	MA	MA	MA	MA	MA	MA	MA	MA
Panoquina ocola	13	0.19	0.66	7.34	MA	MA	MA	MA	MA	MA	MA	MA
Paracarystus hypargira	1	NA	NA	7.68	MA	MA	MA	MA	MA	MA	MA	MA
Parcella amarynthina	4	0.23	0.30	6.18	MA	MA	MA	MA	MA	MA	MA	MA
Pareuptychia summandosa	16	0.06	0.30	7.68	MA	MA	MA	MA	MA	MA	MA	MA
Parides anchises	2	0.00	0.00	9.23	MA	MA	MA	MA	MA	MA	MA	MA
Parides neophilus	4	0.77	1.54	9.23	MA	MA	MA	MA	MA	MA	MA	MA
Parphorus decora	1	NA	NA	8.71	MA	MA	MA	MA	MA	MA	MA	MA
Parrhasius polibetes	1	NA	NA	4.39	MA	MA	MA	MA	MA	MA	MA	MA
Paryphthimoides eous	17	0.34	1.08	5.73	MA	MA	MA	MA	MA	MA	MA	MA
Paryphthimoides phronius	5	0.06	0.15	5.73	MA	MA	MA	MA	MA	MA	MA	MA
Paryphthimoides poltys	5	0.12	0.30	7.68	MA	MA	MA	MA	MA	MA	MA	MA
Paulogramma pygas	8	0.54	1.25	8.71	SP (2)	MA	MA	MA	MA	SP (2)	SP (2)	SP (2)
Paulogramma pyracmon	6	0.00	0.00	8.71	MA	MA	MA	MA	MA	MA	MA	MA
Pellicia costimacula	3	0.20	0.30	5.54	MA	MA	MA	MA	MA	MA	MA	MA
Pellicia najoides	1	NA	NA	5.54	MA	MA	MA	MA	MA	MA	MA	MA

Perichares lotus	2	1.39	1.39	5.73	MA	MA	MA	MA	MA	MA	MA	MA
Perichares philetes	1	NA	NA	5.73	MA	MA	MA	MA	MA	MA	MA	MA
Pharneuptychia phares	7	0.31	1.08	6.71	MA	MA	MA	MA	MA	MA	SP (2)	MA
Phemiades pohli	1	NA	NA	8.69	MA	MA	MA	MA	MA	MA	MA	MA
Pheraeus perpulcher	1	NA	NA	7.85	MA	MA	MA	MA	MA	MA	MA	MA
Phocides charon	3	0.10	0.15	7.53	MA	MA	MA	MA	MA	MA	MA	MA
Phocides polybius	3	0.20	0.30	7.53	MA	MA	MA	MA	MA	MA	MA	MA
Phoebis argante	10	0.83	2.04	2.49	SP (2)	ME	MA	MA	MA	MA	MA	SP (2)
Phoebis neocypris	15	0.14	0.81	2.49	MA	ME	MA	MA	MA	MA	MA	MA
Phoebis sennae	14	0.09	0.61	5.35	MA	MA	MA	MA	MA	MA	MA	MA
Phystis simois	2	1.08	1.08	8.52	MA	MA	MA	MA	MA	MA	MA	MA
Polites vibex	12	0.43	1.39	6.02	MA	MA	MA	MA	MA	SP (2)	SP (2)	MA
Polythrix caunus	1	NA	NA	6.02	MA	MA	MA	MA	MA	MA	MA	MA
Polythrix octomaculata	3	0.00	0.00	6.02	MA	MA	MA	MA	MA	MA	MA	MA
Pompeius amblyspila	4	0.08	0.18	5.36	MA	MA	MA	MA	MA	MA	MA	MA
Pompeius dares	2	0.00	0.00	6.77	MA	MA	MA	MA	MA	MA	MA	MA
Pompeius pompeius	2	0.00	0.00	5.36	MA	MA	MA	MA	MA	MA	MA	MA
Praepedaliodes phanias	7	0.09	0.32	12.40	MA	MA	MA	MA	MA	MA	MA	MA
Propertius propertius	2	0.00	0.00	7.00	MA	MA	MA	MA	MA	MA	MA	MA
Pseudodebis euptychidia	6	0.05	0.15	7.86	MA	MA	MA	MA	MA	MA	MA	MA
Pseudolycaena marsyas	4	0.25	0.31	5.64	MA	MA	MA	MA	MA	MA	MA	MA
Pseudopieris nehemia	8	0.08	0.30	10.59	MA	MA	MA	MA	MA	MA	MA	MA
Pseudoscada erruca	3	0.00	0.00	5.38	MA	MA	MA	MA	MA	MA	MA	MA
Pteronymia carlia	2	0.15	0.15	7.19	MA	MA	MA	MA	MA	MA	MA	MA
Pyrgus orcus	22	0.99	2.02	5.03	MA	MA	MA	MA	MA	MA	MA	MA
Pyrgus orcynoides	18	0.57	1.08	5.03	MA	MA	MA	MA	MA	MA	MA	MA
Pyrisitia leuce	13	0.84	3.05	10.25	MA	MA	SP (2)	SP (2)	SP (2)	SP (2)	SP (2)	MA
Pyrisitia nise	15	0.24	0.92	11.48	MA	MA	MA	MA	MA	MA	MA	MA
Pyrrhogyra neaerea	8	1.11	2.17	10.51	MA	MA	MA	MA	MA	MA	SP (2)	SP (2)
Pyrrhopygopsis socrates	2	0.00	0.00	9.73	MA	MA	MA	MA	MA	MA	MA	MA
Quadrus cerialis	1	NA	NA	6.86	MA	MA	MA	MA	MA	MA	MA	MA
Quinta cannae	5	0.09	0.15	8.35	MA	MA	MA	MA	MA	MA	MA	MA
Rekoa marius	1	NA	NA	5.85	MA	MA	MA	MA	MA	MA	MA	MA

Rekoa meton	1	NA	NA	6.51	MA	MA	MA	MA	MA	MA	MA	MA
Rekoa palegon	10	0.09	0.31	5.48	MA	MA	MA	MA	MA	MA	MA	MA
Remella remus	1	NA	NA	7.51	MA	MA	MA	MA	MA	MA	MA	MA
Rhabdodryas trite	4	0.26	0.46	5.35	MA	MA	MA	MA	MA	MA	MA	MA
Riodina lycisca	5	0.12	0.30	4.54	MA	MA	MA	MA	MA	MA	MA	MA
Riodina lysippoides	8	0.74	1.23	4.54	MA	MA	MA	MA	MA	MA	MA	SP (2)
Sarmientoia almeidae	1	NA	NA	10.77	MA	MA	MA	MA	MA	MA	MA	MA
Saturnus reticulata	1	NA	NA	8.87	MA	MA	MA	MA	MA	MA	MA	MA
Selenophanes cassiope	2	0.15	0.15	6.88	MA	MA	MA	MA	MA	MA	MA	MA
Siproeta epaphus	5	0.06	0.15	6.85	MA	MA	MA	MA	MA	MA	MA	MA
Siproeta stelenes	8	0.04	0.15	6.85	MA	MA	MA	MA	MA	MA	MA	MA
Smyrna blomfildia	8	0.38	1.26	9.17	MA	MA	MA	MA	MA	MA	MA	MA
Sodalia coler	11	0.19	0.61	7.67	MA	MA	MA	MA	MA	MA	MA	MA
Splendeuptychia hygina	5	0.09	0.15	8.52	MA	MA	MA	MA	MA	MA	MA	MA
Splendeuptychia libitina	3	0.00	0.00	9.21	MA	MA	MA	MA	MA	MA	MA	MA
Staphylus chlorocephala	1	NA	NA	9.56	MA	MA	MA	MA	MA	MA	MA	MA
Staphylus incisus	2	0.00	0.00	10.96	MA	MA	MA	MA	MA	MA	MA	MA
Staphylus melangon	14	0.04	0.30	6.52	MA	MA	MA	MA	MA	MA	MA	MA
Staphylus musculus	1	NA	NA	5.21	MA	MA	MA	MA	MA	MA	MA	MA
Staphylus tucumanus	2	0.00	0.00	6.52	MA	MA	MA	MA	MA	MA	MA	MA
Staphylus vulgata	2	0.15	0.15	5.21	MA	MA	MA	MA	MA	MA	MA	MA
Stegosatyrus periphas	11	0.50	1.86	7.36	MA	MA	MA	MA	MA	MA	MA	MA
Strephonota sphinx	1	NA	NA	4.26	MA	MA	MA	MA	MA	MA	MA	MA
Strephonota tephraeus	3	0.00	0.00	4.26	MA	MA	MA	MA	MA	MA	MA	MA
Strymon astiocha	2	0.00	0.00	5.20	MA	MA	MA	MA	MA	MA	MA	MA
Strymon bazochii	1	NA	NA	5.71	MA	MA	MA	MA	MA	MA	MA	MA
Strymon bubastus	1	NA	NA	4.15	MA	MA	MA	MA	MA	MA	MA	MA
Strymon cestri	1	NA	NA	5.01	MA	MA	MA	MA	MA	MA	MA	MA
Strymon eurytulus	9	0.26	0.98	4.15	MA	MA	MA	MA	MA	MA	SP (2)	MA
Strymon lucena	1	NA	NA	2.56	MA	ME	MA	MA	MA	MA	MA	MA
Strymon megarus	2	0.15	0.15	2.56	MA	ME	MA	MA	MA	MA	MA	MA
Strymon mulucha	2	0.46	0.46	5.38	MA	MA	MA	MA	MA	MA	MA	MA
Strymon rufofusca	3	0.41	0.61	5.38	MA	MA	MA	MA	MA	MA	MA	MA

Synapte malitiosa	1	NA	NA	7.87	MA	MA	MA	MA	MA	MA	MA	MA
Synapte silius	3	0.10	0.15	7.87	MA	MA	MA	MA	MA	MA	MA	MA
Synargis calyce	3	0.00	0.00	9.55	MA	MA	MA	MA	MA	MA	MA	MA
Synargis ochrophlegma	2	0.00	0.00	8.86	MA	MA	MA	MA	MA	MA	MA	MA
Tatochila autodice	5	0.00	0.00	9.04	MA	MA	MA	MA	MA	MA	MA	MA
Taygetis kerea	1	NA	NA	7.86	MA	MA	MA	MA	MA	MA	MA	MA
Taygetis rufomarginata	5	0.00	0.00	6.73	MA	MA	MA	MA	MA	MA	MA	MA
Taygetis tripunctata	5	0.06	0.15	6.73	MA	MA	MA	MA	MA	MA	MA	MA
Taygetis ypthima	2	0.61	0.61	8.54	MA	MA	MA	MA	MA	MA	MA	MA
Tegosa claudina	18	0.20	0.65	6.67	MA	MA	MA	MA	MA	MA	MA	MA
Tegosa orobia	1	NA	NA	6.67	MA	MA	MA	MA	MA	MA	MA	MA
Telemiades amphion	1	NA	NA	7.18	MA	MA	MA	MA	MA	MA	MA	MA
Telemiades laogonus	1	NA	NA	7.18	MA	MA	MA	MA	MA	MA	MA	MA
Temenis laothoe	8	0.53	1.71	8.21	MA	MA	MA	MA	MA	MA	MA	SP (2)
Theagenes dichrous	1	NA	NA	7.67	MA	MA	MA	MA	MA	MA	MA	MA
Thespieus aspernatus	5	0.00	0.00	7.87	MA	MA	MA	MA	MA	MA	MA	MA
Thespieus dalman	3	0.00	0.00	3.92	MA	MA	MA	MA	MA	MA	MA	MA
Thespieus ethemides	2	0.00	0.00	3.92	MA	MA	MA	MA	MA	MA	MA	MA
Thracides cleanthes	1	NA	NA	8.02	MA	MA	MA	MA	MA	MA	MA	MA
Thyridia psidii	2	0.00	0.00	8.87	MA	MA	MA	MA	MA	MA	MA	MA
Tisias lesueur	3	0.00	0.00	7.19	MA	MA	MA	MA	MA	MA	MA	MA
Tithorea harmonia	7	0.04	0.15	8.52	MA	MA	MA	MA	MA	MA	MA	MA
Trina geometrina	12	1.35	2.65	7.85	SP (2)	MA	SP (2)	MA	SP (2)	SP (2)	SP (2)	SP (2)
Typhedanus undulatus	2	0.15	0.15	5.85	MA	MA	MA	MA	MA	MA	MA	MA
Urbanus albimargo	1	NA	NA	9.07	MA	MA	MA	MA	MA	MA	MA	MA
Urbanus dorantes	6	0.09	0.19	6.09	MA	MA	MA	MA	MA	MA	MA	MA
Urbanus esta	2	0.00	0.00	6.68	MA	MA	MA	MA	MA	MA	MA	MA
Urbanus procne	10	0.06	0.30	5.00	MA	MA	MA	MA	MA	MA	MA	MA
Urbanus pronta	1	NA	NA	6.68	MA	MA	MA	MA	MA	MA	MA	MA
Urbanus simplicius	4	0.08	0.15	6.18	MA	MA	MA	MA	MA	MA	MA	MA
Urbanus teleus	2	0.00	0.00	5.00	MA	MA	MA	MA	MA	MA	MA	MA
Urbanus zagorus	3	0.00	0.00	6.18	MA	MA	MA	MA	MA	MA	MA	MA
Vacerra caniola	2	0.00	0.00	6.67	MA	MA	MA	MA	MA	MA	MA	MA

Vanessa braziliensis	9	0.71	2.80	6.18	SP (2)	MA	SP (2)	SP (2)	SP (2)	SP (2)	SP (2)	SP (2)
Vanessa carye	5	0.00	0.00	6.18	MA	MA	MA	MA	MA	MA	MA	MA
Vehilius inca	1	NA	NA	7.36	MA	MA	MA	MA	MA	MA	MA	MA
Vehilius stictomenes	3	0.00	0.00	8.03	MA	MA	MA	MA	MA	MA	MA	MA
Vettius arva	1	NA	NA	7.85	MA	MA	MA	MA	MA	MA	MA	MA
Vettius lucretius	1	NA	NA	8.02	MA	MA	MA	MA	MA	MA	MA	MA
Vettius marcus	1	NA	NA	7.85	MA	MA	MA	MA	MA	MA	MA	MA
Vinius pulcherrimus	3	0.00	0.00	7.34	MA	MA	MA	MA	MA	MA	MA	MA
Vinius tryhana	2	0.00	0.00	8.02	MA	MA	MA	MA	MA	MA	MA	MA
Viola minor	5	0.09	0.15	8.01	MA	MA	MA	MA	MA	MA	MA	MA
Virga austrinus	1	NA	NA	6.18	MA	MA	MA	MA	MA	MA	MA	MA
Virga silvanus	2	0.00	0.00	9.05	MA	MA	MA	MA	MA	MA	MA	MA
Virga sp.	3	0.00	0.00	6.18	MA	MA	MA	MA	MA	MA	MA	MA
Wallengrenia premnas	7	0.26	0.46	3.45	MA	MA	MA	MA	MA	MA	MA	MA
Wallengrenia sapuca	4	0.00	0.00	3.45	MA	MA	MA	MA	MA	MA	MA	MA
Xeniades orchamus	1	NA	NA	6.52	MA	MA	MA	MA	MA	MA	MA	MA
Xenophanes tryxus	11	0.18	0.46	7.18	MA	MA	MA	MA	MA	MA	MA	MA
Yphthimoides affinis	5	0.18	0.46	5.89	MA	MA	MA	MA	MA	MA	MA	MA
Yphthimoides celmis	21	0.10	0.46	6.62	MA	MA	MA	MA	MA	MA	MA	MA
Yphthimoides mimula	6	0.05	0.15	5.89	MA	MA	MA	MA	MA	MA	MA	MA
Yphthimoides ordinaria	6	0.00	0.00	6.62	MA	MA	MA	MA	MA	MA	MA	MA
Zabuella tenellus	8	0.08	0.33	8.69	MA	MA	MA	MA	MA	MA	MA	MA
Zaretis strigosus	4	3.75	5.71	9.38	SP (2)	SP (2)	SP (2)	SP (2)	SP (2)	SP (2)	SP (2)	SP (2)
Zenis jebus	2	0.00	0.00	7.84	MA	MA	MA	MA	MA	MA	MA	MA
Zera hyacinthinus	1	NA	NA	4.72	MA	MA	MA	MA	MA	MA	MA	MA
Zera tetrastigma	1	NA	NA	4.72	MA	MA	MA	MA	MA	MA	MA	MA
Ziegleria hesperitis	1	NA	NA	6.68	MA	MA	MA	MA	MA	MA	MA	MA
Zizula cyna	7	0.44	1.07	8.86	MA	MA	MA	MA	MA	MA	MA	MA
Zopyrion evenor	5	0.06	0.15	7.68	MA	MA	MA	MA	MA	MA	MA	MA

H. feronia has no BIN assignment because it has more than 1% of ambiguous calls.

Table D. Results of the TCS analyses for a range of ten cut-off (i.e. parsimony limit) values.

Parsimony limit	Maximum connection steps	Subnetworks
90%	16	416
91%	15	420
92%	14	422
93%	13	424
94%	12	427
95%	11	429
96%	9	434
97%	8	441
98%	6	460
99%	4	479

Table E. Detailed results of the ABGD analyses for two distance models (p-distance and K2P), two relative gap widths (X = 1 and X = 1.5) and a range of ten prior intraspecific divergence values (P) between 0.001 (0.1%) and 0.0359 (3.59%). All species formed a single group when P = 3.59 regardless of the settings.

Distance model	X	Partition	Prior intraspecific divergence (P, %)							
			0.1	0.17	0.28	0.46	0.77	1.29	2.15	3.59
p-distance	1.5	Initial	997	549	424	466	424	424	424	1
		Recursive	997	570	494	471	436	430	424	1
	1	Initial	997	549	549	466	424	424	424	1
		Recursive	997	570	570	472	436	430	424	1
K2P	1.5	Initial	997	549	549	424	424	424	424	1
		Recursive	997	573	573	444	436	430	424	1
	1	Initial	997	549	549	424	424	424	424	1
		Recursive	997	575	575	446	437	431	424	1

Table F. Results of the Mantel tests performed on 42 species separately. For each species we inform the number of sequences (N), the maximum geographic and genetic distances registered, the correlation coefficient (*r*), the coefficient of determination (r^2), and the significance of the test (*p*).

Species	N	Max. Distance (km)	Max. Distance (K2P, %)	*r*	r^2	*p*
Actinote pellenea	27	1200.60	0.92	0.143	0.021	0.033
Agraulis vanillae	18	1128.28	0.96	0.070	0.005	0.190
Anartia amathea	18	793.47	0.76	0.037	0.001	0.235
Anartia jatrophae	13	275.46	0.46	-0.113	0.013	0.006
Anthanassa frisia	12	494.81	0.77	-0.102	0.010	0.390
Ascia monuste	12	725.73	0.32	-0.087	0.008	0.388
Chlosyne lacinia	10	407.25	0.30	0.130	0.017	0.446
Danaus eresimus	12	793.47	0.76	-0.183	0.033	0.131
Danaus erippus	12	1085.21	0.76	0.123	0.015	0.203
Diaethria clymena	11	407.25	0.46	-0.305	0.093	0.130
Dircenna dero	10	407.25	0.30	-0.209	0.044	0.617
Dryas iulia	12	494.81	1.39	0.008	0.000	0.459
Eantis thraso	11	494.81	3.63	0.826	0.682	0.007
Eurema deva	18	1280.00	0.31	0.187	0.035	0.110
Eurema elathea	11	797.38	7.24	-0.027	0.001	0.440
Heliconius erato	16	793.47	3.45	-0.142	0.020	0.076
Heliopetes arsalte	10	797.38	0.77	0.085	0.007	0.348
Heliopetes omrina	20	1128.28	1.80	0.318	0.101	0.021
Heraclides thoas	11	1041.57	0.62	0.151	0.023	0.274
Hermeuptychia isabella	22	1116.85	0.46	0.583	0.340	0.001
Hylephila phyleus	14	1059.66	0.81	0.021	0.000	0.401
Junonia genoveva	24	1177.91	1.23	-0.051	0.003	0.150
Leptotes cassius	13	797.38	0.00	NA	NA	NA
Morpho epistrophus	10	371.09	0.61	0.847	0.718	0.004
Morpho helenor	12	480.74	0.67	0.427	0.183	0.006
Ortilia ithra	20	1168.45	1.71	-0.055	0.003	0.230
Panoquina ocola	13	765.16	0.66	-0.082	0.007	0.494

Paryphthimoides eous	17	494.81	1.08	0.050	0.003	0.332
Phoebis neocypris	15	480.74	0.81	0.189	0.036	0.144
Phoebis sennae	14	797.38	0.61	-0.073	0.005	0.404
Polites vibex	12	1007.40	1.39	0.035	0.001	0.304
Pyrgus orcus	22	1166.64	2.02	-0.065	0.004	0.230
Pyrgus orcynoides	18	1166.64	1.08	0.270	0.073	0.009
Pyrisitia leuce	13	471.41	3.05	0.480	0.231	0.024
Pyrisitia nise	15	764.16	0.92	0.141	0.020	0.066
Rekoa palegon	10	494.81	0.31	-0.022	0.001	0.432
Staphylus melangon	14	494.81	0.30	-0.119	0.014	0.310
Stegosatyrus periphas	11	878.83	1.86	0.515	0.265	0.062
Tegosa claudina	18	1116.85	0.65	-0.155	0.024	0.044
Trina geometrina	12	407.25	2.65	0.237	0.056	0.026
Xenophanes tryxus	11	494.81	0.46	0.394	0.155	0.040
Yphthimoides celmis	21	1116.85	0.46	0.336	0.113	0.002
