# Supplementary material for: Barcoding the butterflies of southern South America: Species delimitation efficacy, cryptic diversity and geographic patterns of divergence
Source: PLoS One. 2017 Oct 19;12(10):e0186845. doi: 10.1371/journal.pone.0186845 (PMC5648246; doi:10.1371/journal.pone.0186845)
Supplement: S2 Supporting Information — Results from the “threshVal” and “localMinima” functions implemented in SPIDER. These functions were used to compute two of the four thresholds employed for the BCM and BIC criteria. (PDF) [file pone.0186845.s004.pdf]

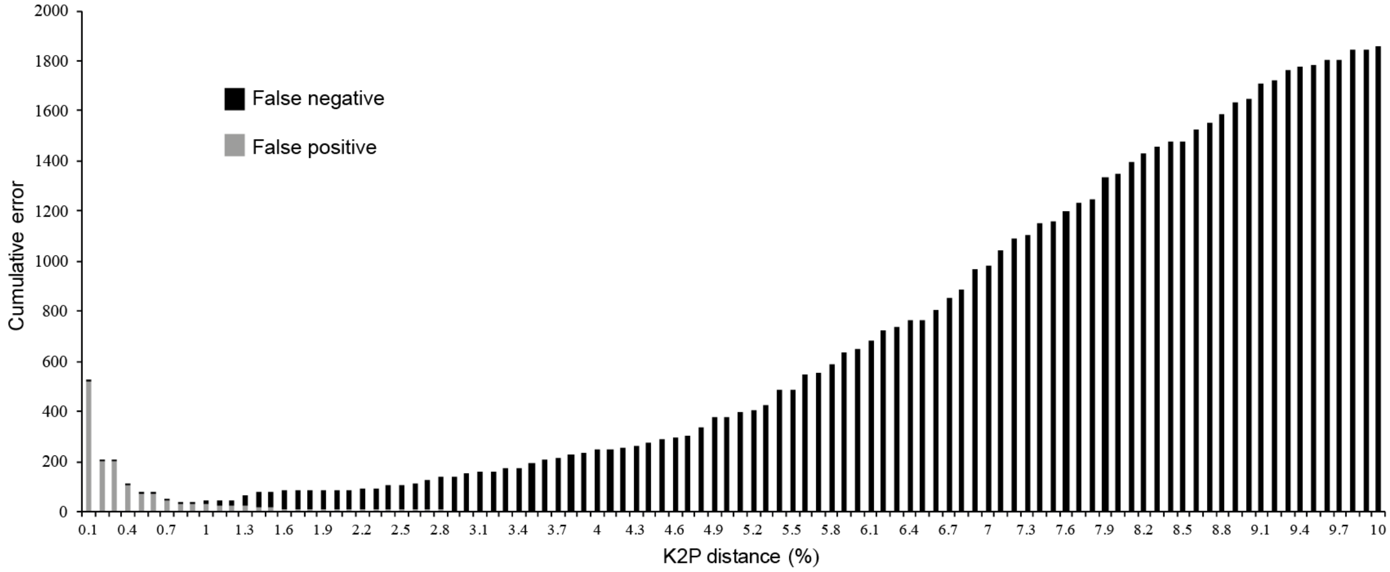


**Fig A. Frequency histogram of false-positive and false-negative identifications (cumulative error) across threshold values from 0.1% to 10% generated with the function "threshVal" in SPIDER.**

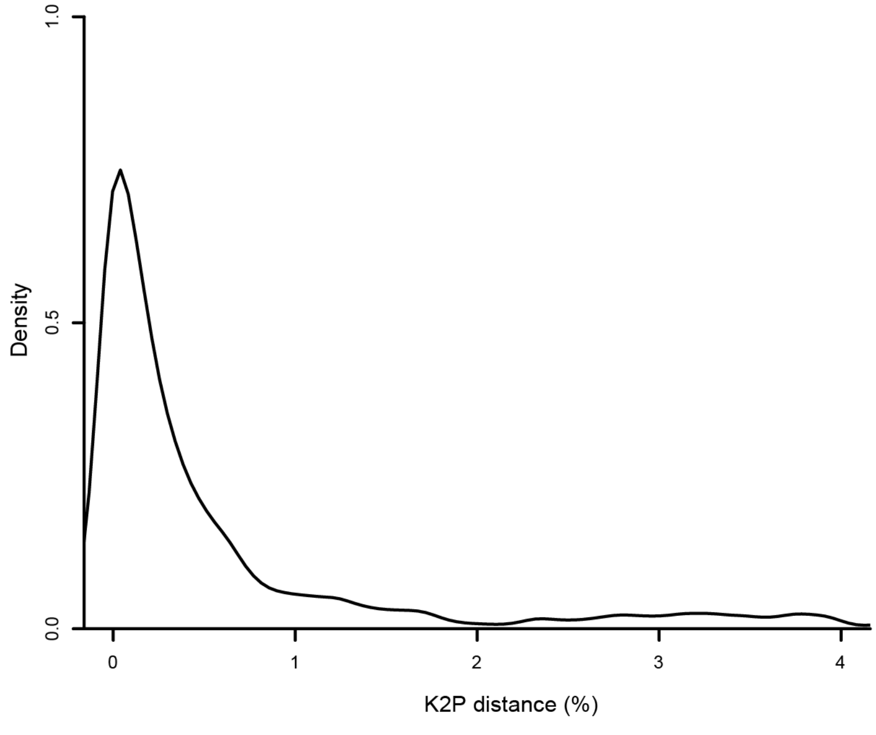


**Fig B. Density plot of all genetic distances obtained with the function 'localMinima' in SPIDER.**
