# Supplementary material for: Barcoding the butterflies of southern South America: Species delimitation efficacy, cryptic diversity and geographic patterns of divergence
Source: PLoS One. 2017 Oct 19;12(10):e0186845. doi: 10.1371/journal.pone.0186845 (PMC5648246; doi:10.1371/journal.pone.0186845)
Supplement: S1 Appendix — Details on how ABGD, TCS and RESL partition the alignment of DNA barcodes into Molecular Operational Taxonomic Units (MOTUs). (PDF) [file pone.0186845.s005.pdf]

Clustering algorithms implemented in our analysis of the butterflies of southern South America.

To assess the presence of cryptic diversity we implemented three different clustering algorithms: Automatic Barcode Gap Discovery [1], statistical parsimony networks [2] as implemented in TCS [3], and the Refined Single Linkage algorithm (RESL, [4]).

ABGD is a statistical method that explores the distribution of all pairwise distances looking for the gap between intra- and interspecific distances. This method detects peaks of slope values in a local function estimated from the ranked pairwise distances which correspond to gaps in the original distribution. After the gap is inferred, the data set is partitioned into groups (i.e. MOTUs) so that distances between sequences in those groups are always larger than the gap distance, and that for each sequence within each group there is at least another sequence in the same group with a distance smaller than the gap distance. This is called the initial partition. The same process is then recursively applied to the groups in the initial partition until no further splitting occur, creating the recursive partition (see [1] for more details on the procedure). We run ABGD on command line and using K2P and uncorrected pairwise distance matrices as inputs and testing two relative gap width values (X = 1.5, 1.0). We recorded all partitioning schemes for a range of prior intraspecific divergence (P) values between 0.001 (0.1%) and 0.1 (10%).

TCS applies the statistical parsimony method to construct haplotype networks. It has been shown that the count of Linnaean species present in a COI alignment greatly matches that of independent statistical parsimony networks inferred by the software [5]. TCS first estimates the maximum number of differences between two haplotypes as a result of single substitutions (i.e. those that are not the result of multiple hits) based on the probability of parsimony defined by the user. The haplotypes are then connected in a network until the substitutions between them exceed the maximum number established by the parsimony limit. When this limit is exceeded, the haplotypes end in unconnected, independent networks. In short, the higher the cut-off value (i.e. parsimony limit), the lower the number of differences that are allowed between haplotypes and the higher the count of unconnected networks. We registered the MOTU count for ten different cut-off values (90%-99%).

RESL is the algorithm on which the Barcode Index Number (BIN) system is based on. This system groups the COI barcode sequences uploaded to BOLD into genetic clusters (i.e. the BINs) based on sequence variation [4]. RESL first delimits initial MOTUs based on single linkage clustering with a 2.2% threshold of maximum divergence allowed within a cluster. Then these MOTUs are revisited with Markov Clustering, a graph analytical approach that uses haplotype similarity and connectivity information to reassess the clusters boundaries. Finally, the partitioning scheme that maximizes the Silhouette score is chosen via the Silhouette Criterion (see [4] for a more detailed explanation). Since BINs on BOLD are delineated based on all COI sequences uploaded to the platform by the users, they are not strictly comparable with the MOTUs delineated with the other algorithms. Therefore, we applied the RESL algorithm exclusively to our data set using the Cluster Sequences analysis tool available on BOLD v4 (http://www.v4.boldsystems.org). However, to assess how the addition of other sequences can modify the outcome of the RESL algorithm, we

did compare the count of MOTUs and their composition between standard BIN assignments available on BOLD and the clusters generated with our data only.

References

1. Puillandre N, Lambert A, Brouillet S, Achaz G. ABGD, Automatic Barcode Gap Discovery for primary species delimitation. Mol Ecol. Blackwell Publishing Ltd; 2012;21: 1864–1877. doi:10.1111/j.1365-294X.2011.05239.x
2. Templeton AR, Crandall KA, Sing CF. A cladistic analysis of phenotypic associations with haplotypes inferred from restriction endonuclease mapping and DNA sequence data. III. Cladogram estimation. Genetics. 1992;132: 619–33. Available: http://www.ncbi.nlm.nih.gov/pubmed/1385266
3. Clement M, Posada D, Crandall KA. TCS: a computer program to estimate gene genealogies. Mol Ecol. 2000;9: 1657–1659. doi:10.1046/j.1365-294x.2000.01020.x
4. Ratnasingham S, Hebert PDN. A DNA-based registry for all animal species: The Barcode Index Number (BIN) System. Fontaneto D, editor. PLoS One. Public Library of Science; 2013;8: e66213. doi:10.1371/journal.pone.0066213
5. Hart MW, Sunday J. Things fall apart: biological species form unconnected parsimony networks. Biol Lett. The Royal Society; 2007;3: 509–12. doi:10.1098/rsbl.2007.0307
