# Supplementary material for: Barcoding the butterflies of southern South America: Species delimitation efficacy, cryptic diversity and geographic patterns of divergence
Source: PLoS One. 2017 Oct 19;12(10):e0186845. doi: 10.1371/journal.pone.0186845 (PMC5648246; doi:10.1371/journal.pone.0186845)
Supplement: S2 Appendix — Details on three new records of butterflies for Argentina generated in the context of the field work carried out for this project. (PDF) [file pone.0186845.s006.pdf]

Three new records for Argentina generated in the context of the field work carried out to assemble the DNA barcode reference library of the butterflies of Argentina.

Pieridae

Coliadinae

Eurema agave pallida (Chavannes, 1850)

Geographic distribution: South of Brazil (Warren *et al*., 2016), in open and humid environments of the eastern Chaco district and the Atlantic forest.
Material examined: Formosa: Departamento Pilcomayo, Parque Nacional Río Pilcomayo, Seccional Estero Poí, E. Núñez Bustos & C. Kopuchian col., 28-29-may-2011, 3 ♂♂ [MACN-Bar-Lep 02615, 02630, 02685].

Comments: It closely resembles the highly variable females of *Eurema elathea flavescens* (Chavannes, 1850) and *E. phiale paula* (Röber, 1909) but is bigger and the black spot of forewings more narrow and sinuous. Possibly it is also in the provinces of Chaco, Corrientes and Misiones. It has been cited for northern Paraguay (Torres & Kochalka, 1993). ENB has seen a specimen of the area of Villarica and looks like to this species.

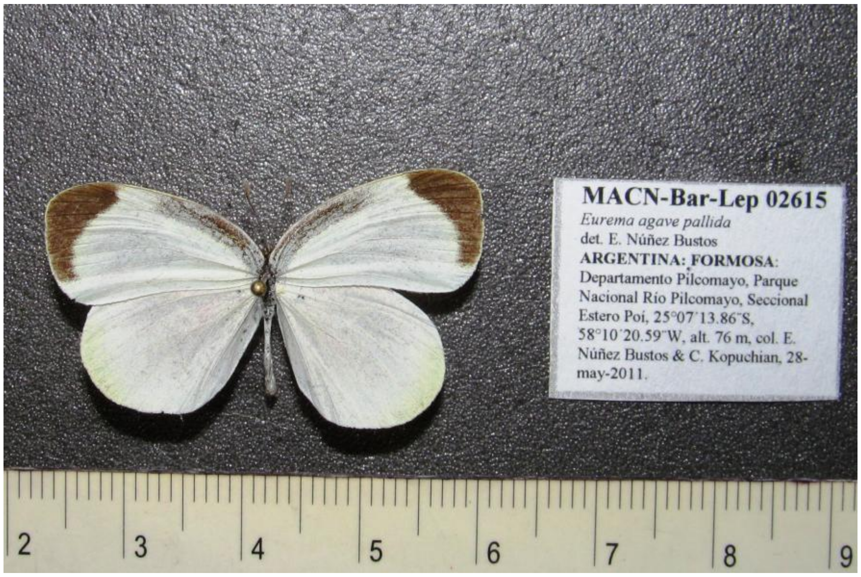

***Eurema agave pallida* (dorsal view)**

Lycaenidae

Theclinae

Mithras hannelore Bálint & Moser, 2001

Geographic distribution: South of Brazil (Santa Catarina and Rio Grande do Sul states) (Bálint & Moser, 2001; Bálint, 2004), in Atlantic forests.

Material examined: Misiones: Departamento Iguazú, Parque Nacional Iguazú, Seccional Yacu-í, E. Núñez Bustos col., 19-dic-2010, 1 ♂ [MACN-Bar-Lep 01328].

Comments: Lamas (2004) suggested that *M. hannelore* and *M. colombiensis* are synonym species. The latter is found in from Colombia to Peru (Johnson & Constantino, 1997; Warren *et al*., 2016). However, given the morphological and geographic differences observed between these two species we considered *M. hannelore* a separate entity. The Argentinian specimen would be the first one that comes from the interior of the Atlantic forest instead of the coastal region, like the exemplary types (Bálint & Moser, 2001). The specimen collected was found in low plants of the shady underbrush. It is very similar to *M. orobia* (Hewitson, 1867), already mentioned by Hayward (1973) for Misiones and by Núñez Bustos (2008, 2009) for Yacutinga and Iguazú reserves.

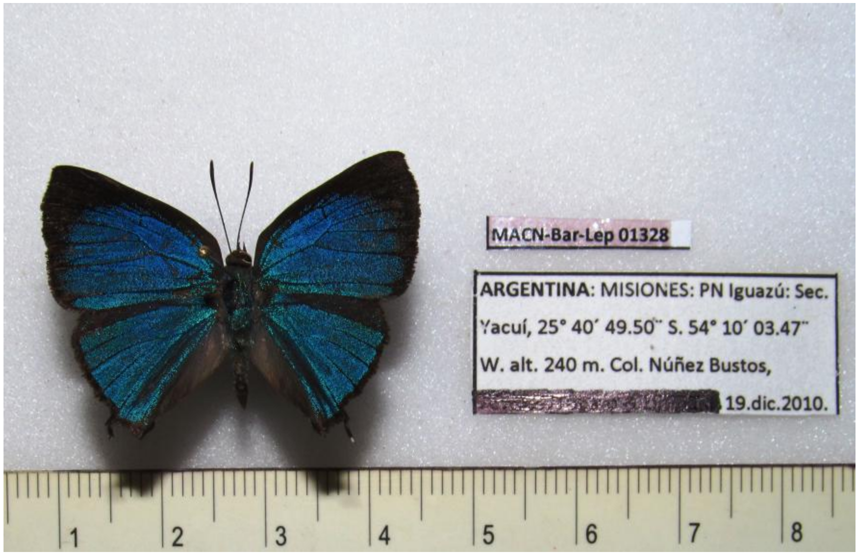

***Mithras hannelore* (dorsal view)**

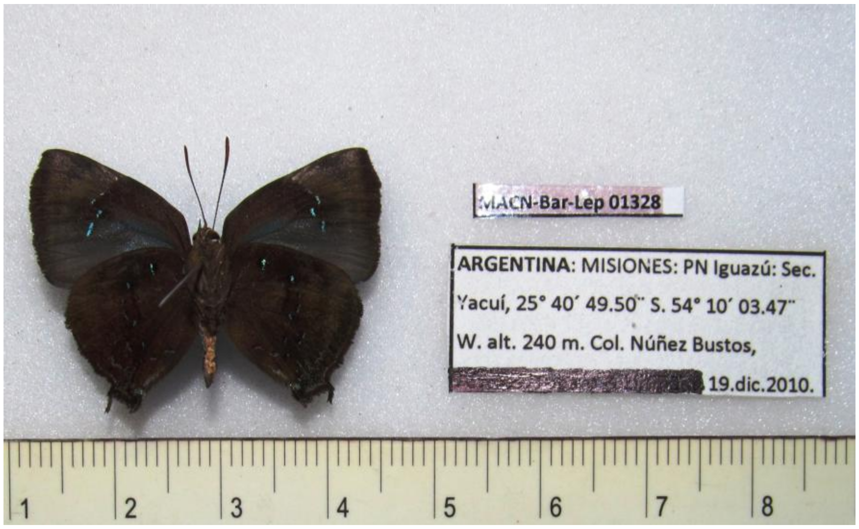

***Mithras hannelore* (ventral view)**

Riodinidae

Riodininae

Melanis hillapana cratippa (Seitz, 1913)

Geographic distritution: Known from northeastern and central Brazil (Warren *et al*., 2016). Found in warm and very humid environments.

Material examined: Formosa: Departamento Pilcomayo, Parque Nacional Río Pilcomayo, Parador Yaguareté, E. Núñez Bustos & C. Kopuchian col., 30-may-2011, 1 ♂ [MACN-Bar-Lep 02736].

Comments: Very similar to *M. aegates cretiplaga* (Stichel, 1910), which is the typical *Melanis* of humid chaco in Argentina. In Paraguay it was mentioned as "*Lymnas hillapana*" for Asunción (Torres & Kochalka, 1993).

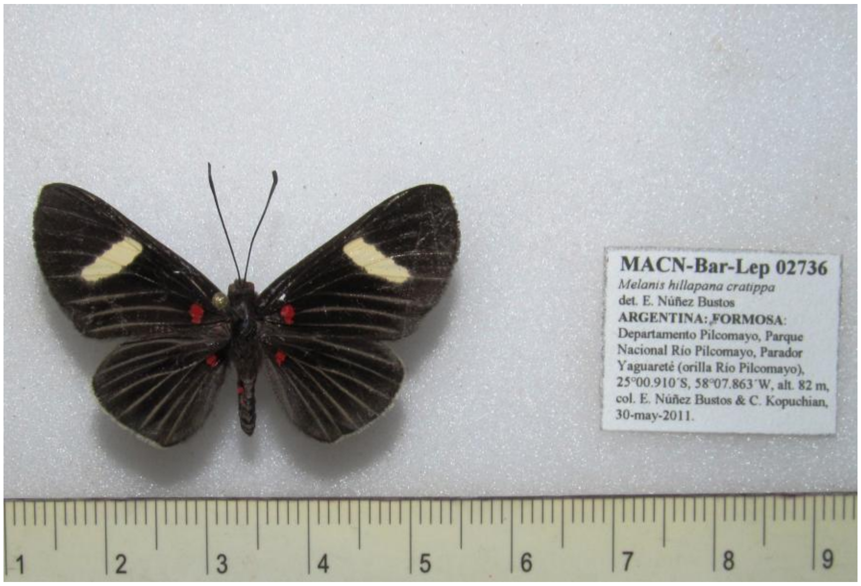

***Melanis hillapana cratippa* (dorsal view)**

References

Bálint, Z. 2004. Further notes on the neotropical hairstreak butterfly genus *Paraspiculatus* (Lepidoptera: Lycaenidae, Eumaeini). *Folia entomologica hungarica* 65: 107-116.

Bálint, Z., & A. Moser. 2001. Notes on the genus *Paraspiculatus* (Insecta: Lepidoptera: Lycaenidae: Eumaeini) with a synopsis of the taxa occurring in southern Brazil. *Annalen der naturhistorischen Museum Wien* 103(B): 249-262.

Hayward, K. J. 1973. Catálogo de los ropalóceros argentinos. *Opera Lilloana* 23: 1-1318.

Lamas, G. (ed.) 2004. Checklist: Part 4 A Hesperioidea - Papilionoidea. In: J. B. Heppner. *Atlas of Neotropical Lepidoptera*. Association for Tropical Lepidoptera. Gainesville.

Nuñez Bustos, E. 2008. Diversidad de mariposas diurnas en la Reserva Privada Yacutinga, Provincia de Misiones, Argentina (Lepidoptera: Hesperioidea y Papilionoidea). Tropical Lepidoptera Research 18(2): 92-101.

Nuñez Bustos, E. 2009. Mariposas diurnas (Lepidoptera: Papilionoidea y Hesperioidea) del Parque Nacional Iguazú, Provincia de Misiones, Argentina. Tropical Lepidoptera Research 19(2): 71-81.

Torres, D. & J. A. Kochalka. 1993. Lista de las mariposas diurnas (Hesperioidea y Papilionoidea) del Paraguay y regiones limítrofes, con datos sobre su distribución en Paraguay. DPNVS, WWF & IBNP-Museo. Biodiversity Support Program. 143 pp.

Warren, A. D., Davis, K. J., Stangeland, E. M., Pelham, J. P., Willmott, K. R., & Grishin, N. V. 2016. Ilustrated Lists of American Butterflies [15-IX-2016]. Available at http://www.butterfliesofamerica.com
