# Supplementary material for: Barcoding the butterflies of southern South America: Species delimitation efficacy, cryptic diversity and geographic patterns of divergence
Source: PLoS One. 2017 Oct 19;12(10):e0186845. doi: 10.1371/journal.pone.0186845 (PMC5648246; doi:10.1371/journal.pone.0186845)
Supplement: S3 Appendix — We provide pictures of some specimens involved in the taxonomic cases discussed in more depth in the Discussion section: A. fulgerator, P. argante, G. muscosa, Emesis russula/E. mandana and Calycopis sp. 1/Calycopos sp. 2/ C. caulonia. (PDF) [file pone.0186845.s007.pdf]

Photos of the specimens associated with the taxonomic cases discussed in more detail.

We provide pictures of some specimens involved in the taxonomic cases discussed in more depth in the Discussion section: *A. fulgerator*, *P. argante, G. muscosa*, *Emesis russula/E. mandana* and *Calycopis sp. 1*/*Calycopos sp.* 2/ *C. caulonia*. One specimen per barcode cluster is represented. The photos show the dorsal and ventral views of each individual together with its voucher code (Sample ID in Table A in S1 Supporting Information). We also specify the Process ID of each individual so the reader can find these specimens in S1 and S2 Figs and in Fig 6. In some cases we included multiple specimens from different taxa in the same picture to facilitate the comparison. Lastly, photos of all the specimens analyzed in our manuscript, together with their corresponding metadata, are available in the public data set "DS-BUNEACAR" (dx.doi.org/10.5883/DS-BUNEACAR) on BOLD (www.boldsystems.org).

Astraptes fulgerator

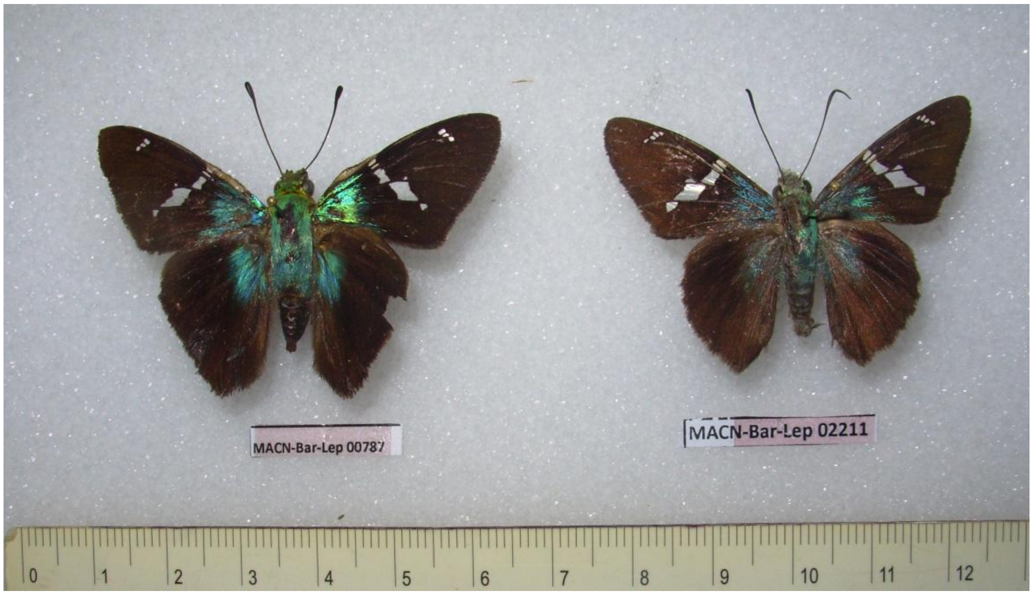

Dorsal view of two specimens of *A. fulgerator* representatives of the two COI lineages found within this species (see Fig 6 in the main text). Left: LEPAR569-11 (male). Right: LEPIG300-11 (female).

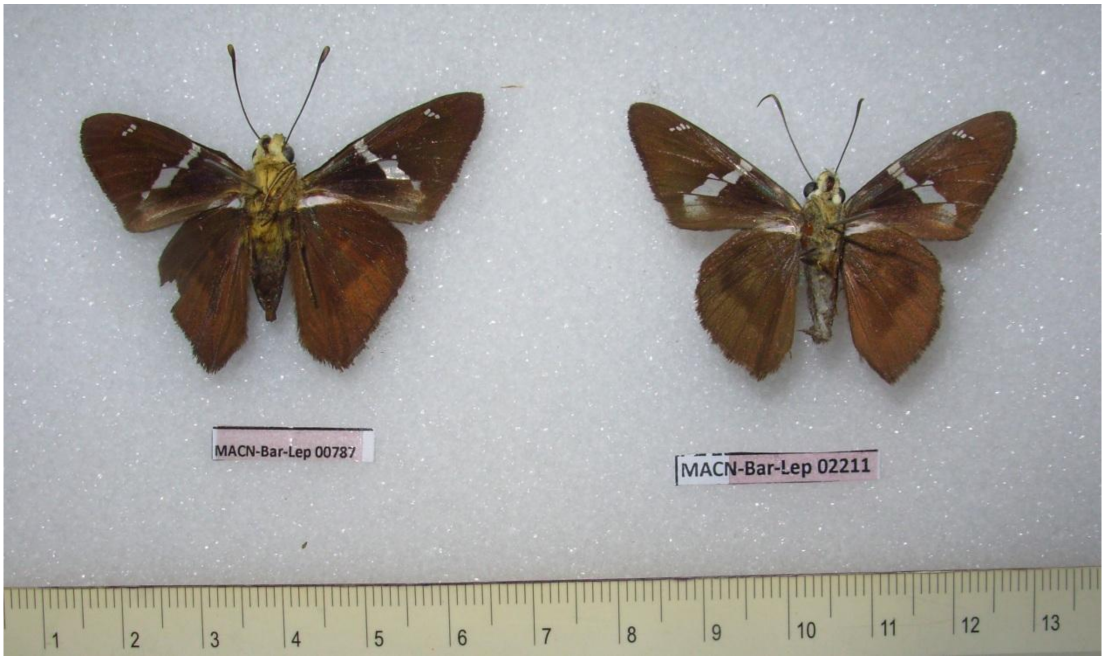

Ventral view of the same two specimens of *A. fulgerator*.

Phoebis argante

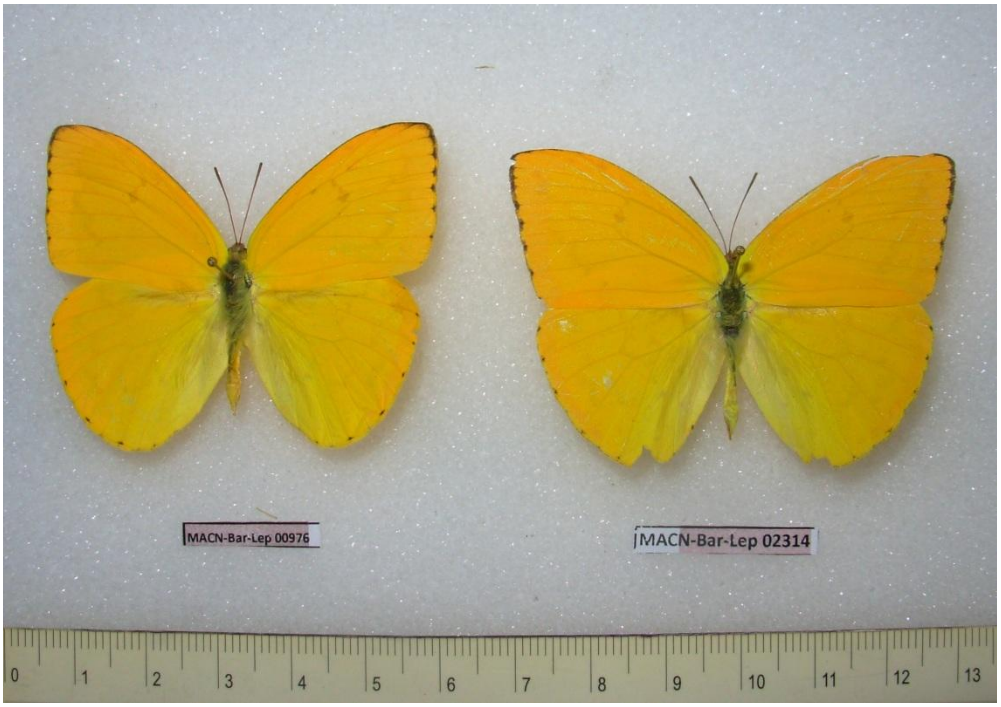

Dorsal view of two specimens of *P. argante* representatives of the two COI lineages found within this species (see Fig 6 in the main text). Left: LEPAR683-11 (male). Right: LEPIG398-11 (male).

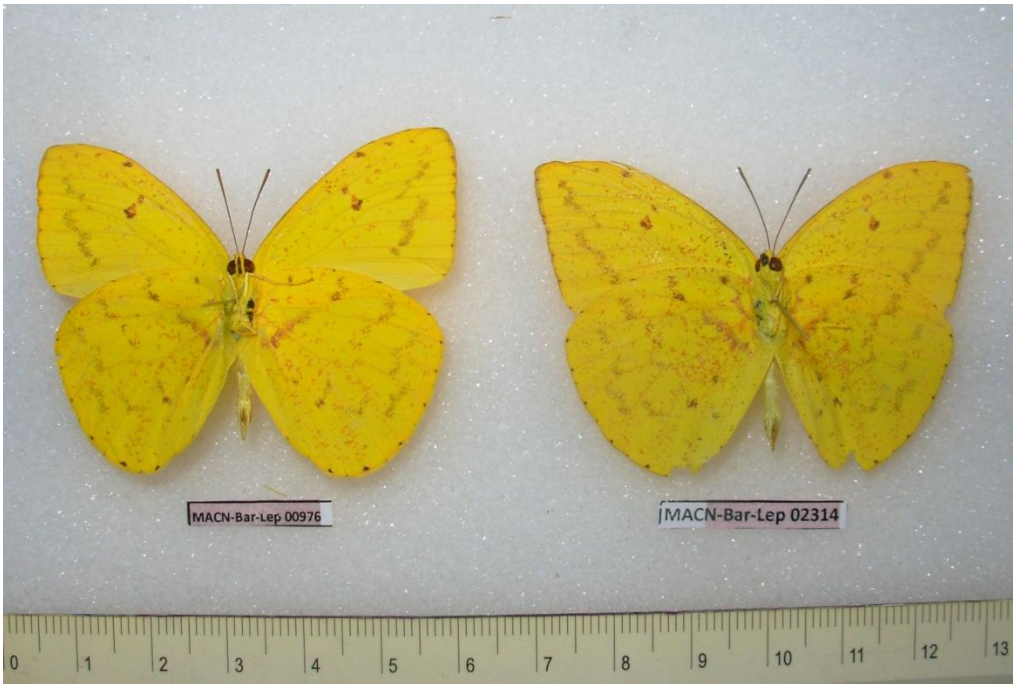

Ventral view of the same two specimens of *P. argante*.

Godartiana muscosa

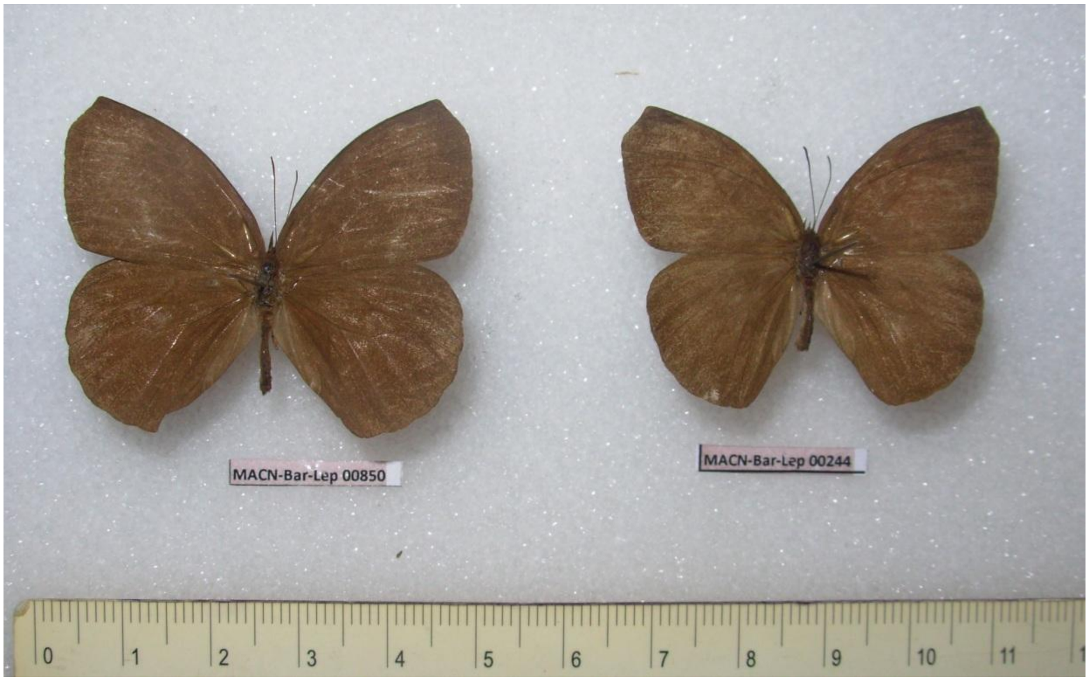

Dorsal view of two specimens of *G. muscosa* representatives of the two COI lineages found within this species (see Fig 6 in the main text). Left: LEPAR623-11 (male). Right: LEPAR330-11 (male).

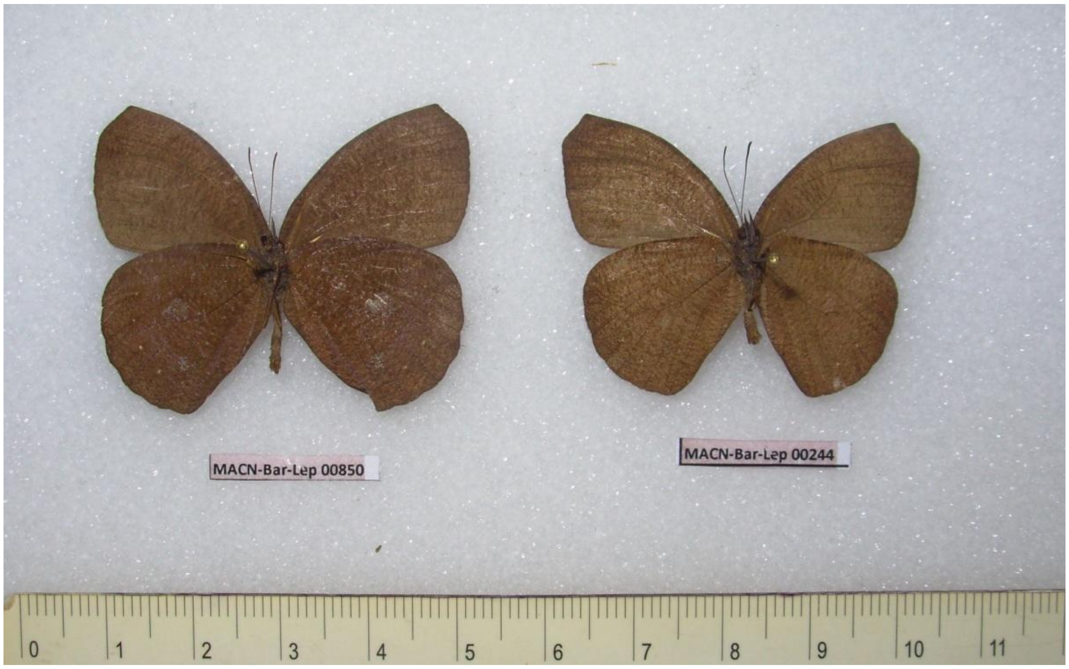

Ventral view of the same two specimens of *G. muscosa*.

Calycopis *sp.* 1, *Calycopis sp.* 2 and *C. caulonia*

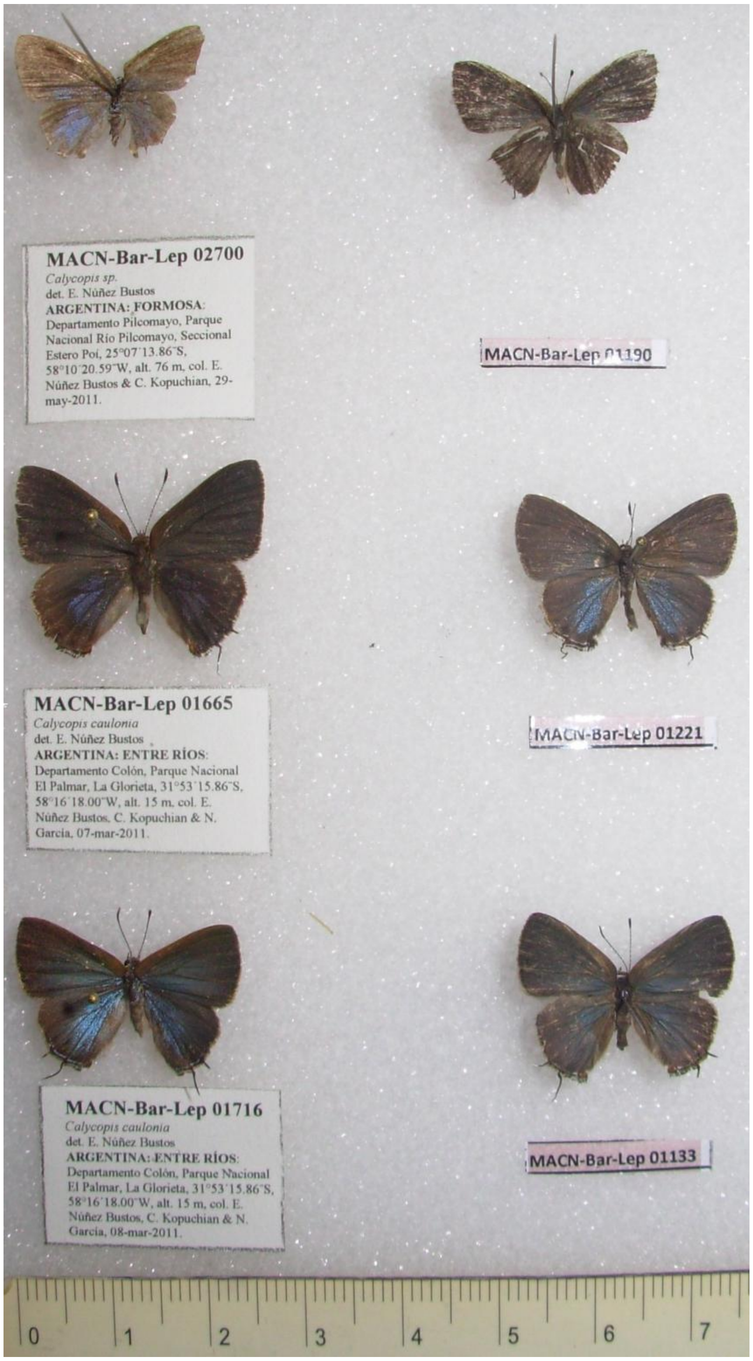

Dorsal view of six specimens of *Calycopis* representing the three taxa discussed in the main text. Top left: *Calycopis sp. 1* (LEPPA345-12). Top right: *Calycopis sp.* 2 (LEPAR812-11). Middle left: *C. caulonia* (LEPAR201-11). Middle right: *Calycopis sp.* 2 (LEPAR836-11). Bottom left: *C. caulonia* (LEPAR221-11). Bottom right: *Calycopis sp.* 2 (LEPAR767-11).

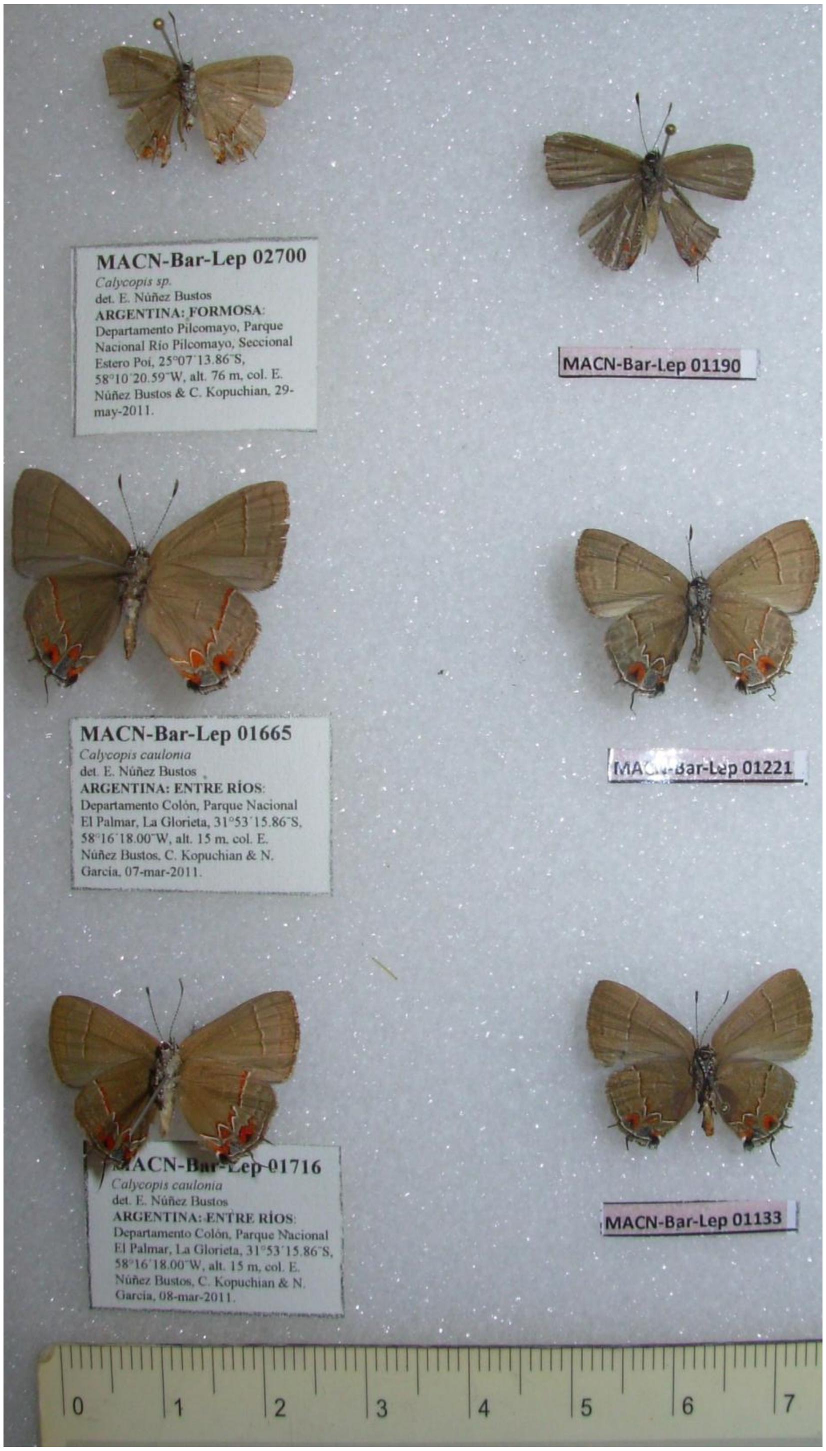

Ventral view of the same specimens of *Calycopis*.

Emesis russula* and *E. mandana

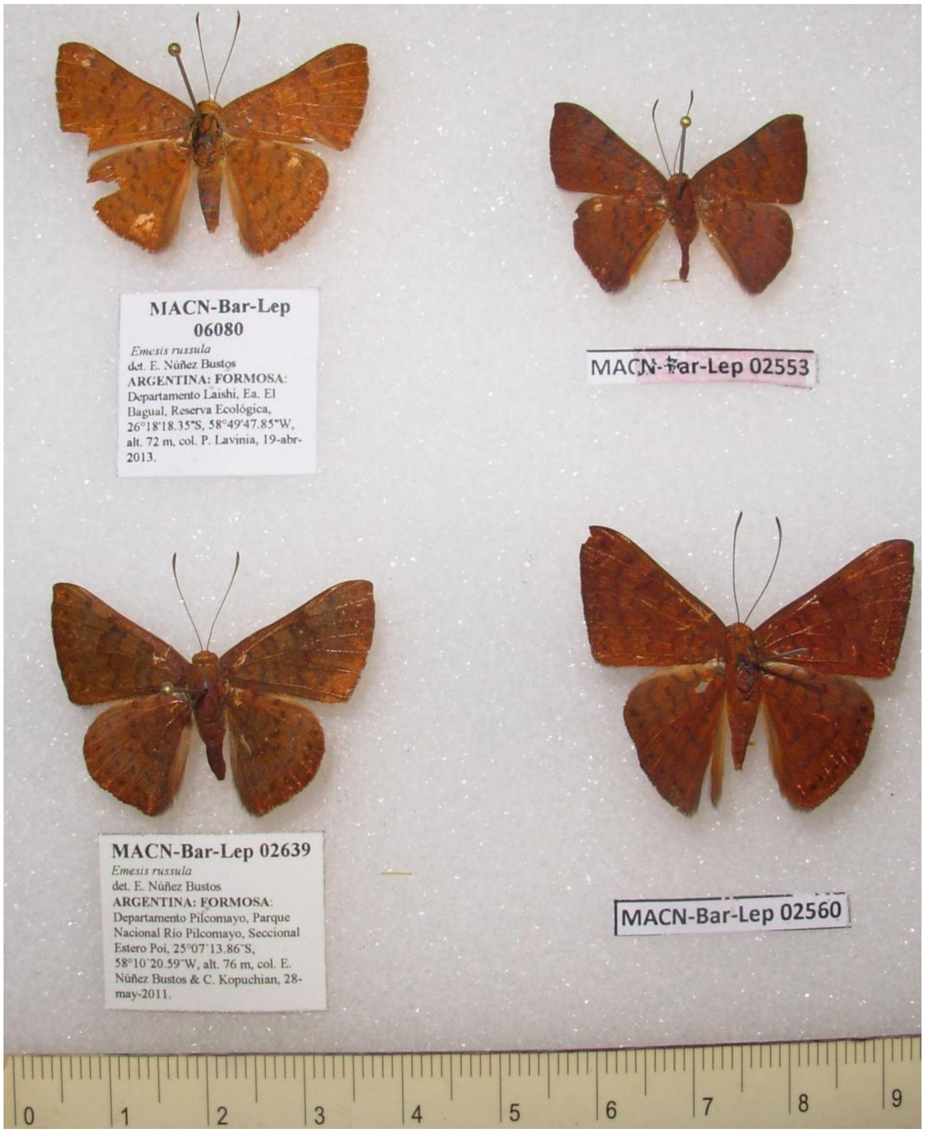

Dorsal view of four specimens representing *E. mandana* and the two COI lineages found within *E. russula*. Top left: *E. russula* from Formosa (LEPAR1013-14). Top right: *E. russula* from Misiones (LEPIG570-11). Bottom left: *E. russula* from Formosa (LEPPA292-12). Bottom right: *E. mandana* from Misiones (LEPIG576-11).

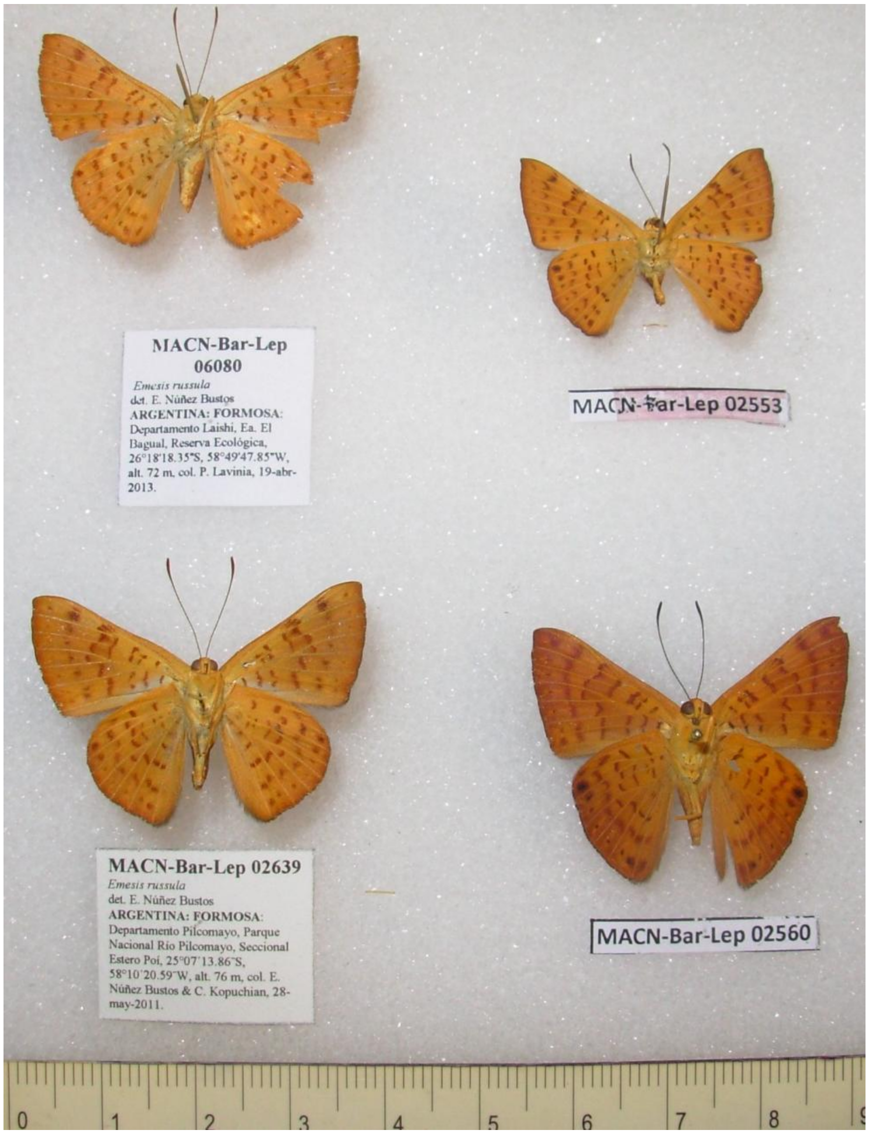

Ventral view of the same specimens of *Emesis*.
